# Polyhedral Ferraboranes with Iron Carbonyl Vertices: Carbonyl Migration Processes in the Iron Tetracarbonyl Derivatives

**DOI:** 10.1021/acs.jpca.3c02944

**Published:** 2023-07-11

**Authors:** Amr A. A. Attia, Alexandru Lupan, R. Bruce King

**Affiliations:** † Faculty of Chemistry and Chemical Engineering, Babes-Bolyai University, ClujNapoca RO-400028, Romania; ‡ Department of Chemistry, University of Georgia, Athens, Georgia 30602, United States

## Abstract

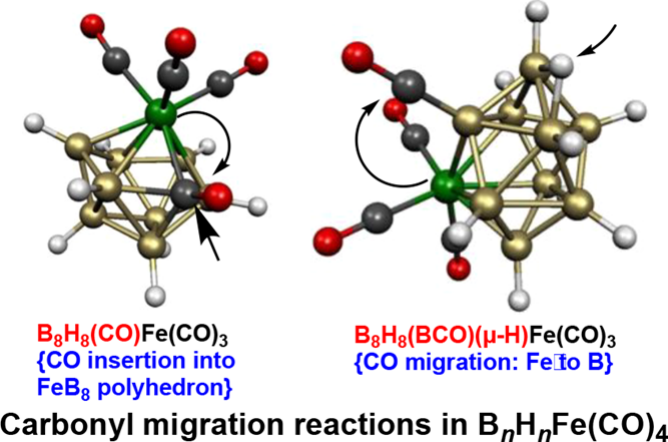

The structures and energetics of the neutral B_*n*–1_H_*n*–1_Fe(*CO*)_*x*_ (*x* = 4, 3) and the dianions [B_*n*–1_H_*n*–1_Fe(CO)_3_]^2–^ (*n* = 6–14) have been investigated by density functional theory. The low-energy structures of the tricarbonyl dianions [B_*n*–1_H_*n*–1_Fe(CO)_3_]^2–^ are all found to have *closo* deltahedral structures in accordance with their 2*n*+2 skeletal electrons. The low-energy structures of the neutral tricarbonyls B_*n*–1_H_*n*–1_Fe(CO)_3_ (*n* = 6–14) with only 2*n* skeletal electrons are based on capped (*n*–1)-vertex *closo* deltahedra (*n* = 6, 7, 8) or *isocloso* deltahedra with a degree 6 vertex for the iron atom. The *closo* 8- and 9-vertex deltahedra are also found in low-energy B_*n*–1_H_*n*–1_Fe(CO)_3_ structures relating to the nondegeneracy of their frontier molecular orbitals. Carbonyl migration occurs in most of the low-energy structures of the tetracarbonyls B_*n*–1_H_*n*–1_Fe(CO)_4_. Thus, migration of a carbonyl group from an iron atom to a boron atom gives *closo* B_*n*–2_H_*n*–2_(BCO)(μ–H)Fe(CO)_3_ structures with a BCO vertex and a hydrogen atom bridging a B–B deltahedral edge. In other low-energy B_*n*–1_H_*n*–1_Fe(CO)_4_ structures, a carbonyl group is inserted into the central *n*-vertex FeB_*n*–1_ deltahedron to give a B_*n*–1_H_*n*–1_(CO)Fe(CO)_3_ structure with a central (*n*+1)-vertex FeCB_*n*–1_ deltahedron that can be an *isocloso* deltahedron or a μ_3_–BH face-capped *n*-vertex FeCB_*n*–2_*closo* deltahedron. Other low-energy B_*n*–1_H_*n*–1_Fe(CO)_4_ structures include B_*n*–1_H_*n*–1_Fe(CO)_2_(μ-CO)_2_ structures with two of the carbonyl groups bridging FeB_2_ faces (*n* = 6, 7, 10) or Fe–B edges (*n* = 12) or structures in which a *closo* B_*n*–1_H_*n*–1_ ligand (*n* = 6, 7, 10, 12) is bonded to an Fe(CO)_4_ unit with exclusively terminal carbonyl groups through B–H–Fe bridges.

## 1 Introduction

**Figure 1:**
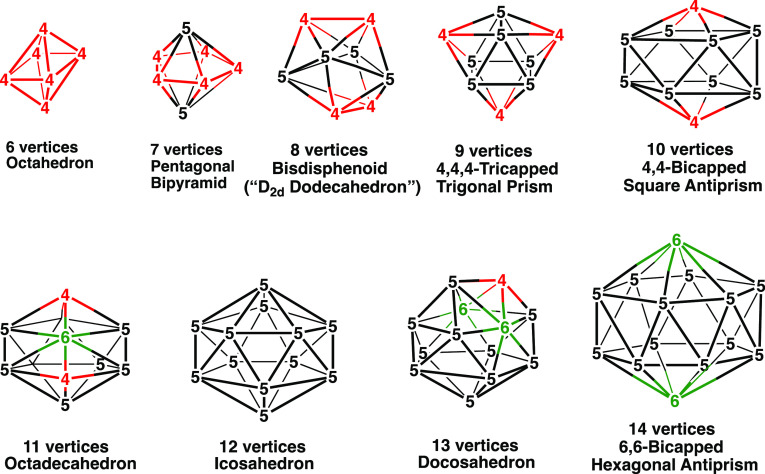
Most spherical *closo* deltahedra having 6 to 14 vertices. Vertices of degrees 4, 5, and 6 are indicated in red, black, and green, respectively.

The pioneering work on polyhedral metallaboranes and metalladicarbaboranes from the research groups of Hawthorne^1^ and Grimes^2^ included many derivatives containing a CpCo (Cp = η^5^-C_5_H_5_) vertex replacing a BH vertex in a borane structure. Both CpCo and BH vertices are donors of two skeletal electrons in the Wade-Mingos electron counting system assuming that each such vertex provides three orbitals for the skeletal bonding^3−5^ as discussed more recently in several articles by Teixidor and collaborators.^6−8^ Thus, cobaltadicarbaboranes of the types CpCoC_2_B_*n*–3_H_*n*–1_ and Cp_2_Co_2_C_2_B_*n*–4_H_*n*–2_ have the favored 2*n* + 2 Wadean skeletal electrons for the most spherical *closo* deltahedral structures with *n* vertices (Figure 1). From the synthetic point of view, the two carbon vertices for such structures can originate from alkynes, RC≡CR, although that leads to thermodynamically less favored structures having carbon atoms at adjacent deltahedral vertices. Note that the deltahedral structures have only sufficient hydrogen atoms (or other external monovalent groups such as alkyl or aryl) for one external E–H bond from each boron and carbon vertex. No “extra” hydrogen atoms bridging deltahedral edges are found in these structures.

**Figure 2:**
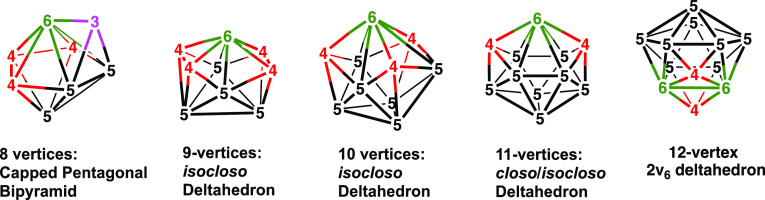
Metallaborane *isocloso* and related deltahedra with 8 to 12 vertices providing at least one degree 6 vertex for a metal atom showing the vertex degrees. Vertices of degrees 3, 4, 5, and 6 are also indicated in pink, red, black, and green, respectively. Note that the 11-vertex *isocloso* deltahedron is the same as the 11-vertex *closo* deltahedron, which necessarily already has a degree 6 vertex.

Iron tricarbonyl vertices, Fe(CO)_3_, are valence isoelectronic with CpCo vertices and likewise donate two skeletal electrons for the Wade-Mingos skeletal bonding scheme.^3−5^ The iron atom in such an Fe(CO)_3_ vertex providing three orbitals for skeletal bonding and three orbitals for σ bonding to the three CO groups can have an allocation of metal valence orbitals similar to that of an octahedral coordination complex FeL_6_ with the polyhedral borane fragment functioning as a tridentate ligand. In this connection, the tricarbonylferradicarbaboranes C_2_B_*n*–3_H_*n*–1_Fe(CO)_3_ have been synthesized having six,^9^ seven,^9,10^ and twelve^11^ vertices, i.e., *n* = 6, 7, and 12, respectively. These *n*-vertex structures all have 2*n* + 2 Wadean skeletal electrons and exhibit the corresponding most spherical *closo* deltahedral structures (Figure 1). However, B_*n*–1_H_*n*–1_Fe(CO)_3_ species without the two C–H vertices have only 2*n* skeletal electrons and thus might be expected to exhibit *isocloso* structures based on deltahedra having a degree 6 vertex for the iron atom (Figure 2).^12−14^

**Figure 3:**
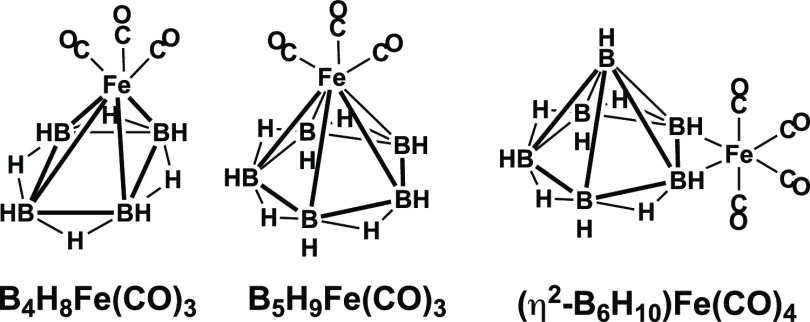
Borane iron carbonyls B_4_H_8_Fe(CO)_3_, B_5_H_9_Fe(CO)_3_, and (η^2^-B_6_H_10_)Fe(CO)_4_.

No examples of B_*n*–1_H_*n*–1_Fe(CO)_3_ systems or their substitution products have been synthesized. The only polyhedral borane iron carbonyls without carbon polyhedral vertices that have been synthesized and characterized at least spectroscopically are hydrogen-rich systems derived from the binary *nido* boranes of the type B_*n*_H_*n*+4_ by replacement of a BH vertex by an isolobal Fe(CO)_3_ vertex (Figure 3). Examples of such species are tetragonal pyramidal^15^ B_4_H_8_Fe(CO)_3_ derived from B_5_H_9_ and pentagonal pyramidal^16,17^ B_5_H_9_Fe(CO)_3_ derived from B_6_H_10_. Hexaborane can also serve as a bidentate ligand in an iron tetracarbonyl complex (η^2^-B_6_H_10_)Fe(CO)_4_ through formation of a B_2_Fe three-center two-electron bond (Figure 3).^18,19^

The experimentally known examples of *closo* deltahedral tricarbonylferraboranes increase the number of Wadean skeletal electrons from 2*n* in a B_*n*–1_H_*n*–1_Fe(CO)_3_ derivative to 2*n* + 2 by replacing two BH vertices with CH vertices to give the corresponding C_2_B_*n*–3_H_*n*–1_Fe(CO)_3_ derivatives. The theoretical study reported in this study uses density functional theory methods to explore the structures and thermochemistry of two other types of tricarbonylferraborane structures having the apparent 2*n* + 2 Wadean skeletal electrons. Thus, a two-electron reduction of neutral B_*n*–1_H_*n*–1_Fe(CO)_3_ to give the corresponding dianion [B_*n*–1_H_*n*–1_Fe(CO)_3_]^2–^ converts the neutral 2*n* skeletal electron system to the 2*n* + 2 skeletal electron dianion. We show that the energetically preferred structures for the [B_*n*–1_H_*n*–1_Fe(CO)_3_]^2–^ dianions are always the most spherical *closo* deltahedra (Figure 1). The only real question of interest is the preferred location of the Fe(CO)_3_ vertex.

This study reports a comprehensive density functional theory analysis of the following types of polyhedral ferraboranes with iron carbonyl vertices organized in the following ways where *n* refers to the number of vertices in the central polyhedron:

(1) The dianions [B_*n*–1_H_*n*–1_Fe(CO)_3_]^2–^ having the 2*n* + 2 skeletal electrons required for a central FeB_*n*–1_*closo* deltahedron (Figure 1). Such ferraborane dianions are unknown experimentally. However, they might be accessible by reductions of the known B_*n*–1_H_*n*+3_Fe(CO)_3_ with bridging hydrogen atoms (Figure 3) with an alkali metal reagent such as potassium on graphite;
(2) The neutral species B_*n*–1_H_*n*–1_Fe(CO)_3_ with iron tricarbonyl vertices having only 2*n* skeletal electrons and possibly accessible through mild oxidation of the above dianions;
(3) The neutral species B_*n*–1_H_*n*–1_Fe(CO)_4_ with iron tetracarbonyl vertices. It might be assumed that adding an “extra” CO group to a B_*n*–1_H_*n*–1_Fe(CO)_3_ derivative having only 2*n* skeletal electrons would provide the “extra” two skeletal electrons for the 2*n* + 2 skeletal electrons normally required for *closo* deltahedral structures (Figure 1). However, this is found to lead to some interesting complications. Having four CO groups on the iron atom leaves only two valence orbitals for skeletal bonding if the iron remains hexacoordinate as in the B_*n*–1_H_*n*–1_Fe(CO)_3_ systems. The topological bonding model^20^ rationalizing the Wade-Mingos 2*n* + 2 skeletal electron rule for *closo* deltahedra (Figure 1)^3−5^ requires three internal orbitals from each vertex atom so that the borane polyhedral fragment effectively remains a tridentate ligand for the metal vertex atom. This is only possible if the iron atom in the Fe(CO)_4_ group effectively has the less favorable coordination number of seven in the B_*n*–1_H_*n*–1_Fe(CO)_4_ derivatives. We find that for many of the low-energy structures in the B_*n*–1_H_*n*–1_Fe(CO)_4_ systems, the “extra” CO group migrates from the iron atom to an adjacent boron atom, leading to a B_*n*–2_B(*CO*)H_*n*–1_Fe(CO)_3_ structure. Donation of the CO lone pair to the boron atom in a B(CO) vertex (i.e., O≡C → B) makes all three boron valence electrons available for skeletal bonding so that a B(CO) vertex becomes a donor of three skeletal electrons. Thus, the B_*n*–2_B(*CO*)H_*n*–1_Fe(CO)_3_ structures with *n* vertices have the favorable 2*n* + 2 skeletal electrons and accordingly are based on the most spherical *closo* deltahedra. We also find examples of low-energy structures in which one or two CO groups have partially migrated away from the Fe(CO)_4_ group to cap F**e**B_2_ faces in the underlying deltahedron.

## 2 Theoretical Methods

The initial model structures are based on various B_*n*_H_*n*_ polyhedral frameworks where systematic substitutions of BH vertices by an Fe(*CO*)_*x*_ unit (*x* = 4, 3) led to 630 initial starting structures of the type B_*n*–1_H_*n*–1_Fe(*CO*)_*x*_ (*n* = 6–14; *x* = 4, 3) (see the Supporting information).

Full geometry optimizations were carried out on the neutral B_*n*–1_H_*n*–1_Fe(*CO*)_*x*_ (*x* = 4, 3) and the dianions [B_*n*–1_H_*n*–1_Fe(CO)_3_]^2–^ (*n* = 6–14) using the B3LYP DFT functional^21−24^ coupled with the double zeta 6-31G(d) basis set. The lowest energy structures were reoptimized at the PBE0/def2-TZVP level of theory.^25^ Single point energy calculations were then performed on the lowest energy structures by using the domain based local pair-natural orbital coupled-cluster method including single and double excitations and perturbative correction for connected triples (DLPNO-CCSD(T)).^26^ The quadruple zeta def2-QZVP basis sets were used in these calculations. The final energies were corrected for zero-point energies taken from the PBE0/def2-TZVP computations.

The nature of the stationary points after optimization was checked by calculations of the harmonic vibrational frequencies. If significant imaginary frequencies were found, the optimization was continued by following the normal modes corresponding to imaginary frequencies to insure that genuine minima were obtained. Highest occupied molecular orbital (HOMO)–lowest unoccupied molecular orbital (LUMO) gaps for the lowest energy structures are provided in the Supporting Information.

All calculations were performed using the Gaussian 09 package^27^ with the default settings for the SCF cycles and geometry optimizations. Single-point DLPNO-CCSD(T) energy calculations were carried out with the ORCA 3.0.3 software package^28^ using very tight convergence criteria.

The B_*n*–1_H_*n*–1_Fe(*CO*)_*x*_ (*x* = 4, 3) (*n* = 6–14) structures are designated as **B(*n*–1)FeC*m*–*x*** where ***n*** is the total number of polyhedral vertices, ***m*** is the total number of CO ligands, and ***x*** is the relative order of the structure on the potential energy scale. However, for the dianions [B_*n*–1_H_*n*–1_Fe(CO)_3_]^2–^ (*n* = 6–14), the shorthand notation **B(*n*–1)FeC*m*M2-*x*** is used. Only the lowest energy and thus potentially chemically significant structures are considered in detail in this study. More comprehensive structural information, including higher energy structures and connectivity information not readily seen in the figures, is given in the Supporting Information.

## 3 Results

### 3.1 Dianions [B_*n*–1_H_*n*–1_Fe(CO)_3_]^2–^ (*n* = 6–14)

**Figure 4:**
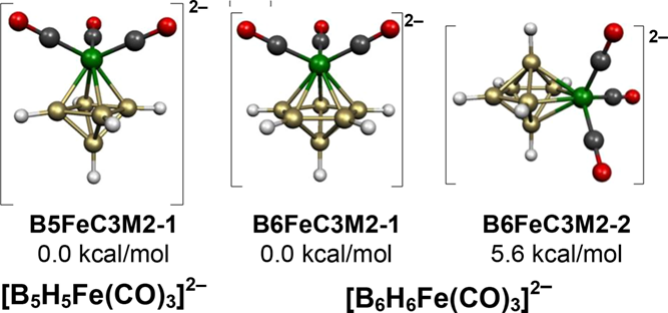
Lowest energy [B_5_H_5_Fe(CO)_3_]^2–^ and [B_6_H_6_Fe(CO)_3_]^2–^ structures.

The dianions [B_*n*–1_H_*n*–1_Fe(CO)_3_]^2–^ (*n* = 6–14) are all predicted to have *closo* deltahedral structures (Figure 1) in accordance with their 2*n* + 2 skeletal electrons. The only issue of interest is the preferred location of the iron vertex in the *closo* deltahedra with nonequivalent vertices. In the octahedral system [B_5_H_5_Fe(CO)_3_]^2–^, all six vertices are equivalent so there is only one low-energy isomer, namely, **B5FeC3M2-1** (Figure 4).

The two lowest energy structures of the 7-vertex [B_6_H_6_Fe(CO)_3_]^2–^ dianion have the expected central FeB_6_ pentagonal bipyramid for this 16-skeletal electron system (Figure 4). The lower energy of these two structures, namely, **B6FeC3M2-1**, has the Fe(CO)_3_ moiety located at a degree 5 axial vertex. The higher energy of these two structures, namely, **B6FeC3M2-2** lying 5.6 kcal/mol above **B6FeC3M2-1**, has the Fe(CO)_3_ moiety located at a degree 4 equatorial vertex.

**Figure 5:**
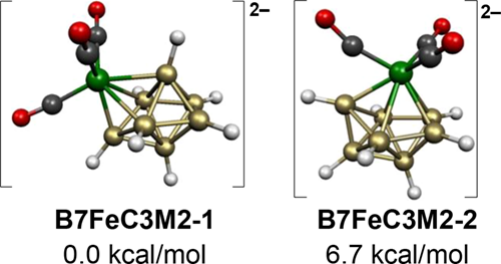
Lowest energy [B_7_H_7_Fe(CO)_3_]^2–^ structures.

The two low-energy structures for the 8-vertex [B_7_H_7_Fe(CO)_3_]^2–^ dianion both have central FeB_7_ bisdisphenoids consistent with their 18 skeletal electrons (=2*n* + 2 for *n* = 8) for a *closo* deltahedron (Figure 5). In the lower energy structure **B7FeC3M2-1**, the Fe(CO)_3_ moiety is located at a degree 4 vertex, which can be considered to resemble locally a cyclobutadiene unit similar to that found in the very stable cyclobutadiene-iron tricarbonyl.^29,30^ In the higher energy [B_7_H_7_Fe(CO)_3_]^2–^ structure **B7FeC3M2-2**, lying 6.7 kcal/mol above **B7FeC3M2-1**, the Fe(CO)_3_ moiety is located at a degree 5 vertex.

**Figure 6:**
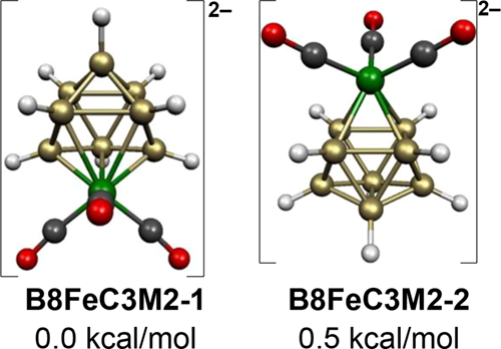
Lowest energy [B_8_H_8_Fe(CO)_3_]^2–^ structures.

The low-energy 9-vertex [B_8_H_8_Fe(CO)_3_]^2–^ dianion structures have the expected central FeB_8_*closo* tricapped trigonal prism (Figure 1). In the lowest energy [B_8_H_8_Fe(CO)_3_]^2–^ structure **B8FeC3M2-1**, the Fe(CO)_3_ moiety is located at one of the degree 5 vertices (Figure 6). However, the isomeric [B_8_H_8_Fe(CO)_3_]^2–^ structure **B8FeC3M2-2** with the Fe(CO)_3_ moiety located at one of the degree 4 vertices of the tricapped trigonal prism has essentially the same energy at only ∼0.5 kcal/mol above **B8FeC3M2-1**. Thus, the location of the iron vertex on the central tricapped trigonal prism in [B_8_H_8_Fe(CO)_3_]^2–^ makes very little energetic difference. The previous theoretical studies on the analogous CpCoB_8_H_8_^2–^ dianion^31^ similarly found the isomeric *closo* structures with the cobalt atom at a degree 4 vertex and with the cobalt atom at a degree 5 vertex to have essentially the same energies within 0.2 kcal/mol.

**Figure 7:**
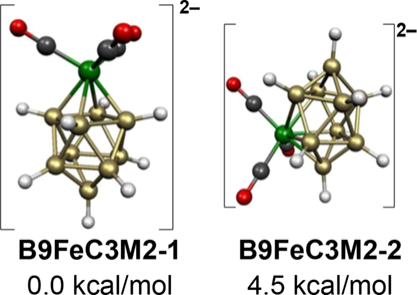
Lowest energy [B_9_H_9_Fe(CO)_3_]^2–^ structures.

Both low-energy structures of the 10-vertex dianion [B_9_H_9_Fe(CO)_3_]^2–^ have central FeB_9_ bicapped square antiprisms consistent with their 22 skeletal electrons (Figure 7). In the lowest energy [B_9_H_9_Fe(CO)_3_]^2–^ structure **B9FeC3M2-1**, the Fe(CO)_3_ moiety is located at one of the degree 4 vertices. The local environment of the iron atom is similar to the very stable cyclobutadiene-iron tricarbonyl,^29^ C_4_H_4_Fe(CO)_3_, or the experimentally known B_4_H_8_Fe(CO)_3_ (Figure 3).^15^ The Fe(CO)_3_ moiety is located at a degree 5 vertex in the higher energy [B_9_H_9_Fe(CO)_3_]^2–^ structure **B9FeC3M2-2**, lying 4.5 kcal/mol in energy above **B9FeC3M2-1**. For the analogous cyclopentadienylcobalt dianion CpCoB_9_H_9_^2–^, the isomers corresponding to **B9FeC3M2-1** and **B9FeC3M2-2** with the cobalt atom at a degree 4 and degree 5 vertex, respectively, are found to have the same energies within ∼1 kcal/mol.^31^

**Figure 8:**
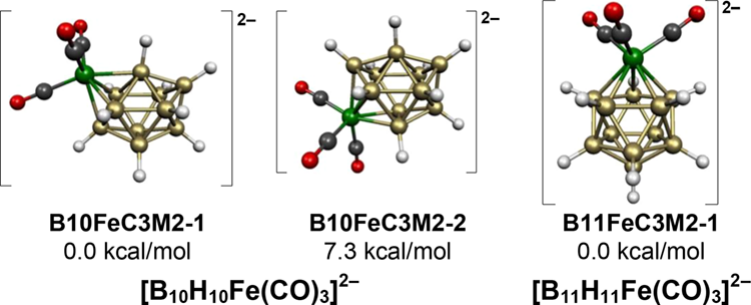
Lowest energy [B_10_H_10_Fe(CO)_3_]^2–^ and [B_11_H_11_Fe(CO)_3_]^2–^ structures.

The two lowest energy structures for the [B_10_H_10_Fe(CO)_3_]^2–^ dianion are based on the most spherical 11-vertex *closo*/*isocloso* deltahedron (Figure 8). The 24 skeletal electrons in these structures suggest that this deltahedron is functioning as a *closo* rather than an *isocloso* deltahedron. The lower energy of these two structures, namely, **B10FeC3M2-1**, has the Fe(CO)_3_ moiety located at one of the degree 4 vertices giving the iron atom a local environment similar to that in cyclobutadiene-iron tricarbonyl^29^ or B_5_H_9_Fe(CO)_3_ (Figure 3).^15^ The higher energy of these structures, namely, **B10FeC3M2-2** lying 7.3 kcal/mol above **B10FeC3M2-1**, has the Fe(CO)_3_ moiety located at a degree 5 vertex not adjacent to the unique degree 6 vertex. For the analogous cyclopentadienylcobalt borane dianion CpCoB_10_H_10_^2–^, the lowest energy structure is an 11-vertex *closo*/*isocloso* deltahedron with the cobalt located at a degree 4 vertex similar to **B10FeC3M2-1**. A CpCoB_10_H_10_^2–^ dianion with the cobalt atom located at a degree 5 vertex two edges away from the unique degree 6 vertex lies 6.7 kcal/mol in energy above the isomer analogous to **B10FeC3M2-1**.^31^ Thus, for the [B_10_H_10_Fe(CO)_3_]^2–^ and CpCoB_10_H_10_^2–^ systems, the two lowest energy structures are completely analogous and have similar relative energies.

Since all 12 vertices of an icosahedron are equivalent like the 6 vertices of an octahedron, there is a unique low-energy [B_11_H_11_Fe(CO)_3_]^2–^ structure **B11FeC3M2-1** with a central FeB_11_ icosahedron (Figure 8).

**Figure 9:**
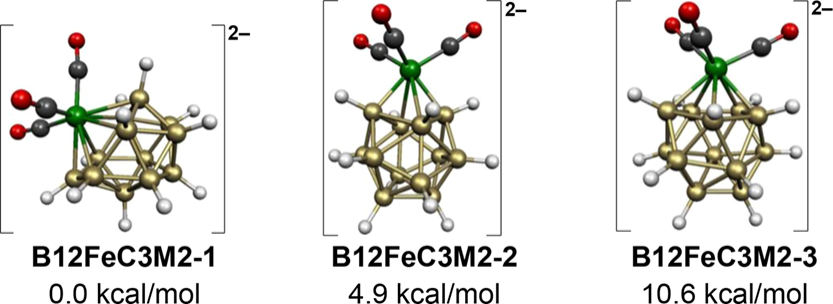
Lowest energy [B_12_H_12_Fe(CO)_3_]^2–^ structures.

The 13-vertex dianion [B_12_H_12_Fe(CO)_3_]^2–^ has 28 skeletal electrons (=2*n* + 2 for *n* = 13) consistent with structures based on the most spherical 13-vertex deltahedron, namely, the docosahedron with two degree 6 vertices, one degree 4 vertex, and 10 degree 5 vertices with the single degree 4 vertex connected to both degree 6 vertices (Figure 1). The lowest energy [B_12_H_12_Fe(CO)_3_]^2–^ structure **B12FeC3M2-1** has the Fe(CO)_3_ moiety located at one of the two degree 6 vertices of this docosahedron (Figure 9). The somewhat higher energy [B_12_H_12_Fe(CO)_3_]^2–^ structure **B12FeC3M2-2**, lying 4.9 kcal/mol above **B12FeC3M2-1**, has the Fe(CO)_3_ moiety located at the unique degree 4 vertex of the FeB_12_ docosahedron. The still higher energy [B_12_H_12_Fe(CO)_3_]^2–^ structure **B12FeC3M2-3**, lying 10.6 kcal/mol in energy above **B12FeC3M2-1**, has the Fe(CO)_3_ moiety located at one of the degree 5 vertices of the docosahedron.

**Figure 10:**
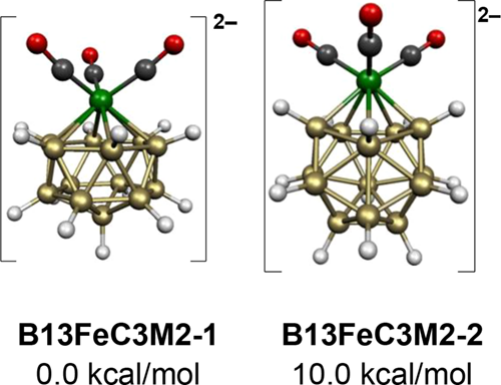
Lowest energy [B_13_H_13_Fe(CO)_3_]^2–^ structures.

The low-energy structures of the 30-skeletal electron 14-vertex dianion [B_13_H_13_Fe(CO)_3_]^2–^ are based on the bicapped hexagonal antiprism, which is the most spherical *closo* 14-vertex deltahedron (Figure 1). In the lowest energy structure **B13FeC3M2-1**, the Fe(CO)_3_ unit is located at one of the two degree 6 vertices of the bicapped hexagonal antiprism (Figure 10). In the higher energy dianion structure **B13FeC3M2-2**, lying 10.0 kcal/mol above **B13FeC3M2-1**, the Fe(CO)_3_ moiety is located at one of the 12 equiv degree 5 vertices of the bicapped hexagonal antiprism.

### 3.2 Neutral Tricarbonyls B_*n*–1_H_*n*–1_Fe(CO)_3_ (*n* = 6–14)

**Figure 11:**
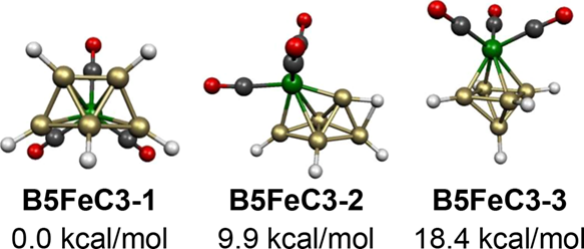
Lowest energy structures for the neutral B_5_H_5_Fe(CO)_3_.

The neutral 6-vertex B_5_H_5_Fe(CO)_3_ system has 12 skeletal electrons (=2*n* for *n* = 6). The expected B_5_Fe polyhedron for this system is the bicapped tetrahedron similar to that found experimentally for the likewise 12-skeletal electron system^32^ Os_6_(CO)_18_. In fact, the two lowest energy B_5_H_5_Fe(CO)_3_ structures are bicapped tetrahedra (Figure 11). The lowest energy such structure **B5FeC3-1** has the Fe(CO)_3_ moiety located at one of the degree 5 vertices, whereas the higher energy structure **B5FeC3-2**, lying 9.9 kcal/mol in energy above **B5FeC3-1**, has the Fe(CO)_3_ moiety located at one of the degree 4 vertices and a hydrogen bridge between a degree 3 and a degree 4 vertex. The still higher energy B_5_H_5_Fe(CO)_3_ structure **B5FeC3-3**, lying at 18.4 kcal/mol has a central FeB_5_ octahedron rather than a bicapped tetrahedron.

**Figure 12:**
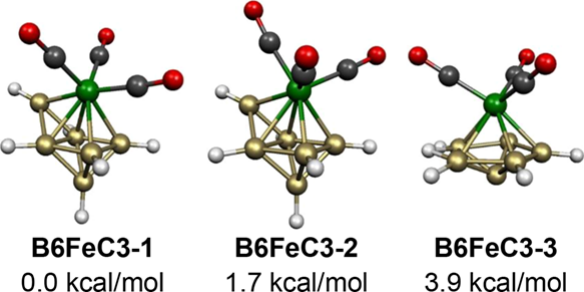
Lowest energy neutral B_6_H_6_Fe(CO)_3_ structures.

The two lowest energy 7-vertex B_6_H_6_Fe(CO)_3_ structures have a central capped octahedron consistent with their 14 skeletal electrons (=2*n* for *n* = 7). In the lower energy of these structures, namely, **B6FeC3-1**, the Fe(CO)_3_ moiety occupies a degree 5 vertex (Figure 12). The other capped octahedral B_6_H_6_Fe(CO)_3_ structure, namely, **B6FeC3-2** lying only 1.7 kcal/mol in energy above **B6FeC3-1**, differs from **B6FeC3-1** only by rotation of the Fe(CO)_3_ unit relative to the boron capping vertex.

The next B_6_H_6_Fe(CO)_3_ structure in terms of energy, namely, **B6FeC3-3**, lying still only 3.9 kcal/mol above **B6FeC3-1**, has a central FeB_6_ pentagonal bipyramid with the distance between the axial iron atom and the opposite likewise axial boron atom being reduced to a bonding distance of 2.336 Å, which is only ∼0.2 Å longer than the shortest Fe–B distance to a boron atom in the equatorial B_5_ pentagon (Figure 12). This additional Fe–B(axial) bonding through the center of this squashed FeB_6_ pentagonal bipyramid in **B6FeC3-3** can compensate for the presence of only 14 rather than 16 skeletal electrons. Also, in **B6FeC3-3**, the hydrogen atom on one of the equatorial pentagon boron atoms moves to bridge a pentagonal B–B edge with B–H distances of ∼1.36 Å.

**Figure 13:**
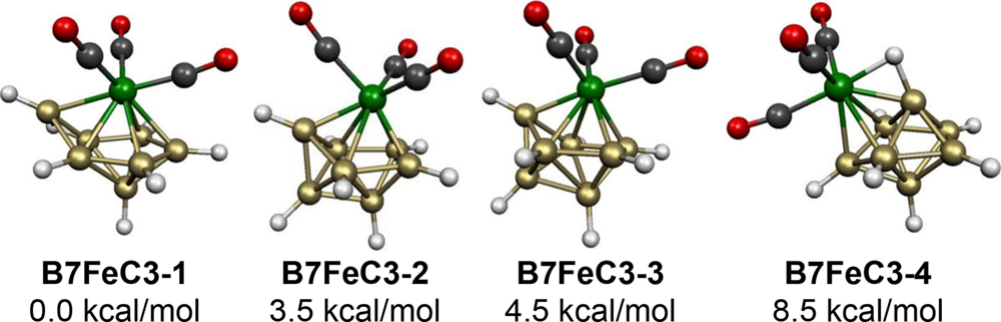
Lowest energy B_7_H_7_Fe(CO)_3_ structures.

Four low-energy structures were found for the 8-vertex 16-skeletal electron system B_7_H_7_Fe(CO)_3_ (Figure 13). The lowest energy such structure **B7FeC3-1** has a central FeB_7_ capped pentagonal bipyramid with the Fe(CO)_3_ moiety located at the unique degree 6 vertex. The central pentagonal bipyramid in this structure is consistent with its 16 skeletal electrons. The next two B_7_H_7_Fe(CO)_3_ structures, namely, **B7FeC3-2** and **B7FeC3-3** lying 3.5 and 4.5 kcal/mol, respectively, in energy above **B7FeC3-1**, are closely related structures with a central FeB_7_ bisdisphenoid having the iron atom located at a degree 5 vertex. The 16 skeletal electrons for these two bisdisphenoidal B_7_H_7_Fe(CO)_3_ structures rather than 18 skeletal electrons for a *closo* bisdisphenoid are reasonable in light of the nondegeneracy of the frontier orbitals of the bisdisphenoid.^33^ The fourth B_7_H_7_Fe(CO)_3_ structure **B7FeC3-4**, lying 8.5 kcal/mol in energy above **B7FeC3-1**, also has a central FeB_7_ bisdisphenoid but with the iron atom located at a degree 4 vertex. In **B7FeC3-4**, one of the hydrogen atoms has migrated from a degree 4 vertex adjacent to the iron atom to form an Fe–H–B bridge with an Fe–H distance of 1.652 Å and a B–H distance of 1.310 Å. This bridging hydrogen atom indirectly draws an otherwise nonbonding iron lone pair into the skeletal bonding, effectively giving **B7FeC3-4** a skeletal electron count of 18 electrons for a *closo* bisdisphenoid.

**Figure 14:**
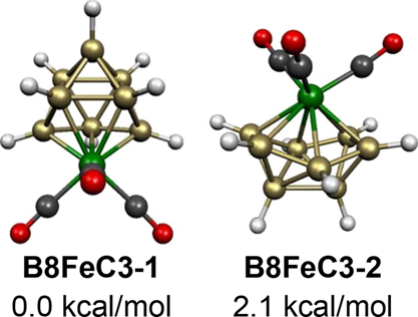
Lowest energy B_8_H_8_Fe(CO)_3_ structures.

Both *closo* and *isocloso* structures were found for the 18-skeletal electron systems B_8_H_8_Fe(CO)_3_ corresponding to 2*n* for *n* = 9 (Figure 14). The lowest energy structure **B8FeC3-1** has the iron atom located at one of the degree 5 vertices of the *closo* tricapped trigonal prism. Note that a stable tricapped trigonal prism structure can have only 18 skeletal electrons as well as 20 skeletal electrons (=2*n* + 2 for *n* = 9) because of the nondegeneracy of its frontier molecular orbitals.^33^ The other low-energy B_8_H_8_Fe(CO)_3_ structure **B8FeC3-2**, lying only 2.1 kcal/mol above **B8FeC3-1**, has the 9-vertex *isocloso* structure (Figure 2) expected for an 18-skeletal electron system. In **B8FeC3-2**, the iron atom is located at the unique degree 6 vertex in accordance with expectation. For the analogous cyclopentadienylcobalt system CpCoB_8_H_8_, the *closo* tricapped trigonal prism is found to be the lowest energy structure.^31^ The *isocloso* isomer of CpCoB_8_H_8_ with a degree 6 cobalt vertex analogous to **B8FeC3-2** is predicted to lie 9.2 kcal/mol in energy above the *closo* structure.

**Figure 15:**
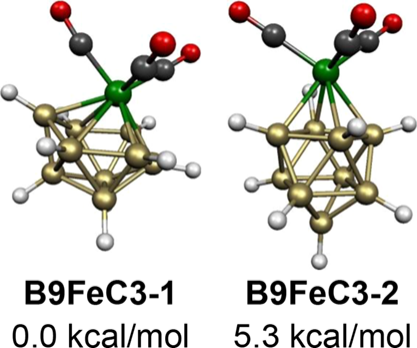
Lowest energy B_9_H_9_Fe(CO)_3_ structures.

The lowest energy B_9_H_9_Fe(CO)_3_ structure **B9FeC3-1** has a central 10-vertex *isocloso* deltahedron (Figure 2) with the Fe(CO)_3_ moiety located at the unique degree 6 vertex consistent with its 20 skeletal electrons (=2*n* for *n* = 10) (Figure 15). The higher energy B_9_H_9_Fe(CO)_3_ structure **B9FeC3-2**, lying 5.3 kcal/mol in energy above **B9FeC3-1**, has a central FeB_9_ bicapped square antiprism with the Fe(CO)_3_ moiety located at a degree 4 vertex. The hydrogen atom on one of the boron vertices adjacent to the iron atom in **B9FeC3-2** moves within the bonding distance of the iron atom to form a B–H–Fe bridge with a B–H distance of 1.326 Å and an Fe–H distance of 1.636 Å. This bridging hydrogen atom brings an otherwise external iron lone electron pair into the skeletal bonding so that **B9FeC3-2** is effectively a 22-skeletal electron system consistent with its *closo* bicapped square antiprism geometry. For the analogous cyclopentadienylcobalt system CpCoB_9_H_9_, the *isocloso* structure analogous to **B9FeC3-1** was found to be the lowest energy structure by a more substantial margin with the lowest energy *closo* structure lying 26.3 kcal/mol in energy above the lowest energy *isocloso* structure.^31^

**Figure 16:**
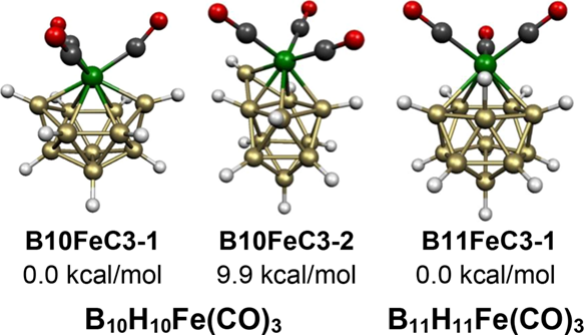
Lowest energy B_10_H_10_Fe(CO)_3_ and B_11_H_11_Fe(CO)_3_ structures.

The lowest energy B_10_H_10_Fe(CO)_3_ structure **B10FeC3-1** has the central most spherical 11-vertex deltahedron that can function either as a *closo* or an *isocloso* deltahedron (Figures 1 and 2). The Fe(CO)_3_ moiety is located at the unique degree 6 vertex in accordance with the expectation (Figure 16). The 11-vertex deltahedron in this B_10_H_10_Fe(CO)_3_ structure **B10FeC3-1** has 22 skeletal electrons (=2*n* for *n* = 11) and thus functions as an *isocloso* rather than a *closo* deltahedron. A CpCoB_10_H_10_ structure analogous to **B10FeC3-1** based on the 11-vertex *closo*/*isocloso* deltahedron with the cobalt atom at the unique degree 6 vertex is found to be the lowest energy structure by more than 19 kcal/mol.^31^

The next higher energy B_10_H_10_Fe(CO)_3_ structure **B10FeC3-2**, lying 9.9 kcal/mol in energy above **B10FeC3-1**, is a very different structure than **B10FeC3-1**. The central FeB_10_ deltahedron is an FeB_9_ bicapped square antiprism in which a face including the degree 4 vertex is capped by the tenth boron atom (Figure 16). The Fe(CO)_3_ moiety in **B10FeC3-2** is located at the degree 4 vertex of the original bicapped square antiprism that is connected to the capping boron vertex to become a degree 5 vertex. The 22 skeletal electrons in **B10FeC3-2** are consistent with the requirement for the central bicapped square antiprism as the *closo* 10-vertex deltahedron (Figure 1).

Icosahedral structures are so favorable in polyhedral borane chemistry that even the lowest energy neutral 12-vertex B_11_H_11_Fe(CO)_3_ structure **B11FeC3-1** with only 24 apparent skeletal electrons rather than 26 skeletal electrons also has a central FeB_11_ icosahedron (Figure 16). However, the terminal hydrogen atom on one of the boron atoms adjacent to the iron atom moves to a B–H–Fe bridging position with an Fe–H distance of 1.620 Å and a B–H distance of 1.356 Å. This B–H–Fe bridge brings an otherwise nonbonding iron lone pair into the skeletal bonding so that B_11_H_11_Fe(CO)_3_ effectively has the required 26 skeletal electrons for its icosahedral structure.

**Figure 17:**
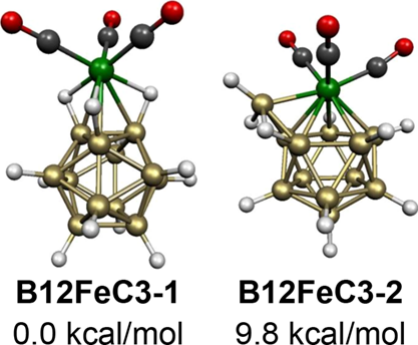
Lowest energy B_12_H_12_Fe(CO)_3_ structures.

The icosahedral B_12_H_12_ unit is such a favorable one that it functions as a ligand to an Fe(*CO*)_*n*_ unit through B–H–Fe bridges in the lowest energy 13-vertex B_12_H_12_Fe(CO)_3_ structure **B12FeC3-1**. (Figure 17). Thus, in **B12FeC3-1**, the B_12_H_12_ ligand coordinates to the Fe(CO)_3_ unit as a tridentate ligand with Fe–H distances of ∼1.79 Å, B–H distances of ∼1.28 Å, and Fe–B distances of ∼2.21 Å. If the B_12_H_12_^2–^ ligand is considered to be the very stable dianion, then, the iron atom in **B12FeC3-1** must be *d*^6^ Fe(II). Thus, **B12FeC3-1** can be considered as a closed-shell octahedral Fe(II) complex with the tridentate B_12_H_12_^2–^ ligand coordinating to the iron atom through three B–H bonds coming from one of the triangular faces of the B_12_ icosahedron.

An energetically competitive totally different B_12_H_12_Fe(CO)_3_ structure is found, namely, **B12FeC3-2**, lying 9.8 kcal/mol in energy above **B12FeC3-1** (Figure 17). The central FeB_12_ deltahedron in **B12FeC3-2** is a capped icosahedron with the Fe(CO)_3_ moiety located at one of the degree 6 vertices resulting from the capping process. The Fe(CO)_3_ moiety and the 12 BH vertices are each donors of two skeletal electrons, thus making **B12FeC3-2** a 26-skeletal electron system consistent with the central FeB_11_ icosahedron.

**Figure 18:**
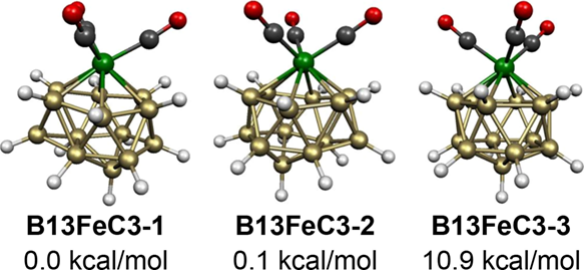
Lowest energy B_13_H_13_Fe(CO)_3_ structures.

The FeB_13_ deltahedron found in the two lowest energy structures for the 14-vertex B_13_H_13_Fe(CO)_3_ system with only 28 skeletal electrons (=2*n* for *n* = 14) can be derived from the bicapped hexagonal antiprism by a diamond-square-diamond rearrangement involving an edge connecting a degree 6 vertex with an adjacent degree 5 vertex (Figure 18). This generates a new deltahedron with three degree 6 vertices and one degree 4 vertex along with 10 degree 5 vertices. Such a 14-vertex deltahedron could be considered as an analogue of an *isocloso* deltahedron. Structures **B13FeC3-1** and **B13FeC3-2** are closely related and have essentially the same energy. A higher energy B_13_H_13_Fe(CO)_3_ structure **B13FeC3-3**, lying 10.9 kcal/mol above **B13FeC3-1**, has an undistorted central bicapped hexagonal antiprism with the Fe(CO)_3_ moiety located at one of the degree 6 vertices.

### 3.3 Neutral Tetracarbonyls B_*n*–1_H_*n*–1_Fe(CO)_4_ (*n* = 6–14)

**Figure 19:**
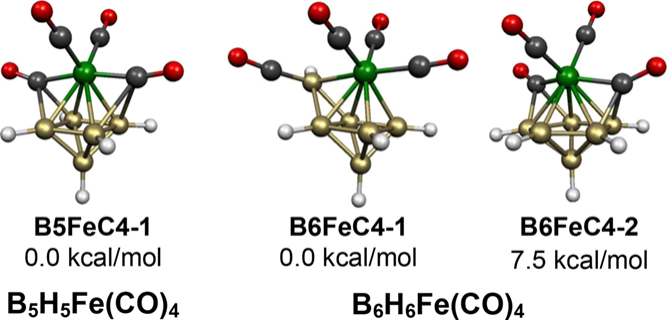
Lowest energy B_5_H_5_Fe(CO)_4_ and B_6_H_6_Fe(CO)_4_ structures.

Adding the two electrons provided by an “extra” carbonyl group to the tricarbonyls B_*n*–1_H_*n*–1_Fe(CO)_3_ (*n* = 6–14) with only 2*n* skeletal electrons might be expected to give the corresponding tetracarbonyls B_*n*–1_H_*n*–1_Fe(CO)_4_ with 2*n* + 2 skeletal electrons like the dianions [B_*n*–1_H_*n*–1_Fe(CO)_3_]^2–^ and thus exhibiting *closo* deltahedra (Figure 1). However, in most B_*n*–1_H_*n*–1_Fe(CO)_4_ systems, the fourth carbonyl group in the lowest energy structures does not remain on the iron atom as a terminal ligand but instead migrates either to an adjacent boron atom or becomes a vertex in a new (*n*+1)-vertex FeCB_*n*–1_ deltahedron. For example, in the 6-vertex B_5_H_5_Fe(CO)_4_ structure **B5FeC4-1**, two of the carbonyl groups of the Fe(CO)_4_ unit are terminal groups and the remaining two carbonyl groups bridge FeB_2_ triangular faces (Figure 19).

The lowest energy structure for the 7-vertex B_6_H_6_Fe(CO)_4_ with 16 skeletal electrons (=2*n* + 2 for *n* = 7), namely, **B6FeC4-1**, is not the *closo* pentagonal bipyramid but instead a capped octahedron (Figure 19). In **B6FeC4-1**, one of the carbonyl groups has migrated from the iron atom to an adjacent boron atom so that this species can be formulated as B_5_H_5_(HBCO)Fe(CO)_3_. The HBCO vertex in **B6FeC4-1** is a donor of two skeletal electrons similar to each of the five BH vertices and the Fe(CO)_3_ moiety. This makes **B6FeC4-1** a 14-skeletal electron system consistent with its central FeB_5_ octahedron. The tendency of the carbonyl group to migrate from iron to the capping boron vertex in **B6FeC4-1** with retention of the terminal hydrogen atom appears to be related to the degree 3 of this vertex.

The higher energy B_6_H_6_Fe(CO)_4_ structure **B6FeC4-2**, lying 7.5 kcal/mol in energy above **B6FeC4-1**, has the expected central FeB_6_ pentagonal bipyramid with the iron atom at one of the degree 5 axial vertices (Figure 19). Two of the carbonyl groups of the Fe(CO)_4_ unit remain bonded as terminal groups to the iron atom, whereas the remaining two carbonyl groups migrate to an adjacent FeB_2_ face to become face-bridging μ_3_-CO groups. Thus, the 7-vertex structure **B6FeC4-2**, described more specifically as B_6_H_6_Fe(CO)_2_(μ_3_-CO)_2_, is closely related to the 6-vertex structure **B5FeC4-1**, which can be described as B_5_H_5_Fe(CO)_2_(μ_3_-CO)_2_.

**Figure 20:**
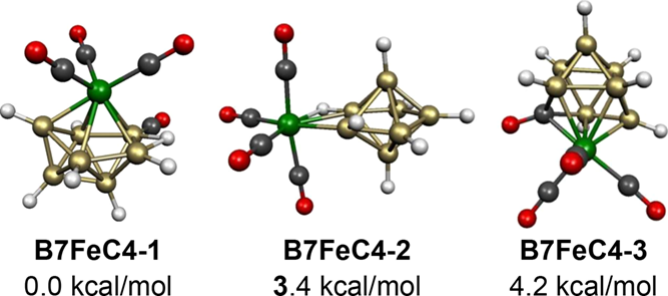
Lowest energy B_7_H_7_Fe(CO)_4_ structures.

The 8-vertex *closo* deltahedron, namely, the bisdisphenoid, has nondegenerate frontier orbitals^33^ and thus can be suitable for both 16- and 18-skeletal electron systems as indicated by the stability of the binary boron chloride B_8_Cl_8_, which has only 16 skeletal electrons.^34,35^ The lowest energy structure **B7FeC4-1** of the 18-skeletal electron system B_7_H_7_Fe(CO)_4_ indeed has a central FeB_7_ bisdisphenoid (Figure 20). However, one of the carbonyl groups of the Fe(CO)_4_ moiety has migrated to an adjacent boron atom. This carbonyl migration drives the hydrogen atom originally bonded to the boron atom receiving the carbonyl group to a bridging position across an adjacent B–B bond. Coordination of the carbonyl group to the boron atom in **B7FeC4-1** allows all three valence electrons of that boron to become skeletal electrons so that the BCO vertex is a donor of three skeletal electrons. There still remains the hydrogen atom bridging the BCO vertex to an adjacent vertex to donate an additional electron. With the Fe(CO)_3_ vertex as well as the remaining six BH vertices each functioning as donors of two skeletal electrons, structure **B7FeC4-1** has the expected 18-skeletal electrons (=2*n* + 2 for *n* = 8) for a *closo* bisdisphenoid.

The B_7_H_7_Fe(CO)_4_ structure **B7FeC4-2**, lying 3.4 kcal/mol in energy above **B7FeC4-1**, can be dissected formally into a neutral B_7_H_7_ ligand functioning as a two-electron donor to an Fe(CO)_4_ unit, thereby giving the iron atom the favored 18-electron configuration (Figure 20). This can also be regarded formally as a substitution product of the stable Fe(CO)_4_I_2_ in which the *closo*-B_7_H_7_^2–^ dianion has displaced two iodide ions. The B_7_H_7_^2–^ dianion is a trihapto ligand forming an Fe–H bond of length 1.800 Å and two Fe–B bonds of lengths 2.095 and 2.228 Å to the iron atom.

The third low-energy B_7_H_7_Fe(CO)_4_ structure, namely, **B7FeC4-3** lying 4.2 kcal/mol in energy above **B7FeC4-1**, is a still different type of structure in which one of the carbonyl groups of the Fe(CO)_4_ vertex has migrated to an adjacent vertex in a central FeCB_7_ tricapped trigonal prism (Figure 20). The Fe(CO)_3_ vertex, the carbonyl vertex, and the seven B–H vertices are all two-electron donors making **B7FeC4-3** an 18-skeletal electron system corresponding to 2*n* for *n* = 9 (counting, of course, the carbon atom of the carbonyl vertex). A 2*n* as well a 2*n* + 2 *closo* skeletal electron count is reasonable for a tricapped trigonal prism in view of the nondegeneracy of its frontier orbitals.^33^

**Figure 21:**
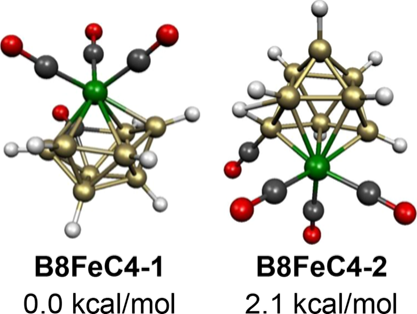
Lowest energy B_8_H_8_Fe(CO)_4_ structures.

The B_8_H_8_Fe(CO)_4_ system has 20 skeletal electrons corresponding to 2*n* + 2 for *n* = 9 for the 9-vertex *closo* deltahedron, namely, the tricapped trigonal prism (Figure 1). However, carbonyl migration occurs in both low-energy B_8_H_8_Fe(CO)_4_ structures to give other central deltahedra. In the lowest energy B_8_H_8_Fe(CO)_4_ structure **B8FeC4-1**, one of the carbonyl groups becomes a deltahedral vertex, leading to the 10-vertex *isocloso* deltahedron (Figure 2) with the iron atom located at the unique degree 6 vertex in accordance with expectation (Figure 21). The 20 skeletal electrons in **B8FeC4-1** corresponding to 2*n* for *n* = 10 are consistent with the skeletal electron requirement for a central 10-vertex FeCB_8_*isocloso* deltahedron.

The carbonyl migration process in the other low-energy 9-vertex B_8_H_8_Fe(CO)_4_ structure **B8FeC4-2**, lying only 2.1 kcal/mol above **B8FeC4-1**, is of a different type involving migration from iron to an adjacent boron atom (Figure 21). The situation in **B8FeC4-2** is similar to that in the lowest energy 8-vertex B_7_H_7_Fe(CO)_4_ structure **B7FeC4-1** (Figure 20). Thus the carbonyl migration in **B8FeC4-2** drives the hydrogen bonded to the boron receiving the carbonyl group into a bridging position to an adjacent boron atom. As for **B7FeC4-1** this carbonyl migration in **B8FeC4-2** preserves the 20 skeletal electrons (=2*n* + 2 for *n* = 9) of the B_7_H_7_Fe(CO)_4_ system consistent with its central *closo* 9-vertex tricapped trigonal prism. In **B8FeC4-2**, the iron atom is located at a degree 5 vertex, and the BCO unit is located at one of the three degree 4 vertices.

**Figure 22:**
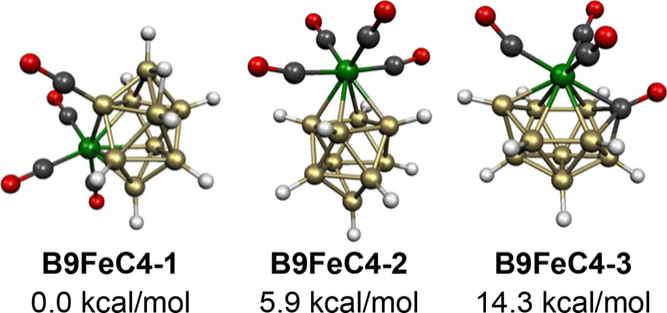
Lowest energy B_9_H_9_Fe(CO)_4_ structures.

The two lowest energy 10-vertex B_9_H_9_Fe(CO)_4_ structures have an FeB_9_ bicapped square antiprism consistent with their 22 skeletal electrons (=2*n* + 2 for *n* = 10) for this 10-vertex *closo* deltahedron (Figure 22). In the lowest energy such structure **B9FeC4-1**, the iron atom is located at one of the degree 5 vertices, but one of the carbonyl groups originally bonded to iron has migrated to an adjacent degree 5 boron vertex. The hydrogen originally bonded to the boron vertex receiving the carbonyl group in **B9FeC4-1** becomes a bridging hydrogen from a degree 4 boron vertex to a degree 5 boron vertex. This carbonyl migration process preserves the 22 skeletal electron configuration. In the higher energy bicapped square antiprism B_9_H_9_Fe(CO)_4_ structure **B9FeC4-2**, lying 5.9 kcal/mol in energy above **B9FeC4-1**, the Fe(CO)_4_ moiety is located at one of the degree 4 vertices and there is no carbonyl migration.

The next higher energy B_9_H_9_Fe(CO)_4_ structure **B9FeC4-3**, lying 14.3 kcal/mol in energy above **B9FeC4-1**, is based on a central *closo*/*isocloso* 11-vertex FeCB_9_ deltahedron (Figures 1 and 2) in which one of the carbonyl groups bonded to the iron atom is inserted in the central deltahedral structure (Figure 22). The remaining Fe(CO)_3_ moiety occupies the unique degree 6 vertex and the carbonyl carbon occupies one of the two degree 4 vertices. Since the Fe(CO)_3_, carbonyl, and BH vertices are each two skeletal electron donors, **B9FeC4-3** is a 22 skeletal electron system, suggesting that the central 11-vertex FeCB_9_ deltahedron is functioning as an *isocloso* deltahedron, as shown in Figure 2.

**Figure 23:**
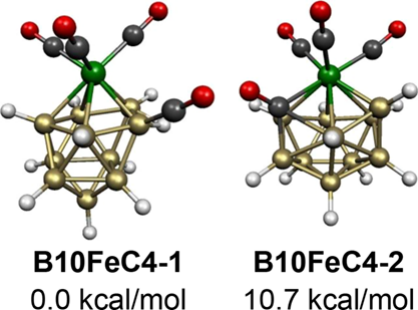
Lowest energy B_10_H_10_Fe(CO)_4_ structures.

The lowest energy structure of the 11-vertex B_10_H_10_Fe(CO)_4_ system **B10FeC4-1** is unusual since it is not a deltahedron but a polyhedron with a single tetragonal B_4_ face (Figure 23). One of the carbonyl groups of the original Fe(CO)_4_ unit has migrated to an adjacent boron vertex that is part of the tetragonal face leaving an Fe(CO)_3_ moiety to occupy a degree 5 vertex. The hydrogen attached to the boron vertex receiving the carbonyl group moves to bridge one of the B–B edges of the tetragonal B_4_ face. Since the single Fe(CO)_3_ vertex and the nine BH vertices are each two skeletal electron donors, the BCO vertex is a three skeletal electron donor, and the bridging hydrogen atom a source of an additional skeletal electron, the B_10_H_10_Fe(CO)_4_ structure **B10FeC4-1** becomes a 24-skeletal electron system (=2*n* + 2 for *n* = 11). This skeletal electron count is that expected for a 11-vertex *closo* deltahedron. The actual 11-vertex FeB_10_ polyhedron found in **B10FeC4-1** can be derived from the 11-vertex *closo* deltahedron (Figure 1) by breaking an edge connecting its unique degree 6 vertex with one of the adjacent degree 5 vertices to form the tetragonal face.

The next B_10_H_10_Fe(CO)_4_ structure **B10FeC4-2**, lying 10.7 kcal/mol in energy above **B10FeC4-1**, has a central 12-vertex FeCB_10_ deltahedron in which one of the carbonyl groups of the Fe(CO)_4_ unit is inserted into an 11-vertex deltahedron (Figure 23). In order to provide a degree 4 vertex for this carbonyl carbon atom, the central FeCB_10_ deltahedron cannot be the regular icosahedron which has only degree 5 vertices. Instead, the 12-vertex deltahedron has two degree 4 vertices including one for the carbon atom, and two degree 6 vertices including one for the iron atom. This 12-vertex deltahedron is found experimentally in the dirhodium complexes Cp*_2_Rh_2_B_10_H_9_(OH) and Cp*_2_Rh_2_B_10_H_8_(OH)_2_ shown by X-ray crystallography to have the rhodium atoms located at the two degree 6 vertices in these 24 skeletal electron systems.^36^ Structure **B10FeC4-2** is also a 24 skeletal electron system since the Fe(CO)_3_ vertex, the carbonyl vertex, and the ten BH vertices each contribute two skeletal electrons to this system.

**Figure 24:**
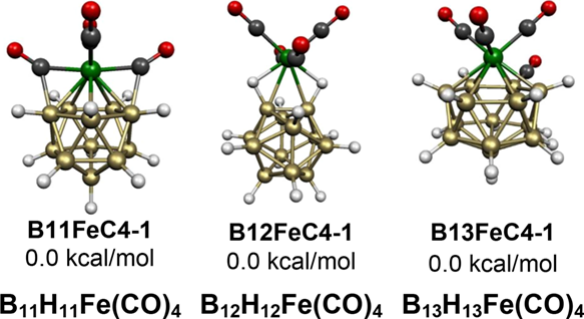
Lowest energy B_11_H_11_Fe(CO)_4_ and B_13_H_13_Fe(CO)_4_ structures.

The iron vertex in the icosahedral structure of the neutral 12-vertex species B_11_H_11_Fe(CO)_4_, namely, **B11FeC4-1**, bears only two terminal carbonyl groups (Figure 24). The remaining two carbonyl groups in **B11FeC4-1** bridge the iron atom to adjacent boron atoms. Furthermore, the icosahedral B_12_H_12_ unit is such a favorable one that it functions as a ligand to an Fe(*CO*)_*n*_ unit through B–H–Fe bridges in the lowest energy neutral 13-vertex B_12_H_12_Fe(CO)_4_ structure **B12FeC4-1**. Thus, in the B_12_H_12_Fe(CO)_4_ structure **B12FeC4-1**, the B_12_H_12_ ligand coordinates to the Fe(CO)_4_ unit as a bidentate ligand through two B–H–Fe bridges with Fe–H distances of ∼1.78 Å, B–H distances of ∼1.28 Å, and Fe–B distances of ∼2.30 Å.

The lowest energy structure of the 30-skeletal electron neutral 14-vertex system B_13_H_13_Fe(CO)_4_, namely, **B13FeC4-1**, has a central FeB_13_ bicapped hexagonal antiprism (Figure 24). However, in **B13FeC4-1**, one of the carbonyl groups of the Fe(CO)_4_ unit has migrated to an adjacent boron atom with the concurrent migration of the terminal hydrogen originally on this boron atom to a bridging position. This process is analogous to that discussed above for the structures **B10FeC4-1**, **B9FeC4-1**, **B8FeC4-2**, and **B7FeC4-1** with 11, 10, 9, and 8 vertices, respectively, in which the carbonyl migration from iron to boron does not affect the skeletal electron count.

## 4 Discussion

The tricarbonyl dianions [B_*n*–1_H_*n*–1_Fe(CO)_3_]^2–^ have the 2*n* + 2 skeletal electrons expected for *closo* deltahedral structures. Therefore, it is not surprising that the central FeB_*n*–1_ polyhedra in all of the low-energy [B_*n*–1_H_*n*–1_Fe(CO)_3_]^2–^ structures are the corresponding most spherical *closo* deltahedra (Figure 1). For 8- to 14-vertex [B_*n*–1_H_*n*–1_Fe(CO)_3_]^2–^ structures with a degree 4 vertex in the central FeB_*n*–1_ deltahedron, the preferred location of the Fe(CO)_3_ group is the degree 4 vertex. This gives the iron atom in such structures a local environment similar to that in cyclobutadiene-iron tricarbonyl,^29^ C_4_H_4_Fe(CO)_3_, or the known borane iron carbonyl^15^ B_4_H_8_Fe(CO)_3_. In the smaller [B_6_H_6_Fe(CO)_3_]^2–^ system, the local environment of the iron atom at a degree 4 vertex deviates significantly from that in C_4_H_4_Fe(CO)_3_ and B_4_H_8_Fe(CO)_3_ so that the isomeric structure with the iron atom located at a degree 5 vertex is energetically favored. In the supraicosahedral [B_*n*–1_H_*n*–1_Fe(CO)_3_]^2–^ systems (*n* = 13, 14), the lowest energy structures have the Fe(CO)_3_ group located at a degree 6 vertex.

The neutral tricarbonyls B_*n*–1_H_*n*–1_Fe(CO)_3_ have only 2*n* skeletal electrons and thus might be expected to have either central *isocloso* FeB_*n*–1_ deltahedra (Figure 2) or (*n*–1)-vertex c*loso* FeB_*n*–2_ deltahedra with one face capped by the remaining BH group as a degree 3 vertex. For the smallest B_*n*–1_H_*n*–1_Fe(CO)_3_ systems, capped *closo* (*n*–1)-vertex deltahedral structures are energetically preferred as exemplified by the bicapped tetrahedral (≡capped trigonal bipyramidal) structures **B5FeC3-1** and **B5FeC3-2** for B_5_H_5_Fe(CO)_3_, the capped octahedral structures **B6FeC3-1** and **B6FeC3-2** for B_6_H_6_Fe(CO)_3_, the capped pentagonal bipyramidal structure **B7FeC3-1** for B_7_H_7_Fe(CO)_3_, the capped bicapped square antiprismatic structure **B10FeC3-2** for B_10_H_10_Fe(CO)_3_, and the capped icosahedral B_12_H_12_Fe(CO)_3_ structure **B12FeC3-2** for B_12_H_12_Fe(CO)_3_. The *closo* 8- and 9-vertex deltahedra, namely, the bisdisphenoid and tricapped trigonal prism, have nondegenerate frontier molecular orbitals^33^ and thus can be preferred structures for not only 2*n* + 2 but also 2*n* skeletal electron systems. In the borane iron tricarbonyls, this is exemplified by the bisdisphenoidal B_7_H_7_Fe(CO)_3_ structures **B7FeC3-2**, **B7FeC3-3**, and **B7FeC3-4** and the tricapped trigonal prismatic B_8_H_8_Fe(CO)_3_ structure **B8FeC3-1**. *Isocloso* deltahedra (Figure 2) begin to appear in the 9-vertex systems as exemplified by the 9-vertex **B8FeC3–2** and the 10-vertex **B9FeC3-1**. The 11-vertex structure **B10FeC3-1** is also of this type having the iron atom at the unique degree 6 vertex of the most spherical 11-vertex deltahedron (Figures 1 and 2) that can be either a *closo* or *isocloso* deltahedron. The 14-vertex deltahedron found in the B_13_H_13_Fe(CO)_3_ structures **B13FeC3-1** and **B13FeC3-2** has three degree 6 vertices and one degree 4 vertex as well as 10 degree 5 vertices and is derived from the bicapped hexagonal antiprism by a diamond-square-diamond rearrangement. This deltahedron may be regarded as the equivalent of an *isocloso* deltahedron for 14-vertex structures.

The 12-vertex B_11_H_11_Fe(CO)_3_ structure **B11FeC3-1** has a central FeB_11_ icosahedron despite having only 24 (=2*n* for *n* = 12) rather than the 26 (=2*n* + 2 for *n* = 12) skeletal electrons expected for the most spherical *closo* icosahedron. However, a terminal hydrogen on a boron atom adjacent to the iron atom in **B11FeC3-1** moves over to form a B–H–Fe bridge with the iron atom, thereby drawing an otherwise external iron lone pair into the skeletal bonding and effectively adding two skeletal electrons. Thus, this bridging hydrogen atom in **B11FeC3-1** makes this a 26 skeletal electron structure consistent with its central FeB_11_ icosahedron. A hydrogen atom forms a similar B–H–Fe bridge in the 10-vertex B_9_H_9_Fe(CO)_3_ structure **B9FeC3-2** with a central *closo* deltahedron, namely, the bicapped square antiprism.

The special stability of boron icosahedra leads to a special structure **B12FeC3-1** as the lowest energy structure for the 13-vertex B_12_H_12_Fe(CO)_3_ system. Thus, **B12FeC3-1** has an icosahedral B_12_H_12_ tridentate ligand bonding to an external Fe(CO)_3_ moiety through three B–H–Fe bridges. The iron atom in **B12FeC3-1** effectively has octahedral coordination through the three carbonyl groups and the three B–H–Fe bridges.

The Wade-Mingos rules^3−5^ for skeletal bonding in polyhedral boranes and related clusters assume three internal orbitals from each vertex atom for skeletal bonding. Thus, Fe(CO)_3_ and BH vertices are both donors of two skeletal electrons so that the neutral species B_*n*–1_H_*n*–1_Fe(CO)_3_ are 2*n* skeletal electron systems. Adding another CO group to give B_*n*–1_H_*n*–1_Fe(CO)_4_ derivatives would lead to a 2*n* + 2 Wadean skeletal electron system, thereby suggesting most spherical *closo* central FeB_*n*–1_ deltahedra (Figure 1). However, such B_*n*–1_H_*n*–1_Fe(CO)_4_ structures retaining all four carbonyl groups as terminal ligands bonded to the iron atom require involvement of seven iron orbitals from its nine valence orbital sp^3^d^5^ manifold corresponding to an Fe(CO)_4_ vertex acting as a donor of four skeletal electrons. Partial or complete migration of one of the carbonyl groups of the Fe(CO)_4_ moiety to an adjacent atom can reduce the iron orbital requirement for cluster bonding to a more favorable six, namely, one orbital for the Fe–C σ bond to each of the three remaining carbonyl groups as well as three internal orbitals for deltahedral cluster skeletal bonding. The stability of deltahedral B_*n*–1_H_*n*–1_ species as dianions suggests a formal Fe(II) oxidation state for the iron atom in neutral B_*n*–1_H_*n*–1_Fe(*CO*)_*n*_ derivatives. Thus migration of one of the carbonyl groups away from the iron atom in such B_*n*–1_H_*n*–1_Fe(CO)_4_ derivatives leads to a local environment of the iron vertex as distorted octahedral d^6^ Fe(II) corresponding to a closed shell iron configuration.

**Figure 25:**
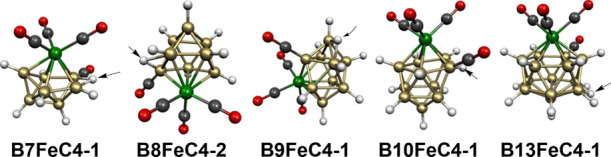
Comparison of the five low-energy B_*n*–2_H_*n*–2_(BCO)(μ–H)Fe(CO)_3_ structures showing by arrows the location of the μ–H hydrogen atoms bridging B–B edges.

The most common type of CO migration in the B_*n*–1_H_*n*–1_Fe(CO)_4_ structures is from the iron atom to an adjacent boron atom to give a BCO vertex. Such a BCO vertex becomes a donor of three skeletal electrons since the CO lone pair provides both electrons required for the external bonding of the boron vertex. The hydrogen that was originally on the boron vertex receiving the carbonyl group becomes a bridge across a B–B edge. The resulting B_*n*–2_H_*n*–2_(BCO)(μ–H)Fe(CO)_3_ structures become 2*n* + 2 skeletal electron systems with the BH and Fe(CO)_3_ vertices providing two skeletal electrons each, the BCO vertex providing three skeletal electrons, and the bridging hydrogen atom an extra skeletal electron. The low-energy B_*n*–2_H_*n*–2_(BCO)(μ–H)Fe(CO)_3_ structures are **B7FeC4-1**, **B8FeC4-2**, **B9FeC4-1**, **B10FeC4-1**, and **B13FeC4-1** having 8, 9, 10, 11, and 14 vertices, respectively, in their central FeB_*n*–1_ deltahedra (Figure 25). Not surprisingly, because of their 2*n* + 2 skeletal electrons, their central FeB_*n*–1_ deltahedra are the most spherical *closo* deltahedra (Figure 1). Such B_*n*–2_H_*n*–2_(BCO)(μ–H)Fe(CO)_3_ structures are the lowest energy structures for the 8, 10, 11, and 14-vertex systems.

**Figure 26:**
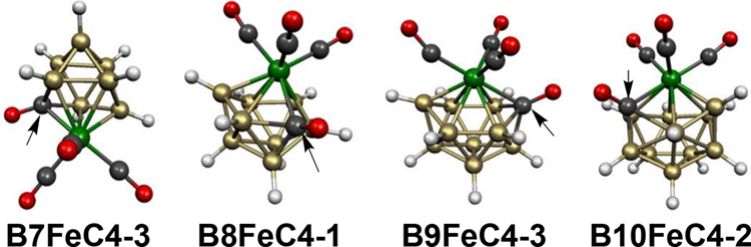
Comparison of the four low-energy B_*n*–1_H_*n*–1_(CO)Fe(CO)_3_ structures showing by arrows the location of the CO deltahedral vertices.

Another type of CO migration away from the iron atom in B_*n*–1_H_*n*–1_Fe(CO)_4_ structures involves insertion of a CO group into the central FeB_*n*–1_ deltahedron to give a central (*n* + 1)-vertex FeCB_*n*–1_ deltahedron. Since the CO vertex as well as the BH and Fe(CO)_3_ vertices are each donors of two skeletal electrons, these B_*n*–1_H_*n*–1_(CO)Fe(CO)_3_ systems are 2(*n* + 1) skeletal electron systems and thus might be expected to exhibit either (*n* + 1)-vertex *isocloso* structures with the iron atom at a degree 6 vertex (Figure 2) or a capped *n-*vertex deltahedral structure. Low-energy B_*n*–1_H_*n*–1_(CO)Fe(CO)_3_ structures having a CO vertex in a central (*n* + 1)-vertex FeCB_*n*–1_ deltahedron include **B7FeC4-3**, **B8FeC4-1**, **B9FeC4-3**, and **B10FeC4-2** (Figure 26). The central FeCB_*n*–1_ deltahedra in **B8FeC4-1**, **B9FeC4-3**, and **B10FeC4-2** are the corresponding (*n* + 1)-vertex *isocloso* deltahedra (Figure 2) with the iron atom at a degree 6 vertex. Note that the 11-vertex *isocloso* deltahedron is the same as the 11-vertex *closo* deltahedron, and the 12-vertex *isocloso* deltahedron necessarily has two degree 6 vertices, one of which is occupied by the iron atom in **B10FeC4-2**. Also, note that in the only lowest-energy B_*n*–1_H_*n*–1_(CO)Fe(CO)_3_ structure **B8FeC4-1**, the central 10-vertex *isocloso* deltahedron has been shown to be a particularly favorable *isocloso* deltahedron in systematic theoretical studies on other 2*n* skeletal electron systems such as Cp_2_Fe_2_C_2_B_*n*–4_H_*n*–4_^37^ and CpMC_2_B_*n*–3_H_*n*–3_ (M = Mn, Re).^38^

The B_*n*–2_H_*n*–2_(BCO)(μ–H)Fe(CO)_3_ and B_*n*–1_H_*n*–1_(CO)Fe(CO)_3_ structures are the only examples of low-energy structures where a CO group has migrated completely away from the iron atom fully severing the Fe–CO bond. In another type of low-energy B_*n*–1_H_*n*–1_Fe(CO)_4_ structure, two of the carbonyl groups of the Fe(CO)_4_ moiety become bridging carbonyl groups either across Fe–B edges or FeB_2_ faces. Such B_*n*–1_H_*n*–1_Fe(CO)_2_(μ–CO)_2_ structures include **B5FeC4-1**, **B6FeC4-1**, and **B11FeC4-1** in which the central FeB_*n*–1_ deltahedra are the corresponding most spherical *closo* deltahedra (Figure 1), namely, the 6-vertex octahedron, the 7-vertex pentagonal bipyramid, and the 12-vertex icosahedron, respectively. In the two smaller such structures, namely, **B5FeC4-1** and **B6FeC4-1**, the bridging carbonyl groups are μ_3_-CO groups bridging FeB_2_ faces. However, in **B11FeC4-1**, the bridging carbonyl groups are μ-CO groups bridging FeB edges. The 10-vertex B_9_H_9_Fe(CO)_4_ structure **B9FeC4-2** with a central bicapped square antiprism may also be considered in this category with two weakly face semibridging μ_3_-CO groups with B–C distances of 2.26, 2.27, and 2.27 Å.

A few low-energy B_*n*–1_H_*n*–1_Fe(CO)_4_ structures are found containing an intact Fe(CO)_4_ unit with all terminal carbonyl groups. Such structures may be formally regarded as an Fe(CO)_4_ complex of a B_*n*–1_H_*n*–1_ ligand. In all such structures, the B_*n*–1_H_*n*–1_ unit has a *closo* deltahedral structure (Figure 1) and thus formally can be considered as the dianion B_*n*–1_H_*n*–1_^2–^. This makes the Fe(CO)_4_ unit formally an Fe(II) dication Fe(CO)_4_^2+^. Thus B_*n*–1_H_*n*–1_Fe(CO)_4_ complexes can be generated formally be replacing the two halides in Fe(CO)_4_X_2_ (X = Cl, Br, I) derivatives with the B_*n*–1_H_*n*–1_^2–^ dianion. Alternatively, such complexes can be obtained formally by replacing one carbonyl group in Fe(CO)_5_ by a neutral B_*n*–1_H_*n*–1_ ligand, which is formally a two-electron donor ligand, to preserve the favored 18-electron configuration of the iron atom. The two low-energy structures of this type are **B7FeC4-2**, in which a B_7_H_7_ pentagonal bipyramid is bonded to the Fe(CO)_4_ unit through an Fe–H–B bridge and a direct Fe–B bond (Figure 20) and **B12FeC4-1**, in which a B_12_H_12_ icosahedron is bonded to the Fe(CO)_4_ unit through two Fe–H–B bridges (Figure 24).

## 5 Summary

The dianions [B_*n*–1_H_*n*–1_Fe(CO)_3_]^2–^ (*n* = 6–14) have 2*n* + 2 skeletal electrons. This is consistent with the observation that all of their low-energy structures exhibit the most spherical *closo* deltahedral structures expected for this skeletal electron count without any examples of carbonyl migration.

The situation with the neutral B_*n*–1_H_*n*–1_Fe(*CO*)_*n*_ (*n* = 3, 4) derivatives is more complicated. The B_*n*–1_H_*n*–1_Fe(CO)_3_ (*n* = 6–14) tricarbonyl systems have only 2*n* skeletal electrons and thus might be expected to exhibit low-energy capped (*n*–1)-vertex *closo* deltahedral structures or *isocloso* structures with a degree 6 vertex for the iron atom. Low-energy capped deltahedral structures are found for the smaller systems having 6 to 8 vertices, whereas *isocloso* structures are found for the systems with 9 to 11 vertices. In addition, the *closo* 8- and 9-vertex deltahedra appear in low-energy B_*n*–1_H_*n*–1_Fe(CO)_3_ structures consistent with the nondegeneracy of their frontier orbitals.^33^ The lowest energy B_13_H_13_Fe(CO)_3_ structure is a novel 14-vertex *isocloso* deltahedron.

The stability of boron icosahedra leads to special types of low-energy B_*n*–1_H_*n*–1_Fe(CO)_3_ (*n* = 12, 13) structures for the 12- and 13-vertex tricarbonyl systems. Thus, the lowest energy B_11_H_11_Fe(CO)_3_ structure has a central FeB_11_ icosahedron with a bridging hydrogen along one of the Fe–B edges. The lowest energy B_12_H_12_Fe(CO)_3_ structure has an icosahedral B_12_H_12_ ligand coordinated to the Fe(CO)_3_ moiety through three B–H–Fe bridges.

The B_*n*–1_H_*n*–1_Fe(CO)_4_ (*n* = 6–14) tetracarbonyl systems have 2*n* + 2 skeletal electrons, suggesting the most spherical *closo* deltahedral structures. However, in most of the low-energy B_*n*–1_H_*n*–1_Fe(CO)_4_ structures, one of the carbonyl groups migrates from the iron atom to give structures of one of the following two types:

(1) Migration from an iron atom to a boron atom gives a B_*n*–2_H_*n*–2_(BCO)(μ-H)Fe(CO)_3_ structure with a BCO vertex and a hydrogen atom bridging a B–B deltahedral edge. Such a carbonyl migration retains the 2*n* + 2 skeletal electron count and leads to structures with central FeB_*n*–1_*closo* deltahedra.
(2) Insertion of the carbonyl group into the central *n*-vertex FeB_*n*–1_ deltahedron to give a B_*n*–1_H_*n*–1_(CO)Fe(CO)_3_ structure with a central (*n* + 1)-vertex FeCB_*n*–1_ deltahedron having 2(*n* + 1) skeletal electrons. This corresponds either to an *n-*vertex *closo* FeCB_*n*–2_ deltahedron with a face capped by a degree 3 boron vertex or to an (*n* + 1)-vertex *isocloso* deltahedron.

Other low-energy B_*n*–1_H_*n*–1_Fe(CO)_4_ structures are of the following types:

(1) B_*n*–1_H_*n*–1_Fe(CO)_2_(μ–CO)_2_ structures in which two of the carbonyl groups bridge FeB_2_ faces (*n* = 6, 7, 10) or Fe–B edges (*n* = 12) of a central *n*-vertex FeB_*n*–1_*closo* deltahedron.
(2) (B_*n*–1_H_*n*–1_)Fe(CO)_4_ structures (*n* = 6, 7, 10, 12) in which a *closo* deltahedral B_*n*–1_H_*n*–1_ ligand is bonded through B–H–Fe bridges to an Fe(CO)_4_ moiety with exclusively terminal carbonyl groups.

## Supporting Information Available

Complete Gaussian09 reference; distance matrices and energy rankings for the B_5_H_5_Fe(*CO*)_*x*_ (*x* = 4, 3, 2) structures; distance matrices and energy rankings for the B_6_H_6_Fe(*CO*)_*x*_ (*x* = 4, 3, 2) structures; distance matrices and energy rankings for the B_7_H_7_Fe(*CO*)_*x*_ (*x* = 4, 3, 2) structures; distance matrices and energy rankings for the B_8_H_8_Fe(*CO*)_*x*_ (*x* = 4, 3, 2) structures; distance matrices and energy rankings for the B_9_H_9_Fe(*CO*)_*x*_ (*x* = 4, 3, 2) structures; distance matrices and energy rankings for the B_10_H_10_Fe(*CO*)_*x*_ (*x* = 4, 3, 2) structures; distance matrices and energy rankings for the B_11_H_11_Fe(*CO*)_*x*_ (*x* = 4, 3, 2) structures; distance matrices and energy rankings for the B_12_H_12_Fe(*CO*)_*x*_ (*x* = 4, 3, 2) structures; distance matrices and energy rankings for the [B_5_H_5_Fe(CO)_3_]^2–^ structures; distance matrices and energy rankings for the [B_6_H_6_Fe(CO)_3_]^2–^ structures; distance matrices and energy rankings for the [B_7_H_7_Fe(CO)_3_]^2–^ structures; distance matrices and energy rankings for the [B_8_H_8_Fe(CO)_3_]^2–^ structures; distance matrices and energy rankings for the [B_9_H_9_Fe(CO)_3_]^2–^ structures; distance matrices and energy rankings for the [B_10_H_10_Fe(CO)_3_]^2–^ structures; distance matrices and energy rankings for the [B_11_H_11_Fe(CO)_3_]^2–^ structures; distance matrices and energy rankings for the [B_12_H_12_Fe(CO)_3_]^2–^ structures; and orbital energies and HOMO–LUMO gaps for the lowest energy structures (PDF)
Separate file of concatenated (XYZ) coordinates of the optimized structures

## Supplementary Material

jp3c02944_si_001.pdf

jp3c02944_si_002.xyz
