# Supplementary material for: Polyhedral Ferraboranes with Iron Carbonyl Vertices: Carbonyl Migration Processes in the Iron Tetracarbonyl Derivatives
Source: J Phys Chem A. 2023 Jul 11;127(28):5887–98. doi: 10.1021/acs.jpca.3c02944 (PMC10364080; doi:10.1021/acs.jpca.3c02944)
Supplement: Supplementary file 1 — jp3c02944_si_001.pdf [file jp3c02944_si_001.pdf]

## Carbonyl Migration Reactions in Borane Iron Carbonyl Chemistry

Amr A. A. Attia,[1] Alexandru Lupan,[*1] and R. Bruce King[*2]

[1]*Faculty of Chemistry and Chemical Engineering, Babeş-Bolyai University, Cluj-Napoca, Romania*
[2]*Department of Chemistry, University of Georgia, Athens, Georgia, 30602, USA*

## Supporting Information

Complete Gaussian09 Reference.
Tables S1A-S1F: Distance matrices and energy rankings for the $B_5H_5Fe(CO)_x$ ($x$ = 4, 3, 2) structures.
Tables S2A-S2F: Distance matrices and energy rankings for the $B_6H_6Fe(CO)_x$ ($x$ = 4, 3, 2) structures.
Tables S3A-S3F: Distance matrices and energy rankings for the $B_7H_7Fe(CO)_x$ ($x$ = 4, 3, 2) structures.
Tables S4A-S4F: Distance matrices and energy rankings for the $B_8H_8Fe(CO)_x$ ($x$ = 4, 3, 2) structures.
Tables S5A-S5F: Distance matrices and energy rankings for the $B_9H_9Fe(CO)_x$ ($x$ = 4, 3, 2) structures.
Tables S6A-S6F: Distance matrices and energy rankings for the $B_{10}H_{10}Fe(CO)_x$ ($x$ = 4, 3, 2) structures.
Tables S7A-S7F: Distance matrices and energy rankings for the $B_{11}H_{11}Fe(CO)_x$ ($x$ = 4, 3, 2) structures.
Tables S8A-S8F: Distance matrices and energy rankings for the $B_{12}H_{12}Fe(CO)_x$ ($x$ = 4, 3, 2) structures.
Tables S9A-S9B: Distance matrices and energy rankings for the $[B_5H_5Fe(CO)_3]^{2-}$ structures.
Tables S10A-S10B: Distance matrices and energy rankings for the $[B_6H_6Fe(CO)_3]^{2-}$ structures.
Tables S11A-S11B: Distance matrices and energy rankings for the $[B_7H_7Fe(CO)_3]^{2-}$ structures.
Tables S12A-S12B: Distance matrices and energy rankings for the $[B_8H_8Fe(CO)_3]^{2-}$ structures.
Tables S13A-S13B: Distance matrices and energy rankings for the $[B_9H_9Fe(CO)_3]^{2-}$ structures.
Tables S14A-S14B: Distance matrices and energy rankings for the $[B_{10}H_{10}Fe(CO)_3]^{2-}$ structures.
Tables S15A-S15B: Distance matrices and energy rankings for the $[B_{11}H_{11}Fe(CO)_3]^{2-}$ structures.
Tables S16A-S16B: Distance matrices and energy rankings for the $[B_{12}H_{12}Fe(CO)_3]^{2-}$ structures.
Table S17: Orbital energies and HOMO-LUMO gaps for the lowest energy structures.

Complete Gaussian09 Reference.

Gaussian 09, Revision E.01, M. J. Frisch, G. W. Trucks, H. B. Schlegel, G. E. Scuseria, M. A. Robb, J. R. Cheeseman, G. Scalmani, V. Barone, B. Mennucci, G. A. Petersson, H. Nakatsuji, M. Caricato, X. Li, H. P. Hratchian, A. F. Izmaylov, J. Bloino, G. Zheng, J. L. Sonnenberg, M. Hada, M. Ehara, K. Toyota, R. Fukuda, J. Hasegawa, M. Ishida, T. Nakajima, Y. Honda, O. Kitao, H. Nakai, T. Vreven, J. A. Montgomery, Jr., J. E. Peralta, F. Ogliaro, M. Bearpark, J. J. Heyd, E. Brothers, K. N. Kudin, V. N. Staroverov, R. Kobayashi, J. Normand, K. Raghavachari, A. Rendell, J. C. Burant, S. S. Iyengar, J. Tomasi, M. Cossi, N. Rega, J. M. Millam, M. Klene, J. E. Knox, J. B. Cross, V. Bakken, C. Adamo, J. Jaramillo, R. Gomperts, R. E. Stratmann, O. Yazyev, A. J. Austin, R. Cammi, C. Pomelli, J. W. Ochterski, R. L. Martin, K. Morokuma, V. G. Zakrzewski, G. A. Voth, P. Salvador, J. J. Dannenberg, S. Dapprich, A. D. Daniels, O. Farkas, J. B. Foresman, J. V. Ortiz, J. Cioslowski, and D. J. Fox, Gaussian, Inc., Wallingford CT, 2009.

Table S1A: Distance table for the lowest-lying $B_5H_5Fe(CO)_2$ optimized structures obtained at the PBE0/def2-TZVP level of theory. Included are the zero-point corrected absolute energy in (a.u.) at the DLPNO-CCSD(T)/def2-QZVP level of theory with zero-point energy obtained from the PBE0/def2-TZVP computations, relative energies in (kcal/mol) and symmetry. For clarity, only the atoms forming the cluster framework are shown.

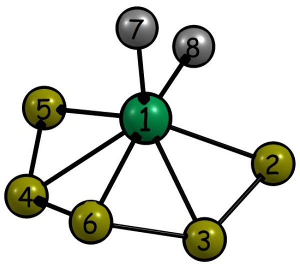


1. -1616.03171943 0.0 $C_s$

| | | 1 | 2 | 3 | 4 | 5 |
|---|---|---|---|---|---|---|
| 1 | Fe | 0.000000 | | | | |
| 2 | B | 1.894147 | 0.000000 | | | |
| 3 | B | 1.993401 | 1.650724 | 0.000000 | | |
| 4 | B | 1.993614 | 3.473684 | 2.693500 | 0.000000 | |
| 5 | B | 1.893672 | 3.468299 | 3.473815 | 1.650836 | 0.000000 |
| 6 | B | 2.035645 | 2.999930 | 1.688963 | 1.689390 | 2.999814 |

| | | 6 |
|---|---|---|
| 6 | B | 0.000000 |

Fe1-C7: 1.761094 Fe1-C8: 1.733844

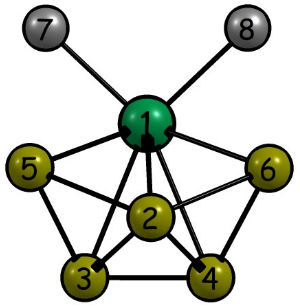


2. -1616.02755526 +2.6 $C_s$

| | | 1 | 2 | 3 | 4 | 5 |
|---|---|---|---|---|---|---|
| 1 | Fe | 0.000000 | | | | |
| 2 | B | 2.211463 | 0.000000 | | | |
| 3 | B | 2.176785 | 1.725152 | 0.000000 | | |
| 4 | B | 2.175974 | 1.724743 | 1.595071 | 0.000000 | |
| 5 | B | 1.979656 | 1.786950 | 1.677556 | 2.763156 | 0.000000 |
| 6 | B | 1.980796 | 1.787946 | 2.764287 | 1.676585 | 3.025315 |

| | | 6 |
|---|---|---|
| 6 | B | 0.000000 |

Fe1-C7: 1.765541 Fe1-C8: 1.765274

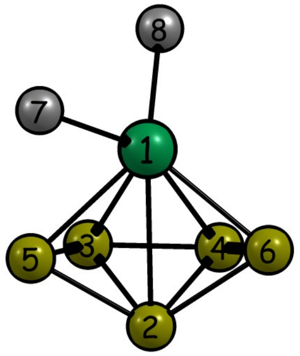


3. -1616.01814442 +8.5 $C_1$

| | | 1 | 2 | 3 | 4 | 5 |
|---|---|---|---|---|---|---|
| 1 | Fe | 0.000000 | | | | |
| 2 | B | 2.234645 | 0.000000 | | | |
| 3 | B | 2.169802 | 1.729579 | 0.000000 | | |
| 4 | B | 2.236050 | 1.732208 | 1.627234 | 0.000000 | |
| 5 | B | 1.970639 | 1.738251 | 1.695380 | 2.783747 | 0.000000 |
| 6 | B | 1.945741 | 1.763679 | 2.712289 | 1.646304 | 2.930099 |

| | | 6 |
|---|---|---|
| 6 | B | 0.000000 |

Fe1-C7: 1.778900 Fe1-C8: 1.784144

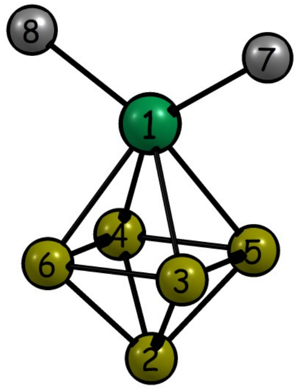


4. -1616.01155228 +12.7 $C_{2\mathrm{v}}$

| | | 1 | 2 | 3 | 4 | 5 |
|---|---|---|---|---|---|---|
| 1 | Fe | 0.000000 | | | | |
| 2 | B | 2.827348 | 0.000000 | | | |
| 3 | B | 2.033999 | 1.746767 | 0.000000 | | |
| 4 | B | 2.034550 | 1.746312 | 2.497078 | 0.000000 | |
| 5 | B | 2.035040 | 1.746205 | 1.729021 | 1.801768 | 0.000000 |
| 6 | B | 2.033246 | 1.747198 | 1.801538 | 1.729088 | 2.497152 |

| | | 6 |
|---|---|---|
| 6 | B | 0.000000 |

Fe1-C7: 1.848304  Fe1-C8: 1.848315

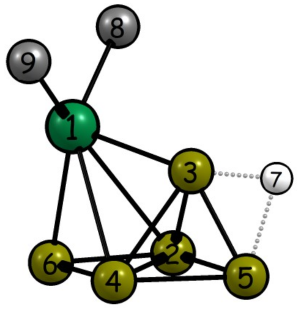


5. -1616.00801501 +14.9 $C_{\mathrm{s}}$

| | | 1 | 2 | 3 | 4 | 5 |
|---|---|---|---|---|---|---|
| 1 | Fe | 0.000000 | | | | |
| 2 | B | 2.303595 | 0.000000 | | | |
| 3 | B | 1.820354 | 1.803309 | 0.000000 | | |
| 4 | B | 2.308314 | 1.846907 | 1.803619 | 0.000000 | |
| 5 | B | 3.149582 | 1.775635 | 1.586881 | 1.775742 | 0.000000 |
| 6 | B | 1.931019 | 1.666479 | 2.545976 | 1.665634 | 2.902697 |
| 7 | H | 3.053119 | 2.580330 | 1.258333 | 2.579365 | 1.450647 |

| | | 6 | 7 |
|---|---|---|---|
| 6 | B | 0.000000 | |
| 7 | H | 3.656787 | 0.000000 |

Fe1-C8: 1.772403  Fe1-C9: 1.773302

Table S1B: Distance table for the lowest-lying $B_5H_5Fe(CO)_3$ optimized structures obtained at the PBE0/def2-TZVP level of theory. Included are the zero-point corrected absolute energy in (a.u.) at the DLPNO-CCSD(T)/def2-QZVP level of theory with zero-point energy obtained from the PBE0/def2-TZVP computations, relative energies in (kcal/mol) and symmetry. For clarity, only the atoms forming the cluster framework are shown.

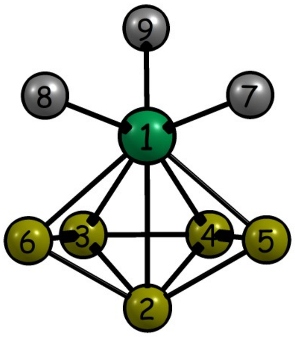


1. -1729.25327442 0.0 $C_s$

| | | 1 | 2 | 3 | 4 | 5 |
|---|---|---|---|---|---|---|
| 1 | Fe | 0.000000 | | | | |
| 2 | B | 2.215975 | 0.000000 | | | |
| 3 | B | 2.203753 | 1.708713 | 0.000000 | | |
| 4 | B | 2.203350 | 1.709054 | 1.609431 | 0.000000 | |
| 5 | B | 1.995370 | 1.763139 | 2.776408 | 1.698490 | 0.000000 |
| 6 | B | 1.995083 | 1.763008 | 1.698831 | 2.776436 | 2.996755 |
| | | 6 | | | | |
| 6 | B | 0.000000 | | | | |

Fe1-C7: 1.761865 Fe1-C8: 1.761857 Fe1-C9: 1.777320

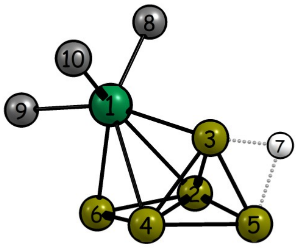


2. -1729.23744235 +9.9 $C_s$

| | | 1 | 2 | 3 | 4 | 5 |
|---|---|---|---|---|---|---|
| 1 | Fe | 0.000000 | | | | |
| 2 | B | 2.209692 | 0.000000 | | | |
| 3 | B | 1.885061 | 1.772627 | 0.000000 | | |
| 4 | B | 2.209285 | 1.815223 | 1.771771 | 0.000000 | |
| 5 | B | 3.156881 | 1.778620 | 1.589186 | 1.778973 | 0.000000 |
| 6 | B | 1.980000 | 1.706644 | 2.691247 | 1.707223 | 2.956697 |
| 7 | H | 3.122591 | 2.576542 | 1.253891 | 2.577938 | 1.481963 |
| | | 6 | 7 | | | |
| 6 | B | 0.000000 | | | | |
| 7 | H | 3.783795 | 0.000000 | | | |

Fe1-C8: 1.764641 Fe1-C9: 1.767204 Fe1-C10: 1.764713

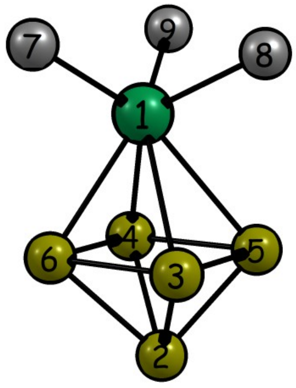


3. -1729.22388943 +18.4 $C_s$

| | | 1 | 2 | 3 | 4 | 5 |
|---|---|---|---|---|---|---|
| 1 | Fe | 0.000000 | | | | |
| 2 | B | 2.878976 | 0.000000 | | | |
| 3 | B | 2.166444 | 1.702749 | 0.000000 | | |
| 4 | B | 2.005253 | 1.799120 | 2.521710 | 0.000000 | |
| 5 | B | 2.165700 | 1.702875 | 1.872309 | 1.744818 | 0.000000 |
| 6 | B | 2.005273 | 1.799113 | 1.744924 | 1.769675 | 2.521286 |
| | | 6 | | | | |
| 6 | B | 0.000000 | | | | |

Fe1-C7: 1.809779 Fe1-C8: 1.791571 Fe1-C9: 1.809676

Table S1C: Distance table for the lowest-lying $B_5H_5Fe(CO)_4$ optimized structures obtained at the PBE0/def2-TZVP level of theory. Included are the zero-point corrected absolute energy in (a.u.) at the DLPNO-CCSD(T)/def2-QZVP level of theory with zero-point energy obtained from the PBE0/def2-TZVP computations, relative energies in (kcal/mol) and symmetry. For clarity, only the atoms forming the cluster framework are shown.

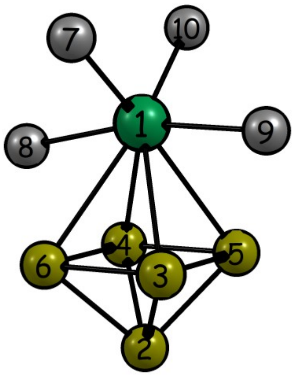


1. -1842.43508661 0.0 $C_{4v}$

| | | 1 | 2 | 3 | 4 | 5 |
|---|---|---|---|---|---|---|
| 1 | Fe | 0.000000 | | | | |
| 2 | B | 2.897445 | 0.000000 | | | |
| 3 | B | 2.139766 | 1.711053 | 0.000000 | | |
| 4 | B | 2.175374 | 1.731606 | 2.548889 | 0.000000 | |
| 5 | B | 2.139806 | 1.710688 | 1.876877 | 1.725961 | 0.000000 |
| 6 | B | 2.175058 | 1.731464 | 1.725609 | 1.873858 | 2.548299 |

| | | 6 |
|---|---|---|
| 6 | B | 0.000000 |

Fe1-C7: 1.786121 Fe1-C8: 1.828412
Fe1-C9: 1.780731 Fe1-C10: 1.786074

Table S1D: Energy ranking for the $B_5H_5Fe(CO)_2$ structures at the PBE0/def2-TZVP level of theory.

| No. | Initial structure | Final Energy (a.u.) | Relative Energy (kcal/mol) |
|---|---|---|---|
| 1 | 06v-04-PentPyr—fe1—2co-pbe0 | -1616.8456480 | 0.00 |
| 2 | 06v-03-CapTetrPyr—fe1—2co-pbe0 | -1616.8433020 | 1.47 |
| 3 | 06v-03-CapTetrPyr—fe2—2co-pbe0 | -1616.8432980 | 1.47 |
| 4 | 06v-02-BicappTd—fe2—2co-pbe0 | -1616.8432950 | 1.48 |
| 5 | 06v-01-Oh—fe1—2co-pbe0 | -1616.8377240 | 4.97 |
| 6 | 06v-02-BicappTd—fe1—2co-pbe0 | -1616.8246040 | 13.21 |

Table S1E: Energy ranking for the $B_5H_5Fe(CO)_2$ structures at the PBE0/def2-TZVP level of theory.

| No. | Initial structure | Final Energy (a.u.) | Relative Energy (kcal/mol) |
|---|---|---|---|
| 1 | 06v-04-PentPyr—fe1—2co-pbe0 | -1616.8456480 | 0.00 |
| 2 | 06v-03-CapTetrPyr—fe1—2co-pbe0 | -1616.8433020 | 1.47 |
| 3 | 06v-03-CapTetrPyr—fe2—2co-pbe0 | -1616.8432980 | 1.47 |
| 4 | 06v-02-BicappTd—fe2—2co-pbe0 | -1616.8432950 | 1.48 |
| 5 | 06v-01-Oh—fe1—2co-pbe0 | -1616.8377240 | 4.97 |
| 6 | 06v-02-BicappTd—fe1—2co-pbe0 | -1616.8246040 | 13.21 |

Table S1F: Energy ranking for the $B_5H_5Fe(CO)_2$ structures at the PBE0/def2-TZVP level of theory.

| No. | Initial structure | Final Energy (a.u.) | Relative Energy (kcal/mol) |
|---|---|---|---|
| 1 | 06v-01-Oh—fe1—4co-r-54-pbe0 | -1843.3157550 | 0.00 |
| 2 | 06v-01-Oh—fe1—4co-i-54-pbe0 | -1843.3157200 | 0.02 |
| 3 | 06v-03-CapTetrPyr—fe2—4co-pbe0 | -1843.2926620 | 14.49 |
| 4 | 06v-02-BicappTd—fe1—4co-pbe0 | -1843.2926470 | 14.50 |
| 5 | 06v-04-PentPyr—fe1—4co-pbe0 | -1843.2901740 | 16.05 |
| 6 | 06v-03-CapTetrPyr—fe1—4co-pbe0 | -1843.2659040 | 31.28 |
| 7 | 06v-02-BicappTd—fe2—4co-pbe0 | -1843.2659010 | 31.28 |

Table S2A: Distance table for the lowest-lying $B_6H_6Fe(CO)_2$ optimized structures obtained at the PBE0/def2-TZVP level of theory. Included are the zero-point corrected absolute energy in (a.u.) at the DLPNO-CCSD(T)/def2-QZVP level of theory with zero-point energy obtained from the PBE0/def2-TZVP computations, relative energies in (kcal/mol) and symmetry. For clarity, only the atoms forming the cluster framework are shown.

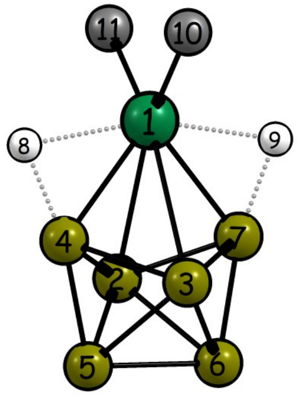


1. -1641.44794334 0.0 $C_{2v}$

| | | 1 | 2 | 3 | 4 | 5 |
|---|---|---|---|---|---|---|
| 1 | Fe | 0.000000 | | | | |
| 2 | B | 2.348466 | 0.000000 | | | |
| 3 | B | 2.348194 | 2.484221 | 0.000000 | | |
| 4 | B | 1.874354 | 1.780251 | 1.779256 | 0.000000 | |
| 5 | B | 3.161410 | 1.842421 | 1.842375 | 1.596217 | 0.000000 |
| 6 | B | 3.161651 | 1.842079 | 1.841878 | 2.566612 | 1.737714 |
| 7 | B | 1.874401 | 1.778226 | 1.779403 | 2.324041 | 2.566217 |
| 8 | H | 1.692824 | 2.703184 | 2.703752 | 1.304729 | 2.884194 |
| 9 | H | 1.692459 | 2.702602 | 2.702504 | 3.077196 | 3.758388 |

| | | 6 | 7 | 8 | 9 |
|---|---|---|---|---|---|
| 6 | B | 0.000000 | | | |
| 7 | B | 1.596252 | 0.000000 | | |
| 8 | H | 3.758765 | 3.077424 | 0.000000 | |
| 9 | H | 2.884402 | 1.304887 | 3.342160 | 0.000000 |

Fe1-C10: 1.794862 Fe1-C11: 1.794847

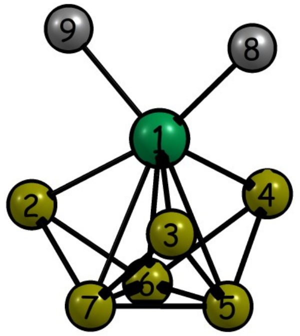


2. -1641.44005065 +5.0 $C_s$

| | | 1 | 2 | 3 | 4 | 5 |
|---|---|---|---|---|---|---|
| 1 | Fe | 0.000000 | | | | |
| 2 | B | 1.941169 | 0.000000 | | | |
| 3 | B | 1.869301 | 2.884263 | 0.000000 | | |
| 4 | B | 1.862276 | 2.886965 | 2.892724 | 0.000000 | |
| 5 | B | 2.194543 | 2.812276 | 1.835441 | 1.842493 | 0.000000 |
| 6 | B | 2.204136 | 1.767980 | 2.871251 | 1.827121 | 1.676095 |
| 7 | B | 2.205094 | 1.765386 | 1.818771 | 2.878912 | 1.679675 |

| | | 6 | 7 |
|---|---|---|---|
| 6 | B | 0.000000 | |
| 7 | B | 1.708779 | 0.000000 |

Fe1-C8: 1.739508 Fe1-C9: 1.758274

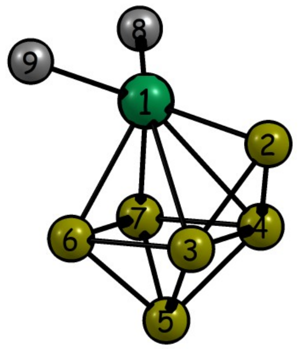


3. -1641.43475724 +8.3 $C_s$

| | | 1 | 2 | 3 | 4 | 5 |
|---|---|---|---|---|---|---|
| 1 | Fe | 0.000000 | | | | |
| 2 | B | 1.874951 | 0.000000 | | | |
| 3 | B | 2.263330 | 1.700220 | 0.000000 | | |
| 4 | B | 2.262836 | 1.700460 | 1.806470 | 0.000000 | |
| 5 | B | 2.884321 | 2.902952 | 1.730485 | 1.730948 | 0.000000 |
| 6 | B | 2.035973 | 2.917260 | 1.808875 | 2.542998 | 1.706932 |
| 7 | B | 2.035795 | 2.916995 | 2.542738 | 1.807939 | 1.706875 |

| | | 6 | 7 |
|---|---|---|---|
| 6 | B | 0.000000 | |
| 7 | B | 1.769108 | 0.000000 |

Fe1-C8: 1.795921 Fe1-C9: 1.796035

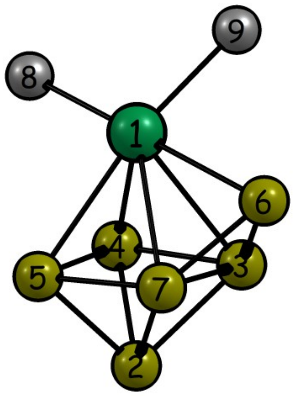


4. -1641.43334499 +9.2 $C_1$

| | | 1 | 2 | 3 | 4 | 5 |
|---|---|---|---|---|---|---|
| 1 | Fe | 0.000000 | | | | |
| 2 | B | 2.886868 | 0.000000 | | | |
| 3 | B | 2.135351 | 1.771281 | 0.000000 | | |
| 4 | B | 2.031550 | 1.727921 | 1.759075 | 0.000000 | |
| 5 | B | 2.114731 | 1.722685 | 2.555050 | 1.826413 | 0.000000 |
| 6 | B | 2.022391 | 2.987074 | 1.822873 | 3.113002 | 3.045654 |
| 7 | B | 2.080391 | 1.732453 | 1.771064 | 2.433337 | 1.701603 |

| | | 6 | 7 |
|---|---|---|---|
| 6 | B | 0.000000 | |
| 7 | B | 1.635488 | 0.000000 |

Fe1-C8: 1.775129 Fe1-C9: 1.773430

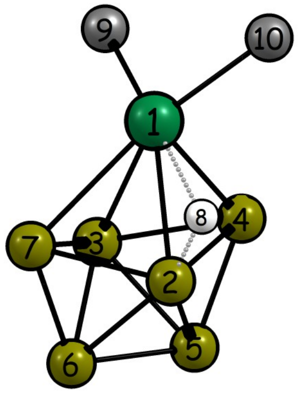


5. -1641.40111452 +29.4 $C_1$

| | | 1 | 2 | 3 | 4 | 5 |
|---|---|---|---|---|---|---|
| 1 | Fe | 0.000000 | | | | |
| 2 | B | 1.979425 | 0.000000 | | | |
| 3 | B | 2.301501 | 2.002649 | 0.000000 | | |
| 4 | B | 2.051992 | 1.803546 | 1.843340 | 0.000000 | |
| 5 | B | 3.146708 | 1.709684 | 1.797727 | 1.701750 | 0.000000 |
| 6 | B | 3.209352 | 1.742623 | 1.831163 | 2.836142 | 1.707179 |
| 7 | B | 2.158675 | 1.782347 | 1.750326 | 2.885795 | 2.709800 |
| 8 | H | 1.707359 | 1.318899 | 3.010524 | 2.415317 | 2.940039 |

| | | 6 | 7 | 8 |
|---|---|---|---|---|
| 6 | B | 0.000000 | | |
| 7 | B | 1.625145 | 0.000000 | |
| 8 | H | 2.950444 | 2.419639 | 0.000000 |

Fe1-C9: 1.765634 Fe1-C10: 1.773718

Table S2B: Distance table for the lowest-lying $B_6H_6Fe(CO)_3$ optimized structures obtained at the PBE0/def2-TZVP level of theory. Included are the zero-point corrected absolute energy in (a.u.) at the DLPNO-CCSD(T)/def2-QZVP level of theory with zero-point energy obtained from the PBE0/def2-TZVP computations, relative energies in (kcal/mol) and symmetry. For clarity, only the atoms forming the cluster framework are shown.

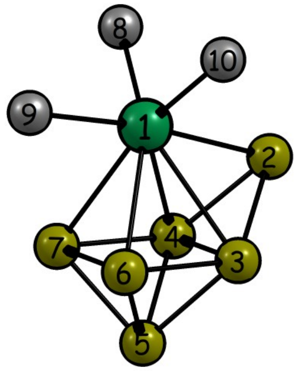


1. -1754.66084374 0.0 $C_s$

| | | 1 | 2 | 3 | 4 | 5 |
|---|---|---|---|---|---|---|
| 1 | Fe | 0.000000 | | | | |
| 2 | B | 1.975907 | 0.000000 | | | |
| 3 | B | 2.148523 | 1.742719 | 0.000000 | | |
| 4 | B | 2.147423 | 1.741421 | 1.769764 | 0.000000 | |
| 5 | B | 2.916470 | 3.012678 | 1.751983 | 1.752760 | 0.000000 |
| 6 | B | 2.108518 | 3.068444 | 1.729096 | 2.506797 | 1.717552 |
| 7 | B | 2.108438 | 3.068297 | 2.506259 | 1.729389 | 1.716736 |

| | | 6 | 7 |
|---|---|---|---|
| 6 | B | 0.000000 | |
| 7 | B | 1.860362 | 0.000000 |

Fe1-C8: 1.774309 Fe1-C9: 1.768711 Fe1-C10: 1.774292

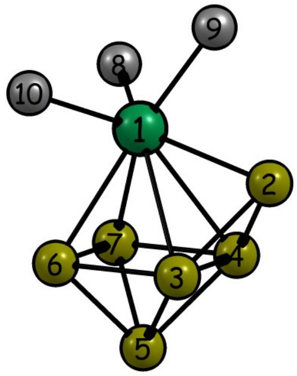


2. -1754.65814875 +1.7 $C_s$

| | | 1 | 2 | 3 | 4 | 5 |
|---|---|---|---|---|---|---|
| 1 | Fe | 0.000000 | | | | |
| 2 | B | 1.974507 | 0.000000 | | | |
| 3 | B | 2.196797 | 1.724035 | 0.000000 | | |
| 4 | B | 2.197721 | 1.725627 | 1.794896 | 0.000000 | |
| 5 | B | 2.917497 | 2.968755 | 1.744262 | 1.744586 | 0.000000 |
| 6 | B | 2.090885 | 3.045150 | 1.781703 | 2.520759 | 1.714843 |
| 7 | B | 2.090367 | 3.043796 | 2.519815 | 1.779354 | 1.715374 |

| | | 6 | 7 |
|---|---|---|---|
| 6 | B | 0.000000 | |
| 7 | B | 1.772563 | 0.000000 |

Fe1-C8: 1.771256 Fe1-C9: 1.774054 Fe1-C10: 1.771053

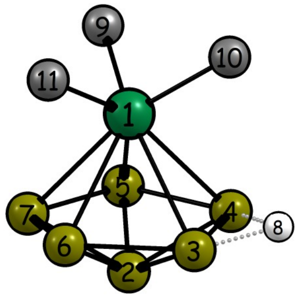


3. -1754.65460845 +3.9 $C_s$

| | | 1 | 2 | 3 | 4 | 5 |
|---|---|---|---|---|---|---|
| 1 | Fe | 0.000000 | | | | |
| 2 | B | 2.335657 | 0.000000 | | | |
| 3 | B | 2.213978 | 1.693593 | 0.000000 | | |
| 4 | B | 2.212442 | 1.694120 | 1.802151 | 0.000000 | |
| 5 | B | 2.139778 | 1.670212 | 2.874428 | 1.810510 | 0.000000 |
| 6 | B | 2.138450 | 1.670268 | 1.811755 | 2.874031 | 2.763919 |
| 7 | B | 2.212866 | 1.660180 | 2.878650 | 2.877886 | 1.700755 |
| 8 | H | 2.632310 | 2.365746 | 1.326332 | 1.327196 | 3.041553 |

| | | 6 | 7 | 8 |
|---|---|---|---|---|
| 6 | B | 0.000000 | | |
| 7 | B | 1.700408 | 0.000000 | |
| 8 | H | 3.041494 | 3.699877 | 0.000000 |

Fe1-C9: 1.758844 Fe1-C10: 1.752782 Fe1-C11: 1.758943

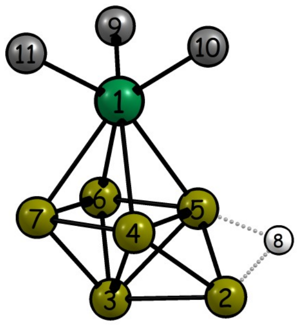


4. -1754.63853310 +14.0 $C_1$

| | | 1 | 2 | 3 | 4 | 5 |
|---|---|---|---|---|---|---|
| 1 | Fe | 0.000000 | | | | |
| 2 | B | 3.265504 | 0.000000 | | | |
| 3 | B | 2.944692 | 1.886571 | 0.000000 | | |
| 4 | B | 2.179830 | 1.767248 | 1.850317 | 0.000000 | |
| 5 | B | 2.010299 | 1.587743 | 1.844506 | 1.819117 | 0.000000 |
| 6 | B | 2.042537 | 2.945722 | 1.765629 | 2.569181 | 1.729631 |
| 7 | B | 2.082626 | 2.996973 | 1.706635 | 1.719947 | 2.423042 |
| 8 | H | 3.142788 | 1.439559 | 2.614556 | 2.635807 | 1.272917 |

| | | 6 | 7 | 8 |
|---|---|---|---|---|
| 6 | B | 0.000000 | | |
| 7 | B | 1.793417 | 0.000000 | |
| 8 | H | 2.845741 | 3.604527 | 0.000000 |

Fe1-C9: 1.763750 Fe1-C10: 1.781814 Fe1-C11: 1.764383

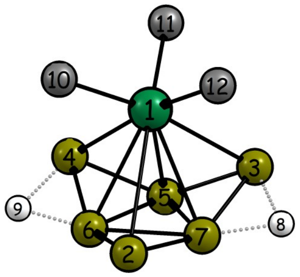


5. -1754.61202234 +30.6 $C_s$

| | | 1 | 2 | 3 | 4 | 5 |
|---|---|---|---|---|---|---|
| 1 | Fe | 0.000000 | | | | |
| 2 | B | 2.576179 | 0.000000 | | | |
| 3 | B | 2.134485 | 3.121006 | 0.000000 | | |
| 4 | B | 2.134411 | 3.120782 | 3.126399 | 0.000000 | |
| 5 | B | 2.115804 | 2.791238 | 1.708681 | 1.710480 | 0.000000 |
| 6 | B | 2.224425 | 1.598999 | 2.938099 | 1.652852 | 1.778096 |
| 7 | B | 2.225067 | 1.598220 | 1.653962 | 2.939678 | 1.779154 |
| 8 | H | 2.840050 | 2.707396 | 1.366489 | 3.960007 | 2.528932 |
| 9 | H | 2.839352 | 2.708551 | 3.959305 | 1.364280 | 2.529541 |

| | | 6 | 7 | 8 | 9 |
|---|---|---|---|---|---|
| 6 | B | 0.000000 | | | |
| 7 | B | 1.888215 | 0.000000 | | |
| 8 | H | 3.170099 | 1.307249 | 0.000000 | |
| 9 | H | 1.308447 | 3.171482 | 4.418695 | 0.000000 |

Fe1-C10: 1.768663 Fe1-C11: 1.756536 Fe1-C12: 1.767883

Table S2C: Distance table for the lowest-lying $B_6H_6Fe(CO)_4$ optimized structures obtained at the PBE0/def2-TZVP level of theory. Included are the zero-point corrected absolute energy in (a.u.) at the DLPNO-CCSD(T)/def2-QZVP level of theory with zero-point energy obtained from the PBE0/def2-TZVP computations, relative energies in (kcal/mol) and symmetry. For clarity, only the atoms forming the cluster framework are shown.

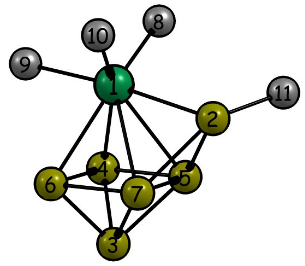


1. -1867.80082241 0.0 $C_1$

| | | 1 | 2 | 3 | 4 | 5 |
|---|---|---|---|---|---|---|
| 1 | Fe | 0.000000 | | | | |
| 2 | B | 2.092970 | 0.000000 | | | |
| 3 | B | 2.898385 | 3.202831 | 0.000000 | | |
| 4 | B | 2.109831 | 3.247588 | 1.687393 | 0.000000 | |
| 5 | B | 2.167951 | 1.909002 | 1.769261 | 1.747962 | 0.000000 |
| 6 | B | 2.104146 | 3.200306 | 1.690030 | 1.903573 | 2.520644 |
| 7 | B | 2.196512 | 1.871261 | 1.776427 | 2.549537 | 1.775868 |
| | | 6 | 7 | | | |
| 6 | B | 0.000000 | | | | |
| 7 | B | 1.742978 | 0.000000 | | | |

Fe1-C8: 1.775703 Fe1-C9: 1.761009
Fe1-C10: 1.781383 Fe1-C11: 3.040564

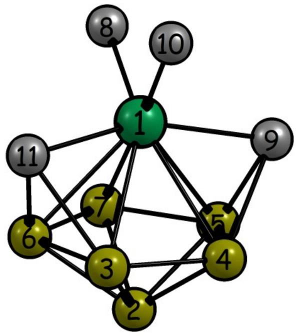


2. -1867.78883826 +7.5 $C_s$

| | | 1 | 2 | 3 | 4 | 5 |
|---|---|---|---|---|---|---|
| 1 | Fe | 0.000000 | | | | |
| 2 | B | 2.699163 | 0.000000 | | | |
| 3 | B | 2.230436 | 1.785544 | 0.000000 | | |
| 4 | B | 2.229815 | 1.786370 | 1.702459 | 0.000000 | |
| 5 | B | 2.252242 | 1.789675 | 2.812127 | 1.775671 | 0.000000 |
| 6 | B | 2.250066 | 1.790076 | 1.777092 | 2.812311 | 2.791874 |
| 7 | B | 2.164161 | 1.811340 | 2.776325 | 2.775390 | 1.690799 |
| | | 6 | 7 | | | |
| 6 | B | 0.000000 | | | | |
| 7 | B | 1.691078 | 0.000000 | | | |

Fe1-C8: 1.780115 Fe1-C9: 1.852178
Fe1-C10: 1.784200 Fe1-C11: 1.853771

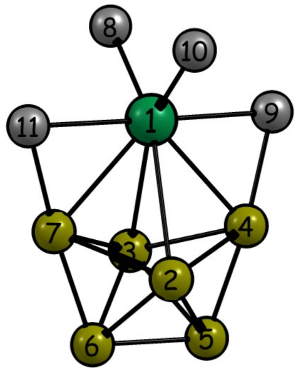


3. -1867.78409046 +10.5 $C_{2v}$

| | | 1 | 2 | 3 | 4 | 5 |
|---|---|---|---|---|---|---|
| 1 | Fe | 0.000000 | | | | |
| 2 | B | 2.339080 | 0.000000 | | | |
| 3 | B | 2.338896 | 2.351008 | 0.000000 | | |
| 4 | B | 2.038857 | 1.843111 | 1.890216 | 0.000000 | |
| 5 | B | 3.209767 | 1.846999 | 1.752079 | 1.669077 | 0.000000 |
| 6 | B | 3.209829 | 1.751789 | 1.847118 | 2.702672 | 1.672151 |
| 7 | B | 2.039237 | 1.890459 | 1.842934 | 2.720410 | 2.702829 |
| | | 6 | 7 | | | |
| 6 | B | 0.000000 | | | | |
| 7 | B | 1.669111 | 0.000000 | | | |

Fe1-C8: 1.774283 Fe1-C9: 1.843451
Fe1-C10: 1.774273 Fe1-C11: 1.843371

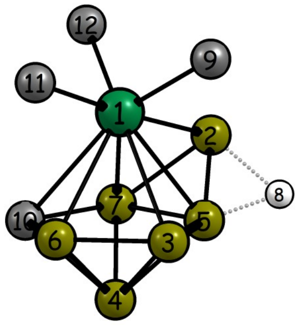


4. -1867.76909947 +19.9 $C_1$

| | | 1 | 2 | 3 | 4 | 5 |
|---|---|---|---|---|---|---|
| 1 | Fe | 0.000000 | | | | |
| 2 | B | 2.133636 | 0.000000 | | | |
| 3 | B | 2.216290 | 3.020970 | 0.000000 | | |
| 4 | B | 2.827569 | 3.046177 | 1.831764 | 0.000000 | |
| 5 | B | 2.133457 | 1.605331 | 1.648348 | 1.774136 | 0.000000 |
| 6 | B | 2.226631 | 3.652764 | 1.657668 | 1.801394 | 2.620597 |
| 7 | B | 2.184428 | 1.768404 | 2.760713 | 1.851221 | 1.708103 |
| 8 | H | 2.885219 | 1.453967 | 2.667242 | 2.965698 | 1.274449 |

| | | 6 | 7 | 8 |
|---|---|---|---|---|
| 6 | B | 0.000000 | | |
| 7 | B | 2.676580 | 0.000000 | |
| 8 | H | 3.858407 | 2.532699 | 0.000000 |

Fe1-C9: 1.776241 Fe1-C10: 2.265709

Fe1-C11: 1.777139 Fe1-C12: 1.781156

Table S2D: Energy ranking for the $B_5H_5Fe(CO)_2$ structures at the PBE0/def2-TZVP level of theory.

| No. | Initial structure | Final Energy (a.u.) | Relative Energy (kcal/mol) |
|---|---|---|---|
| 1 | 07v-01-PentBipyr—fe2—2co-pbe0 | -1642.2914470 | 0.00 |
| 2 | 07v-04-CapOh—fe2—2co-pbe0 | -1642.2864180 | 3.16 |
| 3 | 07v-04-CapOh—fe1—2co-pbe0 | -1642.2864030 | 3.17 |
| 4 | 07v-01-PentBipyr—fe1—2co-pbe0 | -1642.2847820 | 4.18 |
| 5 | 07v-03-TricapTd—fe2—2co-r-22-pbe0 | -1642.2717070 | 12.39 |
| 6 | 07v-03-TricapTd—fe2—2co-i-22-pbe0 | -1642.2716820 | 12.40 |
| 7 | 07v-02-HexPyr—fe1—2co-pbe0 | -1642.2664930 | 15.66 |
| 8 | 07v-04-CapOh—fe4—2co-pbe0 | -1642.2662080 | 15.84 |
| 9 | 07v-04-CapOh—fe3—2co-pbe0 | -1642.2607590 | 19.26 |
| 10 | 07v-03-TricapTd—fe1—2co-pbe0 | -1642.2443270 | 29.57 |
| 11 | 07v-03-TricapTd—fe3—2co-pbe0 | -1642.2443270 | 29.57 |

Table S2E: Energy ranking for the $B_5H_5Fe(CO)_2$ structures at the PBE0/def2-TZVP level of theory.

| No. | Initial structure | Final Energy (a.u.) | Relative Energy (kcal/mol) |
|---|---|---|---|
| 1 | 07v-04-CapOh—fe2—3co-pbe0 | -1755.5295190 | 0.00 |
| 2 | 07v-04-CapOh—fe1—3co-pbe0 | -1755.5293640 | 0.10 |
| 3 | 07v-01-PentBipyr—fe1—3co-pbe0 | -1755.5252250 | 2.69 |
| 4 | 07v-03-TricapTd—fe3—3co-pbe0 | -1755.5252000 | 2.71 |
| 5 | 07v-03-TricapTd—fe1—3co-pbe0 | -1755.5251990 | 2.71 |
| 6 | 07v-04-CapOh—fe3—3co-pbe0 | -1755.5097830 | 12.38 |
| 7 | 07v-01-PentBipyr—fe2—3co-pbe0 | -1755.5097810 | 12.39 |
| 8 | 07v-04-CapOh—fe4—3co-pbe0 | -1755.5097730 | 12.39 |
| 9 | 07v-03-TricapTd—fe2—3co-pbe0 | -1755.4751750 | 34.10 |
| 10 | 07v-02-HexPyr—fe1—3co-pbe0 | -1755.3905180 | 87.22 |

Table S2F: Energy ranking for the $B_5H_5Fe(CO)_2$ structures at the PBE0/def2-TZVP level of theory.

| No. | Initial structure | Final Energy (a.u.) | Relative Energy (kcal/mol) |
|---|---|---|---|
| 1 | 07v-01-PentBipyr—fe1—4co-pbe0 | -1868.7179040 | 0.00 |
| 2 | 07v-04-CapOh—fe4—4co-pbe0 | -1868.7178860 | 0.01 |
| 3 | 07v-04-CapOh—fe3—4co-pbe0 | -1868.7178650 | 0.02 |
| 4 | 07v-01-PentBipyr—fe2—4co-pbe0 | -1868.7096580 | 5.17 |
| 5 | 07v-03-TricapTd—fe1—4co-pbe0 | -1868.7047190 | 8.27 |
| 6 | 07v-04-CapOh—fe2—4co-pbe0 | -1868.6999020 | 11.30 |
| 7 | 07v-04-CapOh—fe1—4co-pbe0 | -1868.6997950 | 11.36 |
| 8 | 07v-03-TricapTd—fe3—4co-pbe0 | -1868.6981570 | 12.39 |
| 9 | 07v-02-HexPyr—fe1—4co-pbe0 | -1868.6956320 | 13.98 |
| 10 | 07v-03-TricapTd—fe2—4co-pbe0 | -1868.6458220 | 45.23 |

Table S3A: Distance table for the lowest-lying $B_7H_7Fe(CO)_2$ optimized structures obtained at the PBE0/def2-TZVP level of theory. Included are the zero-point corrected absolute energy in (a.u.) at the DLPNO-CCSD(T)/def2-QZVP level of theory with zero-point energy obtained from the PBE0/def2-TZVP computations, relative energies in (kcal/mol) and symmetry. For clarity, only the atoms forming the cluster framework are shown.

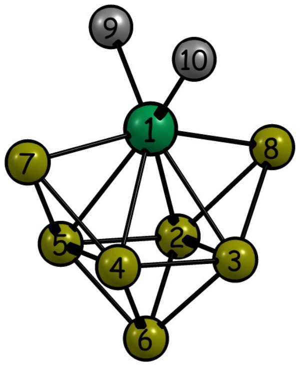


1. -1666.84657237 0.0 $C_{2v}$

| | | 1 | 2 | 3 | 4 | 5 |
|---|---|---|---|---|---|---|
| 1 | Fe | 0.000000 | | | | |
| 2 | B | 2.102040 | 0.000000 | | | |
| 3 | B | 2.102707 | 1.821821 | 0.000000 | | |
| 4 | B | 2.102263 | 2.523992 | 1.747769 | 0.000000 | |
| 5 | B | 2.102311 | 1.748079 | 2.524387 | 1.820319 | 0.000000 |
| 6 | B | 2.872343 | 1.735127 | 1.735490 | 1.735593 | 1.735109 |
| 7 | B | 1.859174 | 3.143166 | 3.142640 | 1.869266 | 1.870536 |
| 8 | B | 1.858881 | 1.869868 | 1.870923 | 3.142933 | 3.142896 |

| | | 6 | 7 | 8 |
|---|---|---|---|---|
| 6 | B | 0.000000 | | |
| 7 | B | 3.110906 | 0.000000 | |
| 8 | B | 3.110546 | 3.650285 | 0.000000 |

Fe1-C9: 1.761978 Fe1-C10: 1.762089

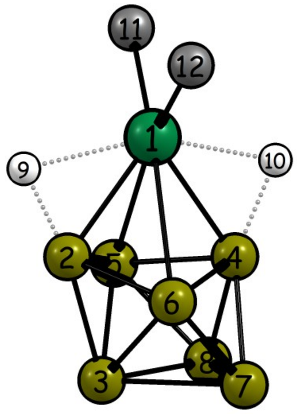


2. -1666.83429315 +7.7 $C_s$

| | | 1 | 2 | 3 | 4 | 5 |
|---|---|---|---|---|---|---|
| 1 | Fe | 0.000000 | | | | |
| 2 | B | 1.865699 | 0.000000 | | | |
| 3 | B | 3.180436 | 1.682545 | 0.000000 | | |
| 4 | B | 1.937764 | 2.220271 | 2.320984 | 0.000000 | |
| 5 | B | 2.307739 | 1.721842 | 1.909788 | 1.832778 | 0.000000 |
| 6 | B | 2.309282 | 1.721731 | 1.908518 | 1.833600 | 2.692223 |
| 7 | B | 3.356889 | 2.778746 | 1.849455 | 1.720569 | 2.734939 |
| 8 | B | 3.356658 | 2.778310 | 1.849108 | 1.720880 | 1.726870 |
| 9 | H | 1.734753 | 1.303098 | 2.972636 | 3.075775 | 2.593087 |
| 10 | H | 1.671766 | 2.967962 | 3.568879 | 1.333183 | 2.664282 |

| | | 6 | 7 | 8 | 9 | 10 |
|---|---|---|---|---|---|---|
| 6 | B | 0.000000 | | | | |
| 7 | B | 1.727088 | 0.000000 | | | |
| 8 | B | 2.734819 | 1.670407 | 0.000000 | | |
| 9 | H | 2.591339 | 3.951977 | 3.952418 | 0.000000 | |
| 10 | H | 2.665162 | 2.919183 | 2.919792 | 3.333335 | 0.000000 |

Fe1-C11: 1.795232 Fe1-C12: 1.795345

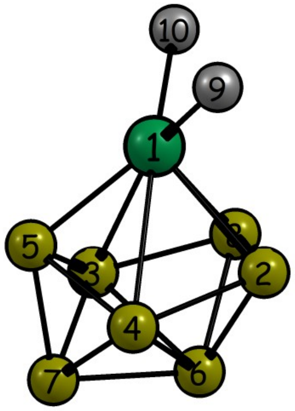


3. -1666.83343919 +8.2 $C_s$

| | | 1 | 2 | 3 | 4 | 5 |
|---|---|---|---|---|---|---|
| 1 | Fe | 0.000000 | | | | |
| 2 | B | 1.997095 | 0.000000 | | | |
| 3 | B | 2.302038 | 2.758541 | 0.000000 | | |
| 4 | B | 2.300711 | 1.759702 | 2.531566 | 0.000000 | |
| 5 | B | 1.873494 | 2.792622 | 1.758300 | 1.757899 | 0.000000 |
| 6 | B | 2.843236 | 1.783377 | 1.888607 | 1.887817 | 2.617808 |
| 7 | B | 3.055831 | 2.908498 | 1.736956 | 1.736135 | 1.683753 |
| 8 | B | 1.997019 | 1.782540 | 1.759337 | 2.758170 | 2.791996 |

| | | 6 | 7 | 8 |
|---|---|---|---|---|
| 6 | B | 0.000000 | | |
| 7 | B | 1.763994 | 0.000000 | |
| 8 | B | 1.784434 | 2.908700 | 0.000000 |

Fe1-C9: 1.798472 Fe1-C10: 1.798620

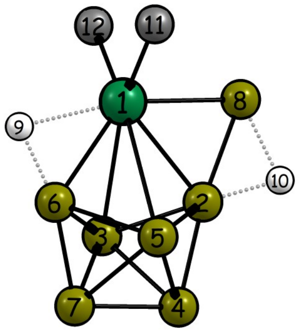


4. -1666.82935339 +10.8 $C_s$

| | | 1 | 2 | 3 | 4 | 5 |
|---|---|---|---|---|---|---|
| 1 | Fe | 0.000000 | | | | |
| 2 | B | 1.999578 | 0.000000 | | | |
| 3 | B | 2.384013 | 1.782069 | 0.000000 | | |
| 4 | B | 3.242371 | 1.639214 | 1.813058 | 0.000000 | |
| 5 | B | 2.384231 | 1.783644 | 2.456141 | 1.812877 | 0.000000 |
| 6 | B | 1.878221 | 2.394708 | 1.800954 | 2.556772 | 1.799497 |
| 7 | B | 3.189040 | 2.601953 | 1.846056 | 1.682228 | 1.846386 |
| 8 | B | 1.906928 | 1.673179 | 3.007346 | 3.305242 | 3.005055 |
| 9 | H | 1.716811 | 3.183789 | 2.732373 | 3.759319 | 2.731911 |
| 10 | H | 2.816526 | 1.298497 | 2.859922 | 2.532226 | 2.860573 |

| | | 6 | 7 | 8 | 9 | 10 |
|---|---|---|---|---|---|---|
| 6 | B | 0.000000 | | | | |
| 7 | B | 1.601260 | 0.000000 | | | |
| 8 | B | 3.385332 | 4.122410 | 0.000000 | | |
| 9 | H | 1.282327 | 2.858624 | 3.605061 | 0.000000 | |
| 10 | H | 3.665429 | 3.834771 | 1.386533 | 4.304271 | 0.000000 |

Fe1-C11: 1.765565 Fe1-C12: 1.765276

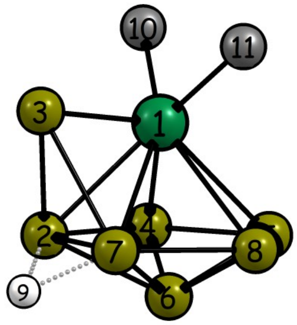


5. -1666.82302361 +14.8 $C_s$

| | | 1 | 2 | 3 | 4 | 5 |
|---|---|---|---|---|---|---|
| 1 | Fe | 0.000000 | | | | |
| 2 | B | 2.102367 | 0.000000 | | | |
| 3 | B | 1.765584 | 2.037927 | 0.000000 | | |
| 4 | B | 2.148881 | 1.848243 | 3.260310 | 0.000000 | |
| 5 | B | 2.232361 | 2.938188 | 3.807499 | 1.709516 | 0.000000 |
| 6 | B | 2.248640 | 1.776131 | 3.120553 | 1.636202 | 1.637787 |
| 7 | B | 2.100550 | 1.846848 | 2.034178 | 2.921928 | 2.938273 |
| 8 | B | 2.147249 | 2.922607 | 3.257696 | 2.773802 | 1.710047 |
| 9 | H | 2.860335 | 1.361435 | 2.308193 | 3.000814 | 3.642936 |

| | | 6 | 7 | 8 | 9 |
|---|---|---|---|---|---|
| 6 | B | 0.000000 | | | |
| 7 | B | 1.776060 | 0.000000 | | |
| 8 | B | 1.636346 | 1.848705 | 0.000000 | |
| 9 | H | 2.136376 | 1.360322 | 3.000460 | 0.000000 |

Fe1-C10: 1.748291 Fe1-C11: 1.748010

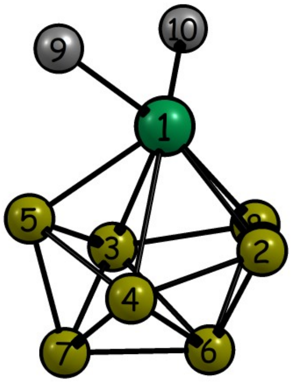


6. -1666.80857151 +23.8 $C_1$

| | | 1 | 2 | 3 | 4 | 5 |
|---|---|---|---|---|---|---|
| 1 | Fe | 0.000000 | | | | |
| 2 | B | 1.950473 | 0.000000 | | | |
| 3 | B | 2.214190 | 2.659683 | 0.000000 | | |
| 4 | B | 2.277838 | 1.726657 | 2.477668 | 0.000000 | |
| 5 | B | 1.982269 | 2.878517 | 1.753752 | 1.841440 | 0.000000 |
| 6 | B | 2.850687 | 1.749718 | 1.919179 | 1.920566 | 2.755209 |
| 7 | B | 3.040617 | 2.835309 | 1.739104 | 1.718811 | 1.719866 |
| 8 | B | 2.080117 | 1.745476 | 1.783666 | 2.775410 | 2.978830 |

| | | 6 | 7 | 8 |
|---|---|---|---|---|
| 6 | B | 0.000000 | | |
| 7 | B | 1.765857 | 0.000000 | |
| 8 | B | 1.735257 | 2.901518 | 0.000000 |

Fe1-C9: 1.825276 Fe1-C10: 1.785809

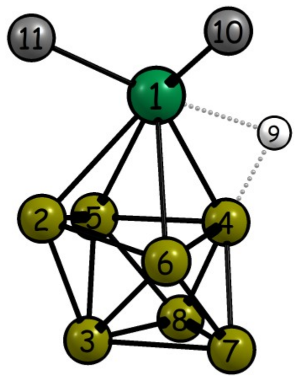


7. -1666.80378583 +26.8 $C_1$

| | | 1 | 2 | 3 | 4 | 5 |
|---|---|---|---|---|---|---|
| 1 | Fe | 0.000000 | | | | |
| 2 | B | 2.105276 | 0.000000 | | | |
| 3 | B | 3.217578 | 1.730990 | 0.000000 | | |
| 4 | B | 1.973059 | 2.489232 | 2.317212 | 0.000000 | |
| 5 | B | 2.260839 | 1.745841 | 1.875007 | 1.821475 | 0.000000 |
| 6 | B | 2.229591 | 1.773323 | 1.890099 | 1.839553 | 2.544396 |
| 7 | B | 3.331367 | 2.917414 | 1.820117 | 1.676408 | 2.697235 |
| 8 | B | 3.353466 | 2.898927 | 1.804859 | 1.693868 | 1.744082 |
| 9 | H | 1.700085 | 3.222027 | 3.526633 | 1.287346 | 2.650117 |

| | | 6 | 7 | 8 | 9 |
|---|---|---|---|---|---|
| 6 | B | 0.000000 | | | |
| 7 | B | 1.770986 | 0.000000 | | |
| 8 | B | 2.723301 | 1.673207 | 0.000000 | |
| 9 | H | 2.590183 | 2.807693 | 2.869915 | 0.000000 |

Fe1-C10: 1.785772 Fe1-C11: 1.780102

Table S3B: Distance table for the lowest-lying $B_7H_7Fe(CO)_3$ optimized structures obtained at the PBE0/def2-TZVP level of theory. Included are the zero-point corrected absolute energy in (a.u.) at the DLPNO-CCSD(T)/def2-QZVP level of theory with zero-point energy obtained from the PBE0/def2-TZVP computations, relative energies in (kcal/mol) and symmetry. For clarity, only the atoms forming the cluster framework are shown.

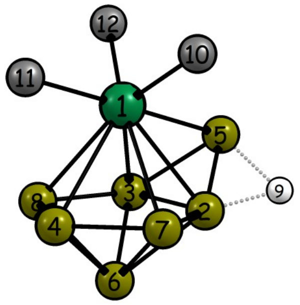


1. -1780.03928650 0.0 $C_1$

| | | 1 | 2 | 3 | 4 | 5 |
|---|---|---|---|---|---|---|
| 1 | Fe | 0.000000 | | | | |
| 2 | B | 2.147447 | 0.000000 | | | |
| 3 | B | 2.161610 | 1.721111 | 0.000000 | | |
| 4 | B | 2.259869 | 2.637820 | 2.748413 | 0.000000 | |
| 5 | B | 2.185750 | 1.595179 | 1.749216 | 3.710318 | 0.000000 |
| 6 | B | 2.787460 | 1.794836 | 1.868779 | 1.761192 | 3.044369 |
| 7 | B | 2.242244 | 1.644248 | 2.792326 | 1.660911 | 3.027745 |
| 8 | B | 2.202412 | 2.682794 | 1.677153 | 1.715204 | 3.172895 |
| 9 | H | 2.922333 | 1.270402 | 2.546467 | 3.870759 | 1.454630 |

| | | 6 | 7 | 8 | 9 |
|---|---|---|---|---|---|
| 6 | B | 0.000000 | | | |
| 7 | B | 1.846678 | 0.000000 | | |
| 8 | B | 1.798299 | 2.755951 | 0.000000 | |
| 9 | H | 2.988031 | 2.642216 | 3.845398 | 0.000000 |

Fe1-C10: 1.777579 Fe1-C11: 1.763497 Fe1-C12: 1.772626

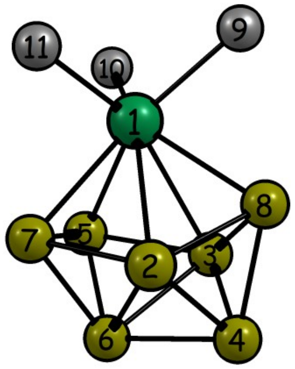


2. -1780.03376977 +3.5 $C_s$

| | | 1 | 2 | 3 | 4 | 5 |
|---|---|---|---|---|---|---|
| 1 | Fe | 0.000000 | | | | |
| 2 | B | 2.257277 | 0.000000 | | | |
| 3 | B | 2.259576 | 2.477446 | 0.000000 | | |
| 4 | B | 3.080303 | 1.741514 | 1.736625 | 0.000000 | |
| 5 | B | 2.042086 | 2.749617 | 1.768151 | 2.881317 | 0.000000 |
| 6 | B | 2.844996 | 1.922223 | 1.925358 | 1.756037 | 1.735647 |
| 7 | B | 2.045829 | 1.769402 | 2.752543 | 2.884327 | 1.792124 |
| 8 | B | 2.026539 | 1.779446 | 1.789127 | 1.728771 | 2.958533 |

| | | 6 | 7 | 8 |
|---|---|---|---|---|
| 6 | B | 0.000000 | | |
| 7 | B | 1.731928 | 0.000000 | |
| 8 | B | 2.741293 | 2.958002 | 0.000000 |

Fe1-C9: 1.795276 Fe1-C10: 1.778454 Fe1-C11: 1.777317

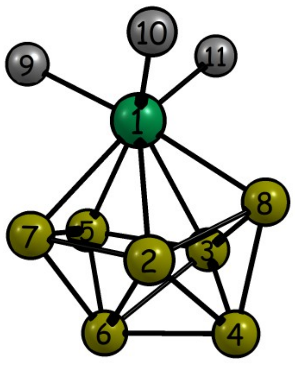


3. -1780.03208419 +4.5 $C_s$

| | | 1 | 2 | 3 | 4 | 5 |
|---|---|---|---|---|---|---|
| 1 | Fe | 0.000000 | | | | |
| 2 | B | 2.208063 | 0.000000 | | | |
| 3 | B | 2.207467 | 2.453906 | 0.000000 | | |
| 4 | B | 3.035132 | 1.742069 | 1.740567 | 0.000000 | |
| 5 | B | 2.071236 | 2.757553 | 1.732057 | 2.883616 | 0.000000 |
| 6 | B | 2.834722 | 1.930284 | 1.933242 | 1.789898 | 1.736054 |
| 7 | B | 2.071936 | 1.731395 | 2.759650 | 2.885433 | 1.879049 |
| 8 | B | 2.007194 | 1.794696 | 1.795857 | 1.717909 | 2.990899 |

| | | 6 | 7 | 8 |
|---|---|---|---|---|
| 6 | B | 0.000000 | | |
| 7 | B | 1.735838 | 0.000000 | |
| 8 | B | 2.782550 | 2.990723 | 0.000000 |

Fe1-C9: 1.778689 Fe1-C10: 1.784510 Fe1-C11: 1.784446

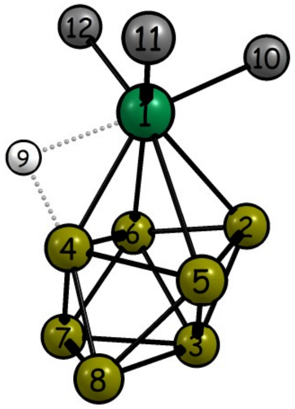


4. -1780.02574355 +8.5 $C_s$

| | | 1 | 2 | 3 | 4 | 5 |
|---|---|---|---|---|---|---|
| 1 | Fe | 0.000000 | | | | |
| 2 | B | 2.126129 | 0.000000 | | | |
| 3 | B | 3.239214 | 1.725855 | 0.000000 | | |
| 4 | B | 1.965681 | 2.447023 | 2.287301 | 0.000000 | |
| 5 | B | 2.288411 | 1.758607 | 1.878278 | 1.831810 | 0.000000 |
| 6 | B | 2.288099 | 1.758674 | 1.878945 | 1.831459 | 2.580144 |
| 7 | B | 3.362593 | 2.909802 | 1.818892 | 1.682146 | 2.729192 |
| 8 | B | 3.362430 | 2.909688 | 1.818531 | 1.682024 | 1.769120 |
| 9 | H | 1.652316 | 3.190883 | 3.520208 | 1.309676 | 2.637573 |

| | | 6 | 7 | 8 | 9 |
|---|---|---|---|---|---|
| 6 | B | 0.000000 | | | |
| 7 | B | 1.769214 | 0.000000 | | |
| 8 | B | 2.728998 | 1.673554 | 0.000000 | |
| 9 | H | 2.636499 | 2.852550 | 2.852806 | 0.000000 |

Fe1-C10: 1.781552 Fe1-C11: 1.791627 Fe1-C12: 1.791573

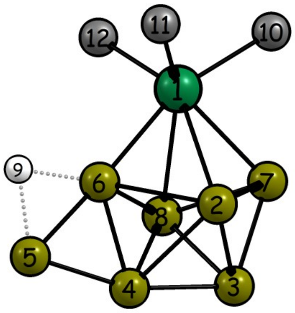


5. -1780.00943579 +18.7 $C_s$

| | | 1 | 2 | 3 | 4 | 5 |
|---|---|---|---|---|---|---|
| 1 | Fe | 0.000000 | | | | |
| 2 | B | 2.192486 | 0.000000 | | | |
| 3 | B | 3.104249 | 1.791360 | 0.000000 | | |
| 4 | B | 3.148367 | 1.953598 | 1.690497 | 0.000000 | |
| 5 | B | 3.533101 | 2.931107 | 3.305822 | 1.656901 | 0.000000 |
| 6 | B | 1.924096 | 1.912317 | 2.647764 | 1.669169 | 1.610113 |
| 7 | B | 1.986720 | 1.790484 | 1.691468 | 2.791336 | 4.021460 |
| 8 | B | 2.194187 | 2.371257 | 1.790916 | 1.953908 | 2.931713 |
| 9 | H | 2.878796 | 3.050965 | 3.860887 | 2.507563 | 1.331769 |

| | | 6 | 7 | 8 | 9 |
|---|---|---|---|---|---|
| 6 | B | 0.000000 | | | |
| 7 | B | 2.708133 | 0.000000 | | |
| 8 | B | 1.911268 | 1.791560 | 0.000000 | |
| 9 | H | 1.318166 | 4.016062 | 3.050880 | 0.000000 |

Fe1-C10: 1.764746 Fe1-C11: 1.761927 Fe1-C12: 1.762296

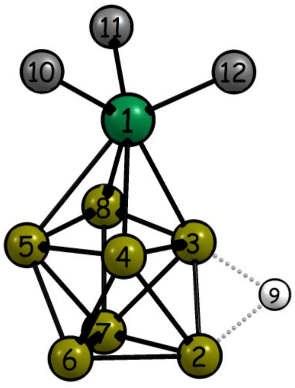


6. -1780.00924293 +18.9 $C_1$

| | | 1 | 2 | 3 | 4 | 5 |
|---|---|---|---|---|---|---|
| 1 | Fe | 0.000000 | | | | |
| 2 | B | 3.342649 | 0.000000 | | | |
| 3 | B | 1.958021 | 1.650523 | 0.000000 | | |
| 4 | B | 2.139190 | 1.978402 | 1.878029 | 0.000000 | |
| 5 | B | 2.196880 | 2.795921 | 2.282434 | 1.861676 | 0.000000 |
| 6 | B | 3.344243 | 1.773265 | 2.460882 | 1.715178 | 1.749519 |
| 7 | B | 3.164552 | 1.883477 | 1.874223 | 2.575865 | 1.885223 |
| 8 | B | 2.032204 | 2.935198 | 1.705427 | 2.797365 | 1.817785 |
| 9 | H | 3.111732 | 1.374484 | 1.321584 | 2.544156 | 3.429046 |

| | | 6 | 7 | 8 | 9 |
|---|---|---|---|---|---|
| 6 | B | 0.000000 | | | |
| 7 | B | 1.728079 | 0.000000 | | |
| 8 | B | 2.896932 | 1.743045 | 0.000000 | |
| 9 | H | 2.982550 | 2.547848 | 2.904825 | 0.000000 |

Fe1-C10: 1.774584 Fe1-C11: 1.769862 Fe1-C12: 1.775078

Table S3C: Distance table for the lowest-lying $B_7H_7Fe(CO)_4$ optimized structures obtained at the PBE0/def2-TZVP level of theory. Included are the zero-point corrected absolute energy in (a.u.) at the DLPNO-CCSD(T)/def2-QZVP level of theory with zero-point energy obtained from the PBE0/def2-TZVP computations, relative energies in (kcal/mol) and symmetry. For clarity, only the atoms forming the cluster framework are shown.

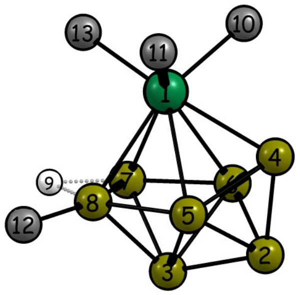


1. -1893.19234118 0.0 $C_1$

| | | 1 | 2 | 3 | 4 | 5 |
|---|---|---|---|---|---|---|
| 1 | Fe | 0.000000 | | | | |
| 2 | B | 3.077244 | 0.000000 | | | |
| 3 | B | 2.976875 | 1.697162 | 0.000000 | | |
| 4 | B | 2.072993 | 1.638685 | 2.721251 | 0.000000 | |
| 5 | B | 2.226319 | 1.755614 | 1.914252 | 1.793341 | 0.000000 |
| 6 | B | 2.271860 | 1.772653 | 1.886933 | 1.847623 | 2.466784 |
| 7 | B | 2.204489 | 2.931754 | 1.844900 | 3.066999 | 2.771115 |
| 8 | B | 2.125635 | 2.906288 | 1.799134 | 3.032841 | 1.803512 |
| 9 | H | 2.600153 | 3.799706 | 2.427924 | 3.913566 | 3.050383 |

| | | 6 | 7 | 8 | 9 |
|---|---|---|---|---|---|
| 6 | B | 0.000000 | | | |
| 7 | B | 1.729732 | 0.000000 | | |
| 8 | B | 2.662243 | 1.727793 | 0.000000 | |
| 9 | H | 2.992998 | 1.371993 | 1.322302 | 0.000000 |

Fe1-C10: 1.762364 Fe1-C11: 1.768173
Fe1-C12: 3.259413 Fe1-C13: 1.780054

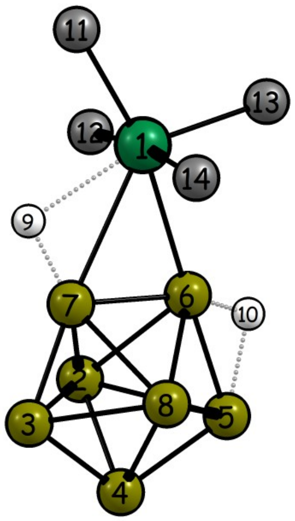


2. -1893.18697177 +3.4 $C_s$

| | | 1 | 2 | 3 | 4 | 5 |
|---|---|---|---|---|---|---|
| 1 | Fe | 0.000000 | | | | |
| 2 | B | 3.470972 | 0.000000 | | | |
| 3 | B | 3.838340 | 1.822927 | 0.000000 | | |
| 4 | B | 4.519873 | 1.733712 | 1.681748 | 0.000000 | |
| 5 | B | 3.798511 | 2.002749 | 2.783733 | 1.710652 | 0.000000 |
| 6 | B | 2.094994 | 1.987246 | 2.696288 | 2.678473 | 1.704042 |
| 7 | B | 2.227875 | 1.784233 | 1.618927 | 2.596260 | 2.676237 |
| 8 | B | 3.358713 | 2.373828 | 1.827260 | 1.814544 | 1.804735 |
| 9 | H | 1.799731 | 2.895074 | 2.602755 | 3.847271 | 3.866769 |
| 10 | H | 2.999487 | 2.333250 | 3.546323 | 2.941512 | 1.433606 |

| | | 6 | 7 | 8 | 9 | 10 |
|---|---|---|---|---|---|---|
| 6 | B | 0.000000 | | | | |
| 7 | B | 1.604679 | 0.000000 | | | |
| 8 | B | 1.826328 | 1.857750 | 0.000000 | | |
| 9 | H | 2.469017 | 1.273399 | 2.917740 | 0.000000 | |
| 10 | H | 1.258779 | 2.747640 | 2.622551 | 3.650172 | 0.000000 |

Fe1-C11: 1.798094 Fe1-C12: 1.820220
Fe1-C13: 1.766618 Fe1-C14: 1.824167

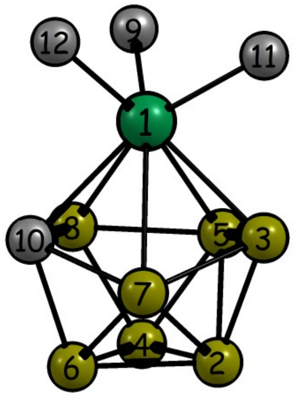


3. -1893.18565304 +4.2 $C_1$

| | | 1 | 2 | 3 | 4 | 5 |
|---|---|---|---|---|---|---|
| 1 | Fe | 0.000000 | | | | |
| 2 | B | 3.085580 | 0.000000 | | | |
| 3 | B | 2.027297 | 1.730880 | 0.000000 | | |
| 4 | B | 3.281964 | 1.745292 | 2.871843 | 0.000000 | |
| 5 | B | 2.190890 | 1.909593 | 1.769340 | 1.730058 | 0.000000 |
| 6 | B | 3.145487 | 1.774382 | 2.873699 | 1.718191 | 2.685060 |
| 7 | B | 2.300415 | 1.824636 | 1.757276 | 2.867367 | 2.697121 |
| 8 | B | 2.151942 | 2.676392 | 2.910204 | 1.718380 | 1.846516 |

| | | 6 | 7 | 8 |
|---|---|---|---|---|
| 6 | B | 0.000000 | | |
| 7 | B | 1.820862 | 0.000000 | |
| 8 | B | 2.047981 | 2.715864 | 0.000000 |

Fe1-C9: 1.789081 Fe1-C10: 2.025917
Fe1-C11: 1.787162 Fe1-C12: 1.807767

Table S3D: Energy ranking for the $B_5H_5Fe(CO)_2$ structures at the PBE0/def2-TZVP level of theory.

| No. | Initial structure | Final Energy (a.u.) | Relative Energy (kcal/mol) |
|---|---|---|---|
| 1 | 08v-01-Bisdisph—fe4—2co-pbe0 | -1667.7166160 | 0.00 |
| 2 | 08v-01-Bisdisph—fe2—2co-pbe0 | -1667.7166110 | 0.00 |
| 3 | 08v-08-Nido—fe1—2co-pbe0 | -1667.7164600 | 0.10 |
| 4 | 08v-10-DicapTrPrB—fe2—2co-pbe0 | -1667.7164590 | 0.10 |
| 5 | 08v-03-Antipr—fe1—2co-pbe0 | -1667.7164470 | 0.11 |
| 6 | 08v-01-Bisdisph—fe3—2co-pbe0 | -1667.7164430 | 0.11 |
| 7 | 08v-01-Bisdisph—fe1—2co-pbe0 | -1667.7164240 | 0.12 |
| 8 | 08v-10-DicapTrPrB—fe3—2co-pbe0 | -1667.7164240 | 0.12 |
| 9 | 08v-11-DicapOh—fe3—2co-pbe0 | -1667.7075540 | 5.69 |
| 10 | 08v-06-Tdallcap—fe1—2co-pbe0 | -1667.7074100 | 5.78 |
| 11 | 08v-09-DicapTrPrA—fe2—2co-pbe0 | -1667.7074040 | 5.78 |
| 12 | 08v-04-TrigPyr—fe3—2co-pbe0 | -1667.7015330 | 9.46 |
| 13 | 08v-09-DicapTrPrA—fe3—2co-pbe0 | -1667.7015040 | 9.48 |
| 14 | 08v-09-DicapTrPrA—fe1—2co-pbe0 | -1667.6981990 | 11.56 |
| 15 | 08v-02-Cube—fe1—2co-pbe0 | -1667.6844140 | 20.21 |
| 16 | 08v-07-Bipirhex—fe1—2co-pbe0 | -1667.6843850 | 20.23 |
| 17 | 08v-05-AntiprTrig—fe3—2co-pbe0 | -1667.6818290 | 21.83 |
| 18 | 08v-05-AntiprTrig—fe2—2co-pbe0 | -1667.6818130 | 21.84 |
| 19 | 08v-08-Nido—fe2—2co-pbe0 | -1667.6732350 | 27.22 |
| 20 | 08v-10-DicapTrPrB—fe1—2co-pbe0 | -1667.6704210 | 28.99 |
| 21 | 08v-07-Bipirhex—fe2—2co-pbe0 | -1667.6693680 | 29.65 |
| 22 | 08v-09-DicapTrPrA—fe4—2co-pbe0 | -1667.6693400 | 29.67 |
| 23 | 08v-04-TrigPyr—fe1—2co-pbe0 | -1667.6681960 | 30.38 |
| 24 | 08v-11-DicapOh—fe2—2co-pbe0 | -1667.6675110 | 30.81 |
| 25 | 08v-05-AntiprTrig—fe1—2co-pbe0 | -1667.6653300 | 32.18 |
| 26 | 08v-04-TrigPyr—fe2—2co-pbe0 | -1667.6653000 | 32.20 |
| 27 | 08v-06-Tdallcap—fe2—2co-pbe0 | -1667.6558230 | 38.15 |
| 28 | 08v-11-DicapOh—fe1—2co-pbe0 | -1667.6195900 | 60.88 |

Table S3E: Energy ranking for the $B_5H_5Fe(CO)_2$ structures at the PBE0/def2-TZVP level of theory.

| No. | Initial structure | Final Energy (a.u.) | Relative Energy (kcal/mol) |
|---|---|---|---|
| 1 | 08v-06-Tdallcap—fe1—3co-pbe0 | -1780.9434740 | 0.00 |
| 2 | 08v-11-DicapOh—fe2—3co-pbe0 | -1780.9434390 | 0.02 |
| 3 | 08v-02-Cube—fe1—3co-pbe0 | -1780.9434210 | 0.03 |
| 4 | 08v-09-DicapTrPrA—fe2—3co-pbe0 | -1780.9394840 | 2.50 |
| 5 | 08v-09-DicapTrPrA—fe3—3co-pbe0 | -1780.9394680 | 2.51 |
| 6 | 08v-06-Tdallcap—fe2—3co-pbe0 | -1780.9394540 | 2.52 |
| 7 | 08v-01-Bisdisph—fe3—3co-pbe0 | -1780.9394460 | 2.53 |
| 8 | 08v-01-Bisdisph—fe1—3co-pbe0 | -1780.9394440 | 2.53 |
| 9 | 08v-03-Antipr—fe1—3co-pbe0 | -1780.9394440 | 2.53 |
| 10 | 08v-08-Nido—fe1—3co-pbe0 | -1780.9394310 | 2.54 |
| 11 | 08v-10-DicapTrPrB—fe2—3co-pbe0 | -1780.9394230 | 2.54 |
| 12 | 08v-01-Bisdisph—fe4—3co-pbe0 | -1780.9332510 | 6.42 |
| 13 | 08v-01-Bisdisph—fe2—3co-pbe0 | -1780.9332490 | 6.42 |
| 14 | 08v-10-DicapTrPrB—fe3—3co-pbe0 | -1780.9331910 | 6.45 |
| 15 | 08v-08-Nido—fe2—3co-pbe0 | -1780.9137430 | 18.66 |
| 16 | 08v-10-DicapTrPrB—fe1—3co-pbe0 | -1780.9137360 | 18.66 |
| 17 | 08v-11-DicapOh—fe3—3co-pbe0 | -1780.9136460 | 18.72 |
| 18 | 08v-07-Bipirhex—fe2—3co-pbe0 | -1780.9123290 | 19.54 |
| 19 | 08v-09-DicapTrPrA—fe4—3co-pbe0 | -1780.9123280 | 19.54 |
| 20 | 08v-11-DicapOh—fe1—3co-pbe0 | -1780.9123270 | 19.55 |
| 21 | 08v-09-DicapTrPrA—fe1—3co-pbe0 | -1780.9123200 | 19.55 |
| 22 | 08v-05-AntiprTrig—fe1—3co-pbe0 | -1780.9037200 | 24.95 |
| 23 | 08v-04-TrigPyr—fe2—3co-pbe0 | -1780.9037190 | 24.95 |
| 24 | 08v-07-Bipirhex—fe1—3co-pbe0 | -1780.8972530 | 29.00 |
| 25 | 08v-05-AntiprTrig—fe2—3co-pbe0 | -1780.8965590 | 29.44 |
| 26 | 08v-05-AntiprTrig—fe3—3co-pbe0 | -1780.8965250 | 29.46 |
| 27 | 08v-04-TrigPyr—fe3—3co-pbe0 | -1780.8510240 | 58.01 |
| 28 | 08v-04-TrigPyr—fe1—3co-pbe0 | -1780.8466630 | 60.75 |

Table S3F: Energy ranking for the $B_5H_5Fe(CO)_2$ structures at the PBE0/def2-TZVP level of theory.

| No. | Initial structure | Final Energy (a.u.) | Relative Energy (kcal/mol) |
|---|---|---|---|
| 1 | 08v-03-Antipr—fe1—4co-pbe0 | -1894.1465700 | 0.00 |
| 2 | 08v-01-Bisdisph—fe3—4co-pbe0 | -1894.1354150 | 7.00 |
| 3 | 08v-01-Bisdisph—fe1—4co-pbe0 | -1894.1354010 | 7.01 |
| 4 | 08v-07-Bipirhex—fe2—4co-pbe0 | -1894.1207620 | 16.19 |
| 5 | 08v-06-Tdallcap—fe1—4co-pbe0 | -1894.1142370 | 20.29 |
| 6 | 08v-10-DicapTrPrB—fe2—4co-pbe0 | -1894.1102260 | 22.81 |
| 7 | 08v-06-Tdallcap—fe2—4co-pbe0 | -1894.1101700 | 22.84 |
| 8 | 08v-08-Nido—fe1—4co-pbe0 | -1894.1100130 | 22.94 |
| 9 | 08v-01-Bisdisph—fe4—4co-pbe0 | -1894.1093600 | 23.35 |
| 10 | 08v-10-DicapTrPrB—fe3—4co-pbe0 | -1894.1093430 | 23.36 |
| 11 | 08v-01-Bisdisph—fe2—4co-pbe0 | -1894.1093360 | 23.36 |
| 12 | 08v-10-DicapTrPrB—fe1—4co-pbe0 | -1894.1093320 | 23.37 |
| 13 | 08v-08-Nido—fe2—4co-pbe0 | -1894.1093190 | 23.38 |
| 14 | 08v-09-DicapTrPrA—fe4—4co-pbe0 | -1894.0993600 | 29.62 |
| 15 | 08v-09-DicapTrPrA—fe1—4co-pbe0 | -1894.0992900 | 29.67 |
| 16 | 08v-09-DicapTrPrA—fe2—4co-pbe0 | -1894.0958440 | 31.83 |
| 17 | 08v-11-DicapOh—fe2—4co-pbe0 | -1894.0954100 | 32.10 |
| 18 | 08v-11-DicapOh—fe1—4co-pbe0 | -1894.0950880 | 32.31 |
| 19 | 08v-05-AntiprTrig—fe1—4co-pbe0 | -1894.0945530 | 32.64 |
| 20 | 08v-04-TrigPyr—fe2—4co-pbe0 | -1894.0945380 | 32.65 |
| 21 | 08v-07-Bipirhex—fe1—4co-pbe0 | -1894.0901770 | 35.39 |
| 22 | 08v-09-DicapTrPrA—fe3—4co-pbe0 | -1894.0866460 | 37.60 |
| 23 | 08v-11-DicapOh—fe3—4co-i-65-pbe0 | -1894.0782490 | 42.87 |
| 24 | 08v-11-DicapOh—fe3—4co-r-65-pbe0 | -1894.0717410 | 46.96 |
| 25 | 08v-04-TrigPyr—fe3—4co-pbe0 | -1894.0621330 | 52.99 |
| 26 | 08v-04-TrigPyr—fe1—4co-pbe0 | -1894.0621180 | 52.99 |
| 27 | 08v-05-AntiprTrig—fe2—4co-pbe0 | -1894.0560380 | 56.81 |
| 28 | 08v-05-AntiprTrig—fe3—4co-pbe0 | -1894.0560220 | 56.82 |
| 29 | 08v-02-Cube—fe1—4co-pbe0 | -1894.0551270 | 57.38 |

Table S4A: Distance table for the lowest-lying $B_8H_8Fe(CO)_2$ optimized structures obtained at the PBE0/def2-TZVP level of theory. Included are the zero-point corrected absolute energy in (a.u.) at the DLPNO-CCSD(T)/def2-QZVP level of theory with zero-point energy obtained from the PBE0/def2-TZVP computations, relative energies in (kcal/mol) and symmetry. For clarity, only the atoms forming the cluster framework are shown.

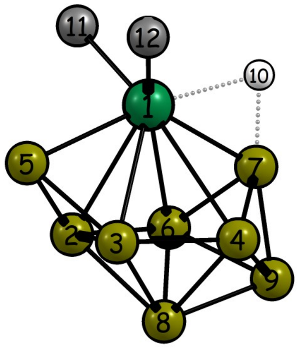


1. -1692.22795108 0.0 $C_s$

| | | 1 | 2 | 3 | 4 | 5 |
|---|---|---|---|---|---|---|
| 1 | Fe | 0.000000 | | | | |
| 2 | B | 2.191512 | 0.000000 | | | |
| 3 | B | 2.189665 | 1.710056 | 0.000000 | | |
| 4 | B | 2.388977 | 2.716681 | 1.702098 | 0.000000 | |
| 5 | B | 1.982027 | 1.711453 | 1.709123 | 3.141248 | 0.000000 |
| 6 | B | 2.391699 | 1.702844 | 2.716210 | 2.620188 | 3.143817 |
| 7 | B | 1.859741 | 2.748637 | 2.748012 | 1.785014 | 3.454633 |
| 8 | B | 3.044817 | 1.799541 | 1.799523 | 1.884704 | 3.064189 |
| 9 | B | 3.106685 | 2.881458 | 2.881032 | 1.798365 | 4.071532 |
| 10 | H | 1.714286 | 3.442772 | 3.443257 | 2.696123 | 3.682348 |

| | | 6 | 7 | 8 | 9 | 10 |
|---|---|---|---|---|---|---|
| 6 | B | 0.000000 | | | | |
| 7 | B | 1.785215 | 0.000000 | | | |
| 8 | B | 1.883624 | 2.544929 | 0.000000 | | |
| 9 | B | 1.797376 | 1.598051 | 1.721798 | 0.000000 | |
| 10 | H | 2.694643 | 1.285810 | 3.706315 | 2.871386 | 0.000000 |

Fe1-C11: 1.777580 Fe1-C12: 1.777494

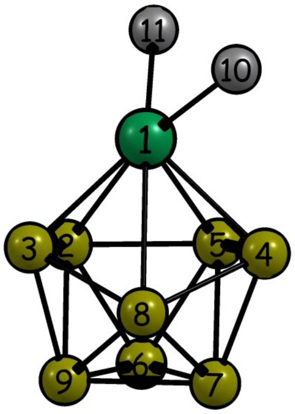


2. -1692.21877952 +5.8 $C_1$

| | | 1 | 2 | 3 | 4 | 5 |
|---|---|---|---|---|---|---|
| 1 | Fe | 0.000000 | | | | |
| 2 | B | 2.183423 | 0.000000 | | | |
| 3 | B | 1.904592 | 1.748938 | 0.000000 | | |
| 4 | B | 1.982089 | 2.931471 | 2.801849 | 0.000000 | |
| 5 | B | 2.120247 | 1.812766 | 2.797990 | 1.791959 | 0.000000 |
| 6 | B | 3.264087 | 1.714865 | 2.800583 | 2.865302 | 1.749393 |
| 7 | B | 3.054174 | 2.682555 | 2.794362 | 1.759083 | 2.009818 |
| 8 | B | 2.282887 | 2.746538 | 1.735193 | 1.773383 | 2.721483 |
| 9 | B | 3.059970 | 1.982334 | 1.725192 | 2.885407 | 2.698276 |

| | | 6 | 7 | 8 | 9 |
|---|---|---|---|---|---|
| 6 | B | 0.000000 | | | |
| 7 | B | 1.746181 | 0.000000 | | |
| 8 | B | 2.869648 | 1.783025 | 0.000000 | |
| 9 | B | 1.758315 | 1.796017 | 1.801758 | 0.000000 |

Fe1-C10: 1.810603 Fe1-C11: 1.815284

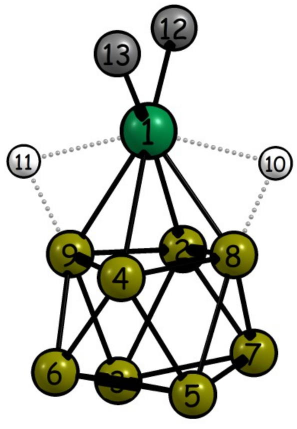

3. -1692.21455410 +8.4 $C_2$

| | | 1 | 2 | 3 | 4 | 5 |
|---|---|---|---|---|---|---|
| 1 | Fe | 0.000000 | | | | |
| 2 | B | 2.229237 | 0.000000 | | | |
| 3 | B | 3.433731 | 1.924046 | 0.000000 | | |
| 4 | B | 2.230812 | 2.871020 | 2.689179 | 0.000000 | |
| 5 | B | 3.435708 | 2.689886 | 1.823680 | 1.924195 | 0.000000 |
| 6 | B | 3.323170 | 2.963702 | 1.714786 | 1.691087 | 1.780416 |
| 7 | B | 3.323628 | 1.691133 | 1.780188 | 2.963001 | 1.714684 |
| 8 | B | 1.925565 | 1.781535 | 2.528441 | 1.822532 | 1.882084 |
| 9 | B | 1.924489 | 1.821702 | 1.878983 | 1.781488 | 2.527035 |
| 10 | H | 1.724090 | 2.556590 | 3.786153 | 2.566900 | 3.139025 |
| 11 | H | 1.720279 | 2.567254 | 3.137736 | 2.556008 | 3.785998 |

| | | 6 | 7 | 8 | 9 | 10 |
|---|---|---|---|---|---|---|
| 6 | B | 0.000000 | | | | |
| 7 | B | 2.911093 | 0.000000 | | | |
| 8 | B | 2.743012 | 1.722282 | 0.000000 | | |
| 9 | B | 1.721098 | 2.740807 | 2.166112 | 0.000000 | |
| 10 | H | 3.880935 | 2.853786 | 1.326508 | 3.002462 | 0.000000 |
| 11 | H | 2.852843 | 3.880540 | 3.002189 | 1.328095 | 3.348571 |

| | | 11 |
|---|---|---|
| 11 | H | 0.000000 |

Fe1-C12: 1.798416 Fe1-C13: 1.798437

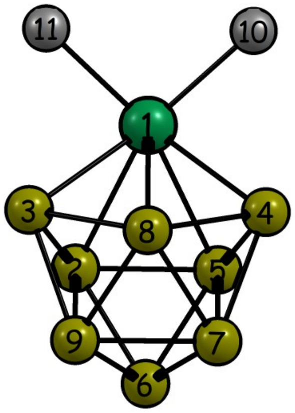

4. -1692.21150735 +10.3 $C_s$

| | | 1 | 2 | 3 | 4 | 5 |
|---|---|---|---|---|---|---|
| 1 | Fe | 0.000000 | | | | |
| 2 | B | 2.117031 | 0.000000 | | | |
| 3 | B | 2.007169 | 1.762198 | 0.000000 | | |
| 4 | B | 2.004478 | 2.892154 | 2.951065 | 0.000000 | |
| 5 | B | 2.120121 | 1.784236 | 2.895847 | 1.764762 | 0.000000 |
| 6 | B | 3.250840 | 1.734542 | 2.844622 | 2.845231 | 1.733660 |
| 7 | B | 3.051798 | 2.675544 | 2.880493 | 1.748970 | 1.998998 |
| 8 | B | 2.249018 | 2.688635 | 1.796167 | 1.793785 | 2.690937 |
| 9 | B | 3.051999 | 1.999195 | 1.747762 | 2.879816 | 2.677446 |

| | | 6 | 7 | 8 | 9 |
|---|---|---|---|---|---|
| 6 | B | 0.000000 | | | |
| 7 | B | 1.746411 | 0.000000 | | |
| 8 | B | 2.851402 | 1.784941 | 0.000000 | |
| 9 | B | 1.747291 | 1.775125 | 1.784767 | 0.000000 |

Fe1-C10: 1.804650 Fe1-C11: 1.804077

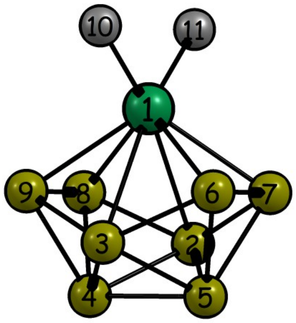

5. -1692.19361873 +21.5 $C_s$

| | | 1 | 2 | 3 | 4 | 5 |
|---|---|---|---|---|---|---|
| 1 | Fe | 0.000000 | | | | |
| 2 | B | 2.320384 | 0.000000 | | | |
| 3 | B | 2.319123 | 2.719685 | 0.000000 | | |
| 4 | B | 2.771051 | 1.796757 | 1.797359 | 0.000000 | |
| 5 | B | 2.776570 | 1.796594 | 1.796610 | 1.823627 | 0.000000 |
| 6 | B | 2.067139 | 2.758275 | 1.706472 | 2.920515 | 1.782387 |
| 7 | B | 2.066084 | 1.706239 | 2.757561 | 2.919881 | 1.782959 |
| 8 | B | 2.060762 | 1.710507 | 2.760859 | 1.782028 | 2.923322 |
| 9 | B | 2.061955 | 2.761943 | 1.710579 | 1.782086 | 2.923847 |

| | | 6 | 7 | 8 | 9 |
|---|---|---|---|---|---|
| 6 | B | 0.000000 | | | |
| 7 | B | 1.726105 | 0.000000 | | |
| 8 | B | 3.409223 | 2.938364 | 0.000000 | |
| 9 | B | 2.940724 | 3.409421 | 1.727914 | 0.000000 |

Fe1-C10: 1.817386 Fe1-C11: 1.817271

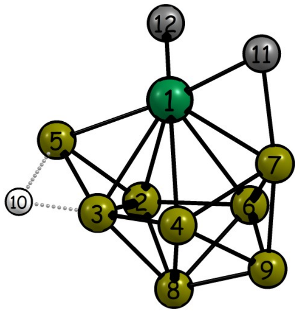


6. -1692.18422461 +27.4 $C_1$

| | | 1 | 2 | 3 | 4 | 5 |
|---|---|---|---|---|---|---|
| 1 | Fe | 0.000000 | | | | |
| 2 | B | 2.168453 | 0.000000 | | | |
| 3 | B | 2.100080 | 1.702084 | 0.000000 | | |
| 4 | B | 2.326303 | 2.749654 | 1.686680 | 0.000000 | |
| 5 | B | 2.053617 | 1.746224 | 1.620713 | 3.107137 | 0.000000 |
| 6 | B | 2.291610 | 1.752307 | 2.640384 | 2.535041 | 3.202857 |
| 7 | B | 2.053145 | 3.041550 | 2.932897 | 1.872551 | 3.767559 |
| 8 | B | 2.975428 | 1.798311 | 1.755580 | 1.898615 | 3.002330 |
| 9 | B | 3.076969 | 2.896951 | 2.809449 | 1.763873 | 4.063673 |
| 10 | H | 2.858360 | 2.543262 | 1.250357 | 2.697026 | 1.559120 |

| | | 6 | 7 | 8 | 9 | 10 |
|---|---|---|---|---|---|---|
| 6 | B | 0.000000 | | | | |
| 7 | B | 1.870887 | 0.000000 | | | |
| 8 | B | 1.879958 | 2.733859 | 0.000000 | | |
| 9 | B | 1.758778 | 1.666500 | 1.692503 | 0.000000 | |
| 10 | H | 3.827009 | 4.071063 | 2.850647 | 4.028239 | 0.000000 |

Fe1-C11: 1.794506 Fe1-C12: 1.771186

Table S4B: Distance table for the lowest-lying $B_8H_8Fe(CO)_3$ optimized structures obtained at the PBE0/def2-TZVP level of theory. Included are the zero-point corrected absolute energy in (a.u.) at the DLPNO-CCSD(T)/def2-QZVP level of theory with zero-point energy obtained from the PBE0/def2-TZVP computations, relative energies in (kcal/mol) and symmetry. For clarity, only the atoms forming the cluster framework are shown.

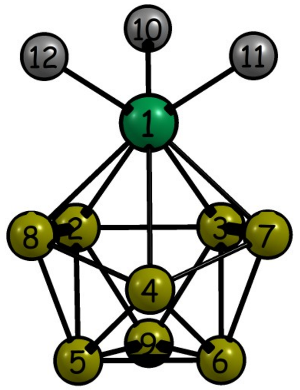


1. -1805.43234096 0.0 $C_s$

| | | 1 | 2 | 3 | 4 | 5 |
|---|---|---|---|---|---|---|
| 1 | Fe | 0.000000 | | | | |
| 2 | B | 2.149673 | 0.000000 | | | |
| 3 | B | 2.149104 | 1.812536 | 0.000000 | | |
| 4 | B | 2.282983 | 2.711342 | 2.711748 | 0.000000 | |
| 5 | B | 3.090261 | 2.010475 | 2.694971 | 1.795844 | 0.000000 |
| 6 | B | 3.089932 | 2.694397 | 2.012126 | 1.794621 | 1.774305 |
| 7 | B | 2.031518 | 2.907766 | 1.776479 | 1.775718 | 2.866064 |
| 8 | B | 2.031551 | 1.776858 | 2.908035 | 1.775137 | 1.739042 |
| 9 | B | 3.279676 | 1.740726 | 1.741134 | 2.864198 | 1.750448 |

| | | 6 | 7 | 8 | 9 |
|---|---|---|---|---|---|
| 6 | B | 0.000000 | | | |
| 7 | B | 1.738900 | 0.000000 | | |
| 8 | B | 2.865106 | 2.923712 | 0.000000 | |
| 9 | B | 1.750786 | 2.841088 | 2.841766 | 0.000000 |

Fe1-C10: 1.792380 Fe1-C11: 1.790077 Fe1-C12: 1.790189

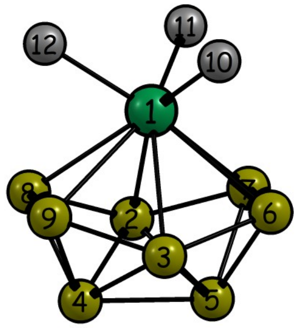


2. -1805.42244408 +6.2 $C_s$

| | | 1 | 2 | 3 | 4 | 5 |
|---|---|---|---|---|---|---|
| 1 | Fe | 0.000000 | | | | |
| 2 | B | 2.211047 | 0.000000 | | | |
| 3 | B | 2.210743 | 2.651370 | 0.000000 | | |
| 4 | B | 2.816578 | 1.846586 | 1.846902 | 0.000000 | |
| 5 | B | 2.803671 | 1.839070 | 1.839337 | 1.852812 | 0.000000 |
| 6 | B | 2.178173 | 2.728767 | 1.697536 | 2.973482 | 1.746306 |
| 7 | B | 2.177651 | 1.697551 | 2.728444 | 2.973312 | 1.746361 |
| 8 | B | 2.204044 | 1.662739 | 2.744607 | 1.740566 | 2.946921 |
| 9 | B | 2.203006 | 2.743670 | 1.663284 | 1.740577 | 2.946936 |

| | | 6 | 7 | 8 | 9 |
|---|---|---|---|---|---|
| 6 | B | 0.000000 | | | |
| 7 | B | 1.721237 | 0.000000 | | |
| 8 | B | 3.555228 | 3.088986 | 0.000000 | |
| 9 | B | 3.089301 | 3.554198 | 1.797067 | 0.000000 |

Fe1-C10: 1.783360 Fe1-C11: 1.783385 Fe1-C12: 1.779181

Table S4C: Distance table for the lowest-lying $B_8H_8Fe(CO)_4$ optimized structures obtained at the PBE0/def2-TZVP level of theory. Included are the zero-point corrected absolute energy in (a.u.) at the DLPNO-CCSD(T)/def2-QZVP level of theory with zero-point energy obtained from the PBE0/def2-TZVP computations, relative energies in (kcal/mol) and symmetry. For clarity, only the atoms forming the cluster framework are shown.

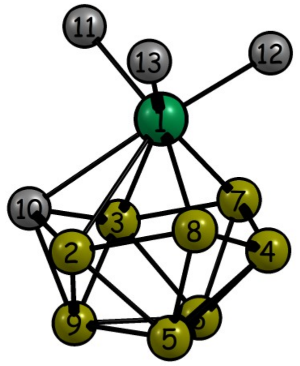

1. -1918.57730388 0.0 $C_1$

| | 1 | 2 | 3 | 4 | 5 |
|---|---|---|---|---|---|
| 1 Fe | 0.000000 | | | | |
| 2 B | 2.343723 | 0.000000 | | | |
| 3 B | 2.286649 | 2.762374 | 0.000000 | | |
| 4 B | 2.286354 | 2.770143 | 2.756386 | 0.000000 | |
| 5 B | 2.994446 | 1.789531 | 2.854840 | 1.827283 | 0.000000 |
| 6 B | 2.981247 | 2.870086 | 1.802888 | 1.819146 | 1.798286 |
| 7 B | 2.119674 | 3.381626 | 1.701017 | 1.753963 | 2.911627 |
| 8 B | 2.124446 | 1.744463 | 3.337731 | 1.699804 | 1.729499 |
| 9 B | 3.068028 | 1.859900 | 1.847985 | 2.896780 | 1.777159 |

| | 6 | 7 | 8 | 9 |
|---|---|---|---|---|
| 6 B | 0.000000 | | | |
| 7 B | 1.721087 | 0.000000 | | |
| 8 B | 2.882267 | 3.012288 | 0.000000 | |
| 9 B | 1.780798 | 2.920382 | 2.935382 | 0.000000 |

Fe1-C10: 2.210700 Fe1-C11: 1.809707
Fe1-C12: 1.780564 Fe1-C13: 1.792742

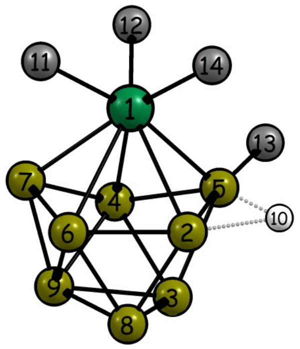

2. -1918.57398521 +2.1 $C_1$

| | 1 | 2 | 3 | 4 | 5 |
|---|---|---|---|---|---|
| 1 Fe | 0.000000 | | | | |
| 2 B | 2.223951 | 0.000000 | | | |
| 3 B | 3.149953 | 2.045578 | 0.000000 | | |
| 4 B | 2.206190 | 2.702788 | 1.849325 | 0.000000 | |
| 5 B | 2.093928 | 1.951986 | 1.842519 | 1.766760 | 0.000000 |
| 6 B | 2.299213 | 1.819562 | 2.663164 | 2.666540 | 3.061157 |
| 7 B | 2.051999 | 2.941911 | 2.929992 | 1.768339 | 3.028149 |
| 8 B | 3.343772 | 1.706725 | 1.667919 | 2.836292 | 2.918081 |
| 9 B | 3.079848 | 2.674385 | 1.862349 | 1.859996 | 2.994184 |
| 10 H | 2.868037 | 1.799487 | 2.161395 | 2.885000 | 1.234763 |

| | 6 | 7 | 8 | 9 | 10 |
|---|---|---|---|---|---|
| 6 B | 0.000000 | | | | |
| 7 B | 1.797924 | 0.000000 | | | |
| 8 B | 1.722441 | 2.905921 | 0.000000 | | |
| 9 B | 1.826555 | 1.700837 | 1.742166 | 0.000000 | |
| 10 H | 3.460196 | 3.968420 | 2.949608 | 3.659415 | 0.000000 |

Fe1-C11: 1.768545 Fe1-C12: 1.772771
Fe1-C13: 3.002788 Fe1-C14: 1.784871

Table S4D: Energy ranking for the $B_5H_5Fe(CO)_2$ structures at the PBE0/def2-TZVP level of theory.

| No. | Initial structure | Final Energy (a.u.) | Relative Energy (kcal/mol) |
|---|---|---|---|
| 1 | 09v-07-Isocloso-6vx—fe1—2co-pbe0 | -1693.1381250 | 0.00 |
| 2 | 09v-07-Isocloso-6vx—fe3—2co-r-89-pbe0 | -1693.1380850 | 0.03 |
| 3 | 09v-01-TricTrPrism—fe2—2co-pbe0 | -1693.1379980 | 0.08 |
| 4 | 09v-06-BicapPentBipyr—fe3—2co-pbe0 | -1693.1379770 | 0.09 |
| 5 | 09v-03-CapSqAntPr—fe3—2co-i-94-pbe0 | -1693.1379670 | 0.10 |
| 6 | 09v-03-CapSqAntPr—fe2—2co-pbe0 | -1693.1379650 | 0.10 |
| 7 | 09v-03-CapSqAntPr—fe3—2co-r-94-pbe0 | -1693.1379490 | 0.11 |
| 8 | 09v-07-Isocloso-6vx—fe3—2co-i-89-pbe0 | -1693.1379410 | 0.12 |
| 9 | 09v-03-CapSqAntPr—fe1—2co-pbe0 | -1693.1311100 | 4.40 |
| 10 | 09v-02-CapCube—fe1—2co-pbe0 | -1693.1299860 | 5.11 |
| 11 | 09v-06-BicapPentBipyr—fe4—2co-pbe0 | -1693.1299630 | 5.12 |
| 12 | 09v-04-Tl99—fe1—2co-pbe0 | -1693.1224350 | 9.85 |
| 13 | 09v-02-CapCube—fe4—2co-pbe0 | -1693.1203120 | 11.18 |
| 14 | 09v-04-Tl99—fe2—2co-pbe0 | -1693.1203070 | 11.18 |
| 15 | 09v-01-TricTrPrism—fe1—2co-pbe0 | -1693.1096550 | 17.87 |
| 16 | 09v-07-Isocloso-6vx—fe2—2co-pbe0 | -1693.1055290 | 20.45 |
| 17 | 09v-02-CapCube—fe5—2co-pbe0 | -1693.1000460 | 23.89 |
| 18 | 09v-04-Tl99—fe3—2co-pbe0 | -1693.0990620 | 24.51 |
| 19 | 09v-02-CapCube—fe3—2co-pbe0 | -1693.0963370 | 26.22 |
| 20 | 09v-06-BicapPentBipyr—fe5—2co-pbe0 | -1693.0895300 | 30.49 |
| 21 | 09v-06-BicapPentBipyr—fe2—2co-pbe0 | -1693.0894430 | 30.55 |
| 22 | 09v-06-BicapPentBipyr—fe1—2co-pbe0 | -1693.0830260 | 34.58 |
| 23 | 09v-02-CapCube—fe2—2co-pbe0 | -1693.0756230 | 39.22 |

Table S4E: Energy ranking for the $B_5H_5Fe(CO)_2$ structures at the PBE0/def2-TZVP level of theory.

| No. | Initial structure | Final Energy (a.u.) | Relative Energy (kcal/mol) |
|---|---|---|---|
| 1 | 09v-01-TricTrPrism—fe2—3co-pbe0 | -1806.3684440 | 0.00 |
| 2 | 09v-01-TricTrPrism—fe1—3co-pbe0 | -1806.3684180 | 0.02 |
| 3 | 09v-03-CapSqAntPr—fe1—3co-pbe0 | -1806.3683890 | 0.03 |
| 4 | 09v-03-CapSqAntPr—fe3—3co-pbe0 | -1806.3683870 | 0.04 |
| 5 | 09v-02-CapCube—fe1—3co-pbe0 | -1806.3683710 | 0.05 |
| 6 | 09v-03-CapSqAntPr—fe2—3co-pbe0 | -1806.3683610 | 0.05 |
| 7 | 09v-06-BicapPentBipyr—fe3—3co-pbe0 | -1806.3683330 | 0.07 |
| 8 | 09v-04-Tl99—fe1—3co-pbe0 | -1806.3630680 | 3.37 |
| 9 | 09v-07-Isocloso-6vx—fe2—3co-pbe0 | -1806.3630680 | 3.37 |
| 10 | 09v-02-CapCube—fe4—3co-pbe0 | -1806.3513790 | 10.71 |
| 11 | 09v-02-CapCube—fe2—3co-pbe0 | -1806.3469170 | 13.51 |
| 12 | 09v-02-CapCube—fe3—3co-pbe0 | -1806.3434670 | 15.67 |
| 13 | 09v-06-BicapPentBipyr—fe5—3co-pbe0 | -1806.3434500 | 15.68 |
| 14 | 09v-04-Tl99—fe3—3co-pbe0 | -1806.3316240 | 23.10 |
| 15 | 09v-04-Tl99—fe2—3co-pbe0 | -1806.3296290 | 24.36 |
| 16 | 09v-06-BicapPentBipyr—fe1—3co-pbe0 | -1806.3237970 | 28.02 |
| 17 | 09v-07-Isocloso-6vx—fe3—3co-pbe0 | -1806.3208140 | 29.89 |
| 18 | 09v-07-Isocloso-6vx—fe1—3co-r-33-pbe0 | -1806.3208070 | 29.89 |
| 19 | 09v-06-BicapPentBipyr—fe2—3co-pbe0 | -1806.3191270 | 30.95 |
| 20 | 09v-07-Isocloso-6vx—fe1—3co-i-33-pbe0 | -1806.3182370 | 31.51 |
| 21 | 09v-06-BicapPentBipyr—fe4—3co-pbe0 | -1806.3153500 | 33.32 |
| 22 | 09v-02-CapCube—fe5—3co-pbe0 | -1806.2905460 | 48.88 |

Table S4F: Energy ranking for the $B_5H_5Fe(CO)_2$ structures at the PBE0/def2-TZVP level of theory.

| No. | Initial structure | Final Energy (a.u.) | Relative Energy (kcal/mol) |
|---|---|---|---|
| 1 | 09v-01-TricTrPrism—fe2—4co-pbe0 | -1919.5700930 | 0.00 |
| 2 | 09v-07-Isocloso-6vx—fe2—4co-pbe0 | -1919.5700930 | 0.00 |
| 3 | 09v-07-Isocloso-6vx—fe1—4co-pbe0 | -1919.5480190 | 13.85 |
| 4 | 09v-02-CapCube—fe2—4co-pbe0 | -1919.5479230 | 13.91 |
| 5 | 09v-04-Tl99—fe2—4co-pbe0 | -1919.5463340 | 14.91 |
| 6 | 09v-04-Tl99—fe3—4co-pbe0 | -1919.5462880 | 14.94 |
| 7 | 09v-02-CapCube—fe1—4co-pbe0 | -1919.5430910 | 16.94 |
| 8 | 09v-03-CapSqAntPr—fe1—4co-pbe0 | -1919.5430690 | 16.96 |
| 9 | 09v-01-TricTrPrism—fe1—4co-pbe0 | -1919.5430370 | 16.98 |
| 10 | 09v-07-Isocloso-6vx—fe3—4co-r-42-pbe0 | -1919.5396810 | 19.08 |
| 11 | 09v-03-CapSqAntPr—fe3—4co-pbe0 | -1919.5391550 | 19.41 |
| 12 | 09v-03-CapSqAntPr—fe2—4co-pbe0 | -1919.5391060 | 19.44 |
| 13 | 09v-02-CapCube—fe4—4co-pbe0 | -1919.5351070 | 21.95 |
| 14 | 09v-04-Tl99—fe1—4co-pbe0 | -1919.5335670 | 22.92 |
| 15 | 09v-07-Isocloso-6vx—fe3—4co-i-42-pbe0 | -1919.5331110 | 23.21 |
| 16 | 09v-06-BicapPentBipyr—fe4—4co-pbe0 | -1919.5243680 | 28.69 |
| 17 | 09v-06-BicapPentBipyr—fe3—4co-pbe0 | -1919.5108640 | 37.17 |
| 18 | 09v-02-CapCube—fe3—4co-pbe0 | -1919.4944340 | 47.48 |
| 19 | 09v-06-BicapPentBipyr—fe1—4co-pbe0 | -1919.4921160 | 48.93 |
| 20 | 09v-06-BicapPentBipyr—fe2—4co-pbe0 | -1919.4895600 | 50.54 |
| 21 | 09v-06-BicapPentBipyr—fe5—4co-pbe0 | -1919.4881220 | 51.44 |
| 22 | 09v-02-CapCube—fe5—4co-pbe0 | -1919.4826810 | 54.85 |

Table S5A: Distance table for the lowest-lying $B_9H_9Fe(CO)_2$ optimized structures obtained at the PBE0/def2-TZVP level of theory. Included are the zero-point corrected absolute energy in (a.u.) at the DLPNO-CCSD(T)/def2-QZVP level of theory with zero-point energy obtained from the PBE0/def2-TZVP computations, relative energies in (kcal/mol) and symmetry. For clarity, only the atoms forming the cluster framework are shown.

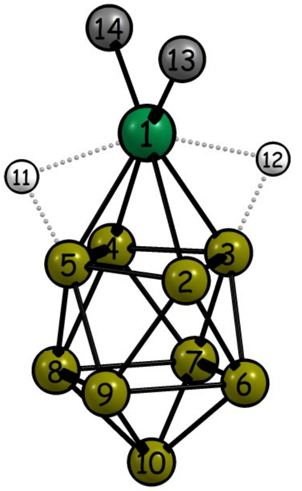


1. -1717.63614029 0.0 $C_{2v}$

| | | 1 | 2 | 3 | 4 | 5 |
|---|---|---|---|---|---|---|
| 1 | Fe | 0.000000 | | | | |
| 2 | B | 2.195208 | 0.000000 | | | |
| 3 | B | 1.931014 | 1.775271 | 0.000000 | | |
| 4 | B | 2.194781 | 2.827522 | 1.775989 | 0.000000 | |
| 5 | B | 1.930491 | 1.774436 | 2.142158 | 1.774962 | 0.000000 |
| 6 | B | 3.395920 | 1.781948 | 1.795346 | 2.922148 | 2.685941 |
| 7 | B | 3.395594 | 2.922629 | 1.795940 | 1.781377 | 2.686219 |
| 8 | B | 3.395033 | 2.920997 | 2.686381 | 1.781505 | 1.794761 |
| 9 | B | 3.394921 | 1.781186 | 2.685389 | 2.920507 | 1.794305 |
| 10 | B | 4.219224 | 2.907551 | 2.823710 | 2.906604 | 2.823924 |
| 11 | H | 1.744496 | 2.522480 | 2.992790 | 2.523477 | 1.314615 |
| 12 | H | 1.744969 | 2.523766 | 1.314337 | 2.523561 | 2.992331 |

| | | 6 | 7 | 8 | 9 | 10 |
|---|---|---|---|---|---|---|
| 6 | B | 0.000000 | | | | |
| 7 | B | 1.897786 | 0.000000 | | | |
| 8 | B | 2.658843 | 1.863974 | 0.000000 | | |
| 9 | B | 1.863260 | 2.658677 | 1.894805 | 0.000000 | |
| 10 | B | 1.722394 | 1.722025 | 1.722019 | 1.722516 | 0.000000 |
| 11 | H | 3.864631 | 3.864972 | 2.940441 | 2.939959 | 4.134175 |
| 12 | H | 2.941064 | 2.940868 | 3.864565 | 3.864065 | 4.133692 |

| | | 11 | 12 |
|---|---|---|---|
| 11 | H | 0.000000 | |
| 12 | H | 3.373968 | 0.000000 |

Fe1-C13: 1.799463 Fe1-C14: 1.799253

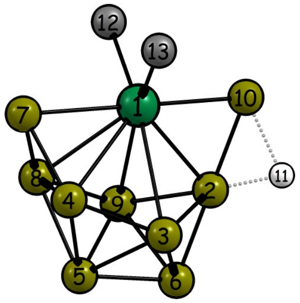


2. -1717.62545670 +6.7 $C_s$

| | | 1 | 2 | 3 | 4 | 5 |
|---|---|---|---|---|---|---|
| 1 | Fe | 0.000000 | | | | |
| 2 | B | 1.982867 | 0.000000 | | | |
| 3 | B | 2.332200 | 1.814687 | 0.000000 | | |
| 4 | B | 2.146963 | 2.851308 | 1.697111 | 0.000000 | |
| 5 | B | 3.021108 | 2.643303 | 1.901865 | 1.754481 | 0.000000 |
| 6 | B | 3.105771 | 1.621062 | 1.791629 | 2.835309 | 1.706140 |
| 7 | B | 2.054940 | 3.659408 | 3.100537 | 1.633648 | 3.036257 |
| 8 | B | 2.180755 | 2.917352 | 2.739531 | 1.686201 | 1.828316 |
| 9 | B | 2.334349 | 1.823393 | 2.565976 | 2.646288 | 1.891735 |
| 10 | B | 1.889590 | 1.663247 | 2.981662 | 3.709630 | 4.082688 |
| 11 | H | 2.864013 | 1.265644 | 2.846291 | 4.066091 | 3.844476 |

| | | 6 | 7 | 8 | 9 | 10 |
|---|---|---|---|---|---|---|
| 6 | B | 0.000000 | | | | |
| 7 | B | 4.086264 | 0.000000 | | | |
| 8 | B | 2.910718 | 1.746172 | 0.000000 | | |
| 9 | B | 1.790598 | 3.197170 | 1.725952 | 0.000000 | |
| 10 | B | 3.284273 | 3.942812 | 3.748809 | 2.983807 | 0.000000 |
| 11 | H | 2.496111 | 4.775584 | 4.121406 | 2.848849 | 1.513447 |

| | | 11 |
|---|---|---|
| 11 | H | 0.000000 |

Fe1-C12: 1.759363 Fe1-C13: 1.755247

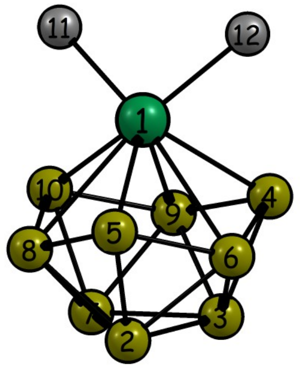


3. -1717.61921591 +10.6 $C_s$

| | | 1 | 2 | 3 | 4 | 5 |
|---|---|---|---|---|---|---|
| 1 | Fe | 0.000000 | | | | |
| 2 | B | 2.971074 | 0.000000 | | | |
| 3 | B | 2.956703 | 1.801104 | 0.000000 | | |
| 4 | B | 2.101637 | 2.909793 | 1.738893 | 0.000000 | |
| 5 | B | 2.020381 | 1.728830 | 2.845300 | 2.929769 | 0.000000 |
| 6 | B | 2.289562 | 1.815222 | 1.801653 | 1.717285 | 1.699951 |
| 7 | B | 2.957523 | 1.800733 | 1.786230 | 2.903287 | 2.846168 |
| 8 | B | 2.287860 | 1.815459 | 2.863852 | 3.366940 | 1.701023 |
| 9 | B | 2.269197 | 2.880090 | 1.808730 | 1.736647 | 3.274427 |
| 10 | B | 2.105027 | 2.909301 | 2.904197 | 3.027905 | 2.931403 |

| | | 6 | 7 | 8 | 9 | 10 |
|---|---|---|---|---|---|---|
| 6 | B | 0.000000 | | | | |
| 7 | B | 2.864624 | 0.000000 | | | |
| 8 | B | 2.775411 | 1.801867 | 0.000000 | | |
| 9 | B | 2.758060 | 1.809222 | 2.756511 | 0.000000 | |
| 10 | B | 3.370307 | 1.738568 | 1.714306 | 1.739217 | 0.000000 |

Fe1-C11: 1.807251 Fe1-C12: 1.806800

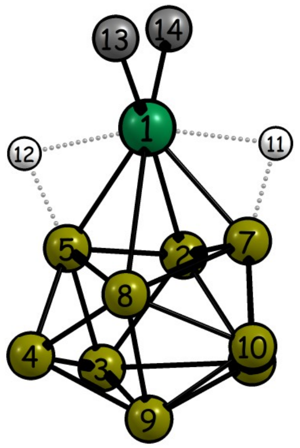


4. -1717.60235981 +21.2 $C_s$

| | | 1 | 2 | 3 | 4 | 5 |
|---|---|---|---|---|---|---|
| 1 | Fe | 0.000000 | | | | |
| 2 | B | 2.237111 | 0.000000 | | | |
| 3 | B | 3.341714 | 1.793704 | 0.000000 | | |
| 4 | B | 3.268176 | 2.911512 | 1.752924 | 0.000000 | |
| 5 | B | 1.928534 | 1.738511 | 1.712928 | 1.670257 | 0.000000 |
| 6 | B | 3.339898 | 1.793054 | 1.985973 | 2.981640 | 2.766771 |
| 7 | B | 1.927413 | 1.736035 | 2.769415 | 3.129576 | 2.381714 |
| 8 | B | 2.498854 | 2.904144 | 2.696862 | 1.760795 | 2.089114 |
| 9 | B | 3.731982 | 2.871733 | 1.891183 | 1.715493 | 2.614990 |
| 10 | B | 3.261189 | 2.909817 | 2.983021 | 2.929700 | 3.121382 |
| 11 | H | 1.682323 | 2.586502 | 3.937537 | 4.122548 | 3.107812 |
| 12 | H | 1.681417 | 2.589303 | 2.985668 | 2.690362 | 1.325028 |

| | | 6 | 7 | 8 | 9 | 10 |
|---|---|---|---|---|---|---|
| 6 | B | 0.000000 | | | | |
| 7 | B | 1.711887 | 0.000000 | | | |
| 8 | B | 2.698446 | 2.099838 | 0.000000 | | |
| 9 | B | 1.889251 | 2.618560 | 1.815611 | 0.000000 | |
| 10 | B | 1.754780 | 1.670983 | 1.762068 | 1.715601 | 0.000000 |
| 11 | H | 2.984013 | 1.324110 | 2.866519 | 3.857215 | 2.689740 |
| 12 | H | 3.935516 | 3.106386 | 2.854182 | 3.853930 | 4.112350 |

| | | 11 | 12 |
|---|---|---|---|
| 11 | H | 0.000000 | |
| 12 | H | 3.316765 | 0.000000 |

Fe1-C13: 1.800859 Fe1-C14: 1.783767

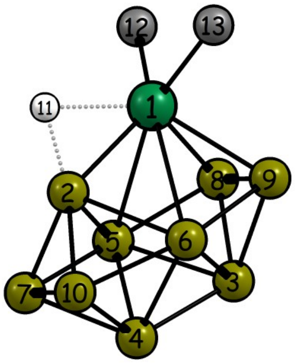


5. -1717.59572376 +25.4 $C_s$

| | | 1 | 2 | 3 | 4 | 5 |
|---|---|---|---|---|---|---|
| 1 | Fe | 0.000000 | | | | |
| 2 | B | 1.850519 | 0.000000 | | | |
| 3 | B | 2.866489 | 2.962966 | 0.000000 | | |
| 4 | B | 3.385233 | 2.384941 | 1.772525 | 0.000000 | |
| 5 | B | 2.314225 | 1.932365 | 1.923057 | 1.891075 | 0.000000 |
| 6 | B | 2.309593 | 1.929783 | 1.922947 | 1.890695 | 2.462172 |
| 7 | B | 3.290412 | 1.711419 | 3.084846 | 1.758418 | 1.820799 |
| 8 | B | 2.017555 | 2.973953 | 1.736852 | 3.025558 | 1.804079 |
| 9 | B | 2.018659 | 2.974767 | 1.736849 | 3.024699 | 2.750700 |
| 10 | B | 3.289261 | 1.710635 | 3.085276 | 1.758336 | 2.735345 |
| 11 | H | 1.730638 | 1.287935 | 3.951442 | 3.658692 | 2.846716 |
| | | 6 | 7 | 8 | 9 | 10 |
| 6 | B | 0.000000 | | | | |
| 7 | B | 2.734367 | 0.000000 | | | |
| 8 | B | 2.746896 | 3.543521 | 0.000000 | | |
| 9 | B | 1.801468 | 3.938797 | 1.748825 | 0.000000 | |
| 10 | B | 1.821224 | 1.690926 | 3.938156 | 3.542934 | 0.000000 |
| 11 | H | 2.841993 | 2.822329 | 3.482980 | 3.483264 | 2.820177 |
| | | 11 | | | | |
| 11 | H | 0.000000 | | | | |

Fe1-C12: 1.774385 Fe1-C13: 1.774218

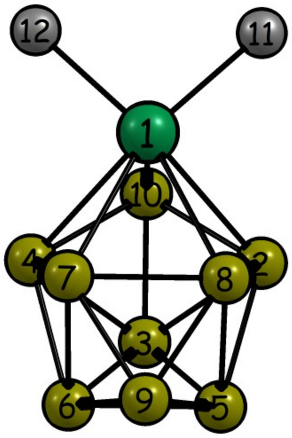


6. -1717.58282426 +33.5 $C_s$

| | | 1 | 2 | 3 | 4 | 5 |
|---|---|---|---|---|---|---|
| 1 | Fe | 0.000000 | | | | |
| 2 | B | 2.168371 | 0.000000 | | | |
| 3 | B | 2.965534 | 1.853925 | 0.000000 | | |
| 4 | B | 2.166499 | 2.675198 | 1.855063 | 0.000000 | |
| 5 | B | 3.312138 | 1.797768 | 1.775144 | 2.820509 | 0.000000 |
| 6 | B | 3.310671 | 2.819859 | 1.775130 | 1.797026 | 1.765402 |
| 7 | B | 2.099055 | 2.813691 | 2.816975 | 1.735956 | 2.628941 |
| 8 | B | 2.099142 | 1.734939 | 2.815338 | 2.812415 | 1.916794 |
| 9 | B | 3.313397 | 2.883991 | 2.950230 | 2.883406 | 1.762900 |
| 10 | B | 1.937030 | 1.842245 | 1.729609 | 1.842786 | 2.998461 |
| | | 6 | 7 | 8 | 9 | 10 |
| 6 | B | 0.000000 | | | | |
| 7 | B | 1.916894 | 0.000000 | | | |
| 8 | B | 2.627998 | 1.832197 | 0.000000 | | |
| 9 | B | 1.762617 | 1.714952 | 1.715955 | 0.000000 | |
| 10 | B | 2.997789 | 2.980841 | 2.979173 | 3.821869 | 0.000000 |

Fe1-C11: 1.813156 Fe1-C12: 1.813485

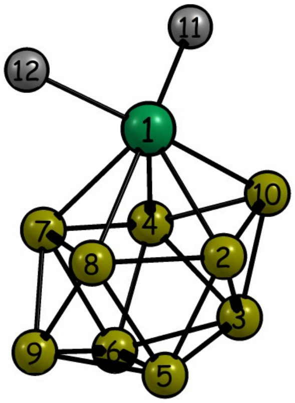


7. -1717.57470028 +38.6 $C_1$

| | | 1 | 2 | 3 | 4 | 5 |
|---|---|---|---|---|---|---|
| 1 | Fe | 0.000000 | | | | |
| 2 | B | 2.035766 | 0.000000 | | | |
| 3 | B | 3.008358 | 1.882506 | 0.000000 | | |
| 4 | B | 2.214054 | 2.590856 | 1.850629 | 0.000000 | |
| 5 | B | 3.276388 | 1.809799 | 1.767069 | 2.791357 | 0.000000 |
| 6 | B | 3.331300 | 2.789167 | 1.749386 | 1.782899 | 1.759705 |
| 7 | B | 2.091991 | 2.758359 | 2.873386 | 1.754325 | 2.666565 |
| 8 | B | 2.108696 | 1.771240 | 2.887847 | 2.831691 | 1.925088 |
| 9 | B | 3.326067 | 2.867536 | 2.961985 | 2.896548 | 1.756295 |
| 10 | B | 2.015302 | 1.774792 | 1.703476 | 1.843113 | 2.948024 |
| | | 6 | 7 | 8 | 9 | 10 |
| 6 | B | 0.000000 | | | | |
| 7 | B | 1.987687 | 0.000000 | | | |
| 8 | B | 2.635347 | 1.821448 | 0.000000 | | |
| 9 | B | 1.786695 | 1.787584 | 1.674089 | 0.000000 | |
| 10 | B | 2.964377 | 3.013532 | 3.060829 | 3.832324 | 0.000000 |

Fe1-C11: 1.799710 Fe1-C12: 1.839486

Table S5B: Distance table for the lowest-lying $B_9H_9Fe(CO)_3$ optimized structures obtained at the PBE0/def2-TZVP level of theory. Included are the zero-point corrected absolute energy in (a.u.) at the DLPNO-CCSD(T)/def2-QZVP level of theory with zero-point energy obtained from the PBE0/def2-TZVP computations, relative energies in (kcal/mol) and symmetry. For clarity, only the atoms forming the cluster framework are shown.

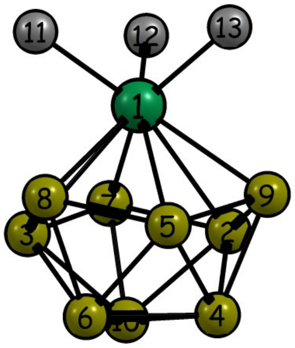


1. -1830.83637759 0.0 $C_1$

| | 1 | 2 | 3 | 4 | 5 |
|---|---|---|---|---|---|
| 1 Fe | 0.000000 | | | | |
| 2 B | 2.291428 | 0.000000 | | | |
| 3 B | 2.292965 | 2.742092 | 0.000000 | | |
| 4 B | 2.998222 | 1.813041 | 2.861114 | 0.000000 | |
| 5 B | 2.290668 | 2.741198 | 2.742080 | 1.808000 | 0.000000 |
| 6 B | 2.998596 | 2.859601 | 1.808173 | 1.788188 | 1.813556 |
| 7 B | 2.142410 | 1.743630 | 1.707763 | 2.913545 | 3.359645 |
| 8 B | 2.144838 | 3.360611 | 1.743766 | 2.895153 | 1.707394 |
| 9 B | 2.142776 | 1.706879 | 3.361624 | 1.735366 | 1.744426 |
| 10 B | 2.998212 | 1.806723 | 1.813627 | 1.788996 | 2.860626 |

| | 6 | 7 | 8 | 9 | 10 |
|---|---|---|---|---|---|
| 6 B | 0.000000 | | | | |
| 7 B | 2.895325 | 0.000000 | | | |
| 8 B | 1.734477 | 3.032770 | 0.000000 | | |
| 9 B | 2.913558 | 3.032321 | 3.034465 | 0.000000 | |
| 10 B | 1.788876 | 1.735245 | 2.913015 | 2.895135 | 0.000000 |

Fe1-C11: 1.788974 Fe1-C12: 1.789318 Fe1-C13: 1.789149

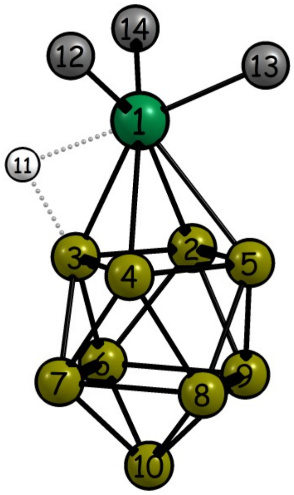


2. -1830.82791036 +5.3 $C_s$

| | 1 | 2 | 3 | 4 | 5 |
|---|---|---|---|---|---|
| 1 Fe | 0.000000 | | | | |
| 2 B | 2.262300 | 0.000000 | | | |
| 3 B | 1.930672 | 1.776300 | 0.000000 | | |
| 4 B | 2.264193 | 2.782194 | 1.776682 | 0.000000 | |
| 5 B | 2.243437 | 1.806976 | 2.257002 | 1.806326 | 0.000000 |
| 6 B | 3.412541 | 1.812093 | 1.733094 | 2.930177 | 2.792273 |
| 7 B | 3.413280 | 2.930052 | 1.732619 | 1.813002 | 2.792929 |
| 8 B | 3.571743 | 2.895081 | 2.629008 | 1.768027 | 1.832513 |
| 9 B | 3.570382 | 1.768528 | 2.628671 | 2.893767 | 1.831552 |
| 10 B | 4.329462 | 2.920637 | 2.755265 | 2.920584 | 2.904727 |
| 11 H | 1.636467 | 2.545610 | 1.325705 | 2.546207 | 3.151482 |

| | 6 | 7 | 8 | 9 | 10 |
|---|---|---|---|---|---|
| 6 B | 0.000000 | | | | |
| 7 B | 1.905059 | 0.000000 | | | |
| 8 B | 2.650407 | 1.852325 | 0.000000 | | |
| 9 B | 1.851507 | 2.650554 | 1.887318 | 0.000000 | |
| 10 B | 1.726109 | 1.726752 | 1.719775 | 1.719706 | 0.000000 |
| 11 H | 2.852972 | 2.852336 | 3.843014 | 3.842678 | 4.064469 |

| | 11 |
|---|---|
| 11 H | 0.000000 |

Fe1-C12: 1.789391 Fe1-C13: 1.790909 Fe1-C14: 1.789188

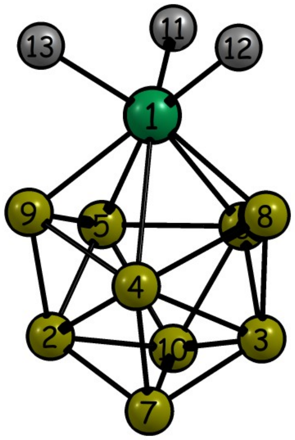


3. -1830.81095954 +16.0 $C_s$

| | | 1 | 2 | 3 | 4 | 5 |
|---|---|---|---|---|---|---|
| 1 | Fe | 0.000000 | | | | |
| 2 | B | 3.137549 | 0.000000 | | | |
| 3 | B | 3.137799 | 2.710737 | 0.000000 | | |
| 4 | B | 2.354399 | 2.029760 | 2.030657 | 0.000000 | |
| 5 | B | 2.183085 | 1.793876 | 2.851133 | 2.655302 | 0.000000 |
| 6 | B | 2.183905 | 2.851815 | 1.793136 | 2.655841 | 1.812881 |
| 7 | B | 3.726070 | 1.689263 | 1.689085 | 1.847616 | 2.876390 |
| 8 | B | 1.976039 | 3.334485 | 1.701613 | 1.904564 | 2.947931 |
| 9 | B | 1.975610 | 1.702087 | 3.336388 | 1.907257 | 1.784884 |
| 10 | B | 3.333307 | 1.789718 | 1.789098 | 2.645628 | 1.803811 |
| | | 6 | 7 | 8 | 9 | 10 |
| 6 | B | 0.000000 | | | | |
| 7 | B | 2.876272 | 0.000000 | | | |
| 8 | B | 1.786673 | 2.923420 | 0.000000 | | |
| 9 | B | 2.948602 | 2.925794 | 3.035651 | 0.000000 | |
| 10 | B | 1.803702 | 1.744986 | 2.916837 | 2.917194 | 0.000000 |

Fe1-C11: 1.780983 Fe1-C12: 1.782508 Fe1-C13: 1.782370

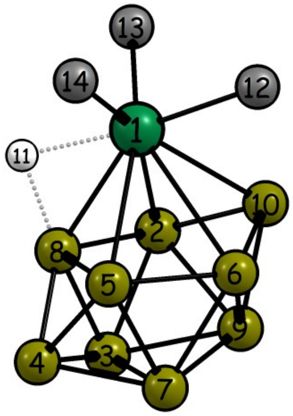


4. -1830.80278255 +21.1 $C_1$

| | | 1 | 2 | 3 | 4 | 5 |
|---|---|---|---|---|---|---|
| 1 | Fe | 0.000000 | | | | |
| 2 | B | 2.222913 | 0.000000 | | | |
| 3 | B | 3.310089 | 1.819962 | 0.000000 | | |
| 4 | B | 3.349142 | 3.008242 | 1.752167 | 0.000000 | |
| 5 | B | 2.368215 | 3.043193 | 2.675987 | 1.731139 | 0.000000 |
| 6 | B | 2.386906 | 2.656447 | 2.837936 | 2.917517 | 1.754257 |
| 7 | B | 3.447690 | 2.867385 | 1.810947 | 1.726570 | 1.814808 |
| 8 | B | 1.993319 | 1.814975 | 1.740626 | 1.633117 | 1.860098 |
| 9 | B | 3.148322 | 1.844242 | 1.828195 | 2.959608 | 2.849327 |
| 10 | B | 2.164751 | 1.700128 | 2.937672 | 3.815659 | 3.099074 |
| 11 | H | 1.629127 | 2.426143 | 3.014915 | 2.799765 | 2.600714 |
| | | 6 | 7 | 8 | 9 | 10 |
| 6 | B | 0.000000 | | | | |
| 7 | B | 1.831452 | 0.000000 | | | |
| 8 | B | 2.643354 | 2.426116 | 0.000000 | | |
| 9 | B | 1.827020 | 1.785508 | 2.678432 | 0.000000 | |
| 10 | B | 1.834084 | 2.996448 | 2.846616 | 1.692820 | 0.000000 |
| 11 | H | 3.404287 | 3.675589 | 1.349102 | 3.737176 | 3.319237 |
| | | 11 | | | | |
| 11 | H | 0.000000 | | | | |

Fe1-C12: 1.790300 Fe1-C13: 1.790588 Fe1-C14: 1.808101

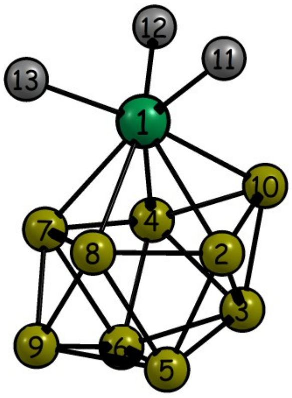


5. -1830.80230759 +21.4 $C_\mathrm{s}$

| | | 1 | 2 | 3 | 4 | 5 |
|---|---|---|---|---|---|---|
| 1 | Fe | 0.000000 | | | | |
| 2 | B | 2.182766 | 0.000000 | | | |
| 3 | B | 3.029168 | 1.871230 | 0.000000 | | |
| 4 | B | 2.182208 | 2.648315 | 1.872761 | 0.000000 | |
| 5 | B | 3.356390 | 1.798407 | 1.754760 | 2.795874 | 0.000000 |
| 6 | B | 3.355438 | 2.794647 | 1.754714 | 1.798087 | 1.729322 |
| 7 | B | 2.101892 | 2.807106 | 2.878495 | 1.745824 | 2.672112 |
| 8 | B | 2.100795 | 1.745419 | 2.877703 | 2.806435 | 1.996578 |
| 9 | B | 3.340456 | 2.874193 | 2.979371 | 2.874347 | 1.795154 |
| 10 | B | 2.022325 | 1.847762 | 1.705025 | 1.846839 | 2.972684 |

| | | 6 | 7 | 8 | 9 | 10 |
|---|---|---|---|---|---|---|
| 6 | B | 0.000000 | | | | |
| 7 | B | 1.995681 | 0.000000 | | | |
| 8 | B | 2.671660 | 1.824091 | 0.000000 | | |
| 9 | B | 1.795189 | 1.737006 | 1.737852 | 0.000000 | |
| 10 | B | 2.971337 | 3.022532 | 3.022001 | 3.837790 | 0.000000 |

Fe1-C11: 1.784988 Fe1-C12: 1.785029 Fe1-C13: 1.810639

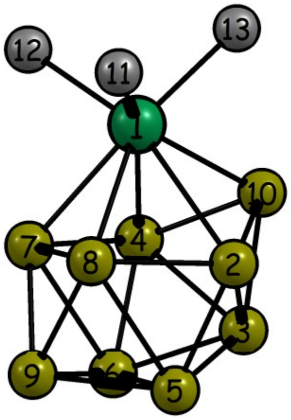


6. -1830.79698268 +24.7 $C_\mathrm{s}$

| | | 1 | 2 | 3 | 4 | 5 |
|---|---|---|---|---|---|---|
| 1 | Fe | 0.000000 | | | | |
| 2 | B | 2.213032 | 0.000000 | | | |
| 3 | B | 3.051962 | 1.862911 | 0.000000 | | |
| 4 | B | 2.208087 | 2.653064 | 1.863950 | 0.000000 | |
| 5 | B | 3.350803 | 1.766219 | 1.732171 | 2.783611 | 0.000000 |
| 6 | B | 3.346918 | 2.781567 | 1.732696 | 1.766228 | 1.742611 |
| 7 | B | 2.078314 | 2.814406 | 2.918504 | 1.814577 | 2.686752 |
| 8 | B | 2.076754 | 1.812628 | 2.918085 | 2.813155 | 2.046243 |
| 9 | B | 3.379011 | 2.887646 | 2.958429 | 2.888077 | 1.785219 |
| 10 | B | 1.984610 | 1.826165 | 1.733122 | 1.824653 | 2.942559 |

| | | 6 | 7 | 8 | 9 | 10 |
|---|---|---|---|---|---|---|
| 6 | B | 0.000000 | | | | |
| 7 | B | 2.041409 | 0.000000 | | | |
| 8 | B | 2.686205 | 1.744484 | 0.000000 | | |
| 9 | B | 1.783148 | 1.773347 | 1.777738 | 0.000000 | |
| 10 | B | 2.941271 | 3.003756 | 3.001231 | 3.819002 | 0.000000 |

Fe1-C11: 1.789601 Fe1-C12: 1.789368 Fe1-C13: 1.784026

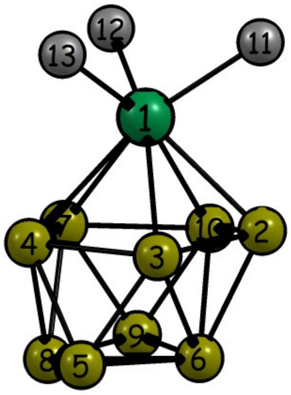


7. -1830.79625896 +25.2 $C_\mathrm{s}$

| | | 1 | 2 | 3 | 4 | 5 |
|---|---|---|---|---|---|---|
| 1 | Fe | 0.000000 | | | | |
| 2 | B | 2.043535 | 0.000000 | | | |
| 3 | B | 2.149676 | 1.728671 | 0.000000 | | |
| 4 | B | 2.175142 | 2.925221 | 1.837076 | 0.000000 | |
| 5 | B | 3.330494 | 2.912119 | 1.690043 | 1.749031 | 0.000000 |
| 6 | B | 3.099363 | 1.819922 | 2.026487 | 2.646058 | 1.876008 |
| 7 | B | 2.175751 | 2.925045 | 2.902547 | 1.812363 | 2.896999 |
| 8 | B | 3.341264 | 3.245781 | 2.713229 | 1.826170 | 1.678149 |
| 9 | B | 3.331371 | 2.911060 | 3.324347 | 2.897058 | 2.941968 |
| 10 | B | 2.152585 | 1.730067 | 2.788561 | 2.904273 | 3.326628 |

| | | 6 | 7 | 8 | 9 | 10 |
|---|---|---|---|---|---|---|
| 6 | B | 0.000000 | | | | |
| 7 | B | 2.646587 | 0.000000 | | | |
| 8 | B | 1.958042 | 1.826042 | 0.000000 | | |
| 9 | B | 1.875763 | 1.750033 | 1.677646 | 0.000000 | |
| 10 | B | 2.028505 | 1.837650 | 2.713768 | 1.689311 | 0.000000 |

Fe1-C11: 1.776129 Fe1-C12: 1.787895 Fe1-C13: 1.787637

Table S5C: Distance table for the lowest-lying $B_9H_9Fe(CO)_4$ optimized structures obtained at the PBE0/def2-TZVP level of theory. Included are the zero-point corrected absolute energy in (a.u.) at the DLPNO-CCSD(T)/def2-QZVP level of theory with zero-point energy obtained from the PBE0/def2-TZVP computations, relative energies in (kcal/mol) and symmetry. For clarity, only the atoms forming the cluster framework are shown.

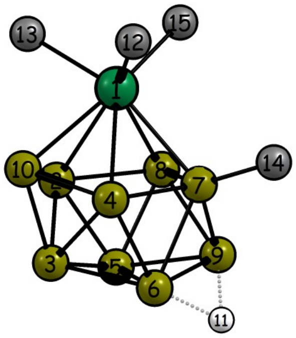


1. -1944.00306565 0.0 $C_1$

| | | 1 | 2 | 3 | 4 | 5 |
|---|---|---|---|---|---|---|
| 1 | Fe | 0.000000 | | | | |
| 2 | B | 2.262984 | 0.000000 | | | |
| 3 | B | 3.098420 | 1.810256 | 0.000000 | | |
| 4 | B | 2.170461 | 2.661831 | 1.949154 | 0.000000 | |
| 5 | B | 3.348251 | 1.763004 | 1.758270 | 2.919829 | 0.000000 |
| 6 | B | 3.374328 | 2.875101 | 1.808451 | 1.880234 | 1.909561 |
| 7 | B | 2.106313 | 2.823751 | 2.800715 | 1.754270 | 2.617268 |
| 8 | B | 2.190740 | 1.771176 | 2.782452 | 2.877084 | 1.793502 |
| 9 | B | 3.410528 | 2.963946 | 2.923199 | 2.996532 | 1.765929 |
| 10 | B | 2.072587 | 1.773109 | 1.698299 | 1.733292 | 2.927468 |
| 11 | H | 4.223780 | 3.731880 | 2.981480 | 3.071061 | 2.317086 |

| | | 6 | 7 | 8 | 9 | 10 |
|---|---|---|---|---|---|---|
| 6 | B | 0.000000 | | | | |
| 7 | B | 1.864623 | 0.000000 | | | |
| 8 | B | 2.652679 | 1.885621 | 0.000000 | | |
| 9 | B | 1.761005 | 1.769343 | 1.763265 | 0.000000 | |
| 10 | B | 2.937800 | 2.923227 | 2.976934 | 3.803208 | 0.000000 |
| 11 | H | 1.331157 | 2.331623 | 2.965896 | 1.334778 | 4.175008 |

| | | 11 |
|---|---|---|
| 11 | H | 0.000000 |

Fe1-C12: 1.768536 Fe1-C13: 1.763113
Fe1-C14: 3.016802 Fe1-C15: 1.779679

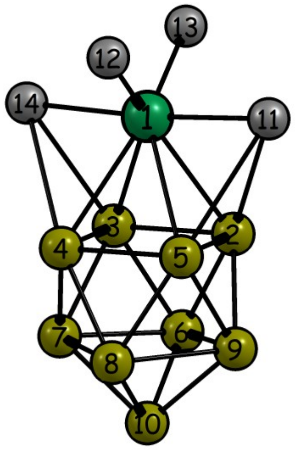


2. -1943.99359029 +5.9 $C_{2v}$

| | | 1 | 2 | 3 | 4 | 5 |
|---|---|---|---|---|---|---|
| 1 | Fe | 0.000000 | | | | |
| 2 | B | 2.202217 | 0.000000 | | | |
| 3 | B | 2.202821 | 1.792196 | 0.000000 | | |
| 4 | B | 2.200497 | 2.654129 | 1.959772 | 0.000000 | |
| 5 | B | 2.202111 | 1.958416 | 2.655503 | 1.791098 | 0.000000 |
| 6 | B | 3.508125 | 1.775410 | 1.775016 | 2.881005 | 2.880359 |
| 7 | B | 3.448745 | 2.811181 | 1.784441 | 1.785081 | 2.810835 |
| 8 | B | 3.508106 | 2.881775 | 2.882245 | 1.775597 | 1.775398 |
| 9 | B | 3.450493 | 1.785468 | 2.811649 | 2.811177 | 1.785157 |
| 10 | B | 4.329028 | 2.895344 | 2.894328 | 2.894170 | 2.894268 |

| | | 6 | 7 | 8 | 9 | 10 |
|---|---|---|---|---|---|---|
| 6 | B | 0.000000 | | | | |
| 7 | B | 1.861361 | 0.000000 | | | |
| 8 | B | 2.628903 | 1.861776 | 0.000000 | | |
| 9 | B | 1.860322 | 2.632521 | 1.861713 | 0.000000 | |
| 10 | B | 1.699172 | 1.741296 | 1.698819 | 1.741842 | 0.000000 |

Fe1-C11: 1.809119 Fe1-C12: 1.794649
Fe1-C13: 1.794522 Fe1-C14: 1.809394

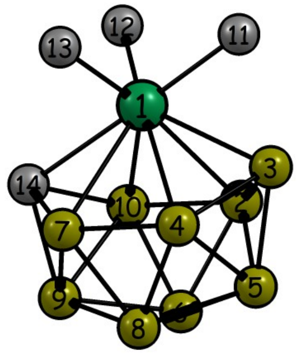


3. -1943.98032802 +14.3 $C_s$

| | | 1 | 2 | 3 | 4 | 5 |
|---|---|---|---|---|---|---|
| 1 | Fe | 0.000000 | | | | |
| 2 | B | 2.253559 | 0.000000 | | | |
| 3 | B | 2.085999 | 1.772367 | 0.000000 | | |
| 4 | B | 2.251244 | 2.696936 | 1.771526 | 0.000000 | |
| 5 | B | 3.034035 | 1.824679 | 1.727267 | 1.825523 | 0.000000 |
| 6 | B | 3.184260 | 1.782966 | 2.932735 | 2.822269 | 1.771588 |
| 7 | B | 2.246217 | 3.215305 | 3.080367 | 1.719844 | 2.901242 |
| 8 | B | 3.184723 | 2.821949 | 2.932846 | 1.784543 | 1.771180 |
| 9 | B | 3.071141 | 2.936422 | 3.752442 | 2.936900 | 2.959021 |
| 10 | B | 2.243493 | 1.719257 | 3.078269 | 3.212788 | 2.900743 |

| | | 6 | 7 | 8 | 9 | 10 |
|---|---|---|---|---|---|---|
| 6 | B | 0.000000 | | | | |
| 7 | B | 2.829067 | 0.000000 | | | |
| 8 | B | 1.773315 | 1.776477 | 0.000000 | | |
| 9 | B | 1.764197 | 1.879891 | 1.765202 | 0.000000 | |
| 10 | B | 1.776929 | 2.733937 | 2.829486 | 1.880205 | 0.000000 |

Fe1-C11: 1.795651 Fe1-C12: 1.796154
Fe1-C13: 1.796164 Fe1-C14: 2.090484

Table S5D: Energy ranking for the $B_5H_5Fe(CO)_2$ structures at the PBE0/def2-TZVP level of theory.

| No. | Initial structure | Final Energy (a.u.) | Relative Energy (kcal/mol) |
|---|---|---|---|
| 1 | 10v-01-BicapSqAntipr–fe1–2co-pbe0 | -1718.5848830 | 0.00 |
| 2 | 10v-08-Isocloso—fe1—2co-pbe0 | -1718.5734370 | 7.18 |
| 3 | 10v-03-BicapCubB—fe2—2co-pbe0 | -1718.5734170 | 7.20 |
| 4 | 10v-04-PentagPrism—fe1—2co-pbe0 | -1718.5573040 | 17.31 |
| 5 | 10v-08-Isocloso—fe2—2co-pbe0 | -1718.5570360 | 17.47 |
| 6 | 10v-08-Isocloso—fe5—2co-r-92-pbe0 | -1718.5496150 | 22.13 |
| 7 | 10v-01-BicapSqAntipr–fe2–2co-pbe0 | -1718.5437400 | 25.82 |
| 8 | 10v-09-DistTetracapOh—fe3—2co-pbe0 | -1718.5394880 | 28.49 |
| 9 | 10v-07-TetrcTriPri2—fe4—2co-pbe0 | -1718.5362570 | 30.51 |
| 10 | 10v-07-TetrcTriPri2—fe1—2co-r-35-pbe0 | -1718.5362520 | 30.52 |
| 11 | 10v-07-TetrcTriPri2—fe1—2co-i-35-pbe0 | -1718.5362420 | 30.52 |
| 12 | 10v-07-TetrcTriPri2—fe6—2co-pbe0 | -1718.5343660 | 31.70 |
| 13 | 10v-07-TetrcTriPri2—fe5—2co-pbe0 | -1718.5343540 | 31.71 |
| 14 | 10v-07-TetrcTriPri2—fe3—2co-pbe0 | -1718.5343210 | 31.73 |
| 15 | 10v-06-TetrcTriPri1—fe2—2co-i-23-pbe0 | -1718.5317260 | 33.36 |
| 16 | 10v-06-TetrcTriPri1—fe2—2co-r-23-pbe0 | -1718.5317190 | 33.36 |
| 17 | 10v-08-Isocloso—fe3—2co-r-52-pbe0 | -1718.5312680 | 33.64 |
| 18 | 10v-08-Isocloso—fe3—2co-i-52-pbe0 | -1718.5312570 | 33.65 |
| 19 | 10v-08-Isocloso—fe5—2co-i-92-pbe0 | -1718.5312440 | 33.66 |
| 20 | 10v-03-BicapCubB—fe4—2co-pbe0 | -1718.5289180 | 35.12 |
| 21 | 10v-08-Isocloso—fe4—2co-pbe0 | -1718.5288970 | 35.13 |
| 22 | 10v-07-TetrcTriPri2—fe2—2co-pbe0 | -1718.5287100 | 35.25 |
| 23 | 10v-09-DistTetracapOh—fe2—2co-pbe0 | -1718.5207010 | 40.27 |
| 24 | 10v-10-BisDisphenoid-Like—fe3—2co-pbe0 | -1718.5206870 | 40.28 |
| 25 | 10v-10-BisDisphenoid-Like—fe5—2co-pbe0 | -1718.5206520 | 40.31 |
| 26 | 10v-05-PentAntiPr—fe1—2co-pbe0 | -1718.5160430 | 43.20 |
| 27 | 10v-03-BicapCubB—fe1—2co-pbe0 | -1718.5158710 | 43.31 |
| 28 | 10v-06-TetrcTriPri1—fe3—2co-pbe0 | -1718.5053280 | 49.92 |
| 29 | 10v-06-TetrcTriPri1—fe1—2co-pbe0 | -1718.5011920 | 52.52 |
| 30 | 10v-03-BicapCubB—fe3—2co-pbe0 | -1718.4989050 | 53.95 |
| 31 | 10v-10-BisDisphenoid-Like—fe2—2co-pbe0 | -1718.4951970 | 56.28 |
| 32 | 10v-02-BicapCubA—fe1—2co-pbe0 | -1718.4906930 | 59.11 |
| 33 | 10v-09-DistTetracapOh—fe1—2co-pbe0 | -1718.4820650 | 64.52 |
| 34 | 10v-02-BicapCubA—fe2—2co-pbe0 | -1718.4820130 | 64.55 |
| 35 | 10v-10-BisDisphenoid-Like—fe4—2co-pbe0 | -1718.4454070 | 87.52 |
| 36 | 10v-10-BisDisphenoid-Like—fe1—2co-pbe0 | -1718.4453960 | 87.53 |

Table S5E: Energy ranking for the $B_5H_5Fe(CO)_2$ structures at the PBE0/def2-TZVP level of theory.

| No. | Initial structure | Final Energy (a.u.) | Relative Energy (kcal/mol) |
|---|---|---|---|
| 1 | 10v-07-TetrcTriPri2—fe1—3co-pbe0 | -1831.8067370 | 0.00 |
| 2 | 10v-07-TetrcTriPri2—fe4—3co-pbe0 | -1831.8067280 | 0.01 |
| 3 | 10v-08-Isocloso—fe1—3co-pbe0 | -1831.8067210 | 0.01 |
| 4 | 10v-03-BicapCubB—fe2—3co-pbe0 | -1831.8066660 | 0.04 |
| 5 | 10v-06-TetrcTriPri1—fe2—3co-pbe0 | -1831.8065890 | 0.09 |
| 6 | 10v-01-BicapSqAntipr–fe1–3co-pbe0 | -1831.7999540 | 4.26 |
| 7 | 10v-07-TetrcTriPri2—fe3—3co-pbe0 | -1831.7789010 | 17.47 |
| 8 | 10v-03-BicapCubB—fe1—3co-pbe0 | -1831.7788630 | 17.49 |
| 9 | 10v-07-TetrcTriPri2—fe6—3co-pbe0 | -1831.7788490 | 17.50 |
| 10 | 10v-08-Isocloso—fe2—3co-pbe0 | -1831.7788350 | 17.51 |
| 11 | 10v-10-BisDisphenoid-Like—fe3—3co-pbe0 | -1831.7761580 | 19.19 |
| 12 | 10v-01-BicapSqAntipr–fe2–3co-pbe0 | -1831.7721260 | 21.72 |
| 13 | 10v-04-PentagPrism—fe1—3co-pbe0 | -1831.7689450 | 23.71 |
| 14 | 10v-07-TetrcTriPri2—fe5—3co-pbe0 | -1831.7689420 | 23.72 |
| 15 | 10v-07-TetrcTriPri2—fe2—3co-pbe0 | -1831.7689080 | 23.74 |
| 16 | 10v-08-Isocloso—fe4—3co-pbe0 | -1831.7689040 | 23.74 |
| 17 | 10v-03-BicapCubB—fe4—3co-pbe0 | -1831.7688930 | 23.75 |
| 18 | 10v-06-TetrcTriPri1—fe3—3co-pbe0 | -1831.7676130 | 24.55 |
| 19 | 10v-08-Isocloso—fe3—3co-pbe0 | -1831.7598430 | 29.43 |
| 20 | 10v-08-Isocloso—fe5—3co-pbe0 | -1831.7598170 | 29.44 |
| 21 | 10v-05-PentAntiPr—fe1—3co-pbe0 | -1831.7499060 | 35.66 |
| 22 | 10v-03-BicapCubB—fe3—3co-pbe0 | -1831.7469790 | 37.50 |
| 23 | 10v-10-BisDisphenoid-Like—fe2—3co-pbe0 | -1831.7441920 | 39.25 |
| 24 | 10v-10-BisDisphenoid-Like—fe5—3co-pbe0 | -1831.7441820 | 39.25 |
| 25 | 10v-10-BisDisphenoid-Like—fe1—3co-pbe0 | -1831.7441360 | 39.28 |
| 26 | 10v-09-DistTetracapOh—fe2—3co-r-20-pbe0 | -1831.7437340 | 39.54 |
| 27 | 10v-09-DistTetracapOh—fe2—3co-i-20-pbe0 | -1831.7437260 | 39.54 |
| 28 | 10v-09-DistTetracapOh—fe1—3co-pbe0 | -1831.7240060 | 51.91 |
| 29 | 10v-10-BisDisphenoid-Like—fe4—3co-pbe0 | -1831.7239820 | 51.93 |
| 30 | 10v-02-BicapCubA—fe1—3co-pbe0 | -1831.7239750 | 51.93 |
| 31 | 10v-02-BicapCubA—fe2—3co-pbe0 | -1831.7239250 | 51.97 |
| 32 | 10v-09-DistTetracapOh—fe3—3co-pbe0 | -1831.7141680 | 58.09 |
| 33 | 10v-06-TetrcTriPri1—fe1—3co-pbe0 | -1831.6715370 | 84.84 |

Table S5F: Energy ranking for the $B_5H_5Fe(CO)_2$ structures at the PBE0/def2-TZVP level of theory.

| No. | Initial structure | Final Energy (a.u.) | Relative Energy (kcal/mol) |
|---|---|---|---|
| 1 | 10v-01-BicapSqAntipr–fe2–4co-r-19-pbe0 | -1945.0100740 | 0.00 |
| 2 | 10v-04-PentagPrism—fe1—4co-pbe0 | -1945.0029220 | 4.49 |
| 3 | 10v-01-BicapSqAntipr–fe1–4co-r-119-pbe0 | -1944.9913080 | 11.78 |
| 4 | 10v-03-BicapCubB—fe4—4co-pbe0 | -1944.9913070 | 11.78 |
| 5 | 10v-08-Isocloso—fe5—4co-pbe0 | -1944.9912910 | 11.79 |
| 6 | 10v-03-BicapCubB—fe1—4co-pbe0 | -1944.9912780 | 11.79 |
| 7 | 10v-08-Isocloso—fe2—4co-pbe0 | -1944.9912730 | 11.80 |
| 8 | 10v-01-BicapSqAntipr–fe1–4co-i-119-pbe0 | -1944.9912630 | 11.80 |
| 9 | 10v-07-TetrcTriPri2—fe2—4co-pbe0 | -1944.9912600 | 11.81 |
| 10 | 10v-07-TetrcTriPri2—fe5—4co-pbe0 | -1944.9912440 | 11.82 |
| 11 | 10v-08-Isocloso—fe3—4co-pbe0 | -1944.9912400 | 11.82 |
| 12 | 10v-07-TetrcTriPri2—fe1—4co-pbe0 | -1944.9776830 | 20.33 |
| 13 | 10v-07-TetrcTriPri2—fe4—4co-pbe0 | -1944.9775000 | 20.44 |
| 14 | 10v-08-Isocloso—fe1—4co-pbe0 | -1944.9774720 | 20.46 |
| 15 | 10v-03-BicapCubB—fe2—4co-pbe0 | -1944.9743430 | 22.42 |
| 16 | 10v-03-BicapCubB—fe3—4co-pbe0 | -1944.9740530 | 22.60 |
| 17 | 10v-10-BisDisphenoid-Like—fe4—4co-pbe0 | -1944.9680570 | 26.37 |
| 18 | 10v-01-BicapSqAntipr–fe2–4co-i-19-pbe0 | -1944.9551150 | 34.49 |
| 19 | 10v-07-TetrcTriPri2—fe3—4co-pbe0 | -1944.9496440 | 37.92 |
| 20 | 10v-07-TetrcTriPri2—fe6—4co-i-17-pbe0 | -1944.9496270 | 37.93 |
| 21 | 10v-07-TetrcTriPri2—fe6—4co-r-17-pbe0 | -1944.9496160 | 37.94 |
| 22 | 10v-05-PentAntiPr—fe1—4co-pbe0 | -1944.9465050 | 39.89 |
| 23 | 10v-08-Isocloso—fe4—4co-pbe0 | -1944.9396590 | 44.19 |
| 24 | 10v-06-TetrcTriPri1—fe3—4co-pbe0 | -1944.9387060 | 44.78 |
| 25 | 10v-10-BisDisphenoid-Like—fe3—4co-pbe0 | -1944.9385370 | 44.89 |
| 26 | 10v-10-BisDisphenoid-Like—fe5—4co-pbe0 | -1944.9385110 | 44.91 |
| 27 | 10v-09-DistTetracapOh—fe3—4co-pbe0 | -1944.9384920 | 44.92 |
| 28 | 10v-10-BisDisphenoid-Like—fe2—4co-pbe0 | -1944.9213310 | 55.69 |
| 29 | 10v-06-TetrcTriPri1—fe2—4co-pbe0 | -1944.9194990 | 56.84 |
| 30 | 10v-09-DistTetracapOh—fe2—4co-pbe0 | -1944.9150190 | 59.65 |
| 31 | 10v-10-BisDisphenoid-Like—fe1—4co-pbe0 | -1944.9144730 | 59.99 |
| 32 | 10v-02-BicapCubA—fe1—4co-pbe0 | -1944.9143900 | 60.04 |
| 33 | 10v-06-TetrcTriPri1—fe1—4co-pbe0 | -1944.9016960 | 68.01 |
| 34 | 10v-09-DistTetracapOh—fe1—4co-pbe0 | -1944.8938770 | 72.91 |
| 35 | 10v-02-BicapCubA—fe2—4co-pbe0 | -1944.8938730 | 72.92 |

Table S6A: Distance table for the lowest-lying $B_{10}H_{10}Fe(CO)_2$ optimized structures obtained at the PBE0/def2-TZVP level of theory. Included are the zero-point corrected absolute energy in (a.u.) at the DLPNO-CCSD(T)/def2-QZVP level of theory with zero-point energy obtained from the PBE0/def2-TZVP computations, relative energies in (kcal/mol) and symmetry. For clarity, only the atoms forming the cluster framework are shown.

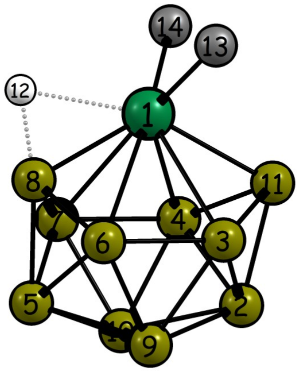


1. -1743.03531904 0.0 $C_s$

| | | 1 | 2 | 3 | 4 | 5 |
|---|---|---|---|---|---|---|
| 1 | Fe | 0.000000 | | | | |
| 2 | B | 2.983030 | 0.000000 | | | |
| 3 | B | 2.231026 | 1.809048 | 0.000000 | | |
| 4 | B | 2.227736 | 1.809504 | 2.694029 | 0.000000 | |
| 5 | B | 3.053190 | 2.967476 | 2.907545 | 2.910092 | 0.000000 |
| 6 | B | 2.335318 | 2.895980 | 1.707454 | 3.248689 | 1.855957 |
| 7 | B | 2.332853 | 2.896569 | 3.247568 | 1.710886 | 1.854964 |
| 8 | B | 1.835331 | 3.441761 | 2.815433 | 2.816331 | 1.697176 |
| 9 | B | 3.219103 | 1.787530 | 1.800288 | 2.833470 | 1.792722 |
| 10 | B | 3.217029 | 1.788380 | 2.832178 | 1.802045 | 1.792429 |
| 11 | B | 2.018307 | 1.733172 | 1.791680 | 1.790086 | 3.747166 |
| 12 | H | 1.842750 | 4.362719 | 3.498551 | 3.502005 | 2.944773 |

| | | 6 | 7 | 8 | 9 | 10 |
|---|---|---|---|---|---|---|
| 6 | B | 0.000000 | | | | |
| 7 | B | 2.831849 | 0.000000 | | | |
| 8 | B | 1.715282 | 1.713458 | 0.000000 | | |
| 9 | B | 1.774659 | 2.858481 | 2.784910 | 0.000000 | |
| 10 | B | 2.858861 | 1.773152 | 2.783596 | 1.774551 | 0.000000 |
| 11 | B | 3.118129 | 3.119122 | 3.370322 | 2.981280 | 2.981295 |
| 12 | H | 2.556740 | 2.560244 | 1.258315 | 3.923060 | 3.923630 |

| | | 11 | 12 |
|---|---|---|---|
| 11 | B | 0.000000 | |
| 12 | H | 3.813241 | 0.000000 |

Fe1-C13: 1.803132 Fe1-C14: 1.803385

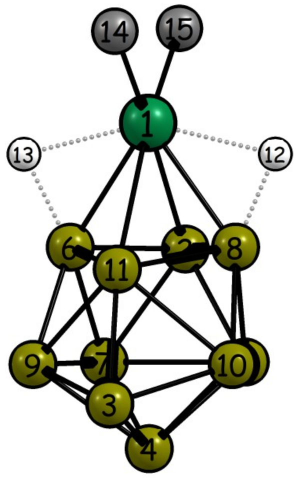


2. -1743.03487269 +0.3 $C_s$

| | | 1 | 2 | 3 | 4 | 5 |
|---|---|---|---|---|---|---|
| 1 | Fe | 0.000000 | | | | |
| 2 | B | 2.233610 | 0.000000 | | | |
| 3 | B | 3.908929 | 3.727808 | 0.000000 | | |
| 4 | B | 4.133278 | 2.952322 | 1.749785 | 0.000000 | |
| 5 | B | 3.366911 | 1.772770 | 2.928370 | 1.797978 | 0.000000 |
| 6 | B | 1.932170 | 1.753107 | 2.900507 | 2.787085 | 2.670626 |
| 7 | B | 3.367367 | 1.773943 | 2.929175 | 1.797304 | 1.879509 |
| 8 | B | 1.932534 | 1.753395 | 2.899925 | 2.788222 | 1.732037 |
| 9 | B | 3.400835 | 2.967012 | 1.707221 | 1.839678 | 2.872809 |
| 10 | B | 3.401932 | 2.967040 | 1.706504 | 1.840880 | 1.810412 |
| 11 | B | 2.262743 | 2.865961 | 1.735379 | 2.642190 | 2.841871 |
| 12 | H | 1.721692 | 2.550922 | 3.948908 | 4.094565 | 2.936030 |
| 13 | H | 1.719526 | 2.550504 | 3.949901 | 4.093991 | 3.860804 |

| | | 6 | 7 | 8 | 9 | 10 |
|---|---|---|---|---|---|---|
| 6 | B | 0.000000 | | | | |
| 7 | B | 1.731750 | 0.000000 | | | |
| 8 | B | 2.201221 | 2.672282 | 0.000000 | | |
| 9 | B | 1.727514 | 1.810535 | 2.968855 | 0.000000 | |
| 10 | B | 2.969270 | 2.873845 | 1.727934 | 2.648668 | 0.000000 |
| 11 | B | 1.873366 | 2.842443 | 1.873174 | 1.902954 | 1.903934 |
| 12 | H | 3.018033 | 3.862274 | 1.318603 | 4.074950 | 2.782468 |
| 13 | H | 1.319176 | 2.936347 | 3.017346 | 2.782707 | 4.075454 |

| | | 11 | 12 | 13 |
|---|---|---|---|---|
| 11 | B | 0.000000 | | |
| 12 | H | 2.599162 | 0.000000 | |
| 13 | H | 2.599112 | 3.346770 | 0.000000 |

Fe1-C14: 1.803241 Fe1-C15: 1.791748

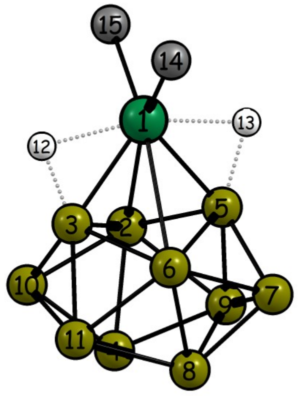

3. -1743.01074350 +15.4 $C_2$

| | | 1 | 2 | 3 | 4 | 5 |
|---|---|---|---|---|---|---|
| 1 | Fe | 0.000000 | | | | |
| 2 | B | 2.401199 | 0.000000 | | | |
| 3 | B | 1.893195 | 1.956766 | 0.000000 | | |
| 4 | B | 3.817469 | 1.884574 | 2.538511 | 0.000000 | |
| 5 | B | 1.894320 | 1.818600 | 2.375849 | 2.825245 | 0.000000 |
| 6 | B | 2.403172 | 2.768405 | 1.817548 | 2.826027 | 1.955332 |
| 7 | B | 3.256671 | 2.965281 | 3.195046 | 2.928691 | 1.642207 |
| 8 | B | 3.818367 | 2.827628 | 2.824950 | 1.782628 | 2.538325 |
| 9 | B | 3.448465 | 1.906099 | 3.086043 | 1.815759 | 1.739715 |
| 10 | B | 3.254202 | 1.834045 | 1.642555 | 1.687737 | 3.194964 |
| 11 | B | 3.448459 | 2.682726 | 1.739226 | 1.819530 | 3.086758 |
| 12 | H | 1.650225 | 2.845915 | 1.331577 | 3.829827 | 3.089526 |
| 13 | H | 1.650554 | 2.673497 | 3.088399 | 4.079606 | 1.331423 |

| | | 6 | 7 | 8 | 9 | 10 |
|---|---|---|---|---|---|---|
| 6 | B | 0.000000 | | | | |
| 7 | B | 1.833693 | 0.000000 | | | |
| 8 | B | 1.883141 | 1.687747 | 0.000000 | | |
| 9 | B | 2.680764 | 1.731920 | 1.818948 | 0.000000 | |
| 10 | B | 2.964200 | 3.861409 | 2.928795 | 3.044057 | 0.000000 |
| 11 | B | 1.905910 | 3.045483 | 1.816967 | 2.938544 | 1.731895 |
| 12 | H | 2.675097 | 4.152683 | 4.080274 | 4.213352 | 2.661297 |
| 13 | H | 2.845719 | 2.662399 | 3.829986 | 2.952631 | 4.150929 |

| | | 11 | 12 | 13 |
|---|---|---|---|---|
| 11 | B | 0.000000 | | |
| 12 | H | 2.952272 | 0.000000 | |
| 13 | H | 4.213402 | 3.270966 | 0.000000 |

Fe1-C14: 1.792073 Fe1-C15: 1.791693

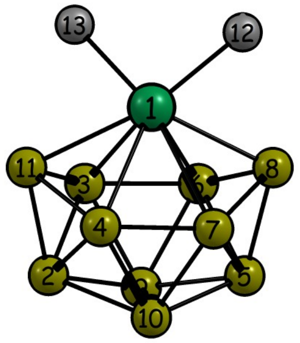

4. -1743.00902966 +16.5 $C_1$

| | | 1 | 2 | 3 | 4 | 5 |
|---|---|---|---|---|---|---|
| 1 | Fe | 0.000000 | | | | |
| 2 | B | 3.004302 | 0.000000 | | | |
| 3 | B | 2.217425 | 1.833549 | 0.000000 | | |
| 4 | B | 2.163456 | 1.832557 | 2.657057 | 0.000000 | |
| 5 | B | 2.997357 | 2.956134 | 2.892655 | 2.876903 | 0.000000 |
| 6 | B | 2.224835 | 2.915640 | 1.717720 | 3.161393 | 1.830563 |
| 7 | B | 2.206356 | 2.909877 | 3.189271 | 1.694873 | 1.835260 |
| 8 | B | 2.062883 | 3.738376 | 3.104659 | 3.023224 | 1.736135 |
| 9 | B | 3.156912 | 1.778880 | 1.776929 | 2.802429 | 1.768737 |
| 10 | B | 3.154221 | 1.777584 | 2.816391 | 1.787453 | 1.769748 |
| 11 | B | 2.080596 | 1.726704 | 1.824942 | 1.726157 | 3.734838 |

| | | 6 | 7 | 8 | 9 | 10 |
|---|---|---|---|---|---|---|
| 6 | B | 0.000000 | | | | |
| 7 | B | 2.699180 | 0.000000 | | | |
| 8 | B | 1.824540 | 1.739509 | 0.000000 | | |
| 9 | B | 1.785201 | 2.824741 | 2.959361 | 0.000000 | |
| 10 | B | 2.826557 | 1.788328 | 2.927173 | 1.775282 | 0.000000 |
| 11 | B | 3.124491 | 3.071580 | 3.709053 | 2.958479 | 2.928881 |

| | | 11 |
|---|---|---|
| 11 | B | 0.000000 |

Fe1-C12: 1.793916 Fe1-C13: 1.790082

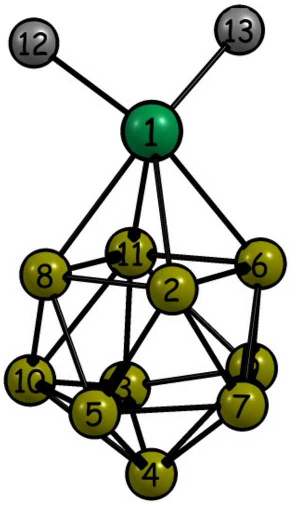


5. -1742.96460214 +44.4 $C_s$

| | | 1 | 2 | 3 | 4 | 5 |
|---|---|---|---|---|---|---|
| 1 | Fe | 0.000000 | | | | |
| 2 | B | 2.064330 | 0.000000 | | | |
| 3 | B | 3.952314 | 3.602698 | 0.000000 | | |
| 4 | B | 4.209581 | 2.951343 | 1.767740 | 0.000000 | |
| 5 | B | 3.417814 | 1.793118 | 2.918806 | 1.779874 | 0.000000 |
| 6 | B | 2.103473 | 1.774702 | 2.927433 | 2.862395 | 2.794765 |
| 7 | B | 3.410996 | 1.793355 | 2.917650 | 1.778376 | 1.778142 |
| 8 | B | 2.112533 | 1.770808 | 2.932186 | 2.867704 | 1.791150 |
| 9 | B | 3.485564 | 2.865624 | 1.731433 | 1.821101 | 2.821673 |
| 10 | B | 3.494890 | 2.862871 | 1.732824 | 1.819406 | 1.784263 |
| 11 | B | 2.234205 | 2.616255 | 1.792885 | 2.690418 | 2.822282 |

| | | 6 | 7 | 8 | 9 | 10 |
|---|---|---|---|---|---|---|
| 6 | B | 0.000000 | | | | |
| 7 | B | 1.787408 | 0.000000 | | | |
| 8 | B | 2.592321 | 2.794876 | 0.000000 | | |
| 9 | B | 1.684390 | 1.787274 | 3.130233 | 0.000000 | |
| 10 | B | 3.127187 | 2.817651 | 1.690283 | 2.677810 | 0.000000 |
| 11 | B | 1.911258 | 2.819353 | 1.916384 | 1.979057 | 1.984511 |

| | | 11 |
|---|---|---|
| 11 | B | 0.000000 |

Fe1-C12: 1.777545 Fe1-C13: 1.778976

Table S6B: Distance table for the lowest-lying $B_{10}H_{10}Fe(CO)_3$ optimized structures obtained at the PBE0/def2-TZVP level of theory. Included are the zero-point corrected absolute energy in (a.u.) at the DLPNO-CCSD(T)/def2-QZVP level of theory with zero-point energy obtained from the PBE0/def2-TZVP computations, relative energies in (kcal/mol) and symmetry. For clarity, only the atoms forming the cluster framework are shown.

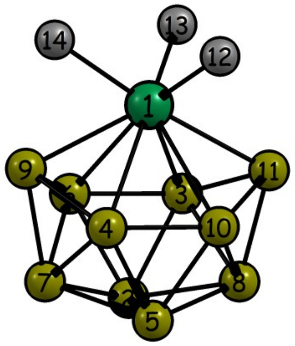


1. -1856.23372140 0.0 $C_s$

| | | 1 | 2 | 3 | 4 | 5 |
|---|---|---|---|---|---|---|
| 1 | Fe | 0.000000 | | | | |
| 2 | B | 3.213270 | 0.000000 | | | |
| 3 | B | 2.235674 | 1.783218 | 0.000000 | | |
| 4 | B | 2.271514 | 2.825348 | 3.186194 | 0.000000 | |
| 5 | B | 3.216113 | 1.767494 | 2.809209 | 1.784662 | 0.000000 |
| 6 | B | 2.266182 | 1.784950 | 1.707273 | 2.716026 | 2.826587 |
| 7 | B | 3.050461 | 1.764926 | 2.884654 | 1.833186 | 1.764321 |
| 8 | B | 3.052619 | 1.777495 | 1.830498 | 2.911124 | 1.777425 |
| 9 | B | 2.069385 | 2.936952 | 3.050593 | 1.787525 | 2.940151 |
| 10 | B | 2.237591 | 2.806928 | 2.663015 | 1.704214 | 1.782393 |
| 11 | B | 2.106339 | 2.946196 | 1.777062 | 3.107134 | 2.949521 |

| | | 6 | 7 | 8 | 9 | 10 |
|---|---|---|---|---|---|---|
| 6 | B | 0.000000 | | | | |
| 7 | B | 1.835465 | 0.000000 | | | |
| 8 | B | 2.911689 | 2.952342 | 0.000000 | | |
| 9 | B | 1.780906 | 1.743295 | 3.723349 | 0.000000 | |
| 10 | B | 3.183224 | 2.880707 | 1.830632 | 3.050622 | 0.000000 |
| 11 | B | 3.102697 | 3.735665 | 1.726590 | 3.691771 | 1.784635 |

| | | 11 |
|---|---|---|
| 11 | B | 0.000000 |

Fe1-C12: 1.785145 Fe1-C13: 1.786433 Fe1-C14: 1.787742

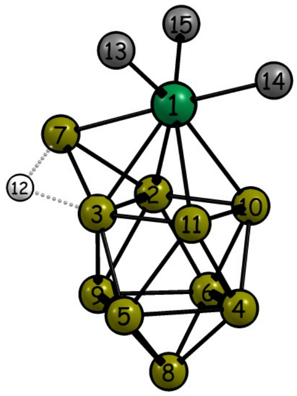


2. -1856.21795027 +9.9 $C_1$

| | | 1 | 2 | 3 | 4 | 5 |
|---|---|---|---|---|---|---|
| 1 | Fe | 0.000000 | | | | |
| 2 | B | 2.139967 | 0.000000 | | | |
| 3 | B | 2.100625 | 1.881688 | 0.000000 | | |
| 4 | B | 3.377239 | 2.827801 | 2.692391 | 0.000000 | |
| 5 | B | 3.457237 | 2.897972 | 1.731378 | 1.865574 | 0.000000 |
| 6 | B | 3.447976 | 1.784444 | 2.765046 | 1.885546 | 2.623851 |
| 7 | B | 2.034791 | 1.895428 | 1.652958 | 4.060876 | 3.339153 |
| 8 | B | 4.283693 | 2.948473 | 2.852646 | 1.725888 | 1.710182 |
| 9 | B | 3.458452 | 1.853400 | 1.812265 | 2.622656 | 1.853764 |
| 10 | B | 2.112619 | 1.809843 | 2.526174 | 1.798336 | 2.897331 |
| 11 | B | 2.146962 | 2.686658 | 1.765255 | 1.762466 | 1.844031 |
| 12 | H | 2.760025 | 2.693182 | 1.287061 | 3.946886 | 2.636407 |

| | | 6 | 7 | 8 | 9 | 10 |
|---|---|---|---|---|---|---|
| 6 | B | 0.000000 | | | | |
| 7 | B | 3.527053 | 0.000000 | | | |
| 8 | B | 1.703966 | 4.132372 | 0.000000 | | |
| 9 | B | 1.816437 | 2.633815 | 1.733236 | 0.000000 | |
| 10 | B | 1.831246 | 3.224039 | 2.925253 | 2.857073 | 0.000000 |
| 11 | B | 2.900419 | 3.113051 | 2.928440 | 2.897933 | 1.916155 |
| 12 | H | 3.843220 | 1.421075 | 3.827971 | 2.471726 | 3.731184 |

| | | 11 | 12 |
|---|---|---|---|
| 11 | B | 0.000000 | |
| 12 | H | 2.904235 | 0.000000 |

Fe1-C13: 1.772327 Fe1-C14: 1.774002 Fe1-C15: 1.772936

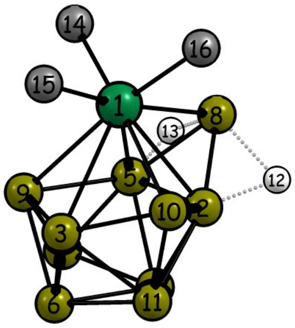


3. -1856.21591915 +11.2 $C_1$

| | | 1 | 2 | 3 | 4 | 5 |
|---|---|---|---|---|---|---|
| 1 | Fe | 0.000000 | | | | |
| 2 | B | 2.161953 | 0.000000 | | | |
| 3 | B | 2.250599 | 2.524742 | 0.000000 | | |
| 4 | B | 3.431494 | 2.917169 | 2.933578 | 0.000000 | |
| 5 | B | 2.199997 | 1.766688 | 2.779365 | 1.665501 | 0.000000 |
| 6 | B | 3.393203 | 2.790449 | 1.808936 | 1.716025 | 2.522557 |
| 7 | B | 3.386757 | 1.834148 | 2.815043 | 1.709510 | 1.792952 |
| 8 | B | 2.149895 | 1.713404 | 3.665870 | 3.360193 | 1.714036 |
| 9 | B | 2.286121 | 2.855422 | 1.797468 | 1.735501 | 1.887397 |
| 10 | B | 2.091494 | 1.729319 | 1.774112 | 3.850758 | 3.011454 |
| 11 | B | 3.112607 | 1.807889 | 1.835377 | 2.942978 | 2.777469 |
| 12 | H | 2.886468 | 1.270391 | 3.761150 | 3.840803 | 2.487728 |
| 13 | H | 2.877521 | 2.480674 | 4.011324 | 2.572078 | 1.283718 |

| | | 6 | 7 | 8 | 9 | 10 |
|---|---|---|---|---|---|---|
| 6 | B | 0.000000 | | | | |
| 7 | B | 1.797481 | 0.000000 | | | |
| 8 | B | 3.956637 | 2.893780 | 0.000000 | | |
| 9 | B | 1.815544 | 2.632737 | 3.299666 | 0.000000 | |
| 10 | B | 2.993421 | 2.998238 | 3.035416 | 3.084179 | 0.000000 |
| 11 | B | 1.807233 | 1.796361 | 3.468955 | 2.862737 | 1.722365 |
| 12 | H | 3.990043 | 2.670998 | 1.504151 | 3.976770 | 2.628839 |
| 13 | H | 3.753834 | 2.679142 | 1.447591 | 3.019075 | 3.956799 |

| | | 11 | 12 | 13 |
|---|---|---|---|---|
| 11 | B | 0.000000 | | |
| 12 | H | 2.899872 | 0.000000 | |
| 13 | H | 3.880408 | 2.571341 | 0.000000 |

Fe1-C14: 1.783354 Fe1-C15: 1.772839 Fe1-C16: 1.765928

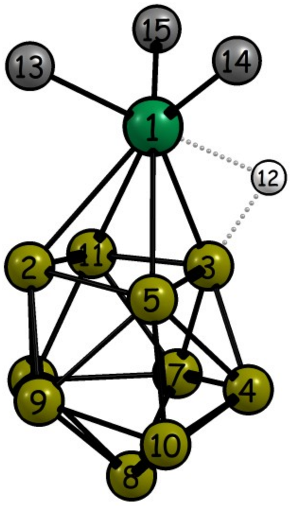


4. -1856.21239174 +13.4 $C_1$

| | | 1 | 2 | 3 | 4 | 5 |
|---|---|---|---|---|---|---|
| 1 | Fe | 0.000000 | | | | |
| 2 | B | 2.273885 | 0.000000 | | | |
| 3 | B | 1.935590 | 2.260429 | 0.000000 | | |
| 4 | B | 3.404152 | 3.049546 | 1.683118 | 0.000000 | |
| 5 | B | 2.341195 | 1.879669 | 1.852095 | 1.937552 | 0.000000 |
| 6 | B | 3.609128 | 1.769750 | 2.611531 | 2.847061 | 2.802321 |
| 7 | B | 3.452629 | 2.748373 | 1.708755 | 1.827966 | 2.856900 |
| 8 | B | 4.291920 | 2.869339 | 2.728501 | 1.825332 | 2.658530 |
| 9 | B | 3.661229 | 1.760918 | 2.919138 | 2.632161 | 1.887226 |
| 10 | B | 4.029061 | 2.964719 | 2.859875 | 1.709203 | 1.765867 |
| 11 | B | 2.367222 | 1.760040 | 1.747550 | 2.977808 | 2.817079 |
| 12 | H | 1.643127 | 3.155038 | 1.306263 | 2.645515 | 2.604508 |

| | | 6 | 7 | 8 | 9 | 10 |
|---|---|---|---|---|---|---|
| 6 | B | 0.000000 | | | | |
| 7 | B | 1.842197 | 0.000000 | | | |
| 8 | B | 1.793278 | 1.781305 | 0.000000 | | |
| 9 | B | 1.789917 | 2.852225 | 1.839773 | 0.000000 | |
| 10 | B | 2.920932 | 2.945097 | 1.765009 | 1.705193 | 0.000000 |
| 11 | B | 1.770365 | 1.807019 | 2.971683 | 2.943643 | 3.732380 |
| 12 | H | 3.841832 | 2.858779 | 4.003821 | 4.074311 | 3.880797 |

| | | 11 | 12 |
|---|---|---|---|
| 11 | B | 0.000000 | |
| 12 | H | 2.584082 | 0.000000 |

Fe1-C13: 1.796121 Fe1-C14: 1.790563 Fe1-C15: 1.784316

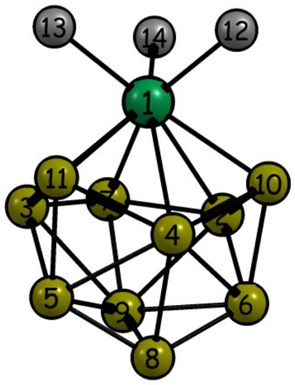


5. -1856.14092802 +58.2 $C_1$

| | | 1 | 2 | 3 | 4 | 5 |
|---|---|---|---|---|---|---|
| 1 | Fe | 0.000000 | | | | |
| 2 | B | 2.259521 | 0.000000 | | | |
| 3 | B | 2.282699 | 2.847430 | 0.000000 | | |
| 4 | B | 2.368029 | 2.786089 | 2.808844 | 0.000000 | |
| 5 | B | 3.139838 | 3.343319 | 1.817669 | 2.000578 | 0.000000 |
| 6 | B | 3.129159 | 1.823895 | 3.390315 | 1.977023 | 2.817128 |
| 7 | B | 2.220745 | 1.679484 | 1.651422 | 3.271591 | 2.922452 |
| 8 | B | 3.625546 | 2.934148 | 2.966189 | 1.891276 | 1.640027 |
| 9 | B | 3.071554 | 1.972376 | 2.026400 | 2.650615 | 1.962318 |
| 10 | B | 2.046869 | 1.731673 | 3.467023 | 1.884694 | 3.439917 |
| 11 | B | 2.060851 | 3.421321 | 1.781340 | 1.806518 | 1.716403 |
| | | 6 | 7 | 8 | 9 | 10 |
| 6 | B | 0.000000 | | | | |
| 7 | B | 2.974821 | 0.000000 | | | |
| 8 | B | 1.634019 | 3.281210 | 0.000000 | | |
| 9 | B | 1.969112 | 1.761688 | 1.742442 | 0.000000 | |
| 10 | B | 1.717330 | 2.995035 | 2.930144 | 2.949977 | 0.000000 |
| 11 | B | 3.380130 | 2.988236 | 2.884220 | 2.957063 | 3.083489 |
| | | 11 | | | | |
| 11 | B | 0.000000 | | | | |

Fe1-C12: 1.783481 Fe1-C13: 1.788394 Fe1-C14: 1.791247

Table S6C: Distance table for the lowest-lying $B_{10}H_{10}Fe(CO)_4$ optimized structures obtained at the PBE0/def2-TZVP level of theory. Included are the zero-point corrected absolute energy in (a.u.) at the DLPNO-CCSD(T)/def2-QZVP level of theory with zero-point energy obtained from the PBE0/def2-TZVP computations, relative energies in (kcal/mol) and symmetry. For clarity, only the atoms forming the cluster framework are shown.

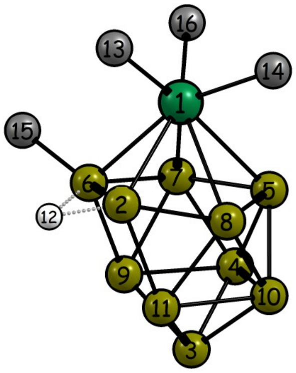


1. -1969.37708589 0.0 $C_1$

| | | 1 | 2 | 3 | 4 | 5 |
|---|---|---|---|---|---|---|
| 1 | Fe | 0.000000 | | | | |
| 2 | B | 2.123166 | 0.000000 | | | |
| 3 | B | 4.177612 | 3.364827 | 0.000000 | | |
| 4 | B | 3.463835 | 3.642018 | 1.745007 | 0.000000 | |
| 5 | B | 2.196063 | 3.004893 | 2.892992 | 1.786251 | 0.000000 |
| 6 | B | 2.112720 | 1.813134 | 3.201567 | 3.019893 | 2.923267 |
| 7 | B | 2.182597 | 2.946176 | 2.897534 | 1.762281 | 1.789965 |
| 8 | B | 2.185857 | 1.720166 | 2.888237 | 2.875040 | 1.827566 |
| 9 | B | 3.356518 | 2.813537 | 1.696002 | 1.855115 | 2.824731 |
| 10 | B | 3.458034 | 3.092588 | 1.728343 | 1.810300 | 1.783537 |
| 11 | B | 3.339775 | 1.905323 | 1.679224 | 2.680842 | 2.821758 |
| 12 | H | 2.821494 | 1.379042 | 3.453363 | 3.855976 | 3.746955 |

| | | 6 | 7 | 8 | 9 | 10 |
|---|---|---|---|---|---|---|
| 6 | B | 0.000000 | | | | |
| 7 | B | 1.786316 | 0.000000 | | | |
| 8 | B | 2.748273 | 2.787776 | 0.000000 | | |
| 9 | B | 1.816905 | 1.822951 | 3.017060 | 0.000000 | |
| 10 | B | 3.459558 | 2.846321 | 1.780175 | 2.682674 | 0.000000 |
| 11 | B | 2.544550 | 2.996955 | 1.807856 | 2.061381 | 1.865192 |
| 12 | H | 1.299224 | 2.989501 | 2.899832 | 2.411827 | 3.818631 |

| | | 11 | 12 |
|---|---|---|---|
| 11 | B | 0.000000 | |
| 12 | H | 2.324688 | 0.000000 |

Fe1-C13: 1.769292 Fe1-C14: 1.773325
Fe1-C15: 2.925706 Fe1-C16: 1.777470

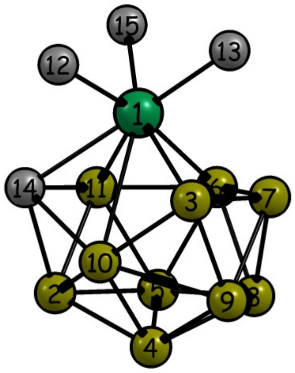


2. -1969.36006412 +10.7 $C_1$

| | | 1 | 2 | 3 | 4 | 5 |
|---|---|---|---|---|---|---|
| 1 | Fe | 0.000000 | | | | |
| 2 | B | 3.096024 | 0.000000 | | | |
| 3 | B | 2.079797 | 3.489336 | 0.000000 | | |
| 4 | B | 3.450835 | 1.730063 | 2.911678 | 0.000000 | |
| 5 | B | 3.292139 | 1.771512 | 3.727932 | 1.775167 | 0.000000 |
| 6 | B | 2.241085 | 2.881112 | 3.069518 | 2.781333 | 1.791983 |
| 7 | B | 2.270679 | 3.600219 | 1.845741 | 2.828089 | 2.922713 |
| 8 | B | 3.315658 | 2.913685 | 2.955421 | 1.770009 | 1.736397 |
| 9 | B | 3.112600 | 3.106887 | 1.698605 | 1.761683 | 2.930440 |
| 10 | B | 2.345588 | 1.948793 | 1.767025 | 1.841247 | 2.862637 |
| 11 | B | 2.249721 | 1.846050 | 3.604527 | 2.855537 | 1.765274 |

| | | 6 | 7 | 8 | 9 | 10 |
|---|---|---|---|---|---|---|
| 6 | B | 0.000000 | | | | |
| 7 | B | 1.769468 | 0.000000 | | | |
| 8 | B | 1.788885 | 1.769095 | 0.000000 | | |
| 9 | B | 2.861323 | 1.788855 | 1.774955 | 0.000000 | |
| 10 | B | 3.055305 | 2.734858 | 2.854607 | 1.937742 | 0.000000 |
| 11 | B | 1.787822 | 3.148919 | 2.939142 | 3.634513 | 2.790729 |

| | | 11 |
|---|---|---|
| 11 | B | 0.000000 |

Fe1-C12: 1.799969 Fe1-C13: 1.783990
Fe1-C14: 2.060034 Fe1-C15: 1.796882

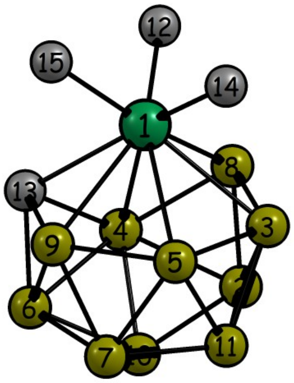


3. -1969.35646055 +12.9 $C_1$

| | | 1 | 2 | 3 | 4 | 5 |
|---|---|---|---|---|---|---|
| 1 | Fe | 0.000000 | | | | |
| 2 | B | 3.113683 | 0.000000 | | | |
| 3 | B | 2.229891 | 1.796131 | 0.000000 | | |
| 4 | B | 2.438050 | 1.898058 | 2.743979 | 0.000000 | |
| 5 | B | 2.224241 | 2.871032 | 1.806723 | 3.093203 | 0.000000 |
| 6 | B | 3.153757 | 3.087856 | 3.611283 | 1.980029 | 2.874145 |
| 7 | B | 3.307113 | 2.916567 | 2.941529 | 2.882709 | 1.783825 |
| 8 | B | 2.064057 | 1.712144 | 1.751159 | 1.850759 | 3.043964 |
| 9 | B | 2.231738 | 3.589051 | 3.127976 | 2.797366 | 1.761026 |
| 10 | B | 3.503245 | 1.750107 | 2.847850 | 1.858795 | 2.791757 |
| 11 | B | 3.297315 | 1.770561 | 1.773151 | 2.858310 | 1.787790 |

| | | 6 | 7 | 8 | 9 | 10 |
|---|---|---|---|---|---|---|
| 6 | B | 0.000000 | | | | |
| 7 | B | 1.762504 | 0.000000 | | | |
| 8 | B | 3.548524 | 3.734004 | 0.000000 | | |
| 9 | B | 1.838799 | 1.773257 | 3.567550 | 0.000000 | |
| 10 | B | 1.743495 | 1.771930 | 2.974097 | 2.854160 | 0.000000 |
| 11 | B | 2.916221 | 1.743884 | 2.923405 | 2.923176 | 1.773206 |

| | | 11 |
|---|---|---|
| 11 | B | 0.000000 |

Fe1-C12: 1.790697 Fe1-C13: 2.112943
Fe1-C14: 1.786476 Fe1-C15: 1.811246

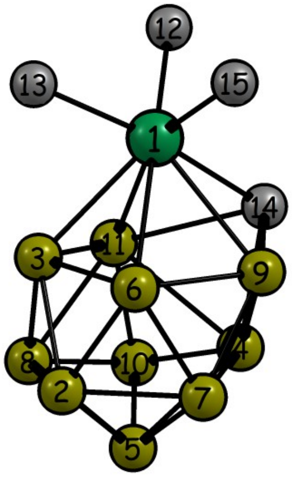


4. -1969.34192591 +22.1 $C_1$

| | | 1 | 2 | 3 | 4 | 5 |
|---|---|---|---|---|---|---|
| 1 | Fe | 0.000000 | | | | |
| 2 | B | 3.417097 | 0.000000 | | | |
| 3 | B | 2.122706 | 1.783316 | 0.000000 | | |
| 4 | B | 3.179811 | 2.835735 | 3.039145 | 0.000000 | |
| 5 | B | 4.094418 | 1.768348 | 2.843468 | 1.900072 | 0.000000 |
| 6 | B | 2.184528 | 1.776361 | 1.807488 | 2.852253 | 2.940689 |
| 7 | B | 3.345912 | 1.784410 | 2.782069 | 1.817762 | 1.753773 |
| 8 | B | 3.562403 | 1.784602 | 1.798251 | 2.724762 | 1.778127 |
| 9 | B | 2.183790 | 2.933833 | 2.873956 | 1.912990 | 3.084319 |
| 10 | B | 3.793641 | 2.907178 | 2.899838 | 1.776645 | 1.775907 |
| 11 | B | 2.244725 | 2.819884 | 1.801054 | 2.144492 | 2.719683 |

| | | 6 | 7 | 8 | 9 | 10 |
|---|---|---|---|---|---|---|
| 6 | B | 0.000000 | | | | |
| 7 | B | 1.789878 | 0.000000 | | | |
| 8 | B | 2.978571 | 2.848751 | 0.000000 | | |
| 9 | B | 1.780604 | 1.744699 | 3.608356 | 0.000000 | |
| 10 | B | 3.653155 | 2.930429 | 1.719088 | 3.399923 | 0.000000 |
| 11 | B | 2.859360 | 3.040226 | 1.893655 | 2.782333 | 1.731422 |

| | | 11 |
|---|---|---|
| 11 | B | 0.000000 |

Fe1-C12: 1.793977 Fe1-C13: 1.788151
Fe1-C14: 2.006559 Fe1-C15: 1.783246

Table S6D: Energy ranking for the $B_5H_5Fe(CO)_2$ structures at the PBE0/def2-TZVP level of theory.

| No. | Initial structure | Final Energy (a.u.) | Relative Energy (kcal/mol) |
|---|---|---|---|
| 1 | 11v-01-EdgeCoalscedIh–fe2–2co-pbe0 | -1744.0165450 | 0.00 |
| 2 | 11v-05-TricapCubeB–fe4–2co-pbe0 | -1744.0115440 | 3.14 |
| 3 | 11v-04-TricapCubeA–fe4–2co-pbe0 | -1744.0115430 | 3.14 |
| 4 | 11v-04-TricapCubeA–fe2–2co-pbe0 | -1744.0115390 | 3.14 |
| 5 | 11v-01-EdgeCoalscedIh–fe1–2co-pbe0 | -1744.0115240 | 3.15 |
| 6 | 11v-04-TricapCubeA–fe1–2co-pbe0 | -1744.0114830 | 3.18 |
| 7 | 11v-03-CapPentAnPr–fe3–2co-pbe0 | -1743.9968420 | 12.36 |
| 8 | 11v-05-TricapCubeB–fe1–2co-pbe0 | -1743.9881300 | 17.83 |
| 9 | 11v-01-EdgeCoalscedIh–fe4–2co-pbe0 | -1743.9804550 | 22.65 |
| 10 | 11v-01-EdgeCoalscedIh–fe3–2co-i-30-pbe0 | -1743.9689100 | 29.89 |
| 11 | 11v-01-EdgeCoalscedIh–fe3–2co-r-30-pbe0 | -1743.9688930 | 29.90 |
| 12 | 11v-06-CapPentPrA–fe1–2co-pbe0 | -1743.9624740 | 33.93 |
| 13 | 11v-04-TricapCubeA–fe3–2co-pbe0 | -1743.9624490 | 33.95 |
| 14 | 11v-03-CapPentAnPr–fe1–2co-pbe0 | -1743.9586870 | 36.31 |
| 15 | 11v-01-EdgeCoalscedIh–fe5–2co-pbe0 | -1743.9586770 | 36.31 |
| 16 | 11v-02-PentaCapTriPri–fe2–2co-pbe0 | -1743.9536010 | 39.50 |
| 17 | 11v-03-CapPentAnPr–fe2–2co-i-27-pbe0 | -1743.9534150 | 39.61 |
| 18 | 11v-03-CapPentAnPr–fe2–2co-r-27-pbe0 | -1743.9534070 | 39.62 |
| 19 | 11v-05-TricapCubeB–fe2–2co-r-90-pbe0 | -1743.9511450 | 41.04 |
| 20 | 11v-05-TricapCubeB–fe2–2co-i-90-pbe0 | -1743.9480330 | 42.99 |
| 21 | 11v-05-TricapCubeB–fe3–2co-pbe0 | -1743.9434160 | 45.89 |
| 22 | 11v-02-PentaCapTriPri–fe1–2co-pbe0 | -1743.9038660 | 70.71 |

Table S6E: Energy ranking for the $B_5H_5Fe(CO)_2$ structures at the PBE0/def2-TZVP level of theory.

| No. | Initial structure | Final Energy (a.u.) | Relative Energy (kcal/mol) |
|---|---|---|---|
| 1 | 11v-03-CapPentAnPr–fe3–3co-pbe0 | -1857.2334350 | 0.00 |
| 2 | 11v-01-EdgeCoalscedIh–fe1–3co-pbe0 | -1857.2334120 | 0.01 |
| 3 | 11v-02-PentaCapTriPri–fe1–3co-pbe0 | -1857.2334090 | 0.02 |
| 4 | 11v-04-TricapCubeA–fe2–3co-pbe0 | -1857.2334090 | 0.02 |
| 5 | 11v-04-TricapCubeA–fe1–3co-pbe0 | -1857.2224060 | 6.92 |
| 6 | 11v-04-TricapCubeA–fe4–3co-pbe0 | -1857.2224060 | 6.92 |
| 7 | 11v-01-EdgeCoalscedIh–fe2–3co-pbe0 | -1857.2223840 | 6.93 |
| 8 | 11v-02-PentaCapTriPri–fe2–3co-pbe0 | -1857.2136450 | 12.42 |
| 9 | 11v-05-TricapCubeB–fe2–3co-pbe0 | -1857.2091140 | 15.26 |
| 10 | 11v-03-CapPentAnPr–fe2–3co-i-12-pbe0 | -1857.2079730 | 15.98 |
| 11 | 11v-05-TricapCubeB–fe4–3co-pbe0 | -1857.2079500 | 15.99 |
| 12 | 11v-03-CapPentAnPr–fe2–3co-r-12-pbe0 | -1857.2079460 | 15.99 |
| 13 | 11v-01-EdgeCoalscedIh–fe4–3co-pbe0 | -1857.2079280 | 16.01 |
| 14 | 11v-06-CapPentPrA–fe1–3co-i-17-pbe0 | -1857.2079160 | 16.01 |
| 15 | 11v-06-CapPentPrA–fe1–3co-r-17-pbe0 | -1857.2078700 | 16.04 |
| 16 | 11v-04-TricapCubeA–fe3–3co-pbe0 | -1857.2065960 | 16.84 |
| 17 | 11v-01-EdgeCoalscedIh–fe3–3co-pbe0 | -1857.2041840 | 18.36 |
| 18 | 11v-03-CapPentAnPr–fe1–3co-pbe0 | -1857.1965040 | 23.17 |
| 19 | 11v-01-EdgeCoalscedIh–fe5–3co-pbe0 | -1857.1964780 | 23.19 |
| 20 | 11v-05-TricapCubeB–fe1–3co-pbe0 | -1857.1883240 | 28.31 |
| 21 | 11v-05-TricapCubeB–fe3–3co-pbe0 | -1857.1055180 | 80.27 |

Table S6F: Energy ranking for the $B_5H_5Fe(CO)_2$ structures at the PBE0/def2-TZVP level of theory.

| No. | Initial structure | Final Energy (a.u.) | Relative Energy (kcal/mol) |
|---|---|---|---|
| 1 | 11v-01-EdgeCoalscedIh–fe3–4co-pbe0 | -1970.4077120 | 0.00 |
| 2 | 11v-04-TricapCubeA–fe4–4co-pbe0 | -1970.4076330 | 0.05 |
| 3 | 11v-02-PentaCapTriPri–fe1–4co-pbe0 | -1970.4068480 | 0.54 |
| 4 | 11v-04-TricapCubeA–fe2–4co-pbe0 | -1970.4055060 | 1.38 |
| 5 | 11v-04-TricapCubeA–fe1–4co-pbe0 | -1970.4054280 | 1.43 |
| 6 | 11v-01-EdgeCoalscedIh–fe1–4co-pbe0 | -1970.4041590 | 2.23 |
| 7 | 11v-03-CapPentAnPr–fe3–4co-pbe0 | -1970.3935790 | 8.87 |
| 8 | 11v-04-TricapCubeA–fe3–4co-pbe0 | -1970.3935570 | 8.88 |
| 9 | 11v-05-TricapCubeB–fe3–4co-pbe0 | -1970.3935320 | 8.90 |
| 10 | 11v-05-TricapCubeB–fe4–4co-pbe0 | -1970.3935230 | 8.90 |
| 11 | 11v-01-EdgeCoalscedIh–fe4–4co-pbe0 | -1970.3918840 | 9.93 |
| 12 | 11v-05-TricapCubeB–fe1–4co-pbe0 | -1970.3918180 | 9.97 |
| 13 | 11v-01-EdgeCoalscedIh–fe2–4co-pbe0 | -1970.3918000 | 9.98 |
| 14 | 11v-02-PentaCapTriPri–fe2–4co-i-18-pbe0 | -1970.3846120 | 14.50 |
| 15 | 11v-02-PentaCapTriPri–fe2–4co-r-18-pbe0 | -1970.3846120 | 14.50 |
| 16 | 11v-06-CapPentPrA–fe1–4co-pbe0 | -1970.3786170 | 18.26 |
| 17 | 11v-05-TricapCubeB–fe2–4co-pbe0 | -1970.3785920 | 18.27 |
| 18 | 11v-03-CapPentAnPr–fe1–4co-pbe0 | -1970.3677070 | 25.10 |
| 19 | 11v-03-CapPentAnPr–fe2–4co-pbe0 | -1970.3664840 | 25.87 |
| 20 | 11v-01-EdgeCoalscedIh–fe5–4co-pbe0 | -1970.3506360 | 35.82 |

Table S7A: Distance table for the lowest-lying $B_{11}H_{11}Fe(CO)_2$ optimized structures obtained at the PBE0/def2-TZVP level of theory. Included are the zero-point corrected absolute energy in (a.u.) at the DLPNO-CCSD(T)/def2-QZVP level of theory with zero-point energy obtained from the PBE0/def2-TZVP computations, relative energies in (kcal/mol) and symmetry. For clarity, only the atoms forming the cluster framework are shown.

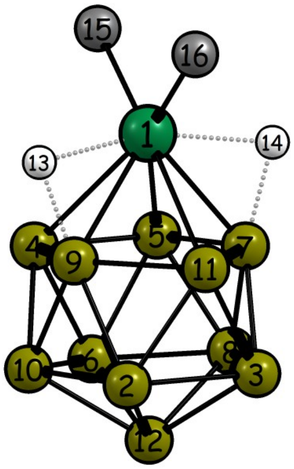


1. -1768.44972536 0.0 $C_s$

| | | 1 | 2 | 3 | 4 | 5 |
|---|---|---|---|---|---|---|
| 1 | Fe | 0.000000 | | | | |
| 2 | B | 3.332704 | 0.000000 | | | |
| 3 | B | 3.332143 | 1.830225 | 0.000000 | | |
| 4 | B | 2.333795 | 3.012033 | 3.500283 | 0.000000 | |
| 5 | B | 2.334063 | 3.501941 | 3.013775 | 1.737597 | 0.000000 |
| 6 | B | 3.509010 | 2.950531 | 2.950113 | 1.769733 | 1.769376 |
| 7 | B | 1.945813 | 2.734080 | 1.745967 | 2.748807 | 1.833064 |
| 8 | B | 3.348236 | 2.919546 | 1.810000 | 2.850165 | 1.773752 |
| 9 | B | 1.946773 | 1.746973 | 2.735192 | 1.831359 | 2.750635 |
| 10 | B | 3.350070 | 1.809252 | 2.917880 | 1.773748 | 2.850685 |
| 11 | B | 2.188199 | 1.778476 | 1.779087 | 3.191185 | 3.193312 |
| 12 | B | 3.928601 | 1.792899 | 1.791145 | 2.897707 | 2.897943 |
| 13 | H | 1.677051 | 3.017822 | 3.890571 | 2.324283 | 3.336495 |
| 14 | H | 1.676046 | 3.890594 | 3.017250 | 3.335265 | 2.324253 |

| | | 6 | 7 | 8 | 9 | 10 |
|---|---|---|---|---|---|---|
| 6 | B | 0.000000 | | | | |
| 7 | B | 2.796918 | 0.000000 | | | |
| 8 | B | 1.792096 | 1.731594 | 0.000000 | | |
| 9 | B | 2.798211 | 2.419416 | 3.152703 | 0.000000 | |
| 10 | B | 1.791607 | 3.151027 | 2.865299 | 1.733606 | 0.000000 |
| 11 | B | 3.585184 | 1.756093 | 2.963765 | 1.755904 | 2.963127 |
| 12 | B | 1.785799 | 2.740516 | 1.797275 | 2.743143 | 1.797342 |
| 13 | H | 3.814318 | 3.132148 | 4.212204 | 1.356233 | 2.882536 |
| 14 | H | 3.812803 | 1.356592 | 2.879158 | 3.132840 | 4.211869 |

| | | 11 | 12 | 13 | 14 |
|---|---|---|---|---|---|
| 11 | B | 0.000000 | | | |
| 12 | B | 2.911317 | 0.000000 | | |
| 13 | H | 2.497837 | 4.075216 | 0.000000 | |
| 14 | H | 2.499152 | 4.073072 | 3.295284 | 0.000000 |

Fe1-C15: 1.803460 Fe1-C16: 1.797361

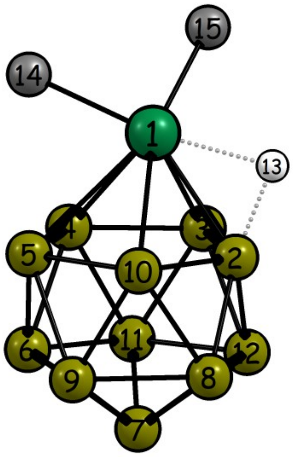


2. -1768.42115520 +17.9 $C_1$

| | | 1 | 2 | 3 | 4 | 5 |
|---|---|---|---|---|---|---|
| 1 | Fe | 0.000000 | | | | |
| 2 | B | 1.955520 | 0.000000 | | | |
| 3 | B | 2.236728 | 1.797087 | 0.000000 | | |
| 4 | B | 2.277714 | 2.698703 | 1.772449 | 0.000000 | |
| 5 | B | 2.278221 | 2.714228 | 3.020893 | 1.886301 | 0.000000 |
| 6 | B | 3.433549 | 3.140498 | 2.888162 | 1.775765 | 1.759016 |
| 7 | B | 3.967032 | 2.701763 | 2.919326 | 2.896758 | 2.886778 |
| 8 | B | 3.326075 | 1.707895 | 3.011699 | 3.428282 | 2.876730 |
| 9 | B | 3.455297 | 2.747071 | 3.525087 | 2.961588 | 1.788457 |
| 10 | B | 2.173049 | 1.768801 | 3.094871 | 2.956003 | 1.745446 |
| 11 | B | 3.456829 | 2.727751 | 1.772412 | 1.780740 | 2.936429 |
| 12 | B | 3.338835 | 1.693291 | 1.815684 | 2.883509 | 3.425171 |
| 13 | H | 1.648249 | 1.328103 | 2.361509 | 3.345309 | 3.387870 |

| | | 6 | 7 | 8 | 9 | 10 |
|---|---|---|---|---|---|---|
| 6 | B | 0.000000 | | | | |
| 7 | B | 1.795648 | 0.000000 | | | |
| 8 | B | 2.881845 | 1.793527 | 0.000000 | | |
| 9 | B | 1.790099 | 1.786446 | 1.792221 | 0.000000 | |
| 10 | B | 2.836338 | 2.873382 | 1.779494 | 1.756729 | 0.000000 |
| 11 | B | 1.777170 | 1.785023 | 2.929840 | 2.922237 | 3.447993 |
| 12 | B | 2.882133 | 1.786508 | 1.827703 | 2.926301 | 2.950538 |
| 13 | H | 4.216071 | 4.021486 | 2.893931 | 3.844979 | 2.402690 |

| | | 11 | 12 | 13 |
|---|---|---|---|---|
| 11 | B | 0.000000 | | |
| 12 | B | 1.803768 | 0.000000 | |
| 13 | H | 3.797020 | 2.861093 | 0.000000 |

Fe1-C14: 1.807438 Fe1-C15: 1.798841

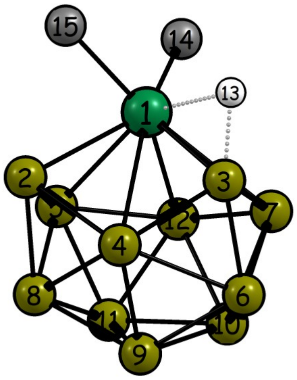


3. -1768.42078308 +18.2 $C_1$

| | | 1 | 2 | 3 | 4 | 5 |
|---|---|---|---|---|---|---|
| 1 | Fe | 0.000000 | | | | |
| 2 | B | 2.007678 | 0.000000 | | | |
| 3 | B | 1.818183 | 2.897410 | 0.000000 | | |
| 4 | B | 2.345553 | 1.876486 | 1.768505 | 0.000000 | |
| 5 | B | 2.217531 | 1.760603 | 3.320958 | 2.751460 | 0.000000 |
| 6 | B | 3.077672 | 3.557336 | 1.660704 | 1.951359 | 3.621034 |
| 7 | B | 2.317223 | 3.651099 | 1.723416 | 2.847261 | 3.157122 |
| 8 | B | 3.049577 | 1.719293 | 3.308372 | 1.892959 | 1.812198 |
| 9 | B | 3.461431 | 3.000267 | 2.833093 | 1.852729 | 2.878360 |
| 10 | B | 3.336110 | 3.780507 | 2.794994 | 2.886753 | 2.979477 |
| 11 | B | 3.277754 | 2.925252 | 3.497312 | 2.836415 | 1.785064 |
| 12 | B | 2.257031 | 3.045746 | 2.806074 | 3.074551 | 1.787128 |
| 13 | H | 1.787764 | 3.272312 | 1.272662 | 2.681848 | 3.881947 |

| | | 6 | 7 | 8 | 9 | 10 |
|---|---|---|---|---|---|---|
| 6 | B | 0.000000 | | | | |
| 7 | B | 1.842456 | 0.000000 | | | |
| 8 | B | 3.091681 | 3.615879 | 0.000000 | | |
| 9 | B | 1.777983 | 2.878610 | 1.750830 | 0.000000 | |
| 10 | B | 1.796063 | 1.765049 | 2.922551 | 1.778153 | 0.000000 |
| 11 | B | 2.931886 | 2.915129 | 1.752291 | 1.773859 | 1.754088 |
| 12 | B | 2.876800 | 1.762385 | 2.848459 | 2.798237 | 1.820515 |
| 13 | H | 2.910527 | 2.503712 | 4.193282 | 4.044304 | 3.925353 |

| | | 11 | 12 | 13 |
|---|---|---|---|---|
| 11 | B | 0.000000 | | |
| 12 | B | 1.777626 | 0.000000 | |
| 13 | H | 4.496515 | 3.496738 | 0.000000 |

Fe1-C14: 1.801807 Fe1-C15: 1.793661

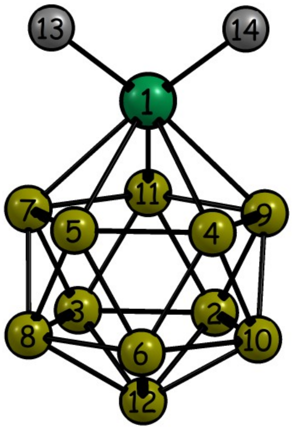


4. -1768.39476586 +34.5 $C_s$

| | | 1 | 2 | 3 | 4 | 5 |
|---|---|---|---|---|---|---|
| 1 | Fe | 0.000000 | | | | |
| 2 | B | 3.271634 | 0.000000 | | | |
| 3 | B | 3.277368 | 1.762159 | 0.000000 | | |
| 4 | B | 2.214879 | 2.957594 | 3.446705 | 0.000000 | |
| 5 | B | 2.220424 | 3.440402 | 2.956302 | 1.767451 | 0.000000 |
| 6 | B | 3.400032 | 2.882011 | 2.885285 | 1.785585 | 1.784184 |
| 7 | B | 2.146415 | 2.932254 | 1.805247 | 2.945820 | 1.817544 |
| 8 | B | 3.373469 | 2.895643 | 1.791122 | 2.938346 | 1.848547 |
| 9 | B | 2.139301 | 1.803332 | 2.935128 | 1.818417 | 2.943897 |
| 10 | B | 3.367747 | 1.791725 | 2.898514 | 1.850615 | 2.933749 |
| 11 | B | 1.902886 | 1.767444 | 1.765669 | 2.834593 | 2.830033 |
| 12 | B | 3.897009 | 1.781177 | 1.782842 | 2.920967 | 2.917100 |

| | | 6 | 7 | 8 | 9 | 10 |
|---|---|---|---|---|---|---|
| 6 | B | 0.000000 | | | | |
| 7 | B | 2.859075 | 0.000000 | | | |
| 8 | B | 1.812123 | 1.716179 | 0.000000 | | |
| 9 | B | 2.855865 | 3.036666 | 3.446241 | 0.000000 | |
| 10 | B | 1.807226 | 3.446126 | 2.941773 | 1.714858 | 0.000000 |
| 11 | B | 3.283704 | 1.808468 | 2.816149 | 1.816302 | 2.820811 |
| 12 | B | 1.771266 | 2.873373 | 1.795265 | 2.871784 | 1.794541 |

| | | 11 | 12 |
|---|---|---|---|
| 11 | B | 0.000000 | |
| 12 | B | 2.825205 | 0.000000 |

Fe1-C13: 1.820349 Fe1-C14: 1.820029

Table S7B: Distance table for the lowest-lying $B_{11}H_{11}Fe(CO)_3$ optimized structures obtained at the PBE0/def2-TZVP level of theory. Included are the zero-point corrected absolute energy in (a.u.) at the DLPNO-CCSD(T)/def2-QZVP level of theory with zero-point energy obtained from the PBE0/def2-TZVP computations, relative energies in (kcal/mol) and symmetry. For clarity, only the atoms forming the cluster framework are shown.

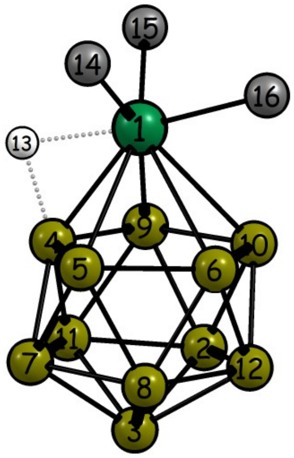


1. -1881.63878635 0.0 $C_s$

| | | 1 | 2 | 3 | 4 | 5 |
|---|---|---|---|---|---|---|
| 1 | Fe | 0.000000 | | | | |
| 2 | B | 3.511346 | 0.000000 | | | |
| 3 | B | 4.022750 | 1.781377 | 0.000000 | | |
| 4 | B | 1.965319 | 2.720314 | 2.694755 | 0.000000 | |
| 5 | B | 2.285950 | 3.524601 | 2.910075 | 1.787258 | 0.000000 |
| 6 | B | 2.303372 | 2.965330 | 2.892518 | 2.649530 | 1.765472 |
| 7 | B | 3.383299 | 2.924205 | 1.789243 | 1.708982 | 1.793831 |
| 8 | B | 3.512692 | 2.923138 | 1.781254 | 2.720453 | 1.771481 |
| 9 | B | 2.285982 | 1.770205 | 2.910524 | 1.789770 | 3.178748 |
| 10 | B | 2.302692 | 1.784001 | 2.894407 | 2.650843 | 3.037906 |
| 11 | B | 3.382558 | 1.798870 | 1.789375 | 1.709236 | 2.998993 |
| 12 | B | 3.457070 | 1.781898 | 1.801621 | 3.091002 | 2.875623 |
| 13 | H | 1.620067 | 3.833807 | 4.044948 | 1.356426 | 2.410637 |

| | | 6 | 7 | 8 | 9 | 10 |
|---|---|---|---|---|---|---|
| 6 | B | 0.000000 | | | | |
| 7 | B | 2.865147 | 0.000000 | | | |
| 8 | B | 1.783606 | 1.799362 | 0.000000 | | |
| 9 | B | 3.037040 | 3.000433 | 3.526017 | 0.000000 | |
| 10 | B | 1.922217 | 3.422456 | 2.967918 | 1.764968 | 0.000000 |
| 11 | B | 3.420470 | 1.818272 | 2.924514 | 1.794178 | 2.865926 |
| 12 | B | 1.765299 | 2.875534 | 1.783059 | 2.874929 | 1.766527 |
| 13 | H | 3.335120 | 2.909437 | 3.837892 | 2.406356 | 3.332863 |

| | | 11 | 12 | 13 |
|---|---|---|---|---|
| 11 | B | 0.000000 | | |
| 12 | B | 2.874979 | 0.000000 | |
| 13 | H | 2.906398 | 4.196125 | 0.000000 |

Fe1-C14: 1.797287 Fe1-C15: 1.797080 Fe1-C16: 1.796670

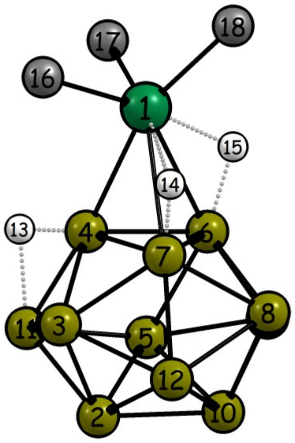


2. -1881.60715681 +19.8 $C_1$

| | | 1 | 2 | 3 | 4 | 5 |
|---|---|---|---|---|---|---|
| 1 | Fe | 0.000000 | | | | |
| 2 | B | 4.461499 | 0.000000 | | | |
| 3 | B | 3.354901 | 1.776918 | 0.000000 | | |
| 4 | B | 2.047960 | 2.630442 | 1.817338 | 0.000000 | |
| 5 | B | 3.766003 | 1.893536 | 2.742399 | 2.136430 | 0.000000 |
| 6 | B | 2.182300 | 3.075609 | 2.897323 | 1.746336 | 1.966176 |
| 7 | B | 2.211758 | 2.730600 | 1.772926 | 1.726146 | 2.800982 |
| 8 | B | 3.387722 | 2.900741 | 2.989026 | 2.924603 | 2.673340 |
| 9 | B | 3.821681 | 3.001192 | 3.681864 | 3.026618 | 1.757594 |
| 10 | B | 4.521855 | 1.791554 | 2.942746 | 3.216973 | 1.881538 |
| 11 | B | 3.746046 | 1.740151 | 1.803833 | 1.702899 | 1.848801 |
| 12 | B | 3.954596 | 1.782905 | 1.779625 | 2.822888 | 2.796594 |
| 13 | H | 2.883215 | 3.032928 | 2.504726 | 1.262232 | 2.406791 |
| 14 | H | 1.823555 | 3.947024 | 2.661729 | 2.457689 | 3.962651 |
| 15 | H | 1.776241 | 4.323683 | 3.911771 | 2.458792 | 3.062232 |

| | | 6 | 7 | 8 | 9 | 10 |
|---|---|---|---|---|---|---|
| 6 | B | 0.000000 | | | | |
| 7 | B | 1.751562 | 0.000000 | | | |
| 8 | B | 1.797533 | 1.763435 | 0.000000 | | |
| 9 | B | 1.648133 | 2.867521 | 1.773441 | 0.000000 | |
| 10 | B | 2.577239 | 2.766389 | 1.791586 | 1.717965 | 0.000000 |
| 11 | B | 2.916220 | 2.866677 | 3.675075 | 3.457140 | 3.059279 |
| 12 | B | 2.793507 | 1.764291 | 1.806437 | 2.953575 | 1.816991 |
| 13 | H | 2.712018 | 2.970874 | 4.046961 | 3.744813 | 3.945408 |
| 14 | H | 2.518921 | 1.264444 | 2.625168 | 3.856999 | 3.968130 |
| 15 | H | 1.273322 | 2.564062 | 2.720798 | 2.529241 | 3.786828 |

| | | 11 | 12 | 13 | 14 | 15 |
|---|---|---|---|---|---|---|
| 11 | B | 0.000000 | | | | |
| 12 | B | 2.961204 | 0.000000 | | | |
| 13 | H | 1.439418 | 3.798630 | 0.000000 | | |
| 14 | H | 3.873686 | 2.817902 | 3.639880 | 0.000000 | |
| 15 | H | 3.900447 | 3.939833 | 3.261296 | 2.769810 | 0.000000 |

Fe1-C16: 1.774525 Fe1-C17: 1.765344 Fe1-C18: 1.811493

Table S7C: Distance table for the lowest-lying $B_{11}H_{11}Fe(CO)_4$ optimized structures obtained at the PBE0/def2-TZVP level of theory. Included are the zero-point corrected absolute energy in (a.u.) at the DLPNO-CCSD(T)/def2-QZVP level of theory with zero-point energy obtained from the PBE0/def2-TZVP computations, relative energies in (kcal/mol) and symmetry. For clarity, only the atoms forming the cluster framework are shown.

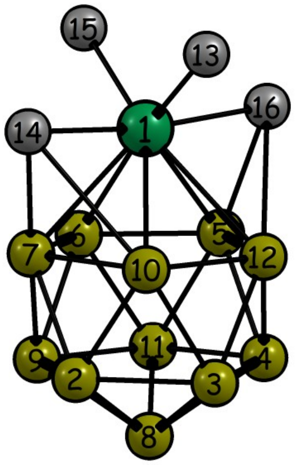


1. -1994.77981793 0.0 $C_s$

| | | 1 | 2 | 3 | 4 | 5 |
|---|---|---|---|---|---|---|
| 1 | Fe | 0.000000 | | | | |
| 2 | B | 3.399425 | 0.000000 | | | |
| 3 | B | 3.465674 | 1.775343 | 0.000000 | | |
| 4 | B | 3.396961 | 2.879662 | 1.773791 | 0.000000 | |
| 5 | B | 2.162438 | 3.397331 | 2.933170 | 1.789936 | 0.000000 |
| 6 | B | 2.209861 | 2.930386 | 3.478638 | 2.932629 | 1.819720 |
| 7 | B | 2.169639 | 1.792749 | 2.939506 | 3.403051 | 2.900481 |
| 8 | B | 3.972346 | 1.788222 | 1.759132 | 1.786354 | 2.872965 |
| 9 | B | 3.402833 | 1.819523 | 2.909033 | 2.903200 | 2.881386 |
| 10 | B | 2.258495 | 1.766844 | 1.779771 | 2.876187 | 2.963824 |
| 11 | B | 3.401587 | 2.903953 | 2.910716 | 1.822440 | 1.777714 |
| 12 | B | 2.257551 | 2.876636 | 1.778436 | 1.765309 | 1.898252 |

| | | 6 | 7 | 8 | 9 | 10 |
|---|---|---|---|---|---|---|
| 6 | B | 0.000000 | | | | |
| 7 | B | 1.818033 | 0.000000 | | | |
| 8 | B | 2.891713 | 2.878170 | 0.000000 | | |
| 9 | B | 1.770121 | 1.777707 | 1.784824 | 0.000000 | |
| 10 | B | 3.048752 | 1.903480 | 2.881703 | 2.959855 | 0.000000 |
| 11 | B | 1.770938 | 2.884721 | 1.785268 | 1.776177 | 3.456640 |
| 12 | B | 3.050389 | 2.970910 | 2.880304 | 3.455463 | 1.790043 |

| | | 11 | 12 |
|---|---|---|---|
| 11 | B | 0.000000 | |
| 12 | B | 2.961248 | 0.000000 |

Fe1-C13: 1.795185 Fe1-C14: 1.842175
Fe1-C15: 1.793481 Fe1-C16: 1.848313

Table S7D: Energy ranking for the $B_5H_5Fe(CO)_2$ structures at the PBE0/def2-TZVP level of theory.

| No. | Initial structure | Final Energy (a.u.) | Relative Energy (kcal/mol) |
|---|---|---|---|
| 1 | 12v-09-2v6-Dh–fe2–2co-pbe0 | -1769.4568560 | 0.00 |
| 2 | 12v-01-Icos–fe1–2co-pbe0 | -1769.4568270 | 0.02 |
| 3 | 12v-09-2v6-Dh–fe3–2co-pbe0 | -1769.4568220 | 0.02 |
| 4 | 12v-08-TetraCappedCube-C2v–fe2–2co-pbe0 | -1769.4567600 | 0.06 |
| 5 | 12v-09-2v6-Dh–fe1–2co-pbe0 | -1769.4567560 | 0.06 |
| 6 | 12v-08-TetraCappedCube-C2v–fe1–2co-pbe0 | -1769.4370780 | 12.41 |
| 7 | 12v-06-DsqAnPr–fe2–2co-pbe0 | -1769.4263030 | 19.17 |
| 8 | 12v-07-TetraCappedCube-D4h–fe2–2co-pbe0 | -1769.4262980 | 19.18 |
| 9 | 12v-02-CubeOh–fe2–2co-pbe0 | -1769.4262890 | 19.18 |
| 10 | 12v-08-TetraCappedCube-C2v–fe4–2co-pbe0 | -1769.4262700 | 19.19 |
| 11 | 12v-06-DsqAnPr–fe1–2co-pbe0 | -1769.4262640 | 19.20 |
| 12 | 12v-05-HexAnPr–fe–2co-pbe0 | -1769.4227720 | 21.39 |
| 13 | 12v-04-HexPr–fe–2co-pbe0 | -1769.4116020 | 28.40 |
| 14 | 12v-03-AnticubeOh–fe2–2co-pbe0 | -1769.3857950 | 44.59 |
| 15 | 12v-02-CubeOh–fe1–2co-i-26-pbe0 | -1769.3832260 | 46.20 |
| 16 | 12v-07-TetraCappedCube-D4h–fe1–2co-i-35-pbe0 | -1769.3831720 | 46.24 |
| 17 | 12v-07-TetraCappedCube-D4h–fe1–2co-r-35-pbe0 | -1769.3831710 | 46.24 |
| 18 | 12v-02-CubeOh–fe1–2co-r-26-pbe0 | -1769.3830960 | 46.29 |
| 19 | 12v-08-TetraCappedCube-C2v–fe3–2co-pbe0 | -1769.3574350 | 62.39 |
| 20 | 12v-03-AnticubeOh–fe1–2co-pbe0 | -1769.3379980 | 74.58 |

Table S7E: Energy ranking for the $B_5H_5Fe(CO)_2$ structures at the PBE0/def2-TZVP level of theory.

| No. | Initial structure | Final Energy (a.u.) | Relative Energy (kcal/mol) |
|---|---|---|---|
| 1 | 12v-09-2v6-Dh–fe1–3co-pbe0 | -1882.6699030 | 0.00 |
| 2 | 12v-09-2v6-Dh–fe2–3co-pbe0 | -1882.6698730 | 0.02 |
| 3 | 12v-08-TetraCappedCube-C2v–fe1–3co-pbe0 | -1882.6698710 | 0.02 |
| 4 | 12v-01-Icos–fe1–3co-pbe0 | -1882.6698630 | 0.03 |
| 5 | 12v-08-TetraCappedCube-C2v–fe2–3co-pbe0 | -1882.6467150 | 14.55 |
| 6 | 12v-05-HexAnPr–fe–3co-pbe0 | -1882.6456880 | 15.20 |
| 7 | 12v-06-DsqAnPr–fe2–3co-pbe0 | -1882.6422260 | 17.37 |
| 8 | 12v-07-TetraCappedCube-D4h–fe2–3co-pbe0 | -1882.6421970 | 17.39 |
| 9 | 12v-08-TetraCappedCube-C2v–fe4–3co-pbe0 | -1882.6421930 | 17.39 |
| 10 | 12v-09-2v6-Dh–fe3–3co-pbe0 | -1882.6421670 | 17.40 |
| 11 | 12v-06-DsqAnPr–fe1–3co-pbe0 | -1882.6421520 | 17.41 |
| 12 | 12v-04-HexPr–fe–3co-pbe0 | -1882.6421500 | 17.42 |
| 13 | 12v-02-CubeOh–fe2–3co-pbe0 | -1882.6421450 | 17.42 |
| 14 | 12v-08-TetraCappedCube-C2v–fe3–3co-pbe0 | -1882.6421290 | 17.43 |
| 15 | 12v-02-CubeOh–fe1–3co-pbe0 | -1882.6259010 | 27.61 |
| 16 | 12v-07-TetraCappedCube-D4h–fe1–3co-pbe0 | -1882.6258620 | 27.64 |
| 17 | 12v-03-AnticubeOh–fe1–3co-pbe0 | -1882.6234660 | 29.14 |
| 18 | 12v-03-AnticubeOh–fe2–3co-pbe0 | -1882.5922590 | 48.72 |

Table S7F: Energy ranking for the $B_5H_5Fe(CO)_2$ structures at the PBE0/def2-TZVP level of theory.

| No. | Initial structure | Final Energy (a.u.) | Relative Energy (kcal/mol) |
|---|---|---|---|
| 1 | 12v-01-Icos–fe1–4co-pbe0 | -1995.8513810 | 0.00 |
| 2 | 12v-09-2v6-Dh–fe3–4co-pbe0 | -1995.8513580 | 0.01 |
| 3 | 12v-09-2v6-Dh–fe2–4co-pbe0 | -1995.8513490 | 0.02 |
| 4 | 12v-06-DsqAnPr–fe2–4co-pbe0 | -1995.8513460 | 0.02 |
| 5 | 12v-09-2v6-Dh–fe1–4co-pbe0 | -1995.8513290 | 0.03 |
| 6 | 12v-08-TetraCappedCube-C2v–fe4–4co-pbe0 | -1995.8513160 | 0.04 |
| 7 | 12v-05-HexAnPr–fe–4co-pbe0 | -1995.8408610 | 6.60 |
| 8 | 12v-08-TetraCappedCube-C2v–fe3–4co-pbe0 | -1995.8131140 | 24.01 |
| 9 | 12v-07-TetraCappedCube-D4h–fe2–4co-pbe0 | -1995.8128240 | 24.19 |
| 10 | 12v-06-DsqAnPr–fe1–4co-pbe0 | -1995.8128020 | 24.21 |
| 11 | 12v-02-CubeOh–fe2–4co-pbe0 | -1995.8126570 | 24.30 |
| 12 | 12v-04-HexPr–fe–4co-pbe0 | -1995.8020070 | 30.98 |
| 13 | 12v-03-AnticubeOh–fe1–4co-pbe0 | -1995.7943180 | 35.81 |
| 14 | 12v-08-TetraCappedCube-C2v–fe1–4co-pbe0 | -1995.7815710 | 43.81 |
| 15 | 12v-03-AnticubeOh–fe2–4co-pbe0 | -1995.7804230 | 44.53 |
| 16 | 12v-08-TetraCappedCube-C2v–fe2–4co-pbe0 | -1995.7474650 | 65.21 |
| 17 | 12v-02-CubeOh–fe1–4co-pbe0 | -1995.7098500 | 88.81 |
| 18 | 12v-07-TetraCappedCube-D4h–fe1–4co-pbe0 | -1995.7098180 | 88.83 |

Table S8A: Distance table for the lowest-lying $B_{12}H_{12}Fe(CO)_2$ optimized structures obtained at the PBE0/def2-TZVP level of theory. Included are the zero-point corrected absolute energy in (a.u.) at the DLPNO-CCSD(T)/def2-QZVP level of theory with zero-point energy obtained from the PBE0/def2-TZVP computations, relative energies in (kcal/mol) and symmetry. For clarity, only the atoms forming the cluster framework are shown.

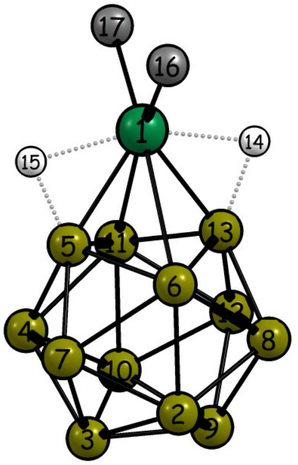


1. -1793.86805124 0.0 $C_{2v}$

| | | 1 | 2 | 3 | 4 | 5 |
|---|---|---|---|---|---|---|
| 1 | Fe | 0.000000 | | | | |
| 2 | B | 4.003526 | 0.000000 | | | |
| 3 | B | 4.420703 | 1.790076 | 0.000000 | | |
| 4 | B | 3.408234 | 2.885731 | 1.767762 | 0.000000 | |
| 5 | B | 1.897190 | 2.845701 | 2.792599 | 1.696040 | 0.000000 |
| 6 | B | 2.375705 | 1.832424 | 2.947002 | 2.941793 | 1.836376 |
| 7 | B | 3.406022 | 1.782733 | 1.767057 | 1.798196 | 1.696025 |
| 8 | B | 3.408017 | 1.783380 | 2.918004 | 3.490311 | 3.080702 |
| 9 | B | 4.421717 | 1.789911 | 1.800968 | 2.917372 | 3.432362 |
| 10 | B | 4.004976 | 2.861064 | 1.789415 | 1.781715 | 2.843935 |
| 11 | B | 2.379586 | 3.389163 | 2.947989 | 1.884194 | 1.835816 |
| 12 | B | 3.409992 | 2.884679 | 2.917018 | 2.990561 | 3.080110 |
| 13 | B | 1.898807 | 2.846250 | 3.433369 | 3.081178 | 2.211077 |
| 14 | H | 1.654419 | 4.059281 | 4.748825 | 4.172532 | 2.996541 |
| 15 | H | 1.652238 | 4.058288 | 4.086107 | 2.774530 | 1.341162 |

| | | 6 | 7 | 8 | 9 | 10 |
|---|---|---|---|---|---|---|
| 6 | B | 0.000000 | | | | |
| 7 | B | 1.883709 | 0.000000 | | | |
| 8 | B | 1.881921 | 2.990387 | 0.000000 | | |
| 9 | B | 2.946321 | 2.916588 | 1.768492 | 0.000000 | |
| 10 | B | 3.386992 | 2.883524 | 2.885614 | 1.790116 | 0.000000 |
| 11 | B | 2.838522 | 2.941570 | 2.941720 | 2.947619 | 1.832478 |
| 12 | B | 2.939725 | 3.488503 | 1.797929 | 1.767276 | 1.783176 |
| 13 | B | 1.834679 | 3.080383 | 1.696771 | 2.793987 | 2.845795 |
| 14 | H | 2.685422 | 4.171951 | 2.774039 | 4.087049 | 4.058735 |
| 15 | H | 2.686045 | 2.772617 | 4.172621 | 4.748960 | 4.058919 |

| | | 11 | 12 | 13 | 14 | 15 |
|---|---|---|---|---|---|---|
| 11 | B | 0.000000 | | | | |
| 12 | B | 1.883389 | 0.000000 | | | |
| 13 | B | 1.835718 | 1.696897 | 0.000000 | | |
| 14 | H | 2.685694 | 2.774432 | 1.340281 | 0.000000 | |
| 15 | H | 2.688497 | 4.173708 | 2.997441 | 3.249288 | 0.000000 |

Fe1-C16: 1.794222 Fe1-C17: 1.794391

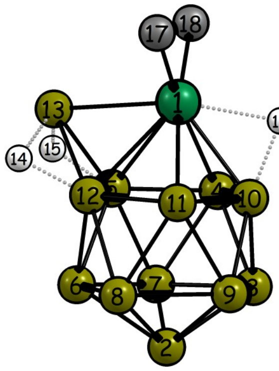

2. -1793.86443931 +2.3 $C_s$

| | | 1 | 2 | 3 | 4 | 5 |
|---|---|---|---|---|---|---|
| 1 | Fe | 0.000000 | | | | |
| 2 | B | 3.930898 | 0.000000 | | | |
| 3 | B | 3.303053 | 1.790256 | 0.000000 | | |
| 4 | B | 2.209028 | 2.929144 | 1.779744 | 0.000000 | |
| 5 | B | 2.131799 | 2.846221 | 2.786280 | 1.741472 | 0.000000 |
| 6 | B | 3.383840 | 1.767782 | 2.850408 | 2.910247 | 1.780513 |
| 7 | B | 3.422838 | 1.797720 | 1.799808 | 1.804913 | 1.773946 |
| 8 | B | 3.421541 | 1.797270 | 2.938114 | 3.541854 | 2.894927 |
| 9 | B | 3.303026 | 1.790313 | 1.833353 | 2.992790 | 3.320973 |
| 10 | B | 1.920862 | 2.759207 | 1.740956 | 1.793148 | 2.606991 |
| 11 | B | 2.207606 | 2.928283 | 2.992281 | 3.156459 | 2.940091 |
| 12 | B | 2.131345 | 2.845884 | 3.320360 | 2.941037 | 1.779218 |
| 13 | B | 2.038623 | 4.214524 | 4.296804 | 3.148268 | 1.758098 |
| 14 | H | 2.797671 | 3.959764 | 4.561283 | 3.958206 | 2.490877 |
| 15 | H | 2.796984 | 3.961097 | 4.010783 | 2.744731 | 1.273313 |
| 16 | H | 1.695294 | 4.092047 | 2.936132 | 2.414828 | 3.298015 |

| | | 6 | 7 | 8 | 9 | 10 |
|---|---|---|---|---|---|---|
| 6 | B | 0.000000 | | | | |
| 7 | B | 1.772607 | 0.000000 | | | |
| 8 | B | 1.773130 | 2.942745 | 0.000000 | | |
| 9 | B | 2.851233 | 2.938882 | 1.800015 | 0.000000 | |
| 10 | B | 3.167998 | 2.790454 | 2.790116 | 1.741387 | 0.000000 |
| 11 | B | 2.909607 | 3.541633 | 1.803573 | 1.780238 | 1.793698 |
| 12 | B | 1.780671 | 2.895254 | 1.773315 | 2.786832 | 2.607217 |
| 13 | B | 2.835403 | 3.506897 | 3.506246 | 4.297281 | 3.490312 |
| 14 | H | 2.547089 | 3.876412 | 2.728147 | 4.010855 | 3.789665 |
| 15 | H | 2.548091 | 2.729731 | 3.876661 | 4.562167 | 3.789116 |
| 16 | H | 4.268176 | 3.890790 | 3.891614 | 2.937426 | 1.340449 |

| | | 11 | 12 | 13 | 14 | 15 |
|---|---|---|---|---|---|---|
| 11 | B | 0.000000 | | | | |
| 12 | B | 1.740417 | 0.000000 | | | |
| 13 | B | 3.147112 | 1.757977 | 0.000000 | | |
| 14 | H | 2.743825 | 1.273041 | 1.504568 | 0.000000 | |
| 15 | H | 3.956846 | 2.490768 | 1.503501 | 2.575973 | 0.000000 |
| 16 | H | 2.417752 | 3.299196 | 3.708533 | 4.245222 | 4.243007 |

| | | 16 |
|---|---|---|
| 16 | H | 0.000000 |

Fe1-C17: 1.776306 Fe1-C18: 1.776243

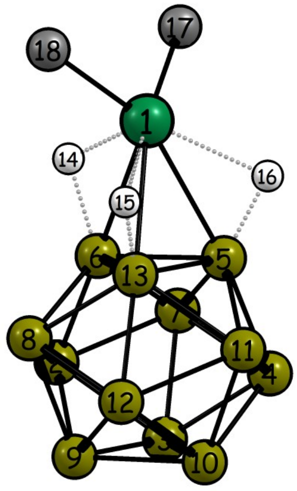


3. -1793.85151592 +10.4 $C_s$

| | | 1 | 2 | 3 | 4 | 5 |
|---|---|---|---|---|---|---|
| 1 | Fe | 0.000000 | | | | |
| 2 | B | 3.867280 | 0.000000 | | | |
| 3 | B | 4.639129 | 1.792582 | 0.000000 | | |
| 4 | B | 3.929563 | 2.944726 | 1.801502 | 0.000000 | |
| 5 | B | 2.189628 | 2.889673 | 2.829800 | 1.748260 | 0.000000 |
| 6 | B | 2.149380 | 1.718094 | 2.756148 | 2.810056 | 1.739386 |
| 7 | B | 3.322786 | 1.814385 | 1.772373 | 1.808089 | 1.789286 |
| 8 | B | 3.322453 | 1.813515 | 2.900953 | 3.455276 | 2.891313 |
| 9 | B | 4.640454 | 1.792843 | 1.794475 | 2.922675 | 3.341431 |
| 10 | B | 4.652147 | 2.904545 | 1.793713 | 1.789404 | 2.797047 |
| 11 | B | 3.404668 | 3.467725 | 2.916149 | 1.822529 | 1.768002 |
| 12 | B | 3.931014 | 2.943783 | 2.921710 | 2.950648 | 2.871008 |
| 13 | B | 2.190483 | 2.890466 | 3.341340 | 2.870724 | 1.756896 |
| 14 | H | 1.582519 | 2.820147 | 4.055218 | 3.962000 | 2.531574 |
| 15 | H | 1.863976 | 3.959697 | 4.581860 | 3.958657 | 2.553950 |
| 16 | H | 1.869921 | 3.960086 | 3.988652 | 2.706330 | 1.251409 |

| | | 6 | 7 | 8 | 9 | 10 |
|---|---|---|---|---|---|---|
| 6 | B | 0.000000 | | | | |
| 7 | B | 1.738195 | 0.000000 | | | |
| 8 | B | 1.738054 | 2.937904 | 0.000000 | | |
| 9 | B | 2.757476 | 2.901331 | 1.774249 | 0.000000 | |
| 10 | B | 3.240275 | 2.885397 | 2.885842 | 1.793492 | 0.000000 |
| 11 | B | 2.833924 | 2.948042 | 2.946579 | 2.915704 | 1.764317 |
| 12 | B | 2.810806 | 3.455244 | 1.808596 | 1.800910 | 1.788664 |
| 13 | B | 1.740968 | 2.892737 | 1.789663 | 2.831348 | 2.797115 |
| 14 | H | 1.370752 | 2.718505 | 2.704691 | 4.051083 | 4.579112 |
| 15 | H | 2.553712 | 3.920460 | 2.661760 | 3.992899 | 3.971584 |
| 16 | H | 2.555516 | 2.660468 | 3.921230 | 4.581269 | 3.967678 |

| | | 11 | 12 | 13 | 14 | 15 |
|---|---|---|---|---|---|---|
| 11 | B | 0.000000 | | | | |
| 12 | B | 1.820741 | 0.000000 | | | |
| 13 | B | 1.766076 | 1.749105 | 0.000000 | | |
| 14 | H | 3.891246 | 3.953738 | 2.524317 | 0.000000 | |
| 15 | H | 2.664838 | 2.713048 | 1.253110 | 2.719377 | 0.000000 |
| 16 | H | 2.662856 | 3.958382 | 2.555471 | 2.736957 | 2.822255 |

| | | 16 |
|---|---|---|
| 16 | H | 0.000000 |

Fe1-C17: 1.793420 Fe1-C18: 1.792968

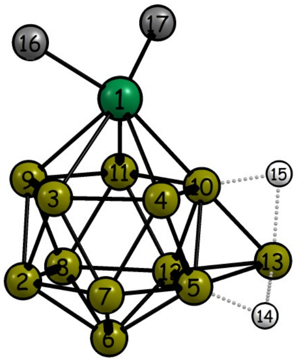


4. -1793.81374397 +34.1 $C_1$

| | | 1 | 2 | 3 | 4 | 5 |
|---|---|---|---|---|---|---|
| 1 | Fe | 0.000000 | | | | |
| 2 | B | 3.368794 | 0.000000 | | | |
| 3 | B | 2.175980 | 1.779765 | 0.000000 | | |
| 4 | B | 2.065076 | 2.836141 | 1.747832 | 0.000000 | |
| 5 | B | 3.294974 | 2.797832 | 2.833190 | 1.768525 | 0.000000 |
| 6 | B | 3.947915 | 1.760793 | 2.902782 | 2.899066 | 1.763922 |
| 7 | B | 3.393274 | 1.788229 | 1.798345 | 1.798346 | 1.741824 |
| 8 | B | 3.357392 | 1.793882 | 2.916561 | 3.367819 | 2.840196 |
| 9 | B | 2.132279 | 1.817498 | 1.833480 | 2.850807 | 3.346439 |
| 10 | B | 2.039002 | 3.346024 | 2.885174 | 1.785206 | 1.797369 |
| 11 | B | 2.126552 | 2.927224 | 2.973444 | 2.925487 | 2.926212 |
| 12 | B | 3.346882 | 2.875731 | 3.432110 | 2.905470 | 1.797910 |
| 13 | B | 3.753173 | 4.185252 | 4.179594 | 2.869753 | 1.721646 |
| 14 | H | 4.263470 | 4.000697 | 3.995683 | 2.642169 | 1.259540 |
| 15 | H | 2.966808 | 4.584123 | 4.080189 | 2.671042 | 2.469277 |

| | | 6 | 7 | 8 | 9 | 10 |
|---|---|---|---|---|---|---|
| 6 | B | 0.000000 | | | | |
| 7 | B | 1.806924 | 0.000000 | | | |
| 8 | B | 1.798608 | 2.929342 | 0.000000 | | |
| 9 | B | 2.916709 | 2.947686 | 1.794541 | 0.000000 | |
| 10 | B | 2.903648 | 2.913257 | 2.844265 | 2.834570 | 0.000000 |
| 11 | B | 2.968128 | 3.503005 | 1.800026 | 1.785159 | 1.792682 |
| 12 | B | 1.813963 | 2.935246 | 1.766958 | 2.891809 | 1.793125 |
| 13 | B | 2.978361 | 3.447483 | 3.516297 | 4.210031 | 1.718847 |
| 14 | H | 2.709139 | 2.736473 | 3.940846 | 4.581892 | 2.515498 |
| 15 | H | 3.861565 | 3.919137 | 3.954828 | 4.090010 | 1.288460 |

| | | 11 | 12 | 13 | 14 | 15 |
|---|---|---|---|---|---|---|
| 11 | B | 0.000000 | | | | |
| 12 | B | 1.833579 | 0.000000 | | | |
| 13 | B | 3.012219 | 1.806589 | 0.000000 | | |
| 14 | H | 3.921169 | 2.553032 | 1.527685 | 0.000000 | |
| 15 | H | 2.792216 | 2.525578 | 1.435082 | 2.554076 | 0.000000 |

Fe1-C16: 1.781898 Fe1-C17: 1.783051

Table S8B: Distance table for the lowest-lying $B_{12}H_{12}Fe(CO)_3$ optimized structures obtained at the PBE0/def2-TZVP level of theory. Included are the zero-point corrected absolute energy in (a.u.) at the DLPNO-CCSD(T)/def2-QZVP level of theory with zero-point energy obtained from the PBE0/def2-TZVP computations, relative energies in (kcal/mol) and symmetry. For clarity, only the atoms forming the cluster framework are shown.

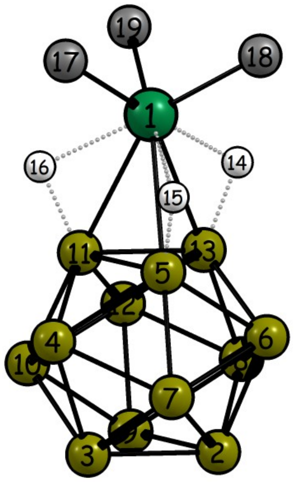


1. -1907.06549830 0.0 $C_{3v}$

| | | 1 | 2 | 3 | 4 | 5 |
|---|---|---|---|---|---|---|
| 1 | Fe | 0.000000 | | | | |
| 2 | B | 4.685079 | 0.000000 | | | |
| 3 | B | 4.680452 | 1.797682 | 0.000000 | | |
| 4 | B | 3.385869 | 2.900991 | 1.763896 | 0.000000 | |
| 5 | B | 2.209785 | 2.782281 | 2.781117 | 1.762190 | 0.000000 |
| 6 | B | 3.392517 | 1.765334 | 2.899893 | 2.947518 | 1.761082 |
| 7 | B | 3.950777 | 1.798875 | 1.797469 | 1.818303 | 1.741693 |
| 8 | B | 3.955618 | 1.798740 | 2.925318 | 3.470101 | 2.848423 |
| 9 | B | 4.682536 | 1.798480 | 1.797958 | 2.900150 | 3.289033 |
| 10 | B | 3.948092 | 2.926732 | 1.798879 | 1.818417 | 2.848033 |
| 11 | B | 2.208617 | 3.288950 | 2.779875 | 1.761135 | 1.714508 |
| 12 | B | 3.390502 | 2.901372 | 2.900271 | 2.947808 | 2.856341 |
| 13 | B | 2.213163 | 2.783627 | 3.289419 | 2.856455 | 1.714817 |
| 14 | H | 1.786252 | 3.984991 | 4.556780 | 3.890782 | 2.515021 |
| 15 | H | 1.785841 | 3.981762 | 3.979417 | 2.662069 | 1.283367 |
| 16 | H | 1.783426 | 4.556767 | 3.978943 | 2.662552 | 2.518068 |
| | | 6 | 7 | 8 | 9 | 10 |
| 6 | B | 0.000000 | | | | |
| 7 | B | 1.819109 | 0.000000 | | | |
| 8 | B | 1.818914 | 2.961879 | 0.000000 | | |
| 9 | B | 2.900390 | 2.925406 | 1.798309 | 0.000000 | |
| 10 | B | 3.469644 | 2.961430 | 2.962653 | 1.798795 | 0.000000 |
| 11 | B | 2.855116 | 2.846887 | 2.847894 | 2.779718 | 1.740051 |
| 12 | B | 2.947706 | 3.469584 | 1.819695 | 1.763814 | 1.818912 |
| 13 | B | 1.762782 | 2.849075 | 1.743299 | 2.781557 | 2.847851 |
| 14 | H | 2.666112 | 3.943765 | 2.735239 | 3.982898 | 3.942275 |
| 15 | H | 2.663991 | 2.729103 | 3.944643 | 4.556226 | 3.942025 |
| 16 | H | 3.892827 | 3.942823 | 3.943504 | 3.978484 | 2.726472 |
| | | 11 | 12 | 13 | 14 | 15 |
| 11 | B | 0.000000 | | | | |
| 12 | B | 1.761031 | 0.000000 | | | |
| 13 | B | 1.713651 | 1.761477 | 0.000000 | | |
| 14 | H | 2.514532 | 2.664814 | 1.284490 | 0.000000 | |
| 15 | H | 2.517044 | 3.893166 | 2.518241 | 2.732050 | 0.000000 |
| 16 | H | 1.283758 | 2.661826 | 2.516446 | 2.730265 | 2.735803 |
| | | 16 | | | | |
| 16 | H | 0.000000 | | | | |

Fe1-C17: 1.803208 Fe1-C18: 1.803239 Fe1-C19: 1.803314

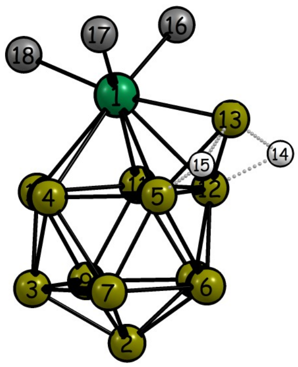


2. -1907.04981686 +9.8 $C_s$

| | | | | | | |
|---|---|---|---|---|---|---|
| | | 1 | 2 | 3 | 4 | 5 |
| 1 | Fe | 0.000000 | | | | |
| 2 | B | 4.000151 | 0.000000 | | | |
| 3 | B | 3.395274 | 1.781490 | 0.000000 | | |
| 4 | B | 2.215055 | 2.929101 | 1.763989 | 0.000000 | |
| 5 | B | 2.147472 | 2.832187 | 2.780406 | 1.774799 | 0.000000 |
| 6 | B | 3.440385 | 1.757053 | 2.851170 | 2.949980 | 1.791001 |
| 7 | B | 3.442765 | 1.799538 | 1.778926 | 1.828229 | 1.744256 |
| 8 | B | 3.442864 | 1.800207 | 2.914949 | 3.492204 | 2.866962 |
| 9 | B | 3.398127 | 1.780956 | 1.821688 | 2.937722 | 3.311137 |
| 10 | B | 2.176823 | 2.897057 | 1.788513 | 1.835220 | 2.840068 |
| 11 | B | 2.215825 | 2.927809 | 2.932887 | 3.022242 | 2.915371 |
| 12 | B | 2.146684 | 2.830056 | 3.306439 | 2.912397 | 1.766331 |
| 13 | B | 2.109205 | 4.142403 | 4.293409 | 3.187487 | 1.721511 |
| 14 | H | 2.808212 | 3.923903 | 4.552288 | 3.973076 | 2.518335 |
| 15 | H | 2.807561 | 3.924989 | 3.981088 | 2.771896 | 1.267254 |
| | | 6 | 7 | 8 | 9 | 10 |
| 6 | B | 0.000000 | | | | |
| 7 | B | 1.778695 | 0.000000 | | | |
| 8 | B | 1.779545 | 2.927587 | 0.000000 | | |
| 9 | B | 2.852110 | 2.915489 | 1.779366 | 0.000000 | |
| 10 | B | 3.412823 | 2.935099 | 2.934137 | 1.789902 | 0.000000 |
| 11 | B | 2.950911 | 3.490890 | 1.828563 | 1.763300 | 1.830373 |
| 12 | B | 1.789806 | 2.864254 | 1.743223 | 2.780307 | 2.837202 |
| 13 | B | 2.740936 | 3.426949 | 3.421892 | 4.293129 | 3.777501 |
| 14 | H | 2.539498 | 3.863671 | 2.661613 | 3.982243 | 4.023317 |
| 15 | H | 2.540585 | 2.662739 | 3.864907 | 4.554972 | 4.024155 |
| | | 11 | 12 | 13 | 14 | 15 |
| 11 | B | 0.000000 | | | | |
| 12 | B | 1.776226 | 0.000000 | | | |
| 13 | B | 3.184150 | 1.716121 | 0.000000 | | |
| 14 | H | 2.775139 | 1.269210 | 1.492241 | 0.000000 | |
| 15 | H | 3.974656 | 2.518310 | 1.502603 | 2.679870 | 0.000000 |

Fe1-C16: 1.778120 Fe1-C17: 1.777708 Fe1-C18: 1.781261

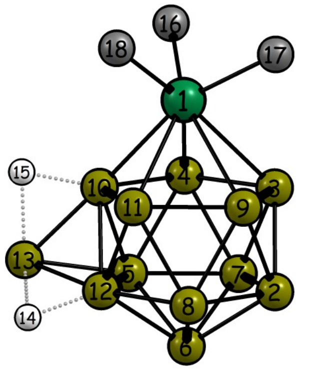


3. -1907.03696035 +17.9 $C_1$

| | | 1 | 2 | 3 | 4 | 5 |
|---|---|---|---|---|---|---|
| 1 | Fe | 0.000000 | | | | |
| 2 | B | 3.406766 | 0.000000 | | | |
| 3 | B | 2.175992 | 1.803779 | 0.000000 | | |
| 4 | B | 2.160778 | 2.911562 | 1.779114 | 0.000000 | |
| 5 | B | 3.374313 | 2.860764 | 2.869268 | 1.810448 | 0.000000 |
| 6 | B | 4.002615 | 1.764466 | 2.911759 | 2.959754 | 1.812616 |
| 7 | B | 3.412746 | 1.786808 | 1.788037 | 1.804746 | 1.768615 |
| 8 | B | 3.438092 | 1.782870 | 2.941604 | 3.488253 | 2.919644 |
| 9 | B | 2.191142 | 1.794165 | 1.845961 | 2.959249 | 3.408691 |
| 10 | B | 2.049817 | 3.327103 | 2.819104 | 1.787021 | 1.795950 |
| 11 | B | 2.156496 | 2.882545 | 2.926416 | 2.987892 | 2.938727 |
| 12 | B | 3.330164 | 2.790296 | 3.332245 | 2.901548 | 1.779316 |
| 13 | B | 3.782562 | 4.181984 | 4.201267 | 2.994697 | 1.802647 |
| 14 | H | 4.289843 | 3.996888 | 4.569033 | 3.896276 | 2.540504 |
| 15 | H | 2.981367 | 4.569536 | 4.078103 | 2.772031 | 2.505217 |
| | | 6 | 7 | 8 | 9 | 10 |
| 6 | B | 0.000000 | | | | |
| 7 | B | 1.798771 | 0.000000 | | | |
| 8 | B | 1.803915 | 2.924918 | 0.000000 | | |
| 9 | B | 2.911126 | 2.928002 | 1.797390 | 0.000000 | |
| 10 | B | 2.907609 | 2.856726 | 2.896634 | 2.839493 | 0.000000 |
| 11 | B | 2.939119 | 3.441329 | 1.809673 | 1.767700 | 1.803481 |
| 12 | B | 1.763654 | 2.836298 | 1.742898 | 2.815031 | 1.785678 |
| 13 | B | 2.982805 | 3.520930 | 3.451528 | 4.162605 | 1.735784 |
| 14 | H | 2.709668 | 3.939033 | 2.738942 | 3.975434 | 2.506834 |
| 15 | H | 3.855479 | 3.951990 | 3.916205 | 4.052824 | 1.296311 |
| | | 11 | 12 | 13 | 14 | 15 |
| 11 | B | 0.000000 | | | | |
| 12 | B | 1.774776 | 0.000000 | | | |
| 13 | B | 2.883609 | 1.723621 | 0.000000 | | |
| 14 | H | 2.616325 | 1.261532 | 1.523222 | 0.000000 | |
| 15 | H | 2.686674 | 2.459143 | 1.423030 | 2.538781 | 0.000000 |

Fe1-C16: 1.772597 Fe1-C17: 1.770661 Fe1-C18: 1.772478

Table S8C: Distance table for the lowest-lying $B_{12}H_{12}Fe(CO)_4$ optimized structures obtained at the PBE0/def2-TZVP level of theory. Included are the zero-point corrected absolute energy in (a.u.) at the DLPNO-CCSD(T)/def2-QZVP level of theory with zero-point energy obtained from the PBE0/def2-TZVP computations, relative energies in (kcal/mol) and symmetry. For clarity, only the atoms forming the cluster framework are shown.

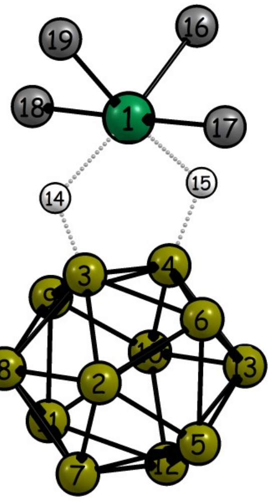


1. -2020.20436761 0.0 $C_{2v}$

| | | 1 | 2 | 3 | 4 | 5 |
|---|---|---|---|---|---|---|
| 1 | Fe | 0.000000 | | | | |
| 2 | B | 3.919681 | 0.000000 | | | |
| 3 | B | 2.298405 | 1.759165 | 0.000000 | | |
| 4 | B | 2.295574 | 2.836415 | 1.693790 | 0.000000 | |
| 5 | B | 4.662705 | 1.808759 | 2.834985 | 2.830701 | 0.000000 |
| 6 | B | 3.065674 | 1.773750 | 1.774206 | 1.772750 | 1.748359 |
| 7 | B | 5.010830 | 1.791233 | 2.796873 | 3.289241 | 1.783217 |
| 8 | B | 3.898621 | 1.834440 | 1.761799 | 2.838262 | 2.933783 |
| 9 | B | 3.021644 | 2.908918 | 1.770107 | 1.768530 | 3.390878 |
| 10 | B | 3.892330 | 3.459281 | 2.842116 | 1.759554 | 2.934578 |
| 11 | B | 4.638730 | 2.937862 | 2.842117 | 2.837752 | 2.911865 |
| 12 | B | 5.009874 | 2.908239 | 3.296414 | 2.794307 | 1.784750 |
| 13 | B | 3.913353 | 2.931361 | 2.839740 | 1.756281 | 1.807900 |
| 14 | H | 1.783214 | 2.660584 | 1.277533 | 2.566070 | 3.985717 |
| 15 | H | 1.779535 | 3.950361 | 2.568038 | 1.278195 | 3.974657 |

| | | 6 | 7 | 8 | 9 | 10 |
|---|---|---|---|---|---|---|
| 6 | B | 0.000000 | | | | |
| 7 | B | 2.840575 | 0.000000 | | | |
| 8 | B | 2.920468 | 1.789850 | 0.000000 | | |
| 9 | B | 2.921838 | 2.828317 | 1.767737 | 0.000000 | |
| 10 | B | 2.922258 | 2.905509 | 2.932307 | 1.768210 | 0.000000 |
| 11 | B | 3.409161 | 1.783182 | 1.811969 | 1.745763 | 1.810779 |
| 12 | B | 2.843321 | 1.787354 | 2.907272 | 2.830455 | 1.791234 |
| 13 | B | 1.774951 | 2.905181 | 3.458162 | 2.909197 | 1.835494 |
| 14 | H | 2.660018 | 3.958507 | 2.647984 | 2.636979 | 3.946551 |
| 15 | H | 2.649853 | 4.566273 | 3.950164 | 2.645695 | 2.646644 |

| | | 11 | 12 | 13 | 14 | 15 |
|---|---|---|---|---|---|---|
| 11 | B | 0.000000 | | | | |
| 12 | B | 1.783702 | 0.000000 | | | |
| 13 | B | 2.936850 | 1.791832 | 0.000000 | | |
| 14 | H | 3.976639 | 4.572305 | 3.954471 | 0.000000 | |
| 15 | H | 3.978080 | 3.952948 | 2.647159 | 2.926524 | 0.000000 |

Fe1-C16: 1.801760 Fe1-C17: 1.878351

Fe1-C18: 1.892608 Fe1-C19: 1.801790

Table S8D: Energy ranking for the $B_5H_5Fe(CO)_2$ structures at the PBE0/def2-TZVP level of theory.

| No. | Initial structure | Final Energy (a.u.) | Relative Energy (kcal/mol) |
|---|---|---|---|
| 1 | 13v-01-Doc-fe3-2co-pbe0 | -1794.9057630 | 0.00 |
| 2 | 13v-03-DitrapIcos-fe3-2co-r-12-pbe0 | -1794.9005600 | 3.26 |
| 3 | 13v-03-DitrapIcos-fe3-2co-i-12-pbe0 | -1794.9005580 | 3.27 |
| 4 | 13v-02-Hen-fe2-2co-pbe0 | -1794.8942170 | 7.25 |
| 5 | 13v-01-Doc-fe2-2co-pbe0 | -1794.8942100 | 7.25 |
| 6 | 13v-03-DitrapIcos-fe2-2co-pbe0 | -1794.8941710 | 7.27 |
| 7 | 13v-01-Doc-fe1-2co-pbe0 | -1794.8550040 | 31.85 |
| 8 | 13v-02-Hen-fe1-2co-pbe0 | -1794.8448540 | 38.22 |
| 9 | 13v-03-DitrapIcos-fe1-2co-pbe0 | -1794.8432060 | 39.26 |
| 10 | 13v-02-Hen-fe3-2co-pbe0 | -1794.8384820 | 42.22 |
| 11 | 13v-02-Hen-fe4-2co-pbe0 | -1794.8384600 | 42.23 |
| 12 | 13v-04-CapHexAnPr-a-fe2-2co-pbe0 | -1794.8100820 | 60.04 |
| 13 | 13v-04-CapHexAnPr-a-fe3-2co-pbe0 | -1794.7826030 | 77.28 |
| 14 | 13v-04-CapHexAnPr-a-fe1-2co-pbe0 | -1794.7664310 | 87.43 |

Table S8E: Energy ranking for the $B_5H_5Fe(CO)_2$ structures at the PBE0/def2-TZVP level of theory.

| No. | Initial structure | Final Energy (a.u.) | Relative Energy (kcal/mol) |
|---|---|---|---|
| 1 | 13v-01-Doc-fe3-3co-pbe0 | -1908.1338940 | 0.00 |
| 2 | 13v-03-DitrapIcos-fe3-3co-pbe0 | -1908.1338920 | 0.00 |
| 3 | 13v-02-Hen-fe2-3co-pbe0 | -1908.1030880 | 19.33 |
| 4 | 13v-02-Hen-fe4-3co-pbe0 | -1908.1030560 | 19.35 |
| 5 | 13v-02-Hen-fe3-3co-pbe0 | -1908.1030390 | 19.36 |
| 6 | 13v-03-DitrapIcos-fe2-3co-pbe0 | -1908.1030330 | 19.37 |
| 7 | 13v-01-Doc-fe2-3co-pbe0 | -1908.1030120 | 19.38 |
| 8 | 13v-01-Doc-fe1-3co-pbe0 | -1908.0930770 | 25.61 |
| 9 | 13v-02-Hen-fe1-3co-pbe0 | -1908.0808280 | 33.30 |
| 10 | 13v-03-DitrapIcos-fe1-3co-pbe0 | -1908.0806790 | 33.39 |
| 11 | 13v-04-CapHexAnPr-a-fe3-3co-pbe0 | -1908.0120920 | 76.43 |
| 12 | 13v-04-CapHexAnPr-a-fe2-3co-pbe0 | -1908.0068830 | 79.70 |
| 13 | 13v-04-CapHexAnPr-a-fe1-3co-pbe0 | -1908.0046220 | 81.12 |

Table S8F: Energy ranking for the $B_5H_5Fe(CO)_2$ structures at the PBE0/def2-TZVP level of theory.

| No. | Initial structure | Final Energy (a.u.) | Relative Energy (kcal/mol) |
|---|---|---|---|
| 1 | 13v-02-Hen-fe4-4co-pbe0 | -2021.3004060 | 0.00 |
| 2 | 13v-02-Hen-fe3-4co-pbe0 | -2021.3003660 | 0.03 |
| 3 | 13v-01-Doc-fe3-4co-pbe0 | -2021.3003380 | 0.04 |
| 4 | 13v-03-DitrapIcos-fe2-4co-pbe0 | -2021.3003120 | 0.06 |
| 5 | 13v-03-DitrapIcos-fe3-4co-pbe0 | -2021.3003110 | 0.06 |
| 6 | 13v-01-Doc-fe2-4co-pbe0 | -2021.2738530 | 16.66 |
| 7 | 13v-02-Hen-fe2-4co-pbe0 | -2021.2737740 | 16.71 |
| 8 | 13v-03-DitrapIcos-fe1-4co-pbe0 | -2021.2515870 | 30.63 |
| 9 | 13v-02-Hen-fe1-4co-r-20-pbe0 | -2021.2512120 | 30.87 |
| 10 | 13v-02-Hen-fe1-4co-i-20-pbe0 | -2021.2512020 | 30.88 |
| 11 | 13v-01-Doc-fe1-4co-pbe0 | -2021.2277130 | 45.62 |
| 12 | 13v-04-CapHexAnPr-a-fe3-4co-pbe0 | -2021.1946040 | 66.39 |
| 13 | 13v-04-CapHexAnPr-a-fe2-4co-pbe0 | -2021.1797580 | 75.71 |
| 14 | 13v-04-CapHexAnPr-a-fe1-4co-pbe0 | -2021.1754520 | 78.41 |

Table S9A: Distance table for the lowest-lying $[B_5H_5Fe(CO)_3]^{2-}$ optimized structures obtained at the PBE0/def2-TZVP level of theory. Included are the zero-point corrected absolute energy in (a.u.) at the DLPNO-CCSD(T)/def2-QZVP level of theory with zero-point energy obtained from the PBE0/def2-TZVP computations, relative energies in (kcal/mol) and symmetry. For clarity, only the atoms forming the cluster framework are shown.

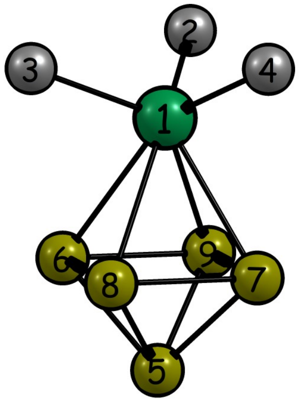


1. -1730.376329 0.0 $C_s$

| | | 1 | 2 | 3 | 4 | 5 |
|---|---|---|---|---|---|---|
| 1 | Fe | 0.000000 | | | | |
| 2 | B | 2.902840 | 0.000000 | | | |
| 3 | B | 2.136648 | 1.705006 | 0.000000 | | |
| 4 | B | 2.110397 | 1.709461 | 2.473814 | 0.000000 | |
| 5 | B | 2.135332 | 1.705286 | 1.756185 | 1.747595 | 0.000000 |
| 6 | B | 2.110929 | 1.709681 | 1.747728 | 1.745314 | 2.473676 |

| | | 6 |
|---|---|---|
| 6 | B | 0.000000 |

Fe1-C2: 1.731572 Fe1-C3: 1.733541 Fe1-C4: 1.731355

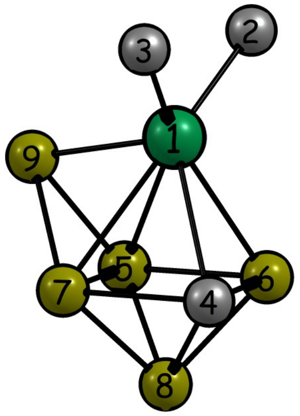


2. -1730.319167 +35.9 $C_1$

| | | 1 | 2 | 3 | 4 | 5 |
|---|---|---|---|---|---|---|
| 1 | Fe | 0.000000 | | | | |
| 2 | B | 2.165699 | 0.000000 | | | |
| 3 | B | 2.039661 | 1.814064 | 0.000000 | | |
| 4 | B | 2.169285 | 1.745458 | 2.476437 | 0.000000 | |
| 5 | B | 2.867786 | 1.724178 | 1.701115 | 1.701284 | 0.000000 |
| 6 | B | 1.817493 | 1.794062 | 3.028645 | 1.798980 | 3.035407 |

| | | 6 |
|---|---|---|
| 6 | B | 0.000000 |

Fe1-C2: 1.729097 Fe1-C3: 1.729605 Fe1-C4: 2.070640

Table S9B: Energy ranking for the $[B_5H_5Fe(CO)_3]^{2-}$ structures at the PBE0/def2-TZVP level of theory.

| No. | Initial structure | Final Energy (a.u.) | Relative Energy (kcal/mol) |
|---|---|---|---|
| 1 | 06v-01-Oh—fe1—3co-dianion-tz.log | -1730.3763290 | 0.0 |
| 2 | 06v-02-BicappTd—fe1—3co-dianion-tz.log | -1730.3191670 | 35.9 |
| 3 | 06v-04-PentPyr—fe1—3co-dianion-tz.log | -1730.3191590 | 35.9 |
| 4 | 06v-02-BicappTd—fe2—3co-dianion-tz.log | -1730.3191560 | 35.9 |
| 5 | 06v-03-CapTetrPyr—fe2—3co-dianion-tz.log | -1730.3191500 | 35.9 |
| 6 | 06v-03-CapTetrPyr—fe1—3co-dianion-tz.log | -1730.3191440 | 35.9 |

Table S10A: Distance table for the lowest-lying $[B_5H_5Fe(CO)_3]^{2-}$ optimized structures obtained at the PBE0/def2-TZVP level of theory. Included are the zero-point corrected absolute energy in (a.u.) at the DLPNO-CCSD(T)/def2-QZVP level of theory with zero-point energy obtained from the PBE0/def2-TZVP computations, relative energies in (kcal/mol) and symmetry. For clarity, only the atoms forming the cluster framework are shown.

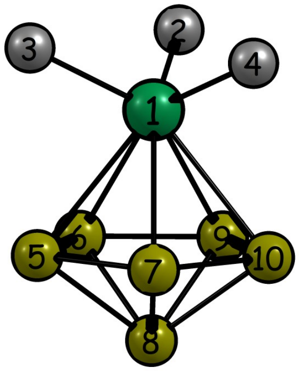


1. -1755.809853 0.0 $C_s$

| | | 1 | 2 | 3 | 4 | 5 |
|---|---|---|---|---|---|---|
| 1 | Fe | 0.000000 | | | | |
| 2 | B | 2.202528 | 0.000000 | | | |
| 3 | B | 2.212922 | 1.684084 | 0.000000 | | |
| 4 | B | 2.212056 | 1.684017 | 2.729662 | 0.000000 | |
| 5 | B | 2.743412 | 1.785718 | 1.784128 | 1.783950 | 0.000000 |
| 6 | B | 2.207711 | 2.724876 | 1.688597 | 2.728198 | 1.786141 |
| 7 | B | 2.207297 | 2.725122 | 2.728121 | 1.689113 | 1.786073 |
| | | 6 | 7 | | | |
| 6 | B | 0.000000 | | | | |
| 7 | B | 1.681755 | 0.000000 | | | |

Fe1-C2: 1.734471 Fe1-C3: 1.734468 Fe1-C4: 1.734341

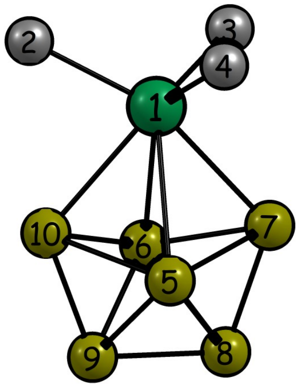


2. -1755.800907 +5.6 $C_s$

| | | 1 | 2 | 3 | 4 | 5 |
|---|---|---|---|---|---|---|
| 1 | Fe | 0.000000 | | | | |
| 2 | B | 2.236586 | 0.000000 | | | |
| 3 | B | 2.256023 | 2.295511 | 0.000000 | | |
| 4 | B | 2.048338 | 1.837210 | 1.846864 | 0.000000 | |
| 5 | B | 3.169425 | 1.813452 | 1.808607 | 1.638301 | 0.000000 |
| 6 | B | 3.172330 | 1.806306 | 1.801715 | 2.677446 | 1.631620 |
| 7 | B | 2.039281 | 1.825221 | 1.831470 | 2.741146 | 2.680350 |
| | | 6 | 7 | | | |
| 6 | B | 0.000000 | | | | |
| 7 | B | 1.650484 | 0.000000 | | | |

Fe1-C2: 1.736298 Fe1-C3: 1.732162 Fe1-C4: 1.730035

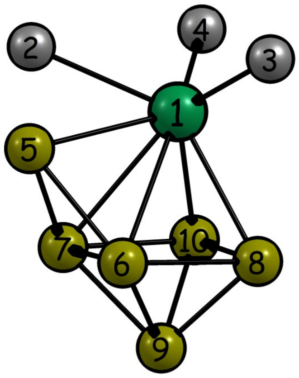


3. -1755.764792 +28.3 $C_1$

| | | 1 | 2 | 3 | 4 | 5 |
|---|---|---|---|---|---|---|
| 1 | Fe | 0.000000 | | | | |
| 2 | B | 2.081912 | 0.000000 | | | |
| 3 | B | 2.236181 | 1.799865 | 0.000000 | | |
| 4 | B | 2.226284 | 1.923398 | 1.760673 | 0.000000 | |
| 5 | B | 2.083999 | 3.166788 | 1.828243 | 2.508913 | 0.000000 |
| 6 | B | 2.891632 | 3.133343 | 1.737722 | 1.734518 | 1.660697 |
| 7 | B | 2.024699 | 3.185008 | 2.527013 | 1.778920 | 1.755521 |
| | | 6 | 7 | | | |
| 6 | B | 0.000000 | | | | |
| 7 | B | 1.724889 | 0.000000 | | | |

Fe1-C2: 1.955853 Fe1-C3: 1.735076 Fe1-C4: 1.719814

Table S10B: Energy ranking for the $[B_5H_5Fe(CO)_3]^{2-}$ structures at the PBE0/def2-TZVP level of theory.

| No. | Initial structure | Final Energy (a.u.) | Relative Energy (kcal/mol) |
|---|---|---|---|
| 1 | 07v-04-CapOh—fe4—3co-dianion-tz.log | -1755.8098530 | 0.0 |
| 2 | 07v-01-PentBipyr—fe1—3co-dianion-tz.log | -1755.8098370 | 0.0 |
| 3 | 07v-03-TricapTd—fe1—3co-dianion-tz.log | -1755.8098280 | 0.0 |
| 4 | 07v-04-CapOh—fe3—3co-dianion-tz.log | -1755.8098210 | 0.0 |
| 5 | 07v-01-PentBipyr—fe1—3co-alter-dianion-tz.log | -1755.8098100 | 0.0 |
| 6 | 07v-01-PentBipyr—fe2—3co-dianion-tz.log | -1755.8009070 | 5.6 |
| 7 | 07v-04-CapOh—fe2—3co-alter-dianion-tz.log | -1755.8008510 | 5.6 |
| 8 | 07v-04-CapOh—fe2—3co-dianion-tz.log | -1755.8008370 | 5.7 |
| 9 | 07v-04-CapOh—fe1—3co-dianion-tz.log | -1755.7647920 | 28.3 |
| 10 | 07v-03-TricapTd—fe3—3co-dianion-tz.log | -1755.7268800 | 52.1 |
| 11 | 07v-03-TricapTd—fe2—3co-dianion-tz.log | -1755.7225080 | 54.8 |
| 12 | 07v-02-HexPyr—fe1—3co-dianion-tz.log | -1755.7213410 | 55.5 |

Table S11A: Distance table for the lowest-lying $[B_5H_5Fe(CO)_3]^{2-}$ optimized structures obtained at the PBE0/def2-TZVP level of theory. Included are the zero-point corrected absolute energy in (a.u.) at the DLPNO-CCSD(T)/def2-QZVP level of theory with zero-point energy obtained from the PBE0/def2-TZVP computations, relative energies in (kcal/mol) and symmetry. For clarity, only the atoms forming the cluster framework are shown.

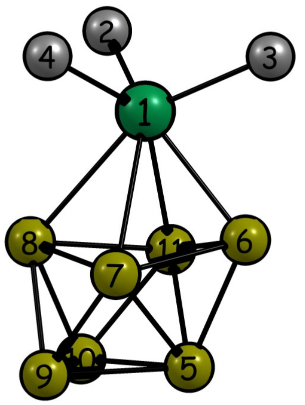


1. -1781.236246 0.0 $C_s$

| | | 1 | 2 | 3 | 4 | 5 |
|---|---|---|---|---|---|---|
| 1 | Fe | 0.000000 | | | | |
| 2 | B | 3.158942 | 0.000000 | | | |
| 3 | B | 1.981798 | 1.728390 | 0.000000 | | |
| 4 | B | 2.222461 | 1.865564 | 1.811593 | 0.000000 | |
| 5 | B | 2.187761 | 2.475421 | 2.702042 | 1.897129 | 0.000000 |
| 6 | B | 3.421795 | 1.780038 | 2.924604 | 1.717255 | 1.765201 |
| 7 | B | 3.422304 | 1.779641 | 2.923671 | 2.637459 | 1.765567 |
| 8 | B | 2.222747 | 1.866369 | 1.810041 | 2.485143 | 1.896794 |

| | | 6 | 7 | 8 |
|---|---|---|---|---|
| 6 | B | 0.000000 | | |
| 7 | B | 1.612625 | 0.000000 | |
| 8 | B | 2.637832 | 1.717608 | 0.000000 |

Fe1-C2: 1.737933 Fe1-C3: 1.732403 Fe1-C4: 1.737874

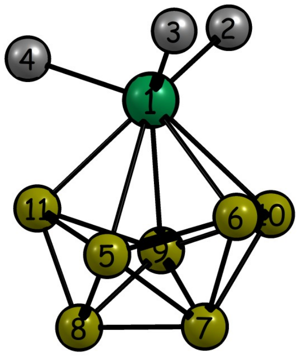


2. -1781.225575 +6.7 $C_s$

| | | 1 | 2 | 3 | 4 | 5 |
|---|---|---|---|---|---|---|
| 1 | Fe | 0.000000 | | | | |
| 2 | B | 2.354249 | 0.000000 | | | |
| 3 | B | 2.184368 | 1.737544 | 0.000000 | | |
| 4 | B | 3.004428 | 1.849341 | 1.779576 | 0.000000 | |
| 5 | B | 3.092652 | 1.764142 | 2.880991 | 1.688125 | 0.000000 |
| 6 | B | 2.351524 | 2.545169 | 2.681355 | 1.849222 | 1.764212 |
| 7 | B | 2.183954 | 2.681900 | 1.638981 | 1.778982 | 2.881176 |
| 8 | B | 2.074431 | 1.905623 | 3.021837 | 2.710942 | 1.633928 |

| | | 6 | 7 | 8 |
|---|---|---|---|---|
| 6 | B | 0.000000 | | |
| 7 | B | 1.737880 | 0.000000 | |
| 8 | B | 1.905817 | 3.022836 | 0.000000 |

Fe1-C2: 1.731184 Fe1-C3: 1.731381 Fe1-C4: 1.734665

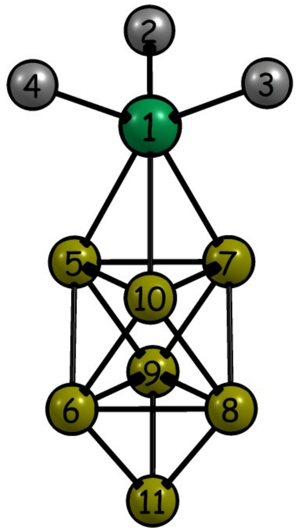


3. -1781.173010 +39.7 $C_{3v}$

| | | 1 | 2 | 3 | 4 | 5 |
|---|---|---|---|---|---|---|
| 1 | Fe | 0.000000 | | | | |
| 2 | B | 2.068918 | 0.000000 | | | |
| 3 | B | 3.447268 | 1.823206 | 0.000000 | | |
| 4 | B | 2.069867 | 1.799364 | 2.581099 | 0.000000 | |
| 5 | B | 3.448312 | 2.581858 | 1.856260 | 1.823131 | 0.000000 |
| 6 | B | 3.447259 | 1.823541 | 1.856312 | 1.823334 | 1.856265 |
| 7 | B | 2.069762 | 1.799002 | 1.823379 | 1.798412 | 1.823692 |
| 8 | B | 4.541909 | 2.941373 | 1.658025 | 2.941186 | 1.658003 |

| | | 6 | 7 | 8 |
|---|---|---|---|---|
| 6 | B | 0.000000 | | |
| 7 | B | 2.581475 | 0.000000 | |
| 8 | B | 1.658079 | 2.941516 | 0.000000 |

Fe1-C2: 1.734894 Fe1-C3: 1.735067 Fe1-C4: 1.734923

Table S11B: Energy ranking for the $[B_5H_5Fe(CO)_3]^{2-}$ structures at the PBE0/def2-TZVP level of theory.

| No. | Initial structure | Final Energy (a.u.) | Relative Energy (kcal/mol) |
|---|---|---|---|
| 1 | 08v-08-Nido—fe2—3co-dianion-tz.log | -1781.2362460 | 0.0 |
| 2 | 08v-01-Bisdisph—fe2—3co-dianion-tz.log | -1781.2362440 | 0.0 |
| 3 | 08v-09-DicapTrPrA—fe3—3co-dianion-tz.log | -1781.2362080 | 0.0 |
| 4 | 08v-01-Bisdisph—fe4—3co-dianion-tz.log | -1781.2362070 | 0.0 |
| 5 | 08v-01-Bisdisph—fe4—3co-alter-dianion-tz.log | -1781.2361960 | 0.0 |
| 6 | 08v-11-DicapOh—fe1—3co-dianion-tz.log | -1781.2361920 | 0.0 |
| 7 | 08v-10-DicapTrPrB—fe3—3co-dianion-tz.log | -1781.2361850 | 0.0 |
| 8 | 08v-03-Antipr—fe1—3co-dianion-tz.log | -1781.2361830 | 0.0 |
| 9 | 08v-10-DicapTrPrB—fe1—3co-dianion-tz.log | -1781.2361800 | 0.0 |
| 10 | 08v-01-Bisdisph—fe3—3co-dianion-tz.log | -1781.2259600 | 6.5 |
| 11 | 08v-06-Tdallcap—fe2—3co-dianion-tz.log | -1781.2259540 | 6.5 |
| 12 | 08v-06-Tdallcap—fe1—3co-alter1-dianion-tz.log | -1781.2259530 | 6.5 |
| 13 | 08v-09-DicapTrPrA—fe2—3co-alter-dianion-tz.log | -1781.2259460 | 6.5 |
| 14 | 08v-08-Nido—fe1—3co-dianion-tz.log | -1781.2259360 | 6.5 |
| 15 | 08v-10-DicapTrPrB—fe2—3co-dianion-tz.log | -1781.2259340 | 6.5 |
| 16 | 08v-04-TrigPyr—fe3—3co-dianion-tz.log | -1781.2255750 | 6.7 |
| 17 | 08v-06-Tdallcap—fe1—3co-alter2-dianion-tz.log | -1781.2255550 | 6.7 |
| 18 | 08v-05-AntiprTrig—fe2—3co-dianion-tz.log | -1781.2255500 | 6.7 |
| 19 | 08v-05-AntiprTrig—fe3—3co-dianion-tz.log | -1781.2255380 | 6.7 |
| 20 | 08v-01-Bisdisph—fe1—3co-dianion-tz.log | -1781.2255360 | 6.7 |
| 21 | 08v-09-DicapTrPrA—fe1—3co-dianion-tz.log | -1781.2255330 | 6.7 |
| 22 | 08v-06-Tdallcap—fe1—3co-alter3-dianion-tz.log | -1781.2255120 | 6.7 |
| 23 | 08v-07-Bipirhex—fe2—3co-dianion-tz.log | -1781.2164890 | 12.4 |
| 24 | 08v-09-DicapTrPrA—fe2—3co-dianion-tz.log | -1781.1908800 | 28.5 |
| 25 | 08v-09-DicapTrPrA—fe4—3co-dianion-tz.log | -1781.1785890 | 36.2 |
| 26 | 08v-11-DicapOh—fe3—3co-dianion-tz.log | -1781.1742850 | 38.9 |
| 27 | 08v-05-AntiprTrig—fe1—3co-dianion-tz.log | -1781.1730100 | 39.7 |
| 28 | 08v-04-TrigPyr—fe2—3co-dianion-tz.log | -1781.1727160 | 39.9 |
| 29 | 08v-07-Bipirhex—fe1—3co-dianion-tz.log | -1781.1717240 | 40.5 |
| 30 | 08v-02-Cube—fe1—3co-dianion-tz.log | -1781.1698830 | 41.6 |
| 31 | 08v-11-DicapOh—fe2—3co-dianion-tz.log | -1781.1517690 | 53.0 |
| 32 | 08v-04-TrigPyr—fe1—3co-dianion-tz.log | -1781.1452780 | 57.1 |
| 33 | 08v-06-Tdallcap—fe1—3co-dianion-tz.log | -1781.1066370 | 81.3 |

Table S12A: Distance table for the lowest-lying $[B_5H_5Fe(CO)_3]^{2-}$ optimized structures obtained at the PBE0/def2-TZVP level of theory. Included are the zero-point corrected absolute energy in (a.u.) at the DLPNO-CCSD(T)/def2-QZVP level of theory with zero-point energy obtained from the PBE0/def2-TZVP computations, relative energies in (kcal/mol) and symmetry. For clarity, only the atoms forming the cluster framework are shown.

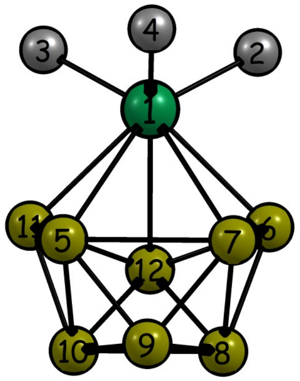


1. -1806.681373 0.0 $C_s$

| | | 1 | 2 | 3 | 4 | 5 |
|---|---|---|---|---|---|---|
| 1 | Fe | 0.000000 | | | | |
| 2 | B | 2.385775 | 0.000000 | | | |
| 3 | B | 2.086250 | 3.032024 | 0.000000 | | |
| 4 | B | 2.386973 | 2.101026 | 1.725640 | 0.000000 | |
| 5 | B | 3.138627 | 2.653248 | 1.722133 | 1.778038 | 0.000000 |
| 6 | B | 3.369106 | 1.685385 | 2.863226 | 1.685527 | 1.713439 |
| 7 | B | 3.138120 | 1.778611 | 2.903098 | 2.653862 | 1.846200 |
| 8 | B | 2.084815 | 1.725934 | 2.958151 | 3.032131 | 2.902438 |
| 9 | B | 2.229324 | 2.677816 | 1.751515 | 2.678030 | 1.894272 |

| | | 6 | 7 | 8 | 9 |
|---|---|---|---|---|---|
| 6 | B | 0.000000 | | | |
| 7 | B | 1.713994 | 0.000000 | | |
| 8 | B | 2.862972 | 1.721542 | 0.000000 | |
| 9 | B | 2.900725 | 1.893879 | 1.750584 | 0.000000 |

Fe1-C2: 1.734530 Fe1-C3: 1.734324 Fe1-C4: 1.738687

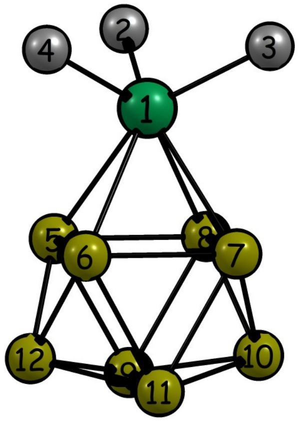


2. -1806.680651 +0.5 $C_s$

| | | 1 | 2 | 3 | 4 | 5 |
|---|---|---|---|---|---|---|
| 1 | Fe | 0.000000 | | | | |
| 2 | B | 2.147017 | 0.000000 | | | |
| 3 | B | 2.136736 | 1.851811 | 0.000000 | | |
| 4 | B | 2.136618 | 2.640699 | 1.872140 | 0.000000 | |
| 5 | B | 2.145964 | 1.891513 | 2.639525 | 1.851708 | 0.000000 |
| 6 | B | 3.460004 | 1.901100 | 2.641162 | 2.641771 | 1.901411 |
| 7 | B | 3.353564 | 2.874152 | 2.869292 | 1.711863 | 1.703268 |
| 8 | B | 3.472063 | 2.639213 | 1.917776 | 1.918001 | 2.638925 |
| 9 | B | 3.353954 | 1.703232 | 1.711614 | 2.869794 | 2.873953 |

| | | 6 | 7 | 8 | 9 |
|---|---|---|---|---|---|
| 6 | B | 0.000000 | | | |
| 7 | B | 1.708282 | 0.000000 | | |
| 8 | B | 1.795496 | 1.702109 | 0.000000 | |
| 9 | B | 1.708106 | 2.833244 | 1.702195 | 0.000000 |

Fe1-C2: 1.732969 Fe1-C3: 1.740439 Fe1-C4: 1.740621

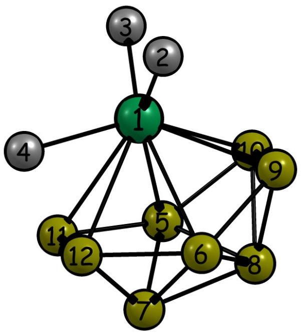


3. -1806.659205 +13.9 $C_s$

| | | 1 | 2 | 3 | 4 | 5 |
|---|---|---|---|---|---|---|
| 1 | Fe | 0.000000 | | | | |
| 2 | B | 2.358422 | 0.000000 | | | |
| 3 | B | 2.347425 | 2.705568 | 0.000000 | | |
| 4 | B | 2.881450 | 1.816062 | 1.818115 | 0.000000 | |
| 5 | B | 2.861188 | 1.803653 | 1.804051 | 1.792786 | 0.000000 |
| 6 | B | 2.176422 | 2.731092 | 1.753874 | 2.951447 | 1.757782 |
| 7 | B | 2.177722 | 1.752492 | 2.728939 | 2.949338 | 1.759094 |
| 8 | B | 2.304784 | 1.780043 | 2.778580 | 1.735671 | 2.960181 |
| 9 | B | 2.301057 | 2.778966 | 1.780306 | 1.735041 | 2.960102 |

| | | 6 | 7 | 8 | 9 |
|---|---|---|---|---|---|
| 6 | B | 0.000000 | | | |
| 7 | B | 1.618648 | 0.000000 | | |
| 8 | B | 3.575174 | 3.167546 | 0.000000 | |
| 9 | B | 3.172790 | 3.572862 | 1.682666 | 0.000000 |

Fe1-C2: 1.734902 Fe1-C3: 1.735598 Fe1-C4: 1.789386

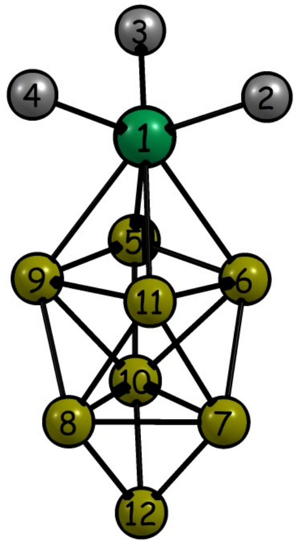


4. -1806.611218 +44.0 $C_s$

| | | 1 | 2 | 3 | 4 | 5 |
|---|---|---|---|---|---|---|
| 1 | Fe | 0.000000 | | | | |
| 2 | B | 2.082832 | 0.000000 | | | |
| 3 | B | 2.133232 | 1.726707 | 0.000000 | | |
| 4 | B | 3.460179 | 3.024585 | 1.879694 | 0.000000 | |
| 5 | B | 3.454328 | 3.013272 | 2.811319 | 1.815001 | 0.000000 |
| 6 | B | 2.127928 | 1.729612 | 2.432923 | 2.826216 | 1.877902 |
| 7 | B | 3.167403 | 1.671020 | 1.893998 | 1.941483 | 1.921078 |
| 8 | B | 2.113596 | 2.676558 | 1.901953 | 1.789203 | 1.787354 |
| 9 | B | 4.432157 | 3.361590 | 2.982014 | 1.641674 | 1.646398 |

| | | 6 | 7 | 8 | 9 |
|---|---|---|---|---|---|
| 6 | B | 0.000000 | | | |
| 7 | B | 1.891965 | 0.000000 | | |
| 8 | B | 1.901694 | 2.449742 | 0.000000 | |
| 9 | B | 2.998293 | 1.722842 | 2.886942 | 0.000000 |

Fe1-C2: 1.736066 Fe1-C3: 1.734782 Fe1-C4: 1.736255

Table S12B: Energy ranking for the $[B_5H_5Fe(CO)_3]^{2-}$ structures at the PBE0/def2-TZVP level of theory.

| No. | Initial structure | Final Energy (a.u.) | Relative Energy (kcal/mol) |
|---|---|---|---|
| 1 | 09v-04-Tl99—fe3—3co-dianion-tz.log | -1806.6813730 | 0.0 |
| 2 | 09v-02-CapCube—fe5—3co-dianion-tz.log | -1806.6813580 | 0.0 |
| 3 | 09v-03-CapSqAntPr—fe1—3co-dianion-tz.log | -1806.6806510 | 0.5 |
| 4 | 09v-07-Isocloso-6vx—fe3—3co-dianion-tz.log | -1806.6806490 | 0.5 |
| 5 | 09v-02-CapCube—fe1—3co-dianion-tz.log | -1806.6806320 | 0.5 |
| 6 | 09v-01-TricTrPrism—fe1—3co-dianion-tz.log | -1806.6803800 | 0.6 |
| 7 | 09v-07-Isocloso-6vx—fe1—3co-dianion-tz.log | -1806.6790790 | 1.4 |
| 8 | 09v-03-CapSqAntPr—fe3—3co-dianion-tz.log | -1806.6790690 | 1.4 |
| 9 | 09v-02-CapCube—fe2—3co-dianion-tz.log | -1806.6790630 | 1.4 |
| 10 | 09v-04-Tl99—fe2—3co-dianion-tz.log | -1806.6790370 | 1.5 |
| 11 | 09v-01-TricTrPrism—fe2—3co-dianion-tz.log | -1806.6790260 | 1.5 |
| 12 | 09v-03-CapSqAntPr—fe2—3co-dianion-tz.log | -1806.6790200 | 1.5 |
| 13 | 09v-07-Isocloso-6vx—fe2—3co-dianion-tz.log | -1806.6790190 | 1.5 |
| 14 | 09v-01-TricTrPrism—fe2—3co-alter-dianion-tz.log | -1806.6789860 | 1.5 |
| 15 | 09v-06-BicapPentBipyr—fe1—3co-dianion-tz.log | -1806.6675910 | 8.6 |
| 16 | 09v-02-CapCube—fe4—3co-dianion-tz.log | -1806.6675750 | 8.7 |
| 17 | 09v-04-Tl99—fe1—3co-alter-dianion-tz.log | -1806.6592050 | 13.9 |
| 18 | 09v-04-Tl99—fe1—3co-dianion-tz.log | -1806.6591840 | 13.9 |
| 19 | 09v-06-BicapPentBipyr—fe3—3co-dianion-tz.log | -1806.6591780 | 13.9 |
| 20 | 09v-02-CapCube—fe3—3co-dianion-tz.log | -1806.6472140 | 21.4 |
| 21 | 09v-06-BicapPentBipyr—fe5—3co-dianion-tz.log | -1806.6471840 | 21.5 |
| 22 | 09v-06-BicapPentBipyr—fe4—3co-dianion-tz.log | -1806.6275480 | 33.8 |
| 23 | 09v-06-BicapPentBipyr—fe2—3co-dianion-tz.log | -1806.6112180 | 44.0 |

Table S13A: Distance table for the lowest-lying $[B_5H_5Fe(CO)_3]^{2-}$ optimized structures obtained at the PBE0/def2-TZVP level of theory. Included are the zero-point corrected absolute energy in (a.u.) at the DLPNO-CCSD(T)/def2-QZVP level of theory with zero-point energy obtained from the PBE0/def2-TZVP computations, relative energies in (kcal/mol) and symmetry. For clarity, only the atoms forming the cluster framework are shown.

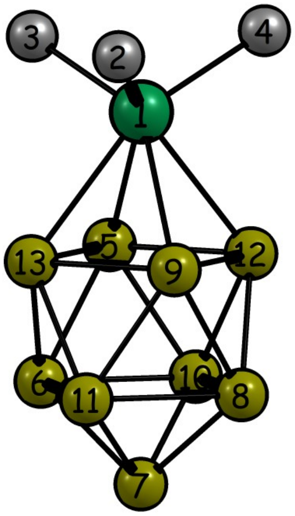


1. -1832.156127 0.0 $C_s$

| | | 1 | 2 | 3 | 4 | 5 |
|---|---|---|---|---|---|---|
| 1 | Fe | 0.000000 | | | | |
| 2 | B | 2.115147 | 0.000000 | | | |
| 3 | B | 3.416077 | 1.797577 | 0.000000 | | |
| 4 | B | 4.261375 | 2.901679 | 1.701785 | 0.000000 | |
| 5 | B | 3.420757 | 2.826953 | 2.596241 | 1.699962 | 0.000000 |
| 6 | B | 2.112205 | 2.600771 | 2.830381 | 2.903667 | 1.795391 |
| 7 | B | 3.419055 | 1.791720 | 1.835399 | 1.700495 | 1.835181 |
| 8 | B | 3.416748 | 2.830274 | 1.835857 | 1.701467 | 1.836146 |
| 9 | B | 2.108947 | 1.833933 | 2.829115 | 2.906487 | 1.800520 |
| 10 | B | 2.118141 | 1.848530 | 1.792346 | 2.898724 | 2.827998 |

| | | 6 | 7 | 8 | 9 | 10 |
|---|---|---|---|---|---|---|
| 6 | B | 0.000000 | | | | |
| 7 | B | 2.826267 | 0.000000 | | | |
| 8 | B | 1.800263 | 2.595749 | 0.000000 | | |
| 9 | B | 1.829634 | 1.801672 | 2.828013 | 0.000000 | |
| 10 | B | 1.843616 | 2.828473 | 1.789937 | 2.600460 | 0.000000 |

Fe1-C2: 1.737624 Fe1-C3: 1.737079 Fe1-C4: 1.738227

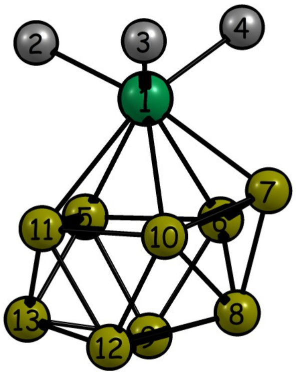


2. -1832.148901 +4.5 $C_s$

| | | 1 | 2 | 3 | 4 | 5 |
|---|---|---|---|---|---|---|
| 1 | Fe | 0.000000 | | | | |
| 2 | B | 2.262209 | 0.000000 | | | |
| 3 | B | 2.256509 | 1.800368 | 0.000000 | | |
| 4 | B | 2.061209 | 3.029969 | 1.763608 | 0.000000 | |
| 5 | B | 3.079737 | 2.815562 | 1.820556 | 1.688264 | 0.000000 |
| 6 | B | 3.373428 | 1.798052 | 1.804448 | 2.954035 | 1.786693 |
| 7 | B | 2.227536 | 2.897351 | 2.617369 | 1.747392 | 1.822554 |
| 8 | B | 2.236907 | 1.957082 | 2.894995 | 3.028742 | 2.824323 |
| 9 | B | 3.356562 | 2.605151 | 2.828829 | 2.947194 | 1.790063 |
| 10 | B | 3.391685 | 1.697163 | 2.914847 | 3.776483 | 2.895302 |

| | | 6 | 7 | 8 | 9 | 10 |
|---|---|---|---|---|---|---|
| 6 | B | 0.000000 | | | | |
| 7 | B | 2.830513 | 0.000000 | | | |
| 8 | B | 2.608481 | 1.813759 | 0.000000 | | |
| 9 | B | 1.815652 | 1.803781 | 1.803130 | 0.000000 | |
| 10 | B | 1.695453 | 2.919577 | 1.697418 | 1.696339 | 0.000000 |

Fe1-C2: 1.747424 Fe1-C3: 1.734432 Fe1-C4: 1.734400

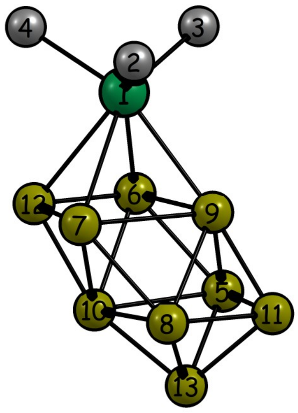


3. -1832.041887 +71.7 $C_\mathrm{s}$

| | | 1 | 2 | 3 | 4 | 5 |
|---|---|---|---|---|---|---|
| 1 | Fe | 0.000000 | | | | |
| 2 | B | 3.486624 | 0.000000 | | | |
| 3 | B | 2.134389 | 1.899273 | 0.000000 | | |
| 4 | B | 2.133498 | 3.110334 | 2.460973 | 0.000000 | |
| 5 | B | 3.489854 | 2.460773 | 3.107591 | 1.900626 | 0.000000 |
| 6 | B | 2.148086 | 1.888544 | 2.020586 | 2.014909 | 1.886725 |
| 7 | B | 3.251860 | 1.972085 | 1.944132 | 1.949225 | 1.968563 |
| 8 | B | 3.835231 | 1.702660 | 3.114754 | 3.113906 | 1.702314 |
| 9 | B | 2.199673 | 3.063489 | 1.663537 | 1.664009 | 3.060370 |
| 10 | B | 4.349787 | 1.700656 | 3.047196 | 3.052116 | 1.699937 |

| | | 6 | 7 | 8 | 9 | 10 |
|---|---|---|---|---|---|---|
| 6 | B | 0.000000 | | | | |
| 7 | B | 2.372420 | 0.000000 | | | |
| 8 | B | 1.692873 | 2.711676 | 0.000000 | | |
| 9 | B | 2.718514 | 1.759662 | 3.882259 | 0.000000 | |
| 10 | B | 2.603399 | 1.733494 | 1.740899 | 3.427601 | 0.000000 |

Fe1-C2: 1.749581 Fe1-C3: 1.749432 Fe1-C4: 1.726821

Table S13B: Energy ranking for the $[B_5H_5Fe(CO)_3]^{2-}$ structures at the PBE0/def2-TZVP level of theory.

| No. | Initial structure | Final Energy (a.u.) | Relative Energy (kcal/mol) |
|---|---|---|---|
| 1 | 10v-10-BisDisphenoid-Like—fe4—3co-dianion-tz.log | -1832.1561270 | 0.0 |
| 2 | 10v-01-BicapSqAntipr–fe1–3co-alter-dianion-tz.log | -1832.1561160 | 0.0 |
| 3 | 10v-05-PentAntiPr—fe1—3co-dianion-tz.log | -1832.1561060 | 0.0 |
| 4 | 10v-03-BicapCubB—fe1—3co-dianion-tz.log | -1832.1561030 | 0.0 |
| 5 | 10v-08-Isocloso—fe3—3co-dianion-tz.log | -1832.1561010 | 0.0 |
| 6 | 10v-08-Isocloso—fe2—3co-dianion-tz.log | -1832.1560930 | 0.0 |
| 7 | 10v-03-BicapCubB—fe4—3co-dianion-tz.log | -1832.1560850 | 0.0 |
| 8 | 10v-02-BicapCubA—fe1—3co-dianion-tz.log | -1832.1560820 | 0.0 |
| 9 | 10v-10-BisDisphenoid-Like—fe1—3co-dianion-tz.log | -1832.1560790 | 0.0 |
| 10 | 10v-07-TetrcTriPri2—fe2—3co-dianion-tz.log | -1832.1560670 | 0.0 |
| 11 | 10v-08-Isocloso—fe5—3co-dianion-tz.log | -1832.1560550 | 0.0 |
| 12 | 10v-07-TetrcTriPri2—fe6—3co-dianion-tz.log | -1832.1560470 | 0.1 |
| 13 | 10v-07-TetrcTriPri2—fe3—3co-dianion-tz.log | -1832.1560310 | 0.1 |
| 14 | 10v-01-BicapSqAntipr–fe1–3co-dianion-tz.log | -1832.1559730 | 0.1 |
| 15 | 10v-04-PentagPrism—fe1—3co-dianion-tz.log | -1832.1489010 | 4.5 |
| 16 | 10v-01-BicapSqAntipr–fe2–3co-dianion-tz.log | -1832.1488960 | 4.5 |
| 17 | 10v-08-Isocloso—fe1—3co-dianion-tz.log | -1832.1488930 | 4.5 |
| 18 | 10v-07-TetrcTriPri2—fe3—3co-alter-dianion-tz.log | -1832.1488880 | 4.5 |
| 19 | 10v-07-TetrcTriPri2—fe5—3co-dianion-tz.log | -1832.1488860 | 4.5 |
| 20 | 10v-03-BicapCubB—fe2—3co-dianion-tz.log | -1832.1488850 | 4.5 |
| 21 | 10v-08-Isocloso—fe4—3co-dianion-tz.log | -1832.1488720 | 4.6 |
| 22 | 10v-06-TetrcTriPri1—fe3—3co-dianion-tz.log | -1832.1488700 | 4.6 |
| 23 | 10v-07-TetrcTriPri2—fe1—3co-dianion-tz.log | -1832.1488590 | 4.6 |
| 24 | 10v-10-BisDisphenoid-Like—fe3—3co-dianion-tz.log | -1832.1488550 | 4.6 |
| 25 | 10v-10-BisDisphenoid-Like—fe5—3co-dianion-tz.log | -1832.1488530 | 4.6 |
| 26 | 10v-06-TetrcTriPri1—fe2—3co-dianion-tz.log | -1832.1488270 | 4.6 |
| 27 | 10v-10-BisDisphenoid-Like—fe2—3co-dianion-tz.log | -1832.1488260 | 4.6 |
| 28 | 10v-07-TetrcTriPri2—fe4—3co-dianion-tz.log | -1832.1488010 | 4.6 |
| 29 | 10v-01-BicapSqAntipr–fe2–3co-alter-dianion-tz.log | -1832.1470840 | 5.7 |
| 30 | 10v-09-DistTetracapOh—fe2—3co-dianion-tz.log | -1832.0907000 | 41.1 |
| 31 | 10v-09-DistTetracapOh—fe3—3co-dianion-tz.log | -1832.0862500 | 43.8 |
| 32 | 10v-03-BicapCubB—fe3—3co-dianion-tz.log | -1832.0594880 | 60.6 |
| 33 | 10v-06-TetrcTriPri1—fe1—3co-dianion-tz.log | -1832.0501410 | 66.5 |
| 34 | 10v-09-DistTetracapOh—fe1—3co-dianion-tz.log | -1832.0418870 | 71.7 |
| 35 | 10v-02-BicapCubA—fe2—3co-dianion-tz.log | -1832.0417810 | 71.8 |

Table S14A: Distance table for the lowest-lying $[B_5H_5Fe(CO)_3]^{2-}$ optimized structures obtained at the PBE0/def2-TZVP level of theory. Included are the zero-point corrected absolute energy in (a.u.) at the DLPNO-CCSD(T)/def2-QZVP level of theory with zero-point energy obtained from the PBE0/def2-TZVP computations, relative energies in (kcal/mol) and symmetry. For clarity, only the atoms forming the cluster framework are shown.

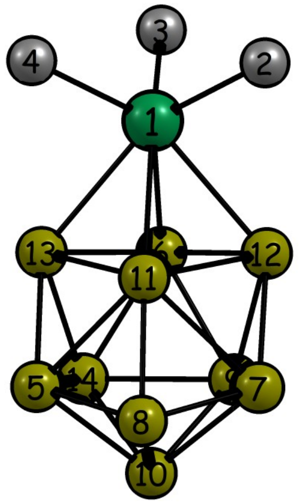


1. -1857.575026 0.0 $C_s$

| | | 1 | 2 | 3 | 4 | 5 |
|---|---|---|---|---|---|---|
| 1 | Fe | 0.000000 | | | | |
| 2 | B | 3.449835 | 0.000000 | | | |
| 3 | B | 2.197007 | 2.932171 | 0.000000 | | |
| 4 | B | 3.444723 | 2.632410 | 2.930214 | 0.000000 | |
| 5 | B | 3.837288 | 1.676378 | 3.631392 | 1.675655 | 0.000000 |
| 6 | B | 3.402265 | 2.813969 | 1.781517 | 1.787019 | 2.880677 |
| 7 | B | 4.162397 | 1.793626 | 2.964111 | 1.794683 | 1.745439 |
| 8 | B | 2.167946 | 1.939470 | 2.701716 | 1.942757 | 1.725163 |
| 9 | B | 2.084911 | 3.216638 | 1.782957 | 1.818753 | 3.024289 |
| 10 | B | 2.086620 | 1.817889 | 1.784141 | 3.210297 | 3.019744 |
| 11 | B | 3.403139 | 1.786457 | 1.781484 | 2.812839 | 2.880262 |

| | | 6 | 7 | 8 | 9 | 10 |
|---|---|---|---|---|---|---|
| 6 | B | 0.000000 | | | | |
| 7 | B | 1.778243 | 0.000000 | | | |
| 8 | B | 2.780102 | 2.638949 | 0.000000 | | |
| 9 | B | 1.765386 | 2.931302 | 2.033926 | 0.000000 | |
| 10 | B | 2.810359 | 2.928685 | 2.022662 | 2.666798 | 0.000000 |
| 11 | B | 1.794103 | 1.778031 | 2.776490 | 2.811383 | 1.765332 |

| | | 11 |
|---|---|---|
| 11 | B | 0.000000 |

Fe1-C2: 1.746804 Fe1-C3: 1.730966 Fe1-C4: 1.747137

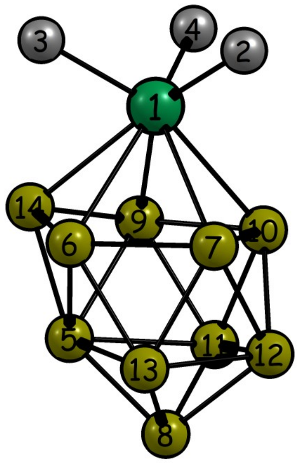


2. -1857.560318 +7.3 $C_s$

| | | 1 | 2 | 3 | 4 | 5 |
|---|---|---|---|---|---|---|
| 1 | Fe | 0.000000 | | | | |
| 2 | B | 3.194672 | 0.000000 | | | |
| 3 | B | 2.210086 | 1.966827 | 0.000000 | | |
| 4 | B | 2.181597 | 2.778591 | 1.817576 | 0.000000 | |
| 5 | B | 4.154675 | 1.730456 | 2.985391 | 2.862765 | 0.000000 |
| 6 | B | 2.192169 | 1.979041 | 2.677667 | 2.881883 | 2.978426 |
| 7 | B | 2.174643 | 2.796410 | 2.884925 | 1.856275 | 2.860134 |
| 8 | B | 3.436508 | 1.999228 | 3.243061 | 2.838301 | 1.666921 |
| 9 | B | 3.438386 | 2.664952 | 2.957294 | 1.776538 | 1.751428 |
| 10 | B | 3.452375 | 1.969826 | 1.838495 | 1.772690 | 1.668266 |
| 11 | B | 2.140141 | 1.729070 | 1.703882 | 2.942759 | 3.343833 |

| | | 6 | 7 | 8 | 9 | 10 |
|---|---|---|---|---|---|---|
| 6 | B | 0.000000 | | | | |
| 7 | B | 1.839581 | 0.000000 | | | |
| 8 | B | 1.842215 | 1.767743 | 0.000000 | | |
| 9 | B | 2.963674 | 1.772640 | 1.790852 | 0.000000 | |
| 10 | B | 3.237390 | 2.841224 | 2.656199 | 1.791444 | 0.000000 |
| 11 | B | 1.682160 | 2.949220 | 3.009500 | 3.638325 | 3.011367 |

| | | 11 |
|---|---|---|
| 11 | B | 0.000000 |

Fe1-C2: 1.741796 Fe1-C3: 1.739786 Fe1-C4: 1.741392

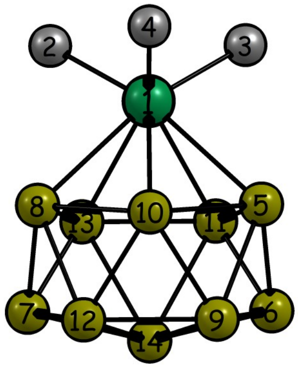


3. -1857.558911 +8.2 $C_s$

| | | 1 | 2 | 3 | 4 | 5 |
|---|---|---|---|---|---|---|
| 1 | Fe | 0.000000 | | | | |
| 2 | B | 2.192084 | 0.000000 | | | |
| 3 | B | 3.344184 | 1.735341 | 0.000000 | | |
| 4 | B | 3.344582 | 3.644824 | 3.349745 | 0.000000 | |
| 5 | B | 2.192364 | 3.066483 | 3.644587 | 1.735648 | 0.000000 |
| 6 | B | 3.350500 | 1.772409 | 1.661056 | 2.980296 | 2.957274 |
| 7 | B | 2.223715 | 1.814715 | 2.870318 | 2.869748 | 1.813375 |
| 8 | B | 2.186206 | 1.880916 | 1.672027 | 2.991675 | 3.028491 |
| 9 | B | 3.351492 | 2.958323 | 2.980829 | 1.661225 | 1.772783 |
| 10 | B | 2.186831 | 3.028438 | 2.991192 | 1.671772 | 1.881275 |
| 11 | B | 3.324525 | 2.681849 | 1.739958 | 1.739464 | 2.681541 |

| | | 6 | 7 | 8 | 9 | 10 |
|---|---|---|---|---|---|---|
| 6 | B | 0.000000 | | | | |
| 7 | B | 1.781040 | 0.000000 | | | |
| 8 | B | 2.670984 | 2.881339 | 0.000000 | | |
| 9 | B | 1.828308 | 1.781159 | 3.239945 | 0.000000 | |
| 10 | B | 3.238838 | 2.880971 | 1.836984 | 2.671331 | 0.000000 |
| 11 | B | 1.966692 | 2.769710 | 1.968728 | 1.966848 | 1.967899 |

| | | 11 |
|---|---|---|
| 11 | B | 0.000000 |

Fe1-C2: 1.739414 Fe1-C3: 1.739278 Fe1-C4: 1.740990

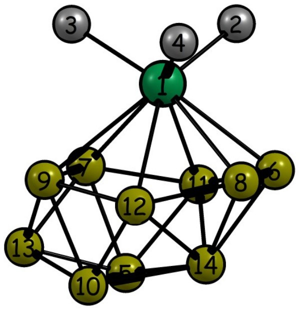


4. -1857.517584 +34.1 $C_s$

| | | 1 | 2 | 3 | 4 | 5 |
|---|---|---|---|---|---|---|
| 1 | Fe | 0.000000 | | | | |
| 2 | B | 3.347898 | 0.000000 | | | |
| 3 | B | 2.238438 | 3.019733 | 0.000000 | | |
| 4 | B | 2.379469 | 1.778176 | 3.178655 | 0.000000 | |
| 5 | B | 2.237833 | 3.460973 | 1.610547 | 3.640549 | 0.000000 |
| 6 | B | 2.378393 | 2.576649 | 3.640410 | 1.956819 | 3.177930 |
| 7 | B | 3.346943 | 1.776969 | 3.461226 | 2.576131 | 3.019247 |
| 8 | B | 2.311324 | 1.793167 | 1.699471 | 1.782306 | 2.791144 |
| 9 | B | 2.308147 | 2.937005 | 2.790523 | 3.021171 | 1.698678 |
| 10 | B | 3.494861 | 1.691680 | 4.133396 | 1.681420 | 4.133092 |
| 11 | B | 2.887530 | 1.876163 | 1.842845 | 2.848485 | 1.843574 |

| | | 6 | 7 | 8 | 9 | 10 |
|---|---|---|---|---|---|---|
| 6 | B | 0.000000 | | | | |
| 7 | B | 1.777428 | 0.000000 | | | |
| 8 | B | 3.022050 | 2.936714 | 0.000000 | | |
| 9 | B | 1.781016 | 1.793878 | 3.043622 | 0.000000 | |
| 10 | B | 1.681847 | 1.691333 | 2.945741 | 2.945741 | 0.000000 |
| 11 | B | 2.849060 | 1.877519 | 1.962615 | 1.965045 | 2.997987 |

| | | 11 |
|---|---|---|
| 11 | B | 0.000000 |

Fe1-C2: 1.737638 Fe1-C3: 1.741035 Fe1-C4: 1.737551

Table S14B: Energy ranking for the $[B_5H_5Fe(CO)_3]^{2-}$ structures at the PBE0/def2-TZVP level of theory.

| No. | Initial structure | Final Energy (a.u.) | Relative Energy (kcal/mol) |
|---|---|---|---|
| 1 | 11v-03-CapPentAnPr–fe3–3co-dianion-tz.log | -1857.5750260 | 0.0 |
| 2 | 11v-05-TricapCubeB–fe4–3co-dianion-tz.log | -1857.5749800 | 0.0 |
| 3 | 11v-01-EdgeCoalscedIh–fe1–3co-dianion-tz.log | -1857.5749710 | 0.0 |
| 4 | 11v-04-TricapCubeA–fe1–3co-dianion-tz.log | -1857.5749380 | 0.1 |
| 5 | 11v-05-TricapCubeB–fe2–3co-dianion-tz.log | -1857.5719680 | 0.0 |
| 6 | 11v-01-EdgeCoalscedIh–fe4–3co-dianion-tz.log | -1857.5719640 | 0.0 |
| 7 | 11v-04-TricapCubeA–fe1–3co-alter1-dianion-tz.log | -1857.5719480 | 0.0 |
| 8 | 11v-06-CapPentPrA–fe1–3co-dianion-tz.log | -1857.5719410 | 0.0 |
| 9 | 11v-05-TricapCubeB–fe1–3co-dianion-tz.log | -1857.5719320 | 0.0 |
| 10 | 11v-04-TricapCubeA–fe3–3co-dianion-tz.log | -1857.5719190 | 0.0 |
| 11 | 11v-05-TricapCubeB–fe3–3co-dianion-tz.log | -1857.5719140 | 0.0 |
| 12 | 11v-04-TricapCubeA–fe1–3co-alter2-dianion-tz.log | -1857.5718930 | 0.0 |
| 13 | 11v-01-EdgeCoalscedIh–fe2–3co-dianion-tz.log | -1857.5718730 | 0.1 |
| 14 | 11v-04-TricapCubeA–fe4–3co-dianion-tz.log | -1857.5603180 | 7.3 |
| 15 | 11v-01-EdgeCoalscedIh–fe3–3co-dianion-tz.log | -1857.5603060 | 7.3 |
| 16 | 11v-02-PentaCapTriPri–fe2–3co-alter2-dianion-tz.log | -1857.5602040 | 7.4 |
| 17 | 11v-01-EdgeCoalscedIh–fe5–3co-dianion-tz.log | -1857.5589110 | 8.2 |
| 18 | 11v-03-CapPentAnPr–fe1–3co-dianion-tz.log | -1857.5587990 | 8.3 |
| 19 | 11v-02-PentaCapTriPri–fe2–3co-alter1-dianion-tz.log | -1857.5512590 | 13.0 |
| 20 | 11v-02-PentaCapTriPri–fe1–3co-dianion-tz.log | -1857.5175840 | 34.1 |
| 21 | 11v-04-TricapCubeA–fe2–3co-dianion-tz.log | -1857.5109240 | 38.3 |
| 22 | 11v-03-CapPentAnPr–fe2–3co-dianion-tz.log | -1857.4730800 | 62.1 |
| 23 | 11v-02-PentaCapTriPri–fe2–3co-dianion-tz.log | -1857.4073410 | 103.3 |

Table S15A: Distance table for the lowest-lying $[B_5H_5Fe(CO)_3]^{2-}$ optimized structures obtained at the PBE0/def2-TZVP level of theory. Included are the zero-point corrected absolute energy in (a.u.) at the DLPNO-CCSD(T)/def2-QZVP level of theory with zero-point energy obtained from the PBE0/def2-TZVP computations, relative energies in (kcal/mol) and symmetry. For clarity, only the atoms forming the cluster framework are shown.

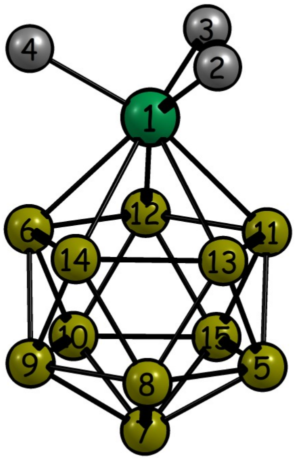


1. -1883.086810 0.0 $C_s$

| | | 1 | 2 | 3 | 4 | 5 |
|---|---|---|---|---|---|---|
| 1 | Fe | 0.000000 | | | | |
| 2 | B | 3.389778 | 0.000000 | | | |
| 3 | B | 2.184722 | 3.406451 | 0.000000 | | |
| 4 | B | 3.963625 | 1.770777 | 2.879157 | 0.000000 | |
| 5 | B | 3.388510 | 1.778025 | 2.896700 | 1.772483 | 0.000000 |
| 6 | B | 3.386301 | 2.871786 | 1.773060 | 1.775324 | 1.772532 |
| 7 | B | 3.395184 | 2.869242 | 1.771215 | 1.769390 | 2.870508 |
| 8 | B | 2.183319 | 1.771683 | 2.949841 | 2.882678 | 2.895409 |
| 9 | B | 2.185931 | 2.898548 | 1.808176 | 2.880740 | 3.408591 |
| 10 | B | 2.179431 | 1.772633 | 2.949981 | 2.878167 | 1.773588 |
| 11 | B | 2.183760 | 2.897850 | 1.835320 | 2.884089 | 1.771870 |
| 12 | B | 3.385972 | 1.772773 | 2.886871 | 1.776461 | 2.873507 |
| | | 6 | 7 | 8 | 9 | 10 |
| 6 | B | 0.000000 | | | | |
| 7 | B | 1.775956 | 0.000000 | | | |
| 8 | B | 3.408958 | 2.900740 | 0.000000 | | |
| 9 | B | 2.888906 | 1.771587 | 1.836461 | 0.000000 | |
| 10 | B | 2.890727 | 3.406445 | 1.817166 | 2.950421 | 0.000000 |
| 11 | B | 1.772331 | 2.902138 | 2.953431 | 2.952975 | 1.823000 |
| 12 | B | 2.873866 | 1.775069 | 1.771730 | 1.772893 | 2.888686 |
| | | 11 | 12 | | | |
| 11 | B | 0.000000 | | | | |
| 12 | B | 3.410970 | 0.000000 | | | |

Fe1-C2: 1.742547 Fe1-C3: 1.741838 Fe1-C4: 1.742253

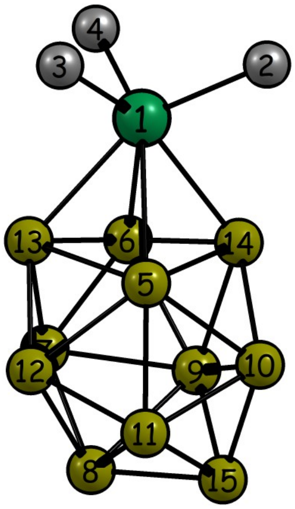


2. -1882.969894 +73.4 $C_1$

| | | 1 | 2 | 3 | 4 | 5 |
|---|---|---|---|---|---|---|
| 1 | Fe | 0.000000 | | | | |
| 2 | B | 2.295797 | 0.000000 | | | |
| 3 | B | 2.194478 | 2.656917 | 0.000000 | | |
| 4 | B | 3.393851 | 2.769938 | 1.743112 | 0.000000 | |
| 5 | B | 4.474359 | 2.893156 | 3.092068 | 1.782885 | 0.000000 |
| 6 | B | 3.516820 | 2.725657 | 1.854024 | 1.915907 | 2.002678 |
| 7 | B | 3.434486 | 1.857105 | 3.000251 | 3.179885 | 2.655002 |
| 8 | B | 4.065323 | 1.870098 | 3.568533 | 2.858736 | 1.790535 |
| 9 | B | 3.465370 | 1.889447 | 2.866682 | 1.779395 | 1.764422 |
| 10 | B | 2.095886 | 2.001642 | 1.816731 | 1.732462 | 2.981556 |
| 11 | B | 2.028971 | 1.961303 | 1.762220 | 2.885935 | 3.389157 |
| 12 | B | 4.631518 | 2.928998 | 3.475715 | 2.940407 | 1.671235 |

| | | 6 | 7 | 8 | 9 | 10 |
|---|---|---|---|---|---|---|
| 6 | B | 0.000000 | | | | |
| 7 | B | 1.956656 | 0.000000 | | | |
| 8 | B | 2.645689 | 1.791557 | 0.000000 | | |
| 9 | B | 2.773365 | 2.859150 | 1.732159 | 0.000000 | |
| 10 | B | 2.887451 | 3.331725 | 3.084068 | 1.756216 | 0.000000 |
| 11 | B | 1.916817 | 1.776210 | 3.058617 | 3.184648 | 2.631484 |
| 12 | B | 1.799827 | 1.643544 | 1.733326 | 2.847967 | 3.838215 |

| | | 11 | 12 |
|---|---|---|---|
| 11 | B | 0.000000 | |
| 12 | B | 2.973570 | 0.000000 |

Fe1-C2: 1.752618 Fe1-C3: 1.742393 Fe1-C4: 1.737185

Table S15B: Energy ranking for the $[B_5H_5Fe(CO)_3]^{2-}$ structures at the PBE0/def2-TZVP level of theory.

| No. | Initial structure | Final Energy (a.u.) | Relative Energy (kcal/mol) |
|---|---|---|---|
| 1 | 12v-02-CubeOh–fe1–3co-dianion-tz.log | -1883.0868100 | 0.0 |
| 2 | 12v-07-TetraCappedCube-D4h–fe1–3co-dianion-tz.log | -1883.0868100 | 0.0 |
| 3 | 12v-09-2v6-Dh–fe1–3co-alter2-dianion-tz.log | -1883.0868030 | 0.0 |
| 4 | 12v-06-DsqAnPr–fe2–3co-dianion-tz.log | -1883.0868010 | 0.0 |
| 5 | 12v-08-TetraCappedCube-C2v–fe4–3co-dianion-tz.log | -1883.0867940 | 0.0 |
| 6 | 12v-09-2v6-Dh–fe2–3co-dianion-tz.log | -1883.0867800 | 0.0 |
| 7 | 12v-09-2v6-Dh–fe1–3co-dianion-tz.log | -1883.0867670 | 0.0 |
| 8 | 12v-09-2v6-Dh–fe3–3co-dianion-tz.log | -1883.0867390 | 0.0 |
| 9 | 12v-01-Icos–fe1–3co-dianion-tz.log | -1883.0867330 | 0.0 |
| 10 | 12v-05-HexAnPr–fe–3co-dianion-tz.log | -1883.0146250 | 45.3 |
| 11 | 12v-06-DsqAnPr–fe1–3co-dianion-tz.log | -1882.9859130 | 63.3 |
| 12 | 12v-08-TetraCappedCube-C2v–fe1–3co-dianion-tz.log | -1882.9727730 | 71.6 |
| 13 | 12v-07-TetraCappedCube-D4h–fe2–3co-dianion-tz.log | -1882.9698940 | 73.4 |
| 14 | 12v-08-TetraCappedCube-C2v–fe2–3co-dianion-tz.log | -1882.9698700 | 73.4 |
| 15 | 12v-08-TetraCappedCube-C2v–fe3–3co-dianion-tz.log | -1882.9698560 | 73.4 |
| 16 | 12v-04-HexPr–fe–3co-dianion-tz.log | -1882.9552610 | 82.5 |
| 17 | 12v-02-CubeOh–fe2–3co-dianion-tz.log | -1882.9552540 | 82.6 |
| 18 | 12v-03-AnticubeOh–fe1–3co-dianion-tz.log | -1882.9425230 | 90.5 |
| 19 | 12v-03-AnticubeOh–fe2–3co-dianion-tz.log | -1882.9350630 | 95.2 |

Table S16A: Distance table for the lowest-lying $[B_5H_5Fe(CO)_3]^{2-}$ optimized structures obtained at the PBE0/def2-TZVP level of theory. Included are the zero-point corrected absolute energy in (a.u.) at the DLPNO-CCSD(T)/def2-QZVP level of theory with zero-point energy obtained from the PBE0/def2-TZVP computations, relative energies in (kcal/mol) and symmetry. For clarity, only the atoms forming the cluster framework are shown.

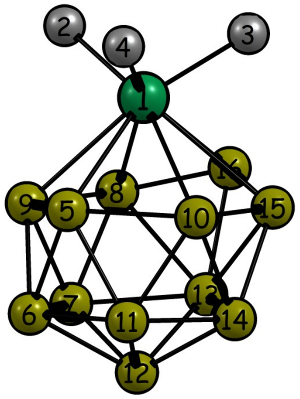


1. -1908.534041 0.0 $C_s$

| | | 1 | 2 | 3 | 4 | 5 |
|---|---|---|---|---|---|---|
| 1 | Fe | 0.000000 | | | | |
| 2 | B | 2.225123 | 0.000000 | | | |
| 3 | B | 3.430938 | 1.783278 | 0.000000 | | |
| 4 | B | 3.359865 | 2.851390 | 1.740764 | 0.000000 | |
| 5 | B | 2.318315 | 3.117985 | 2.999278 | 1.777219 | 0.000000 |
| 6 | B | 2.274128 | 1.793423 | 1.744345 | 1.752064 | 1.846371 |
| 7 | B | 2.295070 | 1.822075 | 2.915927 | 3.484034 | 3.455823 |
| 8 | B | 3.431446 | 1.782157 | 1.771353 | 2.881284 | 3.734476 |
| 9 | B | 3.861491 | 2.846617 | 1.787678 | 1.744434 | 3.025934 |
| 10 | B | 3.174752 | 3.352149 | 2.941612 | 1.903996 | 1.991325 |
| 11 | B | 3.397740 | 2.870984 | 2.886272 | 2.986634 | 3.415010 |
| 12 | B | 2.380865 | 3.144443 | 3.733952 | 3.379546 | 2.812529 |
| 13 | B | 2.211394 | 3.595797 | 3.920633 | 2.938504 | 1.643612 |

| | | 6 | 7 | 8 | 9 | 10 |
|---|---|---|---|---|---|---|
| 6 | B | 0.000000 | | | | |
| 7 | B | 3.063548 | 0.000000 | | | |
| 8 | B | 2.904185 | 1.744003 | 0.000000 | | |
| 9 | B | 2.846274 | 2.840178 | 1.792911 | 0.000000 | |
| 10 | B | 2.925000 | 2.896561 | 2.935107 | 1.806368 | 0.000000 |
| 11 | B | 3.505340 | 1.748727 | 1.738437 | 1.743466 | 1.902687 |
| 12 | B | 3.477284 | 1.842461 | 2.988634 | 2.997748 | 1.941312 |
| 13 | B | 3.067677 | 3.061490 | 3.921684 | 3.412315 | 1.819346 |

| | | 11 | 12 | 13 |
|---|---|---|---|---|
| 11 | B | 0.000000 | | |
| 12 | B | 1.768298 | 0.000000 | |
| 13 | B | 2.973773 | 1.666867 | 0.000000 |

Fe1-C2: 1.743468 Fe1-C3: 1.748213 Fe1-C4: 1.742117

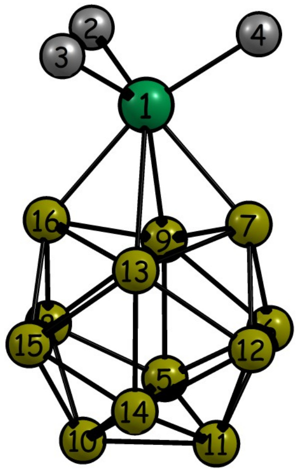


2. -1908.441579 +4.9 $C_s$

| | | 1 | 2 | 3 | 4 | 5 |
|---|---|---|---|---|---|---|
| 1 | Fe | 0.000000 | | | | |
| 2 | B | 4.085255 | 0.000000 | | | |
| 3 | B | 3.525126 | 1.737198 | 0.000000 | | |
| 4 | B | 2.055317 | 2.987989 | 1.758854 | 0.000000 | |
| 5 | B | 3.482854 | 1.741814 | 2.927744 | 3.276085 | 0.000000 |
| 6 | B | 2.397991 | 1.850945 | 1.876684 | 1.927197 | 1.876098 |
| 7 | B | 4.524990 | 1.787179 | 2.866827 | 3.633007 | 1.751884 |
| 8 | B | 4.547162 | 1.785452 | 1.752098 | 2.935876 | 2.869492 |
| 9 | B | 3.523576 | 2.839199 | 1.770657 | 1.758124 | 3.423776 |
| 10 | B | 2.394168 | 3.335052 | 2.882252 | 1.926057 | 2.885487 |
| 11 | B | 4.080410 | 2.847358 | 2.839045 | 2.985999 | 2.843765 |
| 12 | B | 3.481044 | 2.844490 | 3.423671 | 3.276400 | 1.775608 |
| 13 | B | 2.029409 | 3.015985 | 3.326108 | 2.668955 | 1.753319 |

| | | 6 | 7 | 8 | 9 | 10 |
|---|---|---|---|---|---|---|
| 6 | B | 0.000000 | | | | |
| 7 | B | 2.950461 | 0.000000 | | | |
| 8 | B | 2.952613 | 1.761381 | 0.000000 | | |
| 9 | B | 2.882491 | 2.867187 | 1.751286 | 0.000000 | |
| 10 | B | 2.702817 | 2.950593 | 2.951736 | 1.876876 | 0.000000 |
| 11 | B | 3.333438 | 1.787136 | 1.785544 | 1.737812 | 1.848410 |
| 12 | B | 2.884301 | 1.751440 | 2.869666 | 2.929864 | 1.878086 |
| 13 | B | 1.967549 | 2.941877 | 3.671151 | 3.327602 | 1.970693 |

| | | 11 | 12 | 13 |
|---|---|---|---|---|
| 11 | B | 0.000000 | | |
| 12 | B | 1.741444 | 0.000000 | |
| 13 | B | 3.015249 | 1.754253 | 0.000000 |

Fe1-C2: 1.739963 Fe1-C3: 1.740570 Fe1-C4: 1.775943

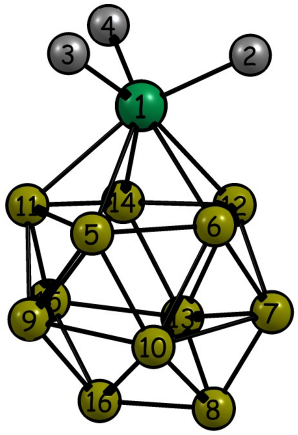


3. -1908.432356 +10.6 $C_s$

| | | 1 | 2 | 3 | 4 | 5 |
|---|---|---|---|---|---|---|
| 1 | Fe | 0.000000 | | | | |
| 2 | B | 2.212144 | 0.000000 | | | |
| 3 | B | 2.142030 | 1.747852 | 0.000000 | | |
| 4 | B | 3.437355 | 3.037445 | 1.778504 | 0.000000 | |
| 5 | B | 4.404916 | 3.396787 | 2.903942 | 1.614703 | 0.000000 |
| 6 | B | 3.393241 | 1.750699 | 2.992718 | 3.425123 | 2.956400 |
| 7 | B | 3.496667 | 1.823611 | 1.911953 | 2.021808 | 1.828362 |
| 8 | B | 2.176472 | 1.852425 | 2.954507 | 3.762668 | 3.878224 |
| 9 | B | 2.141004 | 2.894240 | 1.834333 | 1.772468 | 2.910875 |
| 10 | B | 3.494483 | 3.321518 | 2.917266 | 2.003110 | 1.831112 |
| 11 | B | 2.209855 | 2.892202 | 2.890611 | 3.030114 | 3.400938 |
| 12 | B | 3.393650 | 2.850630 | 3.485357 | 3.416837 | 2.954153 |
| 13 | B | 4.253680 | 2.982791 | 3.343858 | 2.778728 | 1.623588 |

| | | 6 | 7 | 8 | 9 | 10 |
|---|---|---|---|---|---|---|
| 6 | B | 0.000000 | | | | |
| 7 | B | 1.905536 | 0.000000 | | | |
| 8 | B | 1.733324 | 2.976237 | 0.000000 | | |
| 9 | B | 3.491729 | 2.926438 | 2.955807 | 0.000000 | |
| 10 | B | 2.882304 | 2.662508 | 2.976798 | 1.910780 | 0.000000 |
| 11 | B | 2.848695 | 3.321537 | 1.845338 | 1.751224 | 1.827186 |
| 12 | B | 1.748797 | 2.876604 | 1.733457 | 2.994632 | 1.907646 |
| 13 | B | 1.766690 | 1.922218 | 2.945655 | 3.352439 | 1.934512 |

| | | 11 | 12 | 13 |
|---|---|---|---|---|
| 11 | B | 0.000000 | | |
| 12 | B | 1.749435 | 0.000000 | |
| 13 | B | 2.988828 | 1.769229 | 0.000000 |

Fe1-C2: 1.744988 Fe1-C3: 1.745087 Fe1-C4: 1.744905

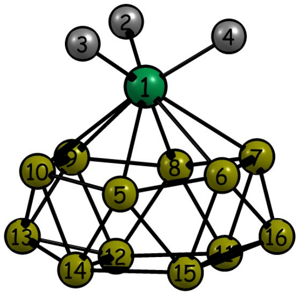


4. -1908.382766 +41.8 $C_s$

| | | 1 | 2 | 3 | 4 | 5 |
|---|---|---|---|---|---|---|
| 1 | Fe | 0.000000 | | | | |
| 2 | B | 2.260242 | 0.000000 | | | |
| 3 | B | 2.304426 | 1.750982 | 0.000000 | | |
| 4 | B | 2.329525 | 3.098362 | 1.978576 | 0.000000 | |
| 5 | B | 2.260446 | 3.186110 | 3.019488 | 1.745238 | 0.000000 |
| 6 | B | 2.314127 | 2.995909 | 3.730841 | 3.251407 | 1.774341 |
| 7 | B | 2.336394 | 1.774726 | 3.253771 | 3.839769 | 3.067921 |
| 8 | B | 3.326998 | 3.164250 | 2.669511 | 1.785165 | 1.786103 |
| 9 | B | 3.283662 | 2.984644 | 3.510442 | 3.038994 | 1.829562 |
| 10 | B | 3.423629 | 2.891143 | 4.137855 | 4.205592 | 2.942426 |
| 11 | B | 3.331257 | 1.778379 | 3.072106 | 3.751985 | 3.169710 |
| 12 | B | 3.277178 | 1.837676 | 1.832368 | 2.658424 | 2.977078 |
| 13 | B | 3.380244 | 2.933003 | 1.669771 | 1.675805 | 2.877561 |

| | | 6 | 7 | 8 | 9 | 10 |
|---|---|---|---|---|---|---|
| 6 | B | 0.000000 | | | | |
| 7 | B | 1.897711 | 0.000000 | | | |
| 8 | B | 3.086045 | 3.734730 | 0.000000 | | |
| 9 | B | 1.835144 | 2.638816 | 1.881625 | 0.000000 | |
| 10 | B | 1.670326 | 1.674546 | 3.445597 | 1.706545 | 0.000000 |
| 11 | B | 2.649098 | 1.787125 | 2.927690 | 1.957124 | 1.670025 |
| 12 | B | 3.498124 | 3.054622 | 1.935320 | 2.469940 | 3.258758 |
| 13 | B | 4.130159 | 4.199225 | 1.674772 | 3.263930 | 4.482609 |

| | | 11 | 12 | 13 |
|---|---|---|---|---|
| 11 | B | 0.000000 | | |
| 12 | B | 1.888325 | 0.000000 | |
| 13 | B | 3.455622 | 1.711740 | 0.000000 |

Fe1-C2: 1.744988 Fe1-C3: 1.745087 Fe1-C4: 1.744905

Table S16BH: Energy ranking for the $[B_5H_5Fe(CO)_3]^{2-}$ structures at the PBE0/def2-TZVP level of theory.

| No. | Initial structure | Final Energy (a.u.) | Relative Energy (kcal/mol) |
|---|---|---|---|
| 1 | 13v-03-DitrapIcos-fe3-3co-dianion-tz.log | -1908.5340410 | 0.0 |
| 2 | 13v-01-Doc-fe2-3co-dianion-tz.log | -1908.4493260 | 0.0 |
| 3 | 13v-02-Hen-fe2-3co-dianion-tz.log | -1908.4493030 | 0.0 |
| 4 | 13v-02-Hen-fe3-3co-dianion-tz.log | -1908.4415790 | 4.9 |
| 5 | 13v-02-Hen-fe4-3co-dianion-tz.log | -1908.4415000 | 4.9 |
| 6 | 13v-01-Doc-fe3-3co-dianion-tz.log | -1908.4414830 | 4.9 |
| 7 | 13v-03-DitrapIcos-fe2-3co-dianion-tz.log | -1908.4414440 | 4.9 |
| 8 | 13v-03-DitrapIcos-fe1-3co-dianion-tz.log | -1908.4323560 | 10.6 |
| 9 | 13v-02-Hen-fe1-3co-dianion-tz.log | -1908.4323270 | 10.7 |
| 10 | 13v-01-Doc-fe1-3co-dianion-tz.log | -1908.4287080 | 12.9 |
| 11 | 13v-04-CapHexAnPr-a-fe3-3co-dianion-tz.log | -1908.3846650 | 40.6 |
| 12 | 13v-04-CapHexAnPr-a-fe1-3co-dianion-tz.log | -1908.3827660 | 41.8 |
| 13 | 13v-04-CapHexAnPr-a-fe2-3co-dianion-tz.log | -1908.3539300 | 59.9 |

HOMO-LUMO gaps for the lowest-lying $(CO)_mFeB_nH_n$ structures (m = 2, 3, 4; n = 5 - 12) as computed using the PBE0/def2-TZVP level of theory.

| Structure | HOMO (a.u.) | LUMO (a.u.) | H/L gap (a.u.) | H/L gap (eV) |
|---|---|---|---|---|
| $(CO)_2$FeB5-1 | -0.319712 | -0.203394 | 0.116318 | 3.17 |
| $(CO)_2$FeB5-2 | -0.290077 | -0.223002 | 0.067075 | 1.83 |
| $(CO)_2$FeB5-3 | -0.288589 | -0.227327 | 0.061262 | 1.67 |
| $(CO)_2$FeB5-4 | -0.288540 | -0.249247 | 0.039293 | 1.07 |
| $(CO)_2$FeB5-5 | -0.292373 | -0.210300 | 0.082073 | 2.23 |
| $(CO)_3$FeB5-1 | -0.307032 | -0.222462 | 0.084570 | 2.30 |
| $(CO)_3$FeB5-2 | -0.310481 | -0.195695 | 0.114786 | 3.12 |
| $(CO)_3$FeB5-3 | -0.303622 | -0.267197 | 0.036425 | 0.99 |
| $(CO)_4$FeB5-1 | -0.305524 | -0.192623 | 0.112901 | 3.07 |
| $(CO)_2$FeB6-1 | -0.300297 | -0.228350 | 0.071947 | 1.96 |
| $(CO)_2$FeB6-2 | -0.285685 | -0.217346 | 0.068339 | 1.86 |
| $(CO)_2$FeB6-3 | -0.301363 | -0.225955 | 0.075408 | 2.05 |
| $(CO)_2$FeB6-4 | -0.288382 | -0.220682 | 0.067700 | 1.84 |
| $(CO)_2$FeB6-5 | -0.291387 | -0.217102 | 0.074285 | 2.02 |
| $(CO)_3$FeB6-1 | -0.310410 | -0.204716 | 0.105694 | 2.88 |
| $(CO)_3$FeB6-2 | -0.308846 | -0.210981 | 0.097865 | 2.66 |
| $(CO)_3$FeB6-3 | -0.306851 | -0.172929 | 0.133922 | 3.64 |
| $(CO)_3$FeB6-4 | -0.305970 | -0.218658 | 0.087312 | 2.38 |
| $(CO)_3$FeB6-5 | -0.307599 | -0.184825 | 0.122774 | 3.34 |
| $(CO)_4$FeB6-1 | -0.307726 | -0.216657 | 0.091069 | 2.48 |
| $(CO)_4$FeB6-2 | -0.304712 | -0.202728 | 0.101984 | 2.78 |
| $(CO)_4$FeB6-3 | -0.307530 | -0.186644 | 0.120886 | 3.29 |
| $(CO)_4$FeB6-4 | -0.301509 | -0.207469 | 0.094040 | 2.56 |
| $(CO)_2$FeB7-1 | -0.316548 | -0.228021 | 0.088527 | 2.41 |
| $(CO)_2$FeB7-2 | -0.298932 | -0.229137 | 0.069795 | 1.90 |
| $(CO)_2$FeB7-3 | -0.300693 | -0.253282 | 0.047411 | 1.29 |
| $(CO)_2$FeB7-4 | -0.310490 | -0.215820 | 0.094670 | 2.58 |
| $(CO)_2$FeB7-5 | -0.301356 | -0.173494 | 0.127862 | 3.48 |
| $(CO)_2$FeB7-6 | -0.294089 | -0.254542 | 0.039547 | 1.08 |
| $(CO)_2$FeB7-7 | -0.285464 | -0.238489 | 0.046975 | 1.28 |
| $(CO)_3$FeB7-1 | -0.312679 | -0.200024 | 0.112655 | 3.07 |
| $(CO)_3$FeB7-2 | -0.313520 | -0.263001 | 0.050519 | 1.37 |
| $(CO)_3$FeB7-3 | -0.314132 | -0.265725 | 0.048407 | 1.32 |
| $(CO)_3$FeB7-4 | -0.306885 | -0.217326 | 0.089559 | 2.44 |
| $(CO)_3$FeB7-5 | -0.307697 | -0.204339 | 0.103358 | 2.81 |
| $(CO)_3$FeB7-6 | -0.306200 | -0.233296 | 0.072904 | 1.98 |
| $(CO)_4$FeB7-1 | -0.308250 | -0.197020 | 0.111230 | 3.03 |
| $(CO)_4$FeB7-2 | -0.307473 | -0.191047 | 0.116426 | 3.17 |
| $(CO)_4$FeB7-3 | -0.306865 | -0.255104 | 0.051761 | 1.41 |

| | | | | |
|---|---|---|---|---|
| $(CO)_2$FeB8-1 | -0.305535 | -0.218402 | 0.087133 | 2.37 |
| $(CO)_2$FeB8-2 | -0.296621 | -0.257056 | 0.039565 | 1.08 |
| $(CO)_2$FeB8-3 | -0.296958 | -0.235868 | 0.061090 | 1.66 |
| $(CO)_2$FeB8-4 | -0.293256 | -0.248759 | 0.044497 | 1.21 |
| $(CO)_2$FeB8-5 | -0.303768 | -0.255418 | 0.048350 | 1.32 |
| $(CO)_2$FeB8-6 | -0.287265 | -0.227453 | 0.059812 | 1.63 |
| $(CO)_3$FeB8-1 | -0.313565 | -0.265743 | 0.047822 | 1.30 |
| $(CO)_3$FeB8-2 | -0.310716 | -0.251122 | 0.059594 | 1.62 |
| $(CO)_4$FeB8-1 | -0.305884 | -0.235986 | 0.069898 | 1.90 |
| $(CO)_4$FeB8-2 | -0.311805 | -0.233291 | 0.078514 | 2.14 |
| $(CO)_2$FeB9-1 | -0.296493 | -0.237156 | 0.059337 | 1.61 |
| $(CO)_2$FeB9-2 | -0.314295 | -0.205571 | 0.108724 | 2.96 |
| $(CO)_2$FeB9-3 | -0.294937 | -0.227968 | 0.066969 | 1.82 |
| $(CO)_2$FeB9-4 | -0.295864 | -0.251199 | 0.044665 | 1.22 |
| $(CO)_2$FeB9-5 | -0.305344 | -0.248505 | 0.056839 | 1.55 |
| $(CO)_2$FeB9-6 | -0.297092 | -0.267352 | 0.029740 | 0.81 |
| $(CO)_2$FeB9-7 | -0.288716 | -0.268965 | 0.019751 | 0.54 |
| $(CO)_3$FeB9-1 | -0.312634 | -0.235484 | 0.077150 | 2.10 |
| $(CO)_3$FeB9-2 | -0.306121 | -0.241029 | 0.065092 | 1.77 |
| $(CO)_3$FeB9-3 | -0.312880 | -0.240318 | 0.072562 | 1.97 |
| $(CO)_3$FeB9-4 | -0.306469 | -0.232796 | 0.073673 | 2.00 |
| $(CO)_3$FeB9-5 | -0.310992 | -0.285933 | 0.025059 | 0.68 |
| $(CO)_3$FeB9-6 | -0.309741 | -0.287985 | 0.021756 | 0.59 |
| $(CO)_3$FeB9-7 | -0.313340 | -0.253312 | 0.060028 | 1.63 |
| $(CO)_4$FeB9-1 | -0.306323 | -0.201253 | 0.105070 | 2.86 |
| $(CO)_4$FeB9-2 | -0.302890 | -0.206480 | 0.096410 | 2.62 |
| $(CO)_4$FeB9-3 | -0.299436 | -0.254309 | 0.045127 | 1.23 |
| $(CO)_2$FeB10-1 | -0.302345 | -0.248246 | 0.054099 | 1.47 |
| $(CO)_2$FeB10-2 | -0.296248 | -0.240491 | 0.055757 | 1.52 |
| $(CO)_2$FeB10-3 | -0.301605 | -0.237294 | 0.064311 | 1.75 |
| $(CO)_2$FeB10-4 | -0.290535 | -0.245866 | 0.044669 | 1.22 |
| $(CO)_2$FeB10-5 | -0.283523 | -0.253850 | 0.029673 | 0.81 |
| $(CO)_3$FeB10-1 | -0.310976 | -0.259653 | 0.051323 | 1.40 |
| $(CO)_3$FeB10-2 | -0.309332 | -0.225265 | 0.084067 | 2.29 |
| $(CO)_3$FeB10-3 | -0.308964 | -0.211517 | 0.097447 | 2.65 |
| $(CO)_3$FeB10-4 | -0.306416 | -0.222987 | 0.083429 | 2.27 |
| $(CO)_3$FeB10-5 | -0.312282 | -0.241017 | 0.071265 | 1.94 |
| $(CO)_4$FeB10-1 | -0.308569 | -0.213950 | 0.094619 | 2.57 |
| $(CO)_4$FeB10-2 | -0.300534 | -0.240815 | 0.059719 | 1.63 |
| $(CO)_4$FeB10-3 | -0.300267 | -0.244060 | 0.056207 | 1.53 |
| $(CO)_4$FeB10-4 | -0.303810 | -0.256531 | 0.047279 | 1.29 |
| $(CO)_2$FeB11-1 | -0.299879 | -0.228847 | 0.071032 | 1.93 |
| $(CO)_2$FeB11-2 | -0.289513 | -0.234756 | 0.054757 | 1.49 |
| $(CO)_2$FeB11-3 | -0.301317 | -0.234984 | 0.066333 | 1.81 |

| | | | | |
|---|---|---|---|---|
| $(CO)_2$FeB11-4 | -0.282980 | -0.271818 | 0.011162 | 0.30 |
| $(CO)_3$FeB11-1 | -0.310402 | -0.227922 | 0.082480 | 2.24 |
| $(CO)_3$FeB11-2 | -0.308417 | -0.210607 | 0.097810 | 2.66 |
| $(CO)_4$FeB11-1 | -0.309658 | -0.194718 | 0.114940 | 3.13 |
| $(CO)_2$FeB12-1 | -0.302945 | -0.237513 | 0.065432 | 1.78 |
| $(CO)_2$FeB12-2 | -0.310780 | -0.225334 | 0.085446 | 2.33 |
| $(CO)_2$FeB12-3 | -0.293097 | -0.236866 | 0.056231 | 1.53 |
| $(CO)_2$FeB12-4 | -0.292832 | -0.230750 | 0.062082 | 1.69 |
| $(CO)_3$FeB12-1 | -0.308763 | -0.196304 | 0.112459 | 3.06 |
| $(CO)_3$FeB12-2 | -0.311706 | -0.207428 | 0.104278 | 2.84 |
| $(CO)_3$FeB12-3 | -0.313262 | -0.202837 | 0.110425 | 3.00 |
| $(CO)_4$FeB12-1 | -0.308753 | -0.193749 | 0.115004 | 3.13 |
